# IGF2BP3 enhances the mRNA stability of E2F3 by interacting with LINC00958 to promote endometrial carcinoma progression

**DOI:** 10.1038/s41420-022-01045-x

**Published:** 2022-06-08

**Authors:** Cuicui Wang, Fanfei Kong, Jian Ma, Jianing Miao, Peng Su, Hui Yang, Qing Li, Xiaoxin Ma

**Affiliations:** 1 Department of Obstetrics and Gynecology, Shengjing Hospital Affiliated to China Medical University, Shenyang, Liaoning Province 110000 PR China; 2 Key Laboratory of Gynecological Oncology of Liaoning Province, Department of Obstetrics and Gynecology, Shengjing Hospital Affiliated to China Medical University, Shenyang, Liaoning Province 110000 PR China; 3 Key Laboratory of Maternal-Fetal Medicine of Liaoning Province, Department of Obstetrics and Gynecology, Shengjing Hospital Affiliated to China Medical University, Shenyang, Liaoning Province 110000 PR China; 4 Medical Research Center, Shengjing Hospital Affiliated to China Medical University, Shenyang, Liaoning Province 110000 PR China

**Keywords:** Endometrial cancer, Mechanisms of disease

## Abstract

Long noncoding RNAs (lncRNAs) play important regulatory roles in a variety of pathological processes involving cancer. However, the exact molecular mechanisms of lncRNA regulation in endometrial carcinoma (EC) remain poorly defined. The aim of this study was to illustrate the mechanism of LINC00958 in regulating the function of IGF2BP3, an RNA binding protein involved in mRNA stability, and their clinical implications in EC. First, we investigated the clinical role of IGF2BP3 in EC and demonstrated its prognostic value. Loss-of-function and gain-of-function studies showed that IGF2BP3 promoted EC cell proliferation, migration and invasion. Then, we carried out RNA immunoprecipitation sequencing (RIP-seq) analysis, RNA pulldown and immunofluorescence-RNA fluorescence in situ hybridization to identify LINC00958 that interacted with IGF2BP3 in the cytoplasm of EC cells. Rescue experiments indicated that knockdown of LINC00958 partially offset the EC cell progression mediated by IGF2BP3. After that, RNA sequencing was used to screen out the downstream genes of IGF2BP3 and LINC00958. The results revealed that IGF2BP3 upregulated E2F3 expression by interacting with LINC00958. Furthermore, RNA stability assays demonstrated that silencing LINC00958 partially rescued the IGF2BP3-mediated promoting effect on the mRNA stability of E2F3. Collectively, this study suggests that LINC00958, as an oncogene, assists IGF2BP3 in stabilizing E2F3 mRNA and ultimately promotes EC progression, providing a promising therapeutic target for patients with EC.

## Introduction

Endometrial carcinoma is the most common malignant tumor of the female reproductive system in developed countries [1, 2]. Standard therapeutic approaches consist of surgical resection, chemotherapeutic, and radiotherapy. For advanced endometrial carcinoma, the efficacy of these therapies remains limited [3–5]. Hence, it is important to clarify the cellular and molecular mechanisms underlying EC and identify potential therapeutic targets for this lethal disease.

Long noncoding RNAs (lncRNAs), referred to as transcripts of noncoding RNAs over 200 nucleotides in length, are involved in various tumorigenesis processes [6–9]. We previously demonstrated that lncRNA-MIR210HG, as a molecular sponge of miR-337-3p/miR-137, regulated HMGA2 expression and promoted EC progression [10]. In addition to the classic “microRNA (miRNA) molecular sponge” theory, lncRNA-SNHG7 stabilizes autophagy-related genes (ATG5 and ATG12) and enhances docetaxel resistance in lung adenocarcinoma by recruiting human antigen R (HuR) [11]. More interestingly, lncRNA-LNC942 directly recruited the methyltransferase METTL14 and stabilized downstream targets of METTL14 via m6A methylation modification [12].

The insulin-like growth factor mRNA-binding protein family (IGF2BPs) consists of three proteins (IGF2BP1-3) [13]. IGF2BP3, also known as IMP3, CT98, or VICKZ3, has been reported as a carcinogenic-related protein in many studies of diverse cancers [14–16]. An important study revealed that IGF2BPs acted as N6-methyladenosine (m6A) readers to recognize m6A-modified mRNAs and enhance their stability, ultimately affecting gene expression [17]. Consistently, in clear cell renal cell carcinoma, lncRNA DMDRMR bound IGF2BP3 to stabilize CDK4 and three extracellular matrix components (COL6A1, LAMA5, and FN1) by specifically enhancing IGF2BP3 activity in a m6A-dependent manner [18]. However, our understanding of lncRNAs modulating IGF2BP3 function in EC progression is still limited.

In this research, we demonstrated that IGF2BP3 facilitated EC development and subsequently screened out LINC00958 interacting with IGF2BP3 by RIP-seq. We further revealed that LINC00958 could promote the ability of IGF2BP3 to enhance the mRNA stability of E2F3. The results of our study illustrate novel insights into the regulation of EC progression mediated by lncRNAs and IGF2BP3, which provide new ideas for therapeutic strategies in EC.

## Results

### IGF2BP3 was upregulated in EC and predicted poor overall survival

**Fig. 1:**
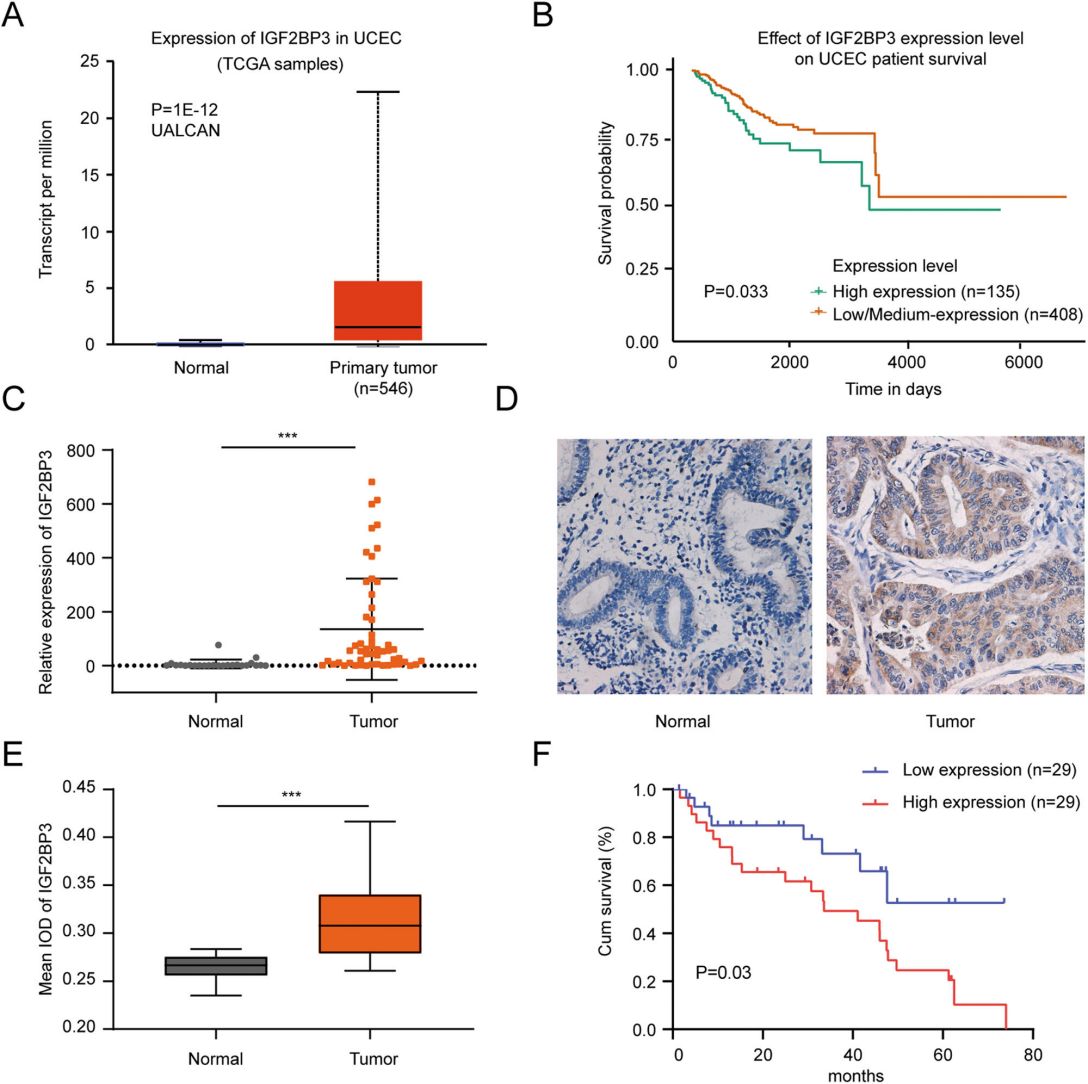
IGF2BP3 was upregulated in EC and predicted poor overall survival. **A** IGF2BP3 mRNA levels in EC obtained from TCGA dataset. **B** Kaplan–Meier analysis of overall survival in EC patients with high and low expression levels of IGF2BP3 from TCGA datasets. **C** The mRNA expression levels of IGF2BP3 in clinical EC tissues and normal endometrial tissues. **D** Representative images of IGF2BP3 protein expression in human endometrial tissues (left: normal endometrial tissue, right: EC tissue) (×200). **E** Mean IOD of IGF2BP3 after immunohistochemistry staining in human endometrial tissues. **F** Kaplan–Meier estimates of overall survival in 58 patients with EC according to the expression level of IGF2BP3 in IHC (patients were divided into low- and high-expression groups by the median of mean IOD, *P* = 0.03). *** *P* < 0.001. Data, mean ± S.D. All experiments were independently repeated with at least three replicates. Abbreviation: EC endometrial carcinoma, TCGA The Cancer Genome Atlas, UCEC Uterine corpus endometrial carcinoma, IOD Integrated optical density.

To investigate the role of IGF2BP3 in EC, we first used data retrieved from the UALCAN platform [19]. and found that IGF2BP3 mRNA expression in tumor tissue was increased compared with that in normal endometrial tissue in The Cancer Genome Atlas (TCGA) dataset (Fig. 1A). Moreover, a Kaplan–Meier plot obtained from the UALCAN platform showed that a high IGF2BP3 expression level had a negative effect on patient survival (Fig. 1B). To confirm the bioinformatics-based results, we measured the expression of IGF2BP3 in 53 EC tissues and 24 normal endometrial tissues by RT–qPCR. Consistently, IGF2BP3 expression was increased in EC tissues (Fig. 1C). Next, to explore the correlation between IGF2BP3 mRNA expression and clinicopathological parameters, we applied the median expression level of IGF2BP3 to split 53 EC patients into high and low expression groups. The results showed that high IGF2BP3 expression was associated with advanced clinical stages of EC, high tumor grade, vascular invasion, and distant metastasis (Table S1). Finally, to further assess the protein expression of IGF2BP3 in EC patients, we carried out immunohistochemical staining of paraffin-embedded clinical specimens (Fig. 1D). The mean integrated optical density (IOD) values demonstrated that the protein level of IGF2BP3 was also upregulated in EC tissue compared to normal endometrial tissue (Fig. 1E). Moreover, high IGF2BP3 expression was clearly related to poor prognosis in EC patients (Fig. 1F).

### IGF2BP3 could promote EC cells progression in vitro

**Fig. 2:**
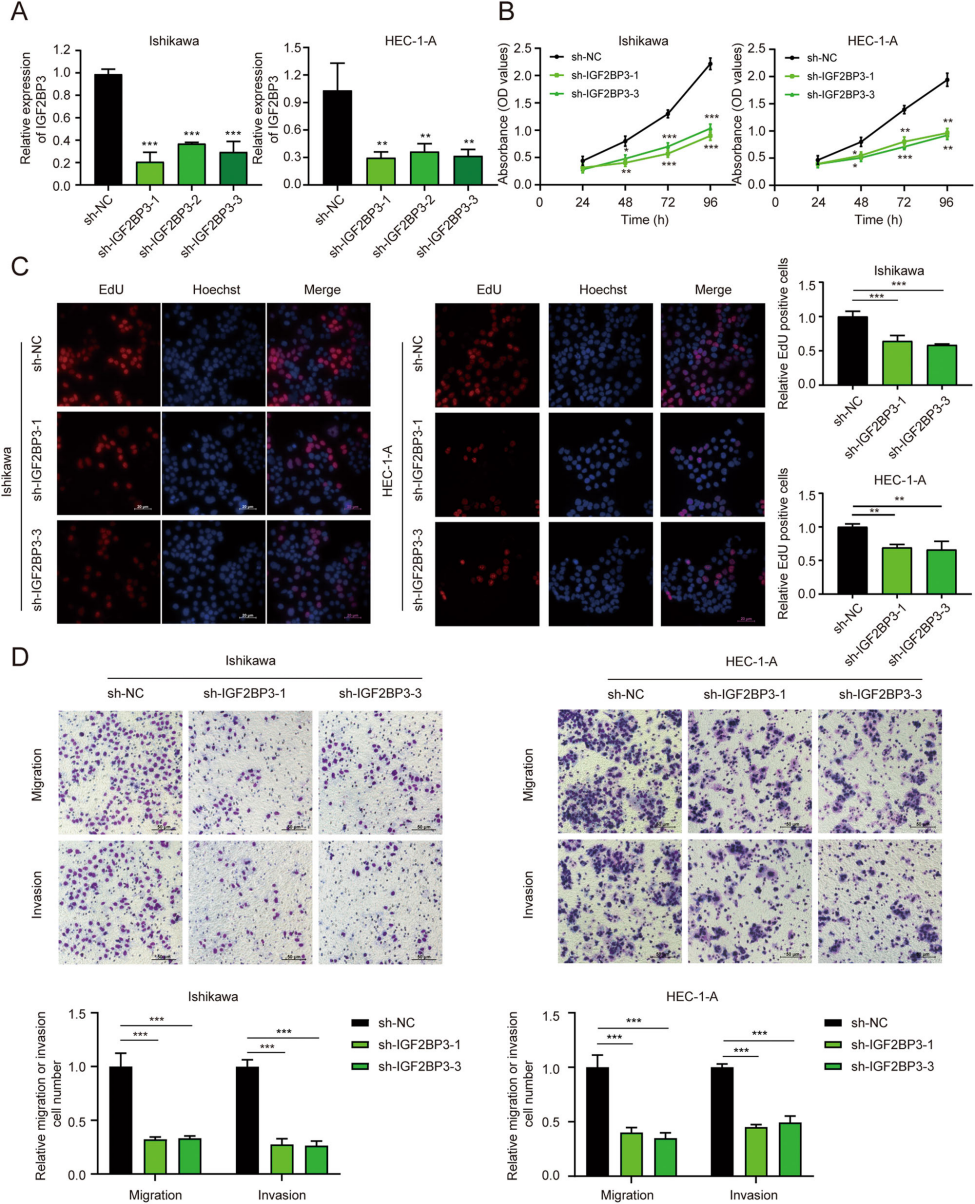
Knockdown of IGF2BP3 inhibited EC cell progression in vitro. **A** The mRNA expression level of IGF2BP3 was knocked down by three shRNAs in Ishikawa and HEC-1-A cells. **B** and **C** CCK-8 assays (**B**) and EdU assays (**C**) were employed to evaluate the cell proliferation ability in IGF2BP3-silenced EC cells. **D** Transwell assays were conducted to assess the migration and invasion of IGF2BP3-silenced EC cells. The quantification results are on the near side of their representative images. *P < 0.05, **P < 0.01, ***P < 0.001. Data, mean ± S.D. All experiments were independently repeated with at least three replicates.

To investigate the oncogenic functions of IGF2BP3 in EC, we stably silenced the expression of IGF2BP3 in Ishikawa and HEC-1-A cell lines and confirmed their knockdown efficiency (Fig. 2A, Fig. S1B). The results demonstrated that stable knockdown of IGF2BP3 remarkably restained EC cell proliferation, migration, and invasion (Fig. 2B, C, D). Furthermore, we overexpressed IGF2BP3 in Ishikawa and HEC-1-A cells and detected their efficiency (Fig. S1A, C). As expected, overexpression of IGF2BP3 obviously facilitated EC cell proliferation, migration, and invasion (Fig. S1D, E, F). Together, these data indicated that IGF2BP3 could boost the progression of EC in vitro.

### IGF2BP3 exerted its tumor-promoting effects by interacting with LINC00958 in the cytoplasm of EC cells

**Fig. 3:**
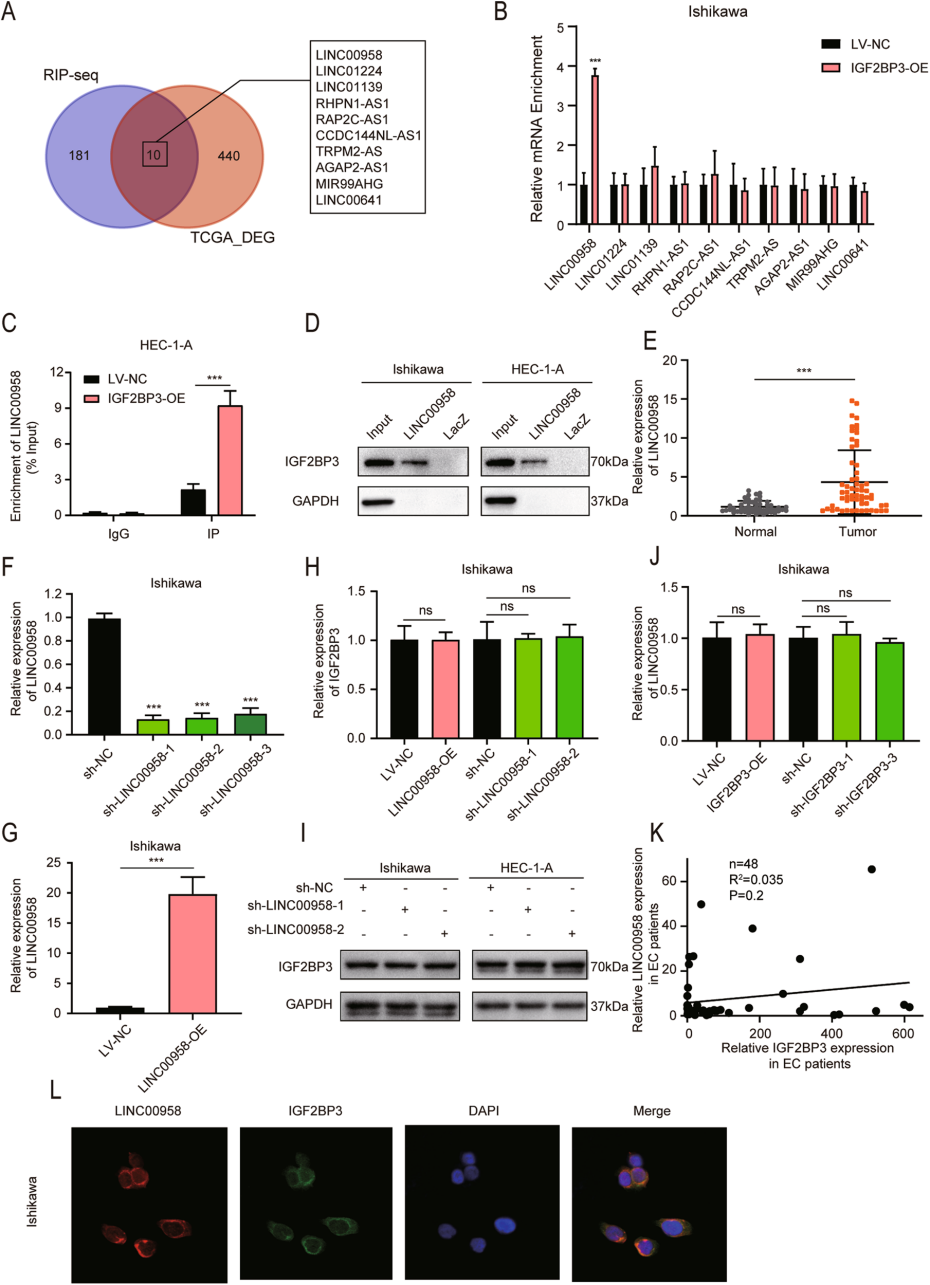
IGF2BP3 could interact with LINC00958 in the cytoplasm of EC cells. **A** IGF2BP3-binding lncRNAs were established by overlap of RIP-seq and differentially expressed genes obtained from TCGA database in UCEC. **B** RIP-qPCR assays showed the interaction between IGF2BP3 and ten potential lncRNAs in Ishikawa cells with stable IGF2BP3 overexpression or negative control. **C** RIP-qPCR assays displayed the enrichment of LINC00958 in HEC-1-A cells overexpressing or not overexpressing IGF2BP3. **D** Western blot assays showed the specific interaction between IGF2BP3 and LINC009958 using biotinylated LINC00958 or lacZ DNA probes from protein extract of Ishikawa and HEC-1-A cells. **E** LINC00958 expression levels in 60 human EC specimens compared to 52 normal endometrial specimens. **F** and **G** The expression levels of LINC00958 after RNAi (**F**) or overexpression (**G**) lentivirus infection in Ishikawa cells. **H** RT–qPCR assays showed that IGF2BP3 was not regulated in either LINC00958-overexpressing or LINC00958-silenced Ishikawa cells. **I** Western blot assays revealed IGF2BP3 expression in LINC00958-silenced Ishikawa and HEC-1-A cells. **J** RT–qPCR analysis showed that LINC00958 was also not regulated in either IGF2BP3-overexpressing or IGF2BP3-silenced Ishikawa cells. **K** Correlation analysis between LINC00958 and IGF2BP3 in 48 EC tissues by detecting mRNA expression. **L** RNA-FISH coupled to immunofluorescence staining assay indicated that LINC00958 colocalized with IGF2BP3 in Ishikawa cells (×800). ***P < 0.001, “ns”, no statistical significance. Data, mean ± S.D. All experiments were independently repeated with at least three replicates.

To explain the possible molecular mechanism underlying the oncogenic roles of IGF2BP3 in EC, we first employed RNA immunoprecipitation (RIP) assays to identify the lncRNAs involved in the regulation of IGF2BP3 function. One hundred ninety-one candidates as IGF2BP3-interacting lncRNAs were identified in the RIP-seq results. Then, we exploited RNA-seq web tools in Lnc2Cancer 3.0 [20] to obtain 450 differentially expressed lncRNAs in uterine corpus endometrial cancer (UCEC) by taking the intersection of three selectable statistical methods with a stringent filter of log_2_FC > 2.0 and FDR < 0.01. The results indicated that ten differentially expressed lncRNAs possessed the potential capacity to bind with IGF2BP3 in EC cells (Fig. 3A).

To verify the lncRNAs that could actually bind with IGF2BP3, we overexpressed IGF2BP3 in EC cells and found that only the interaction between IGF2BP3 and LINC00958 was increased by RIP-qPCR, which meant that LINC00958 specifically bound to the IGF2BP3 protein (Fig. 3B, C). Moreover, RNA pulldown assays with biotin-labeled LINC00958 RNA probes or negative control lacZ were performed. Remarkably, the amount of IGF2BP3 pulled down by LINC00958 RNA probes was increased in EC cells compared to the negative control (Fig. 3D).

It has been reported that LINC00958 has oncogenic properties in EC and predicts poor overall survival [21, 22]. As confirmed by our RT–qPCR results, LINC00958 was obviously elevated in EC tissues (Fig. 3E). After LINC00958 overexpression and RNAi lentivirus were stably infected into EC cells, we evaluated the efficiency (Fig. 3F, G, Fig. S2A, B). Interestingly, although LINC00958 bound with IGF2BP3, they actually regulated neither the mRNA expression levels nor the protein expression levels of each other in both Ishikawa and HEC-1-A cells (Fig. 3H–J, Fig. S2C–E). In addition, the expression level of IGF2BP3 was not correlated with the expression of LINC00958 in 48 EC patients (Fig. 3K). Immunofluorescence-RNA FISH assays displayed the colocalization of LINC00958 and IGF2BP3 in the cytoplasm of EC cells (Fig. 3L, Fig. S2F). This implied that IGF2BP3 mainly interacted with LINC00958 in the cytoplasm of EC cells.

**Fig. 4:**
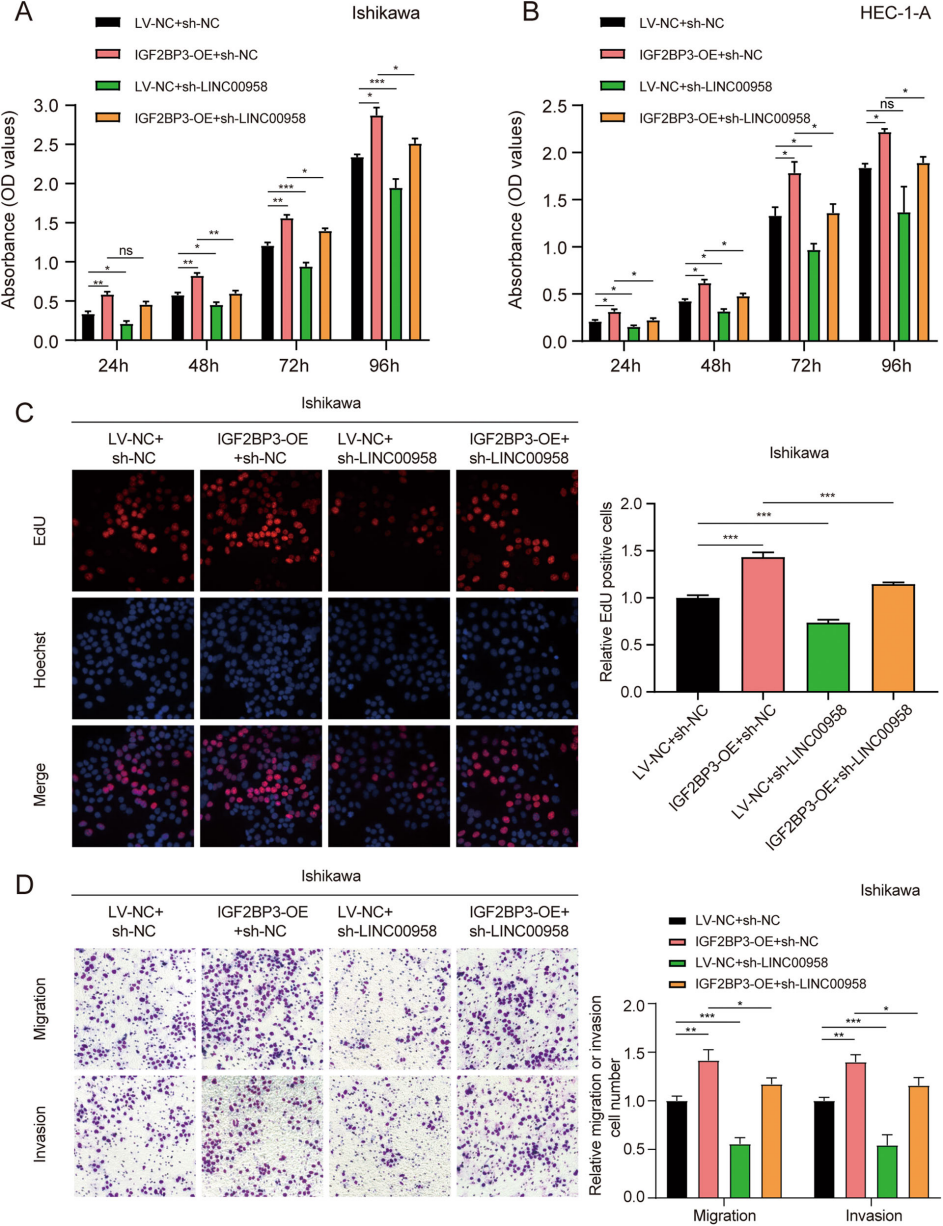
Knockdown of LINC00958 offset the tumor-promoting role of IGF2BP3 in EC. **A** and **B** CCK-8 assays showed the proliferation of Ishikawa cells and HEC-1-A cells upon ectopic expression of IGF2BP3 combined with LINC00958-silenced expression. **C** EdU assays indicated the proliferation ability of Ishikawa cells stably transfected. **D** Migration and invasion assays showed invasion in Ishikawa cells stably transfected. On the right side of representative images are their quantification results. *P < 0.05, **P < 0.01, ***P < 0.001, “ns”, no statistical significance. Data, mean ± S.D. All experiments were independently repeated with at least three replicates.

Functional assays demonstrated that the increased ability to proliferate, migrate and invade in IGF2BP3-overexpressing EC cells was partially rescued by the knockdown of LINC00958 (Fig. 4A–D, Fig. S3A, B). Collectively, all these data indicated that LINC00958 might exert its tumor-promoting effects by binding with IGF2BP3, not regulating it, in EC.

### IGF2BP3 upregulated E2F3 expression by interacting with LINC00958 in EC cells

**Fig. 5:**
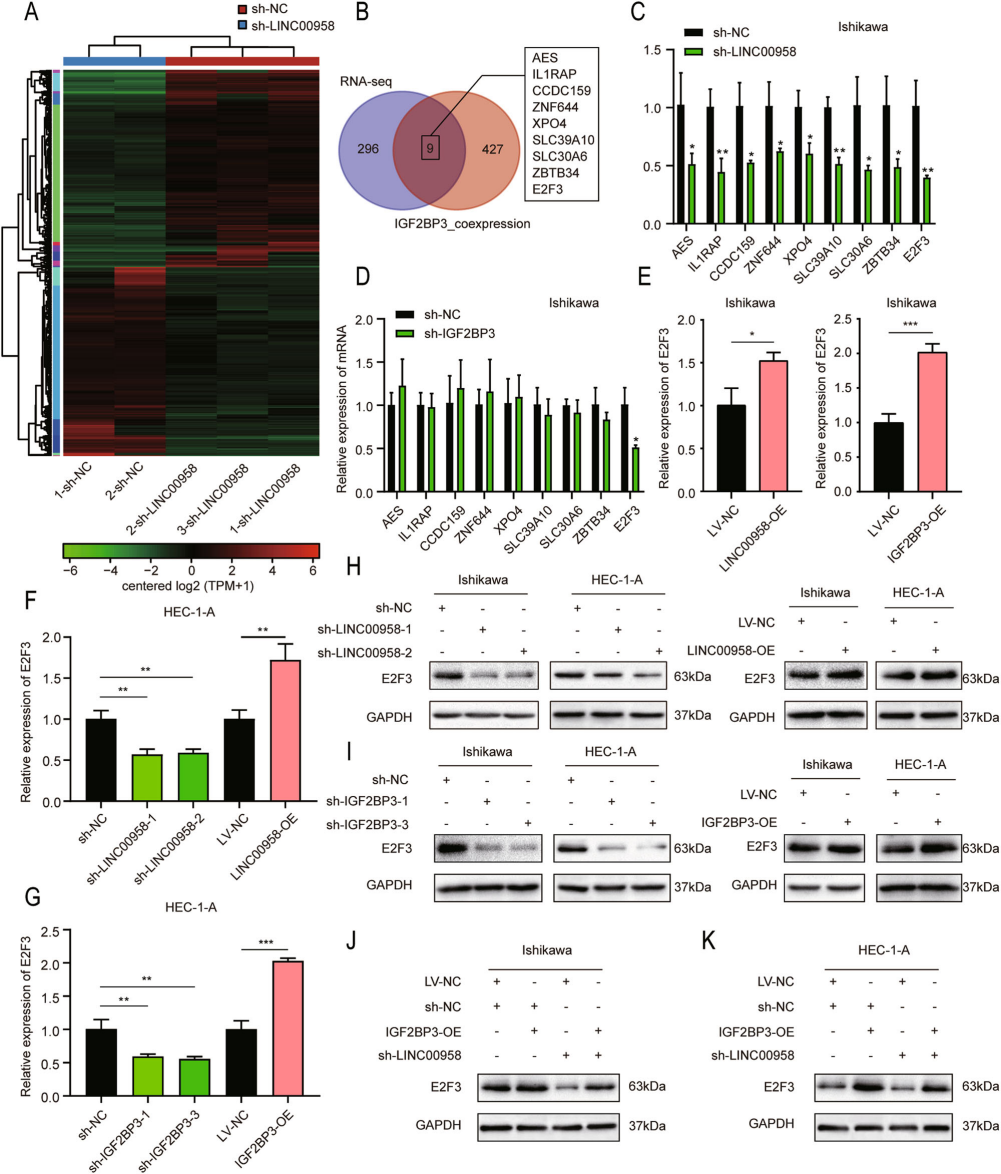
IGF2BP3 upregulated E2F3 expression by interacting with LINC00958 in EC cells. **A** Heatmap of RNA-seq displaying the differentially expressed genes in LINC00958-silenced Ishikawa cells and LINC00958-nonsilenced cells. **B** Venn diagram showing nine potential downstream mRNAs by overlap of RNA-seq and coexpressed genes with IGF2BP3 in UCEC obtained from TCGA dataset. **C**, **D** RT–qPCR assays showed the expression of the nine potential downstream mRNAs in LINC00958-silenced (**C**) and IGF2BP3-silenced (**D**) Ishikawa cells. **E** RT–qPCR confirmed the expression of E2F3 in both LINC00958-overexpressing and IGF2BP3-overexpressing Ishikawa cells. **F~I** RT–qPCR and western blot assays showed the expression of E2F3 in LINC00958–overexpressing and LINC00958-silenced HEC-1-A cells (**F**, **H**) as well as IGF2BP3–overexpressing and IGF2BP3-silenced HEC-1-A cells (**G**, **I**). **J** and **K** Western blot assays revealed the expression of E2F3 in Ishikawa (**J**) and HEC-1-A cells (**K**) stably transfected with negative control or LINC00958-silenced lentivirus and cotransfected with IGF2BP3–overexpressing or corresponding control lentivirus. *P < 0.05, **P < 0.01, ***P < 0.001. Data, mean ± S.D. All experiments were independently repeated with at least three replicates.

To confirm the downstream genes of LINC00958, we first employed RNA sequencing (RNA-seq) to identify the differentially expressed genes when LINC00958 was silenced in Ishikawa cells. A total of 305 mRNAs were regulated after LINC00958 knockdown (fold change>2, *P* < 0.05) (Fig. 5A). Subsequently, we exploited the TCGA database and found 436 mRNAs coexpressed with IGF2BP3 by Pearson correlation analysis with a filter of correlation coefficient |R | > 0.4. Among these mRNAs, nine mRNAs were differentially expressed in LINC00958-silenced Ishikawa cells and coexpressed with IGF2BP3 in the TCGA dataset (Fig. 5B). We further confirmed the expression of nine mRNAs in both LINC00958-silenced and IGF2BP3-silenced Ishikawa cells by RT–qPCR. Only the E2F3 expression level was affected in both cell lines (Fig. 5C, D). Consistently, E2F3 was upregulated in LINC00958-overexpressing cells and IGF2BP3-overexpressing cells (Fig. 5E-I). Finally, we carried out rescue experiments to clarify the interaction between LINC00958 and IGF2BP3 in E2F3 expression regulation. The results indicated that the knockdown of LINC00958 remarkably counteracted the IGF2BP3-induced increase in E2F3 expression in both Ishikawa and HEC-1-A cells (Fig. 5J, K). Taken together, these data demonstrated that IGF2BP3 could regulate E2F3 expression by interacting with LINC00958 in EC cells.

### IGF2BP3 exerted its tumor-promoting effects via E2F3 in EC

**Fig. 6:**
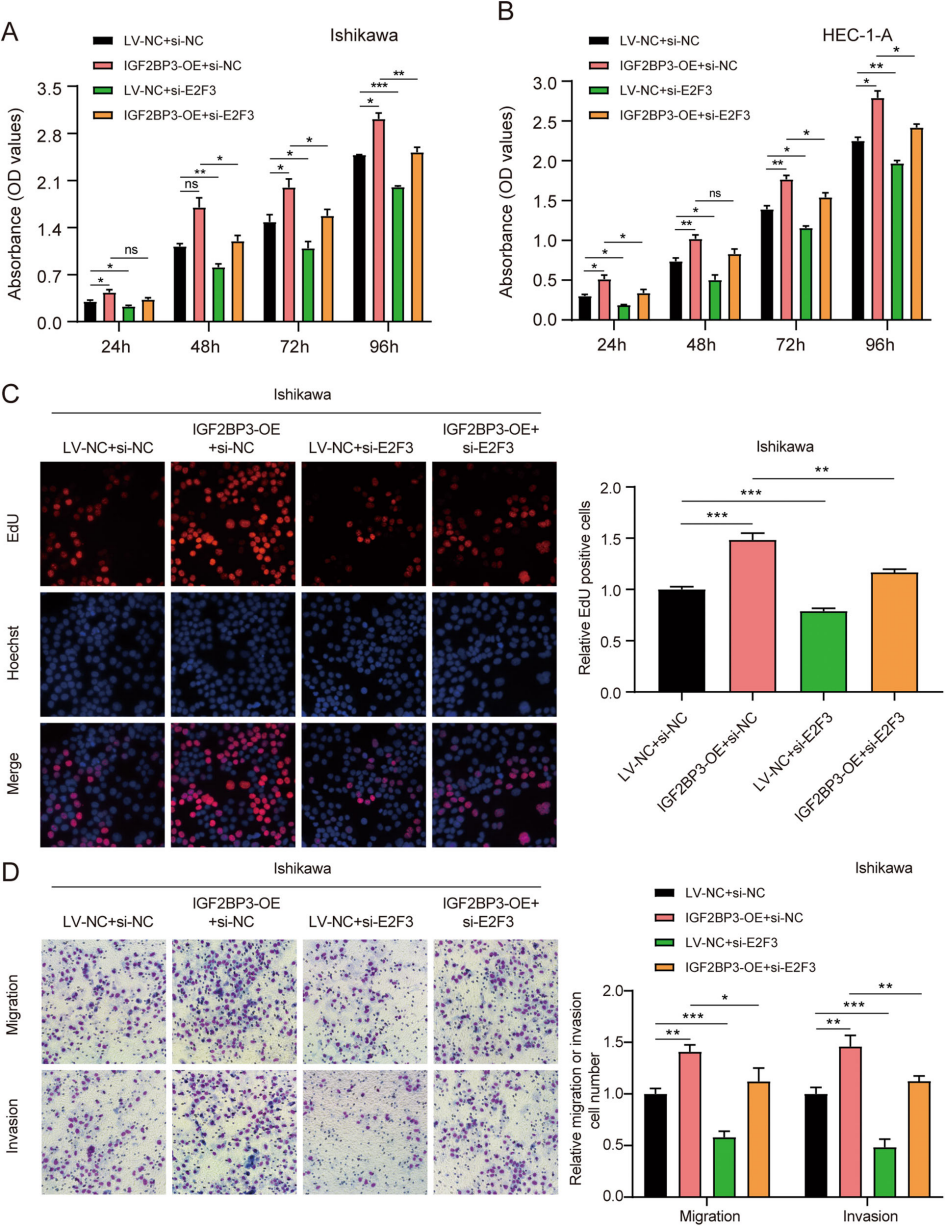
IGF2BP3 exerted its tumor-promoting effects via E2F3 in EC. **A** and **B** CCK-8 assays showed the proliferation of Ishikawa cells and HEC-1-A cells overexpressing IGF2BP3 combined with E2F3-silenced expression. **C** EdU assays displayed the proliferation ability of Ishikawa cells overexpressing IGF2BP3 combined with E2F3-silenced expression. **D** Transwell assays indicated the migration and invasion of Ishikawa cells overexpressing IGF2BP3 combined with E2F3-silenced expression. On the right side of representative images are their quantification results. *P < 0.05, **P < 0.01, ***P < 0.001, “ns”, no statistical significance. Data, mean ± S.D. All experiments were independently repeated with at least three replicates.

Several studies have demonstrated that E2F3 is a cancer-promoting gene in EC [23, 24]. To further clarify the results, we transfected siRNAs into EC cells and tested the efficiency (Fig. S4A). Silencing the expression of E2F3 significantly suppressed EC cell proliferation, migration, and invasion (Fig. S4B–D). Notably, rescue experiments showed that the knockout of E2F3 significantly offset the IGF2BP3-mediated promotion of proliferation and migration in EC cells (Fig. 6A–D, Fig. S5A, B). Collectively, the results indicated that IGF2BP3 promotes EC progression via E2F3 in vitro.

### IGF2BP3 could promote the growth of EC in vivo

**Fig. 7:**
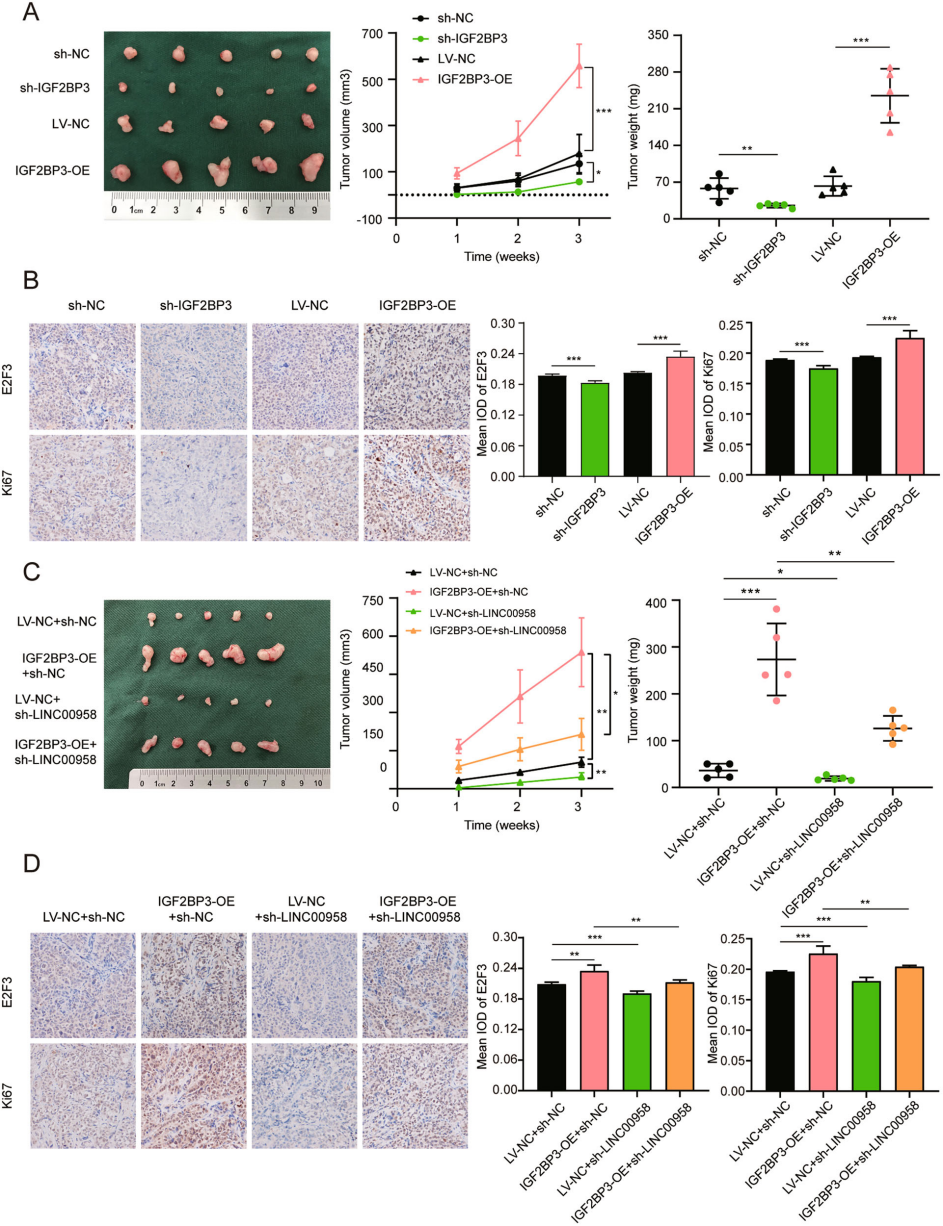
IGF2BP3 could promote EC cells growth in vivo. **A**, **C** Representative pictures (left), the growth curve in vivo (middle), and the weight at the endpoints (right) of xenograft tumors in nude mice (*n* = 5 per group). **B**, **D** Representative images and quantification results of immunohistochemistry staining of xenograft tumors were used to display the expression of E2F3 and Ki67 (× 200). *P < 0.05, **P < 0.01, ***P < 0.001. Data, mean ± S.D.

To confirm the role of IGF2BP3 in EC proliferation in vivo, we established subcutaneous xenograft nude mouse models. Consistent with the in vitro results, knockdown or overexpression of IGF2BP3 in HEC-1-A cells contributed to the corresponding decrease or increase in the growth of subcutaneous xenograft tumors (Fig. 7A). Immunohistochemical experiments showed that E2F3 and Ki67 expression levels were downregulated when IGF2BP3 was silenced and upregulated when IGF2BP3 was overexpressed (Fig. 7B). Moreover, knockdown of LINC00958 partially rescued IGF2BP3-induced proliferation of subcutaneous xenograft tumors (Fig. 7C). In addition, the elevated E2F3 and Ki67 expression in IGF2BP3-overexpressing xenograft tumors was ameliorated by knockdown of LINC00958 (Fig. 7D). Together, in vivo experiments revealed that IGF2BP3 promoted EC cell proliferation and exerted its oncogenic role via LINC00958.

### IGF2BP3 promoted the mRNA stability of E2F3 with the help of LINC00958

**Fig. 8:**
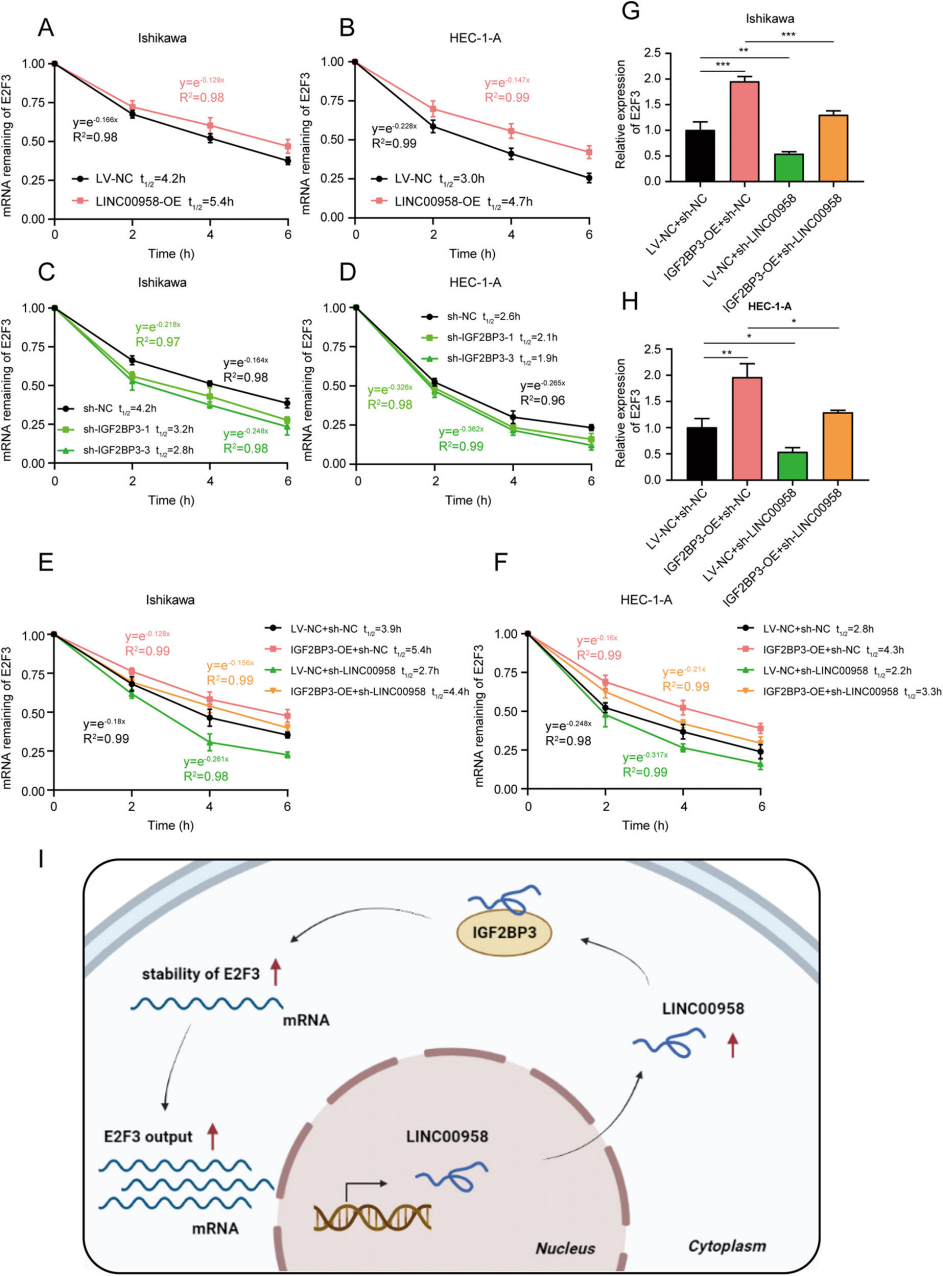
IGF2BP3 promote E2F3 mRNA stability via LINC00958. **A**, **B** Prolonged E2F3 mRNA half-life by overexpressing LINC00958 in EC cells. **C**, **D** Shortened E2F3 mRNA half-life by knocking down IGF2BP3 in EC cells. **E**, **F** Silencing LINC00958 partially counteracted the improved mRNA stability of E2F3 caused by overexpression of IGF2BP3 in EC cells. **G**, **H** RT–qPCR assays showed E2F3 expression levels in IGF2BP3–overexpressing EC cells combined with E2F3-silenced expression. **I** Schematic diagram created with BioRender.com illustrating the role of LINC00958 and IGF2BP3 in EC cells. *P < 0.05, **P < 0.01, ***P < 0.001. Data, mean ± S.D. All experiments were independently repeated with at least three replicates.

Mounting evidence indicates that IGF2BPs, as RNA binding proteins, improve the stability and storage of many target mRNAs, such as MYC, and thus influence gene expression [17, 25, 26]. LncRNAs are also involved in gene regulation by affecting mRNA stability [27–29]. Thus, we further aimed to elucidate the mechanisms underlying LINC00958 regulation of IGF2BP3 function by RNA stability assays. The results showed that both knockdown of IGF2BP3 and overexpression of LINC00958 affected the mRNA stability of E2F3 in HEC-1-A and Ishikawa cells (Fig. 8A–D). Moreover, rescue experiments revealed that the elevated mRNA stability of E2F3 in IGF2BP3-overexpressing EC cells was restored by LINC00958 knockdown (Fig. 8E, F) and consequently affected E2F3 mRNA expression output (Fig. 8G, H). Together, our findings supported that LINC00958 was required for IGF2BP3-mediated prolongation of the E2F3 mRNA half-life.

## Discussion

The expression of IGF2BP3 in EC tissues has been shown to be elevated compared to that in normal endometrial tissues, especially in endometrial serous carcinoma and clear cell carcinoma [30, 31]. However, the function of IGF2BP3 in endometrial carcinoma has not been studied. In this study, we ascertained that IGF2BP3 was upregulated in EC tissues and promoted EC cell progression, which indicated the potential diagnostic or prognostic value of IGF2BP3 in EC.

As an RNA binding protein, IGF2BP3 modulates the stability of multiple mRNAs to facilitate cancer development. In triple-negative breast cancer, IGF2BP3 and IGF2BP2 synergistically recruited CNOT1 complex, reduced the stability of progesterone receptor and ultimately promoted the migration and metastasis of breast cancer [14]. High IGF2BP3 expression enhanced melanoma cell proliferation by accelerating the degradation of EIF4E-BP2 mRNA and promoting the translation of EIF4E [32]. Conversely, IGF2BP3 bound and enhanced the mRNA stability of HMGA2 in the process of melanoma migration and invasion [33]. Recently, a growing body of research has suggested that long noncoding RNAs interact with IGF2BP3 and have substantial effects on cancer progression [34–36]. LncRNA CDKN2B-AS1 was stabilized by IGF2BP3, recruited CBP and SMYD3 and activated NUF2 transcription in renal clear cell carcinoma [37]. IGF2BP3 could promote the interaction between LINC01138 and PRMT5 by combining with LINC01138 and ultimately increase PRMT5 stability in hepatocellular carcinoma [16].

In this study, we applied the RIP-seq assay to identify LINC00958 that interacted with IGF2BP3. LINC00958 was originally recognized as a cancer-promoting gene in bladder cancer [38]. Several studies have shown that LINC00958 is upregulated in many malignancies [39–41]. Regarding endometrial carcinoma, a recent study demonstrated that LINC00958 played an oncogenic role in EC cell proliferation and metastasis by regulating the miR-145-3p/TCF4 axis [21]. Similarly, another study revealed the role of LINC00958/miR-3174/PHF6 axis in the progression of EC [22]. From a new perspective, our results revealed that knockdown of LINC00958 rescued the oncogenic effect of IGF2BP3 in EC proliferation and migration by binding with it in the cytoplasm and impairing the mRNA stability of the downstream oncogene but did not regulate the expression level of IGF2BP3.

To further explore the downstream genes of the LINC00958/IGF2BP3 axis in EC, we combined RNA sequencing results and genes coexpressed with IGF2BP3 from the TCGA database and discovered that E2F3 was the downstream effector. E2F3, as a member of a small family of transcription factors, is involved in the tumorigenesis of many malignancies [42–44]. Some studies have identified E2F3 as an oncogene that promotes the progression of EC [24, 45]. HOXB9, as a transcription factor of the HOX family, facilitates E2F3 expression by binding to its promoter directly in EC cells [24]. MiR-152 targeted E2F3, restrained its expression and then inhibited endometrial carcinogenesis [45]. Consistently, our data revealed that reducing E2F3 suppressed the proliferation and migration of EC cells and that knockdown of E2F3 offset IGF2BP3-induced oncogenic effects. Hence, it shed new light on the oncogenic role of IGF2BP3 in EC.

A prominent study reported that IGF2BP1/2/3 targeted many mRNA transcripts by recognizing the consensus sequence of GG (m6A) C, thus enhancing the stability of target mRNA and regulating gene expression [17]. RNA stability assays in our study indicated that, with the help of LINC00958, IGF2BP3 prolonged the half-life of E2F3 mRNA and finally regulated E2F3 expression levels. Our RIP-seq analysis of IGF2BP3 in Ishikawa cells revealed that E2F3 mRNA also bound to IGF2BP3. To elucidate whether IGF2BP3-induced E2F3 regulation is m6A-dependent, we predicted m6A modification sites based on the consensus “GGAC” sequence via the website SRAMP (http://www.cuilab.cn/sramp/) [46]. Two sites were predicted from the website. Next, we inserted a 415-nt wild-type sequence of E2F3 containing two binding sites into the dual-luciferase reporter plasmid. Unfortunately, the results showed that the relative luciferase activity of the wild-type reporter was not changed after IGF2BP3 silencing (data not shown). This means that IGF2BP3 enhanced the stability of E2F3 mRNA independent of m6A modification. In addition, in the rescue experiment, knocking down LINC00958 could only partially offset the EC cell progression mediated by IGF2BP3, and the elevated mRNA stability of E2F3 in IGF2BP3-overexpressing EC cells was also partially restored by LINC00958 knockdown. These results meant that IGF2BP3 might not only increase the stability of E2F3 mRNA by binding to LINC00958, but also directly affect the stability of E2F3 independently of LINC00958 by binding to the 3’UTR region of E2F3. How LINC00958 interacts with IGF2BP3 upon binding to affect the stability of downstream effector mRNA is something we will study in depth in the future.

In general, this study revealed that IGF2BP3 could promote the proliferation, migration, and invasion of endometrial carcinoma. Mechanistically, IGF2BP3 played a moderating role by altering the stability of E2F3 mRNA. Moreover, LINC00958 helped IGF2BP3 complete its function through binding with it without changing its expression level. The results of our study illustrated novel insights into the regulation of endometrial carcinoma progression mediated by lncRNAs and IGF2BP3, which highlighted the value of IGF2BP3 combined with LINC00958 as predictors for prognosis and therapeutic candidates in EC.

## Materials and methods

### Human tissue specimens

All tissue specimens were taken from patients who underwent curettage or hysterectomy at Shengjing Hospital Affiliated with China Medical University (Shenyang, Liaoning, China) from January 2011 to December 2019. They included 65 fresh endometrial carcinomas and 52 normal endometrial tissues, 58 paraffin-embedded endometrial carcinomas and 20 normal endometrial tissues. All of the patients received no preoperative radiation or chemotherapy treatments. All the samples were diagnosed by at least two qualified histopathologists according to the staging criteria of the International Federation of Obstetrics and Gynecology (2009). The study was approved by the relevant ethics committee of our institution, and each patient gave informed consent to our research.

### Immunohistochemistry

Serial 4 μm thick paraffin sections were used for immunohistochemistry with antibodies specific for IGF2BP3 (ab179807, Abcam, USA, 1:100), E2F3 (DF12390, Affinity Biosciences, Changzhou, China, 1:50), and Ki67 (AF1738, Beyotime, Shanghai, China, 1:50). A streptavidin peroxidase (SP) immunohistochemical kit (Maixin, Fuzhou, China) was used according to the instructions. Images were taken with a Nikon microscope (Eclipse Ci, Nikon Ltd, Japan). Image-Pro Plus 6.0 was employed to analyse the levels of protein expression by calculating the values of mean integrated optical density (integrated optical density/area) for statistical analysis [47].

### Cell transfection and stable cell line construction

Lentiviruses overexpressing IGF2BP3 or LINC00958 and hairpin-derived small RNAs (shRNAs) targeting IGF2BP3 or LINC00958 were purchased from Shanghai GeneChem of China. Seventy-two hours after lentivirus infection, 2.5 µg/ml puromycin was applied to screen for stably infected cells. Small interfering RNAs (siRNAs) (GenePharma, Suzhou, China) targeting E2F3 were introduced into EC cells by JetPRIME transfection reagent (Polyplus Transfection, Illkirch, France). The target sequences of shRNAs and siRNAs are listed in Table S2.

### Western blot

The cells were harvested and lysed on ice with RIPA lysis buffer (Beyotime, Shanghai, China). The BCA protein concentration determination kit (Beyotime, Shanghai, China) was used to determine the protein concentration. After boiling for 10 min, proteins were separated by 10% SDS–PAGE and then transferred to PVDF membranes (Millipore, Bedford, MA, USA). The membrane was subsequently blocked for 1 h in 5% fat-free milk, incubated with the primary antibodies for 1.5 h at 25 °C, and then incubated with the horseradish peroxidase-labeled secondary antibody (Zhongshan Jinqiao, Beijing, China, 1:3000) for 1 h at 25 °C. After repeated washing with TBST, the membranes were developed by an enhanced chemiluminescence kit (Vazyme, Nanjing, China). The primary antibodies used were as follows: GAPDH (AF7021, Affinity Biosciences, Changzhou, China, 1:3000), IGF2BP3 (ab179807, Abcam, USA, 1:1000), and E2F3 (27615-1-AP, Proteintech, Wuhan, China, 1:500).

### RNA immunoprecipitation (RIP)

We performed an RIP assay with an RNA Immunoprecipitation (RIP) Kit (Bes5101, BersinBio, Guangzhou, China) according to the manufacturer’s protocol. Cells were lysed and incubated with 4 µg antibodies against IGF2BP3 (ab177477, Abcam, USA) overnight at 4 °C with constant rotation. IgG was used as a control. Input and coimmunoprecipitated RNAs were extracted, reverse-transcribed and analysed by qPCR.

### RNA pulldown assay

In human Ishikawa and HEC-1-A cells, RNA pulldown assays were conducted according to the instructions of an RNA–protein pulldown kit (NO. 20164, Thermo Scientific, Rockford, IL, USA). Biotin-labeled LINC00958 was synthesized by GenePharma (Suzhou, China) and interacted with cell lysates for 4 hours. LacZ was employed as the negative control. Streptavidin magnetic beads pulled down the proteins bonded with biotin-labeled LINC00958 or LacZ following overnight incubation. The generated protein-RNA-bead compounds were collected, and then the proteins were eluted. Finally, western blot assays were used to test the retrieved proteins. The primary antibodies used were as follows: GAPDH (AF7021, Affinity Biosciences, Changzhou, China, 1:3000), IGF2BP3 (ab179807, Abcam, USA, 1:1000).

### RNA fluorescence in situ hybridization (FISH)

We conducted fluorescence in situ hybridization of RNA with a Ribo Fluorescent in Situ Hybridiazation Kit (RiboBio, Guangzhou, China) according to the manufacturer’s protocols. Specific FISH probes for LINC00958 were designed and synthesized by RiboBio. We captured all images with a confocal laser scanning microscope (Examiner. Z1, Carl Zeiss, Germany).

### Fluorescence immunocytochemical staining

EC cells on glass coverslips were incubated with specific antibodies against IGF2BP3 (ab179807, Abcam, 1:80) overnight at 4 °C and then incubated with Alexa Fluor 488 goat anti-rabbit IgG (Affinity Biosciences, Changzhou, China, 1:200). To detect colocalization, we treated the cells with FISH probes and the anti-IGF2BP3 primary antibody in hybridization buffer at the same time. Images were captured by a confocal laser scanning microscope (Examiner. Z1, Carl Zeiss, Germany).

### RNA stability assay for mRNA lifetime

We seeded EC cells into 12-well plates until 70–80% confluency after 24 h. Then, we added 5 µg/ml actinomycin D (GC16866, GlpBio Technology, USA) to the growth medium and collected them at 0 h, 2 h, 4 h, and 6 h. Total RNA was extracted, reverse-transcribed to cDNA and amplified by qPCR. The half-life of mRNA was calculated according to a previously published article [17].

### In vivo growth assays

The animal experiments were approved by the ethics committee of Shengjing Hospital affiliated with China Medical University (Ethical number: 2020PS190K) and performed according to the relevant regulatory standards. A total of 1 × 10^6^ HEC-1-A cells were subcutaneously injected into the right axilla of 4–6-week-old female BALB/c nude mice (n = 5 per group), which were purchased from HFK Bioscience (Beijing, China). Tumor growth rates were monitored every week. The calculation formula used for the tumor volume was V = π/6 × length×width^2^. Three weeks after injection, the mice were sacrificed. Then, the tumor samples were fixed in 4% paraformaldehyde, paraffin-embedded, and sectioned for immunohistochemical staining.

### Statistical analysis

GraphPad Prism 8.0 (La Jolla, USA) and SPSS 23.0 (Abbott Laboratories, USA) were applied for all statistical analyses. The results are presented as the mean ± standard deviation. We adopted the chi-squared test to evaluate relationships between IGF2BP3 expression and the clinicopathologic features of patients. The Kaplan–Meier method with log-rank tests was employed to analyse overall survival. The linear relationships between two variables were analysed by Pearson’s correlation coefficient. Independent sample Student’s *t* tests were used to compare statistical significance between two groups. When more than two groups were compared, we used one-way analysis of variance. *P* < 0.05 were defined as statistically significant.

## Supplementary information

Original pictures

Original western blot pictures

Supplementary figure and table legends

Supplementary Methods

Supplementary Figure 1

Supplementary Figure 2

Supplementary Figure 3

Supplementary Figure 4

Supplementary Figure 5

Supplementary Table 1

Supplementary Table 2

## Data Availability

Upon reasonable request, the corresponding author may provide the readers with all datasets used or analyzed in the course of the study.
