# Supplementary material for: IGF2BP3 enhances the mRNA stability of E2F3 by interacting with LINC00958 to promote endometrial carcinoma progression
Source: Cell Death Discov. 2022 Jun 8;8:279. doi: 10.1038/s41420-022-01045-x (PMC9177600; doi:10.1038/s41420-022-01045-x)
Supplement: Supplementary file 1 — Original pictures [file 41420_2022_1045_MOESM1_ESM.pdf]

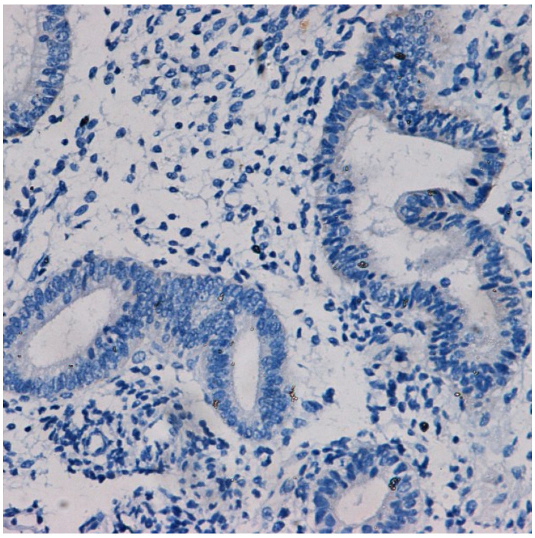

Fig. 1D-Normal

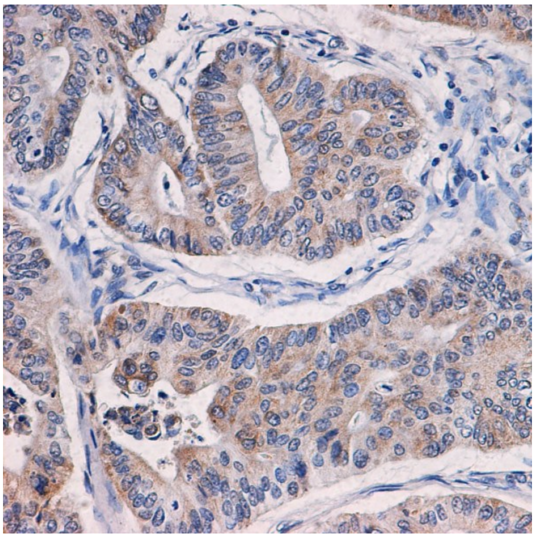

Fig. 1D-Tumor

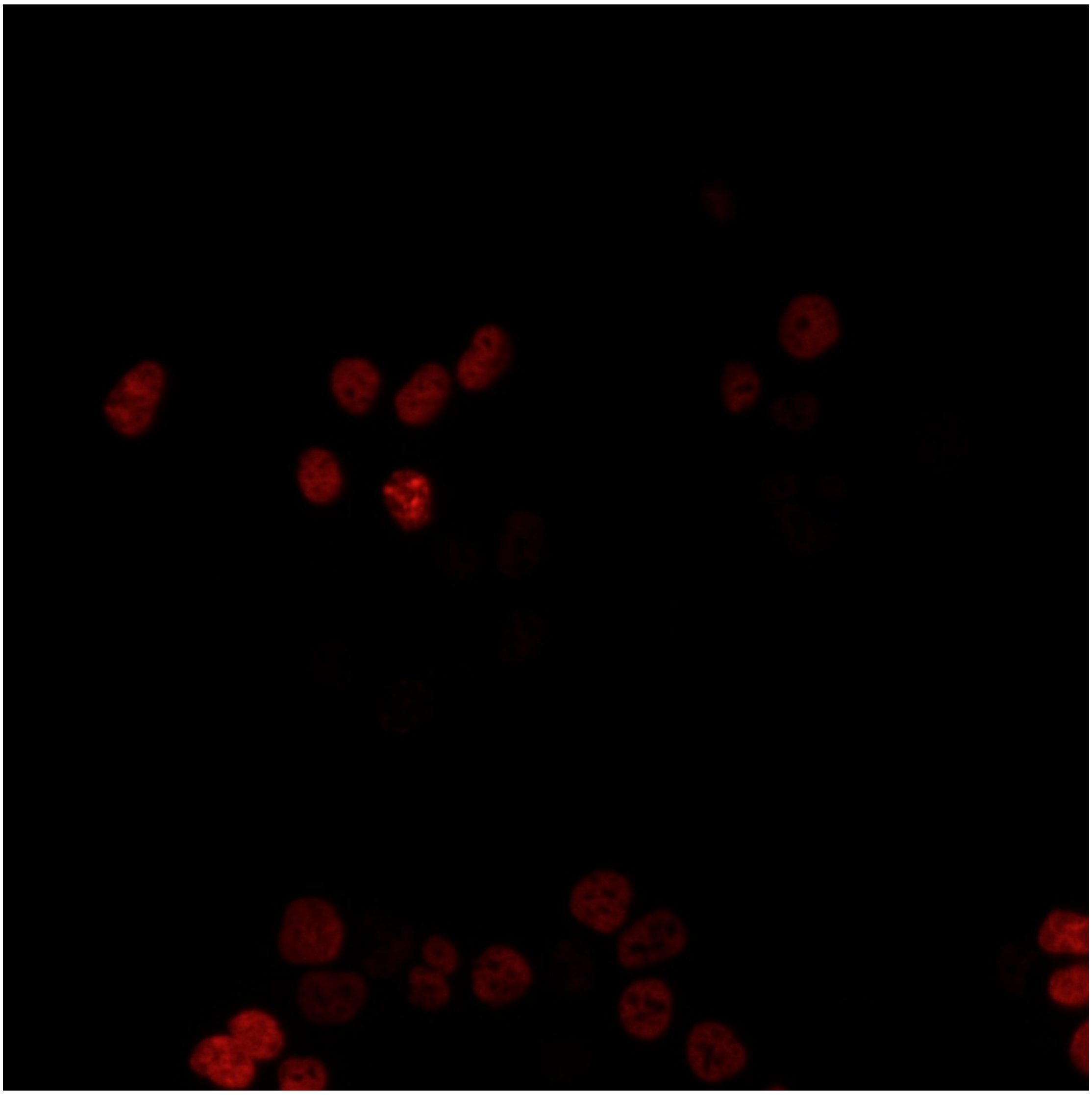

Fig.2C-HEC-1-A-sh-IGF2BP3-1-EdU

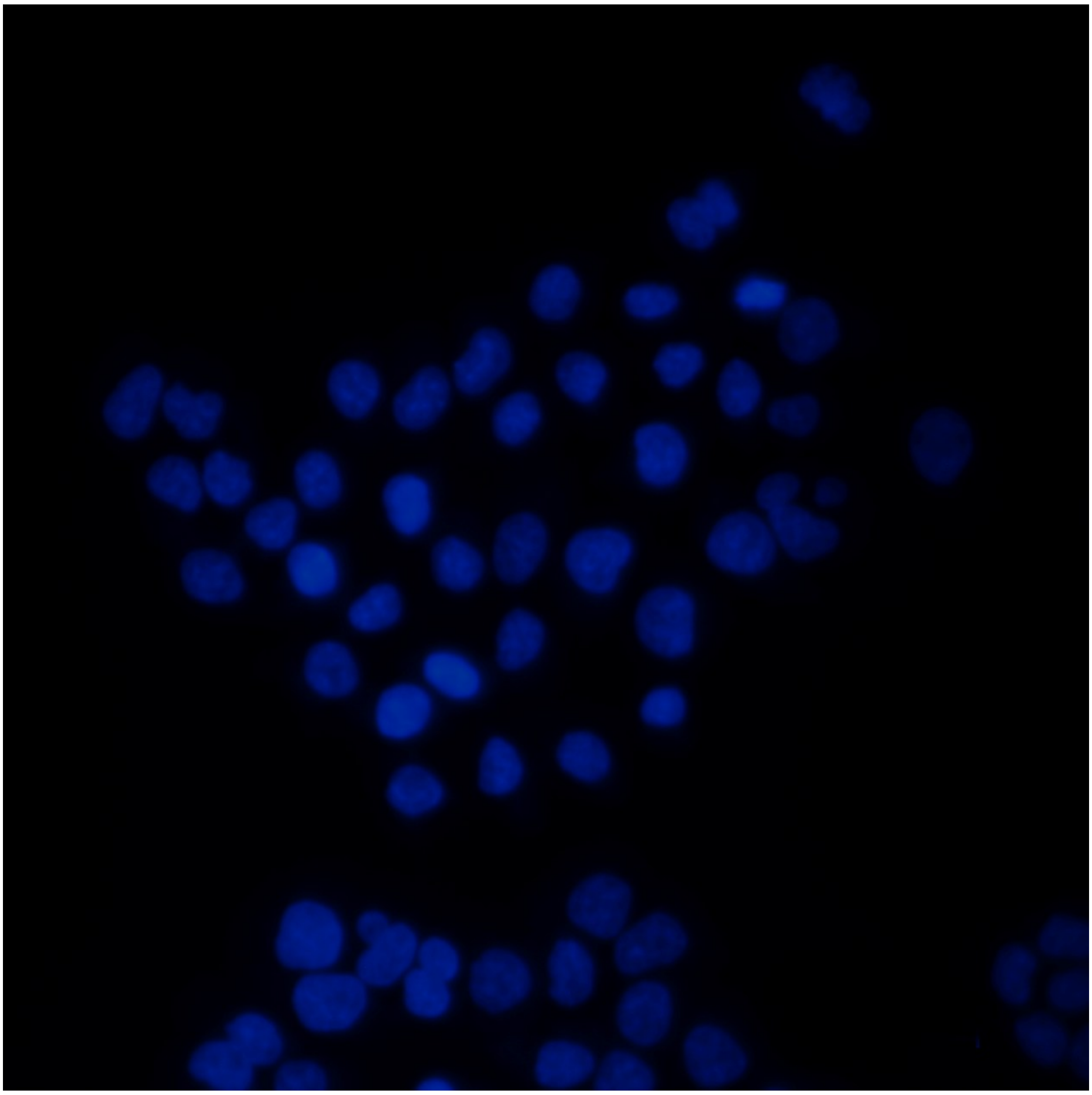

Fig.2C-HEC-1-A-sh-IGF2BP3-1-Hoechest

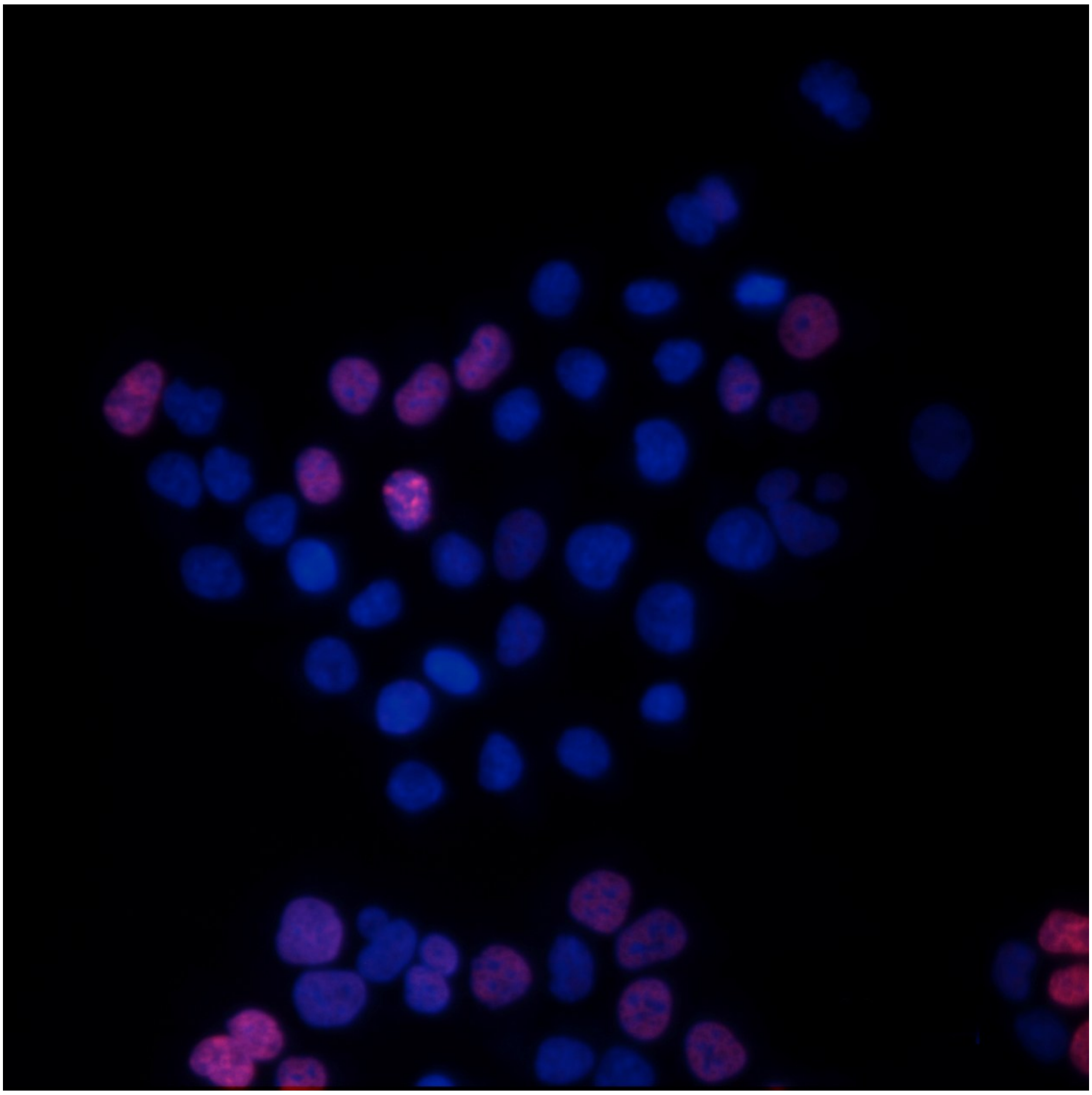

Fig.2C-HEC-1-A-sh-IGF2BP3-1-merge

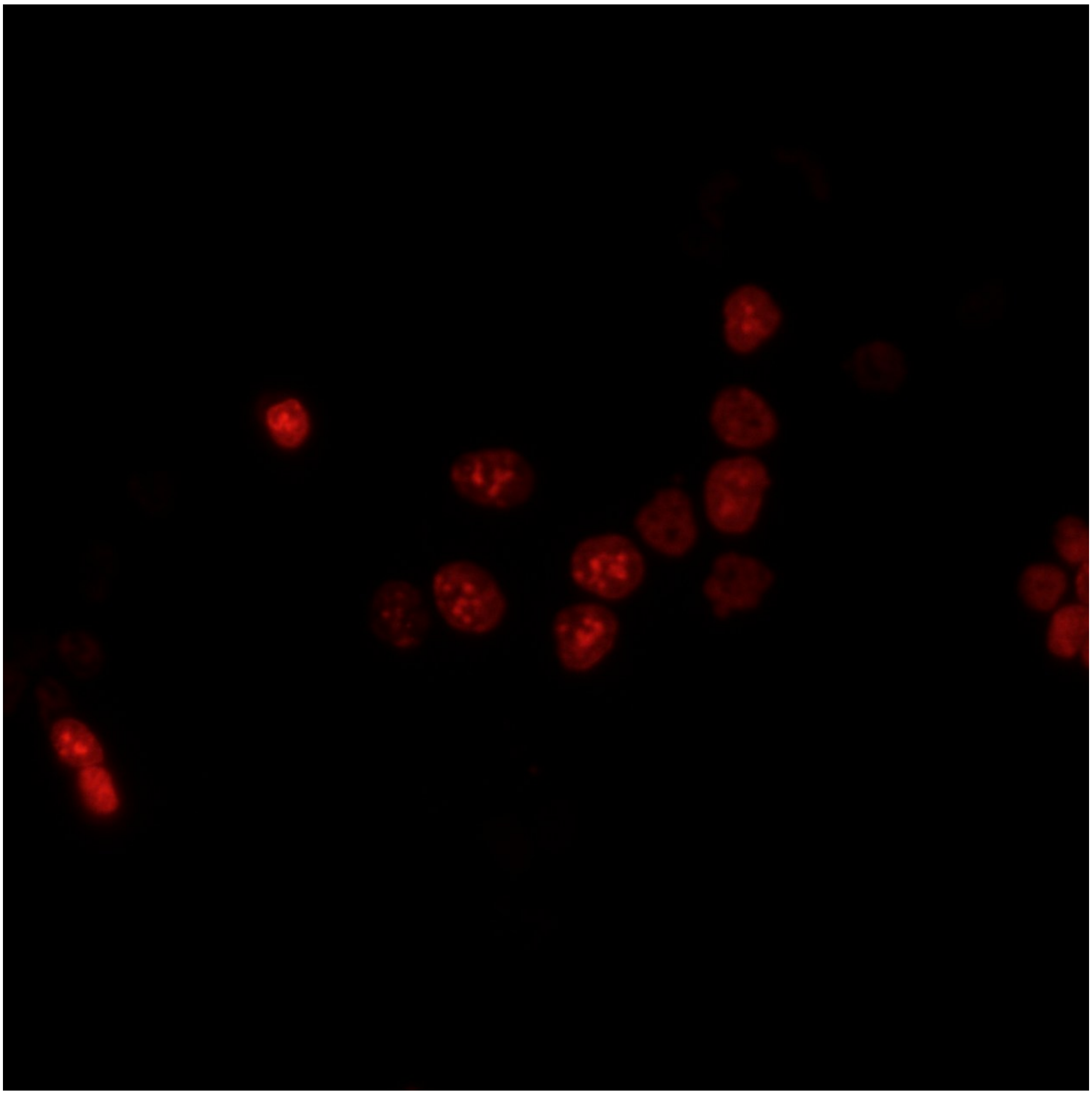

Fig.2C-HEC-1-A-sh-IGF2BP3-3-EdU

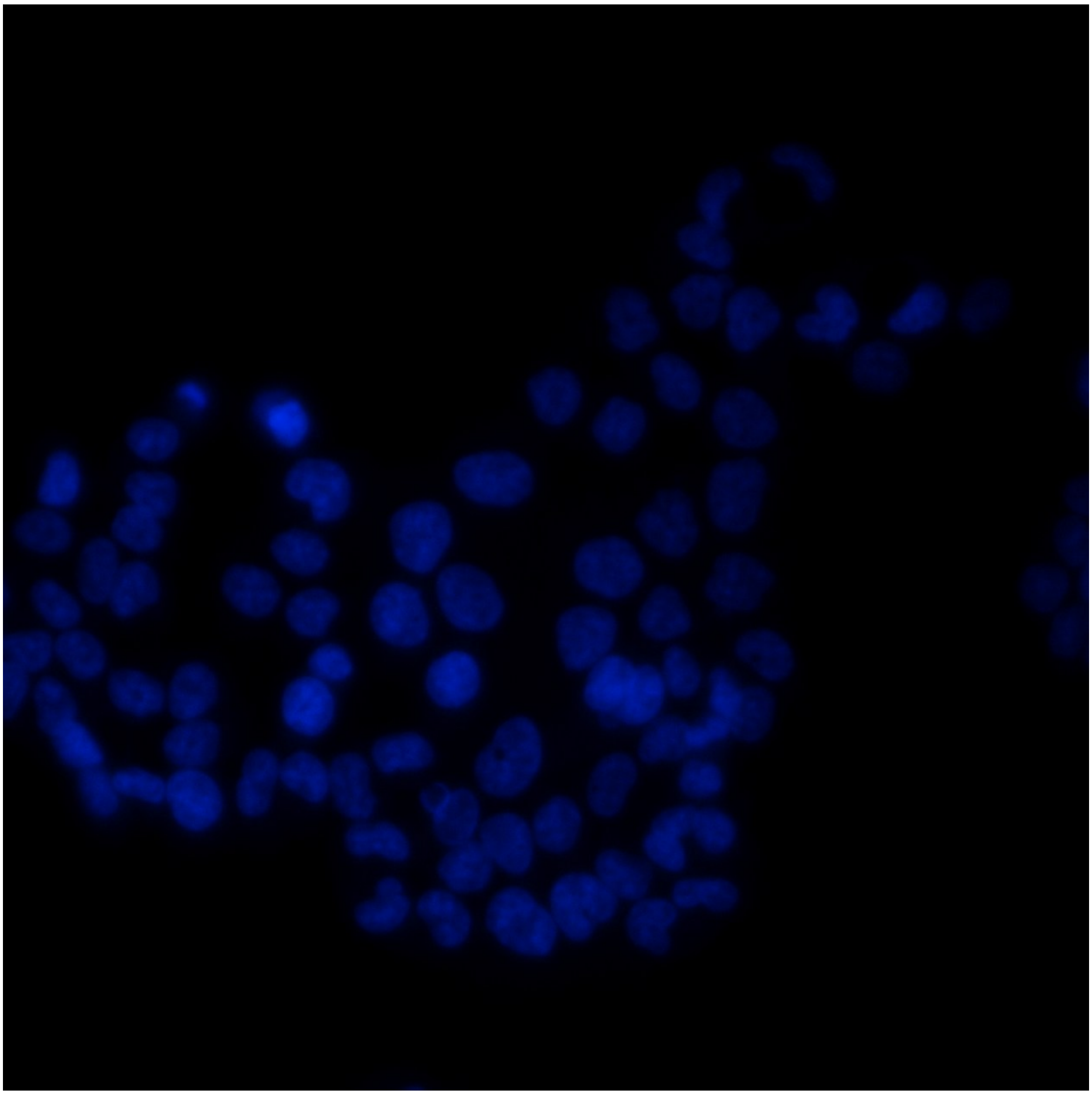

Fig.2C-HEC-1-A-sh-IGF2BP3-3-Hoechest

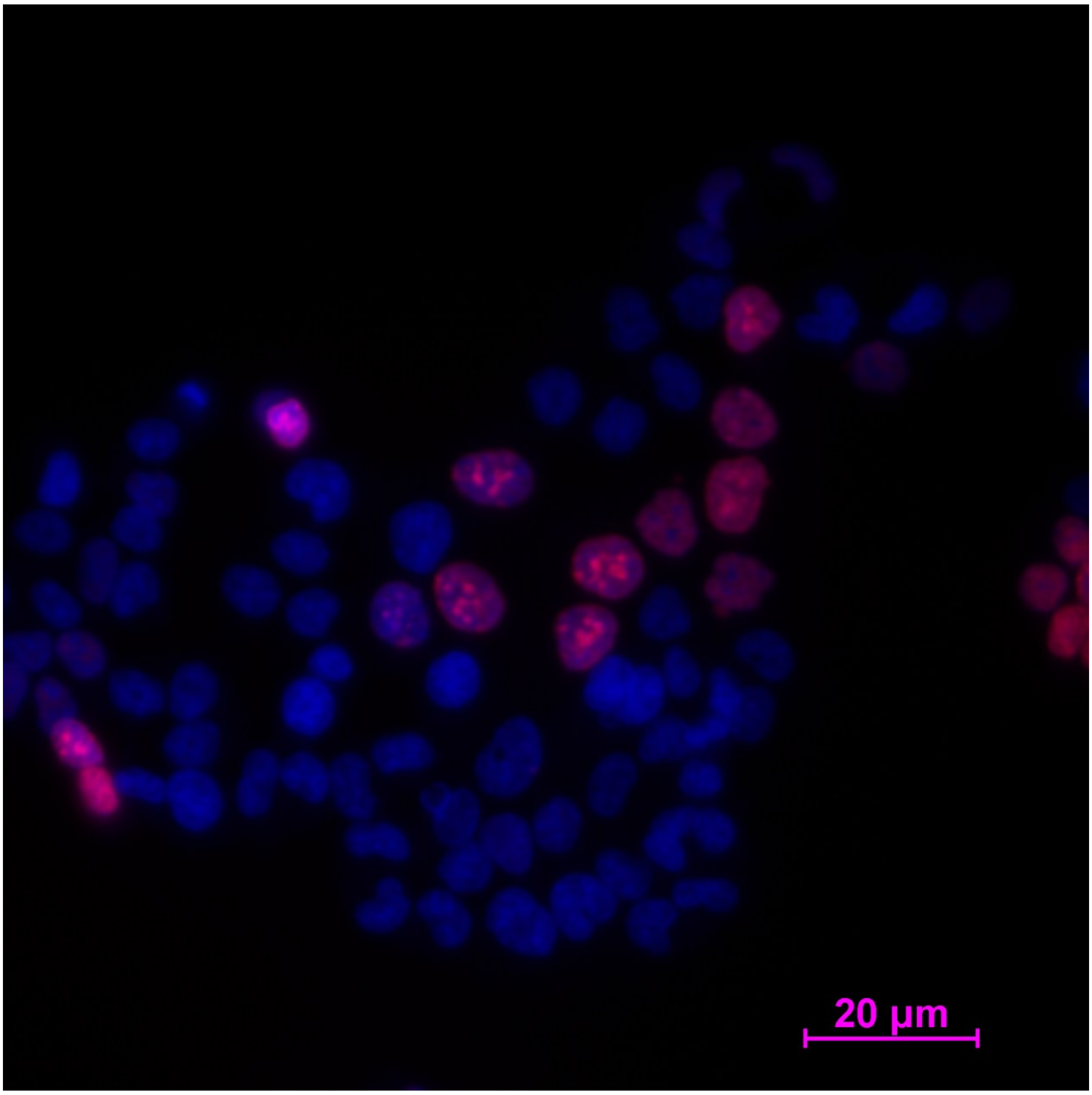

Fig.2C-HEC-1-A-sh-IGF2BP3-3-Merged

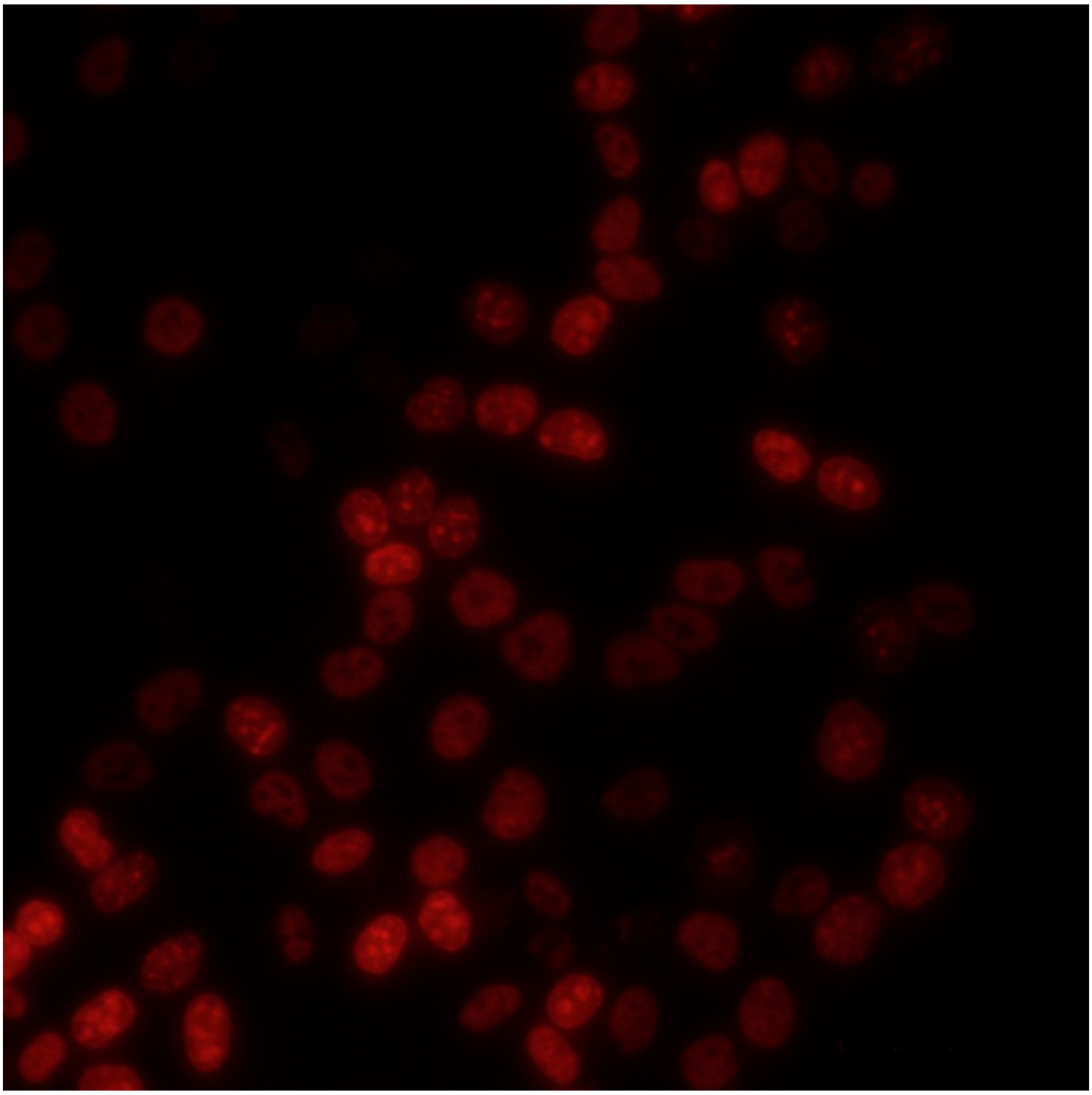

Fig.2C-HEC-1-A-sh-NC-EdU

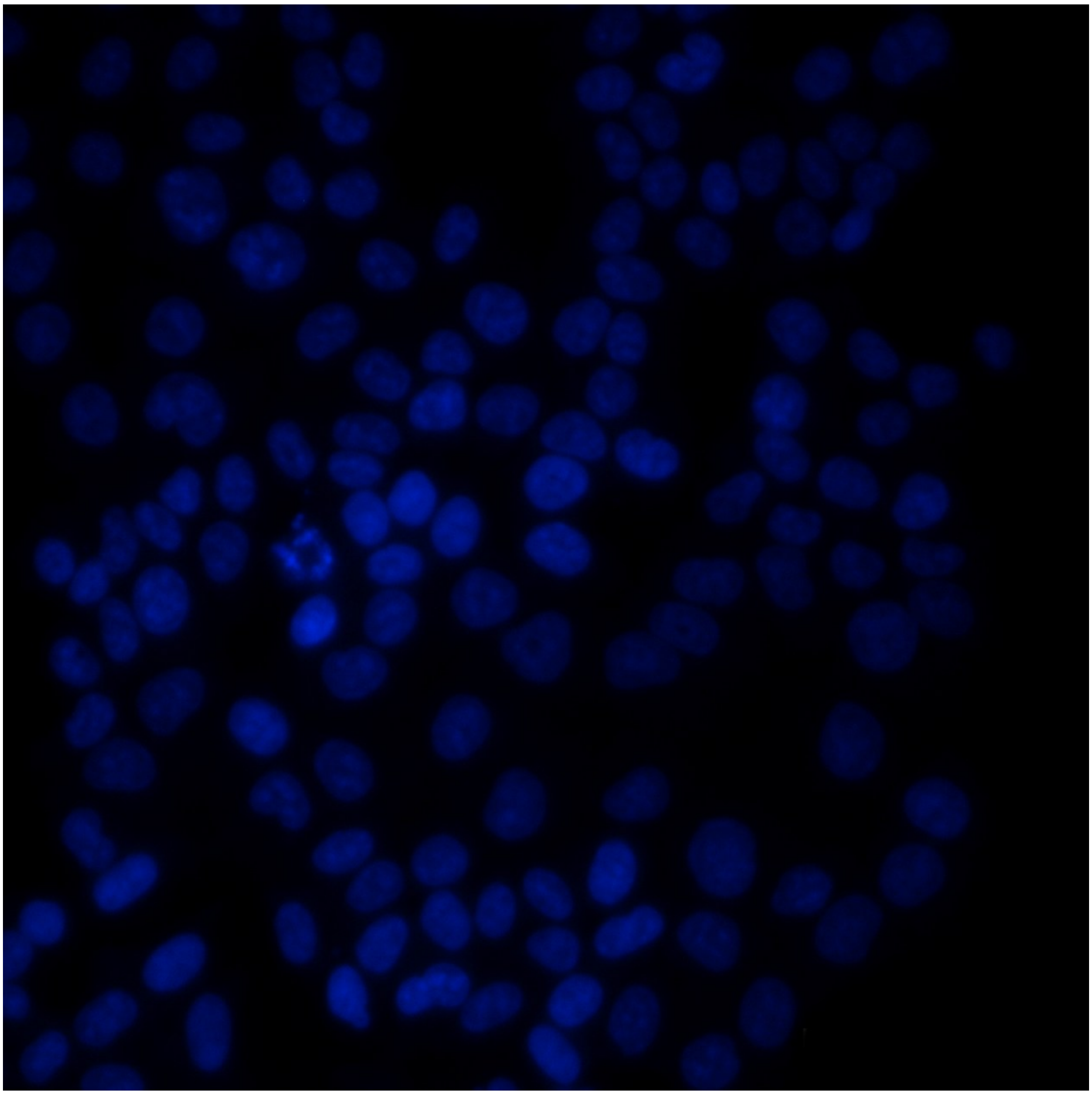

Fig.2C-HEC-1-A-sh-NC-Hoechest

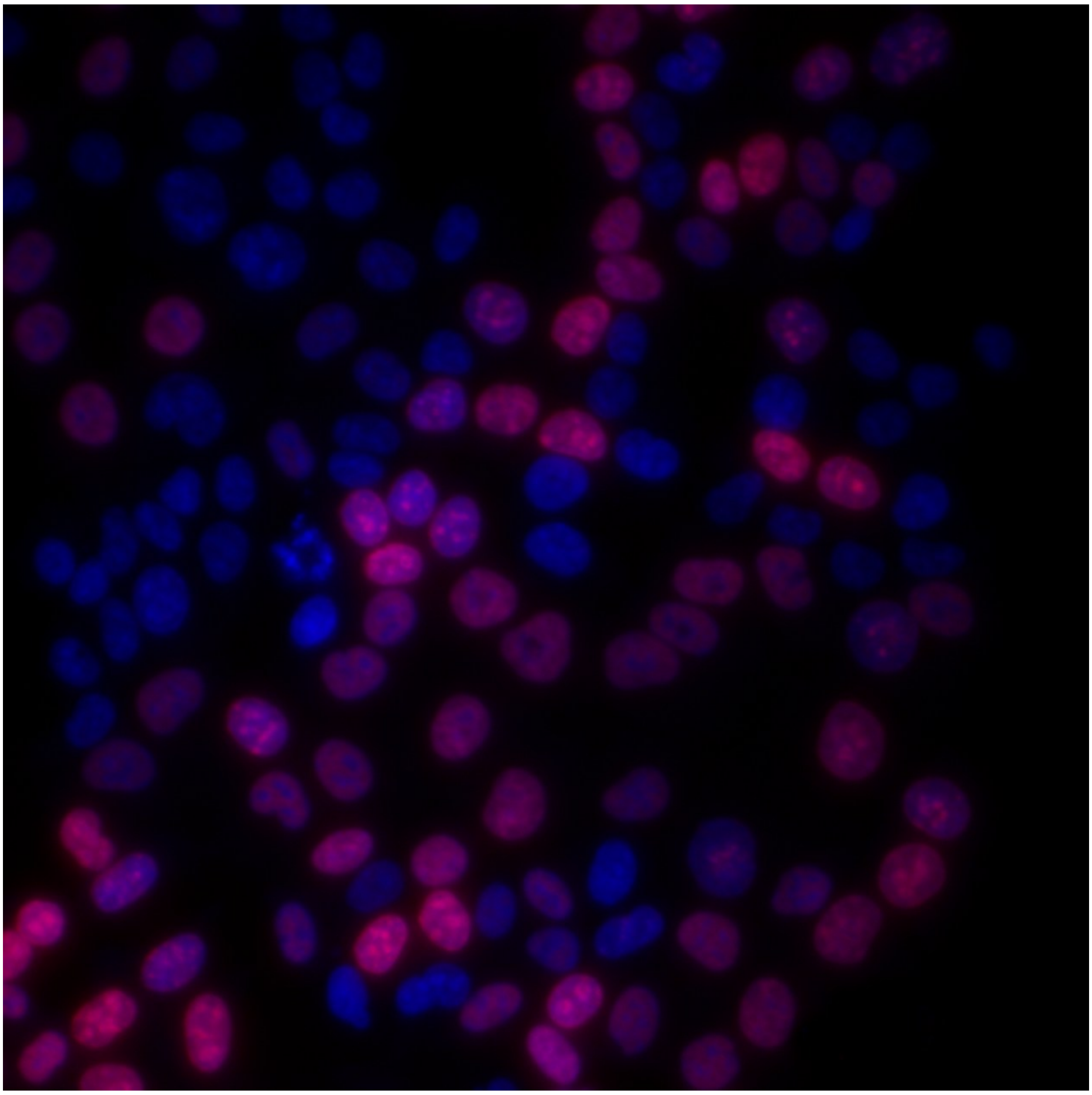

Fig.2C-HEC-1-A-sh-NC-Merged

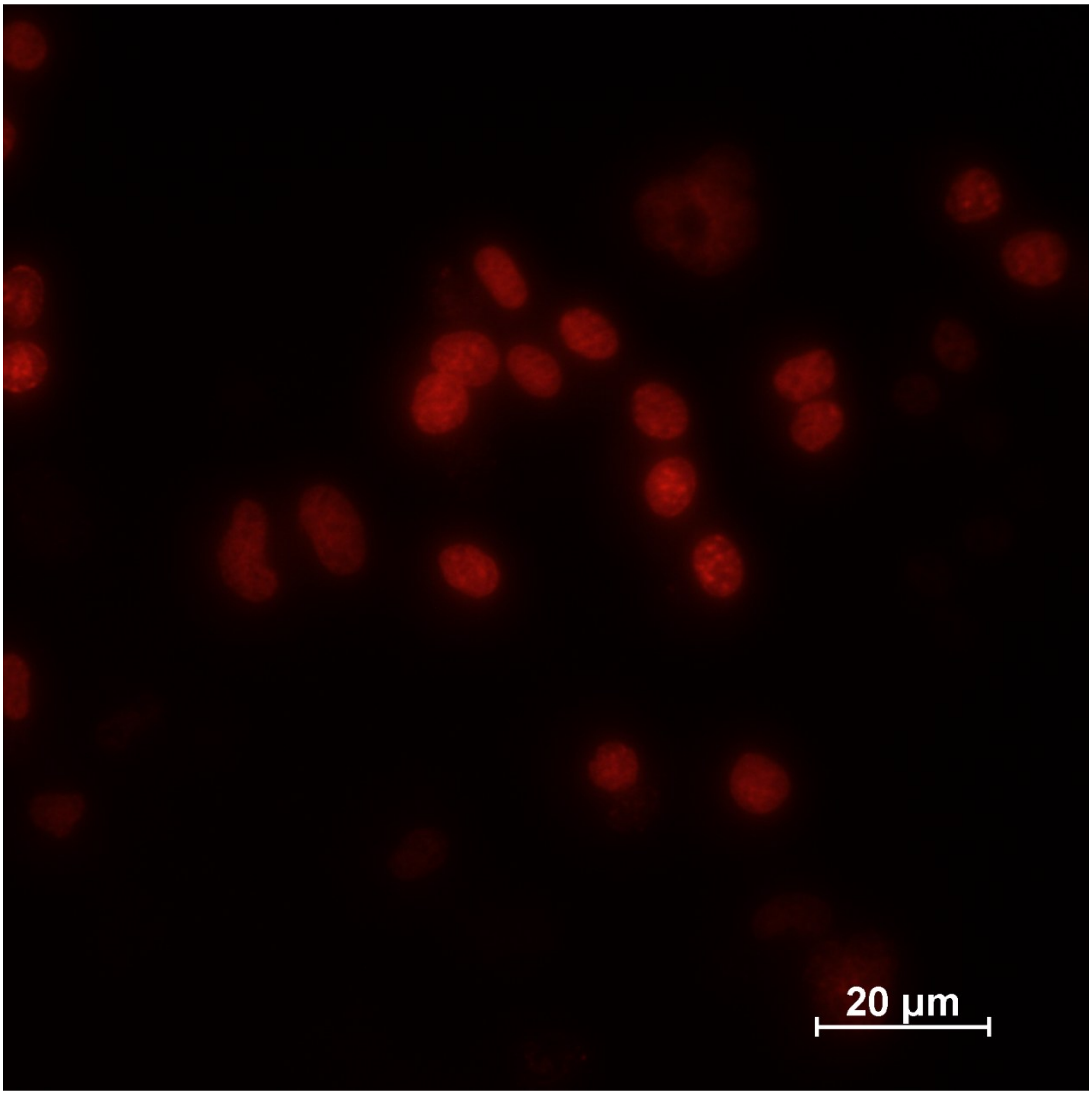

Fig.2C-Ishikawa-sh-IGF2BP3-1-EdU

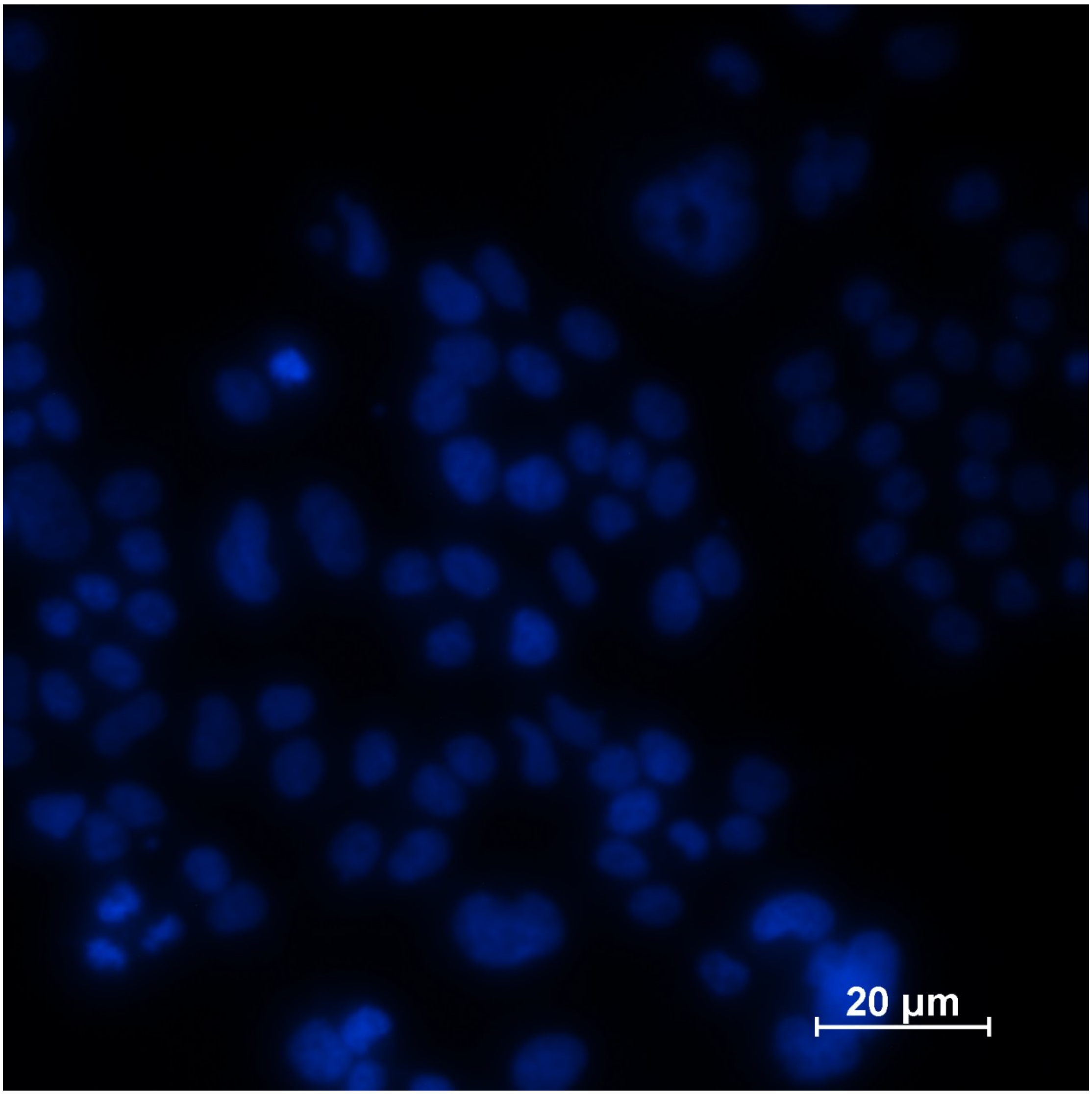

Fig.2C-Ishikawa-sh-IGF2BP3-1-Hoechest

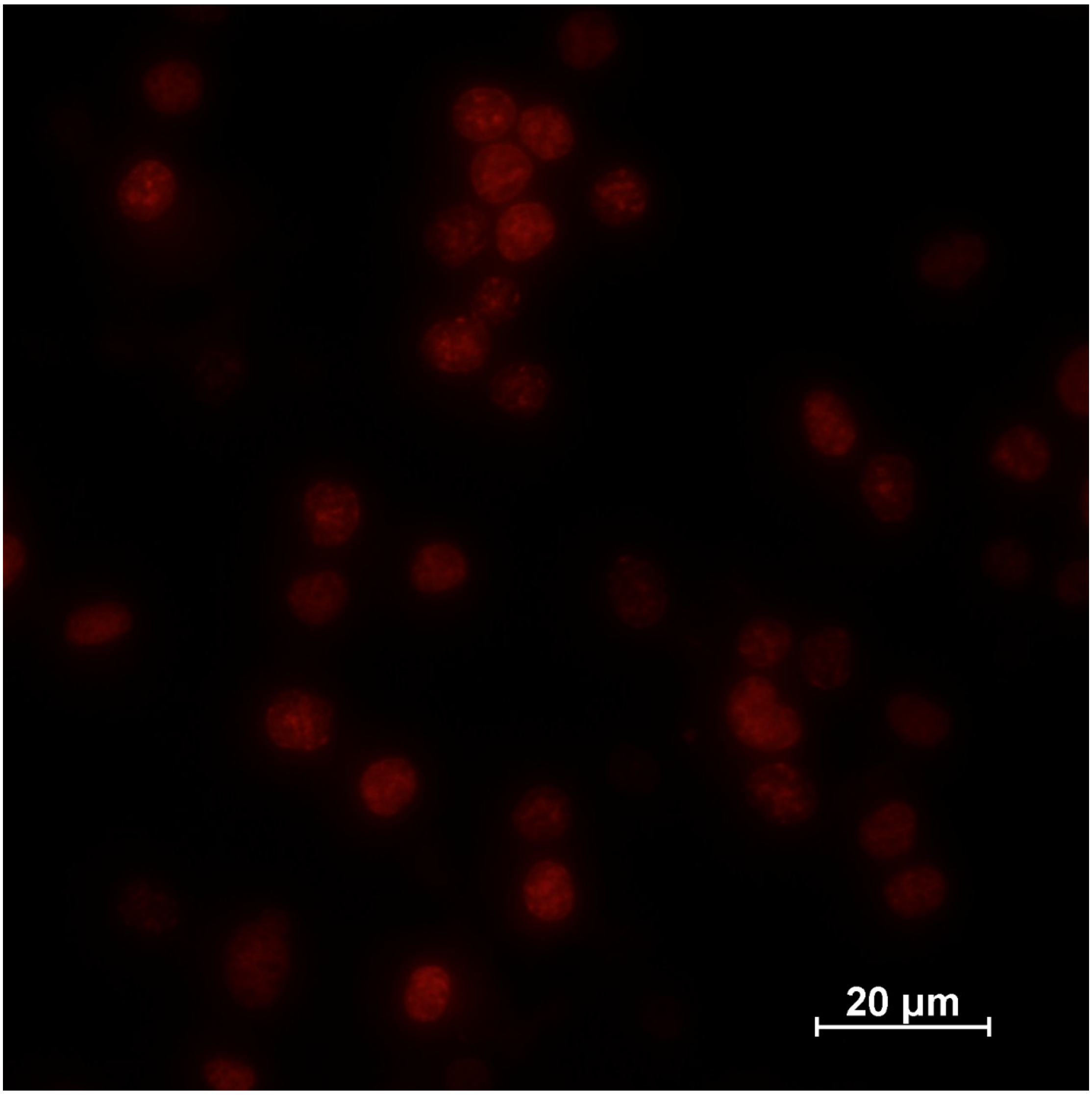

Fig.2C-Ishikawa-sh-IGF2BP3-3-EdU

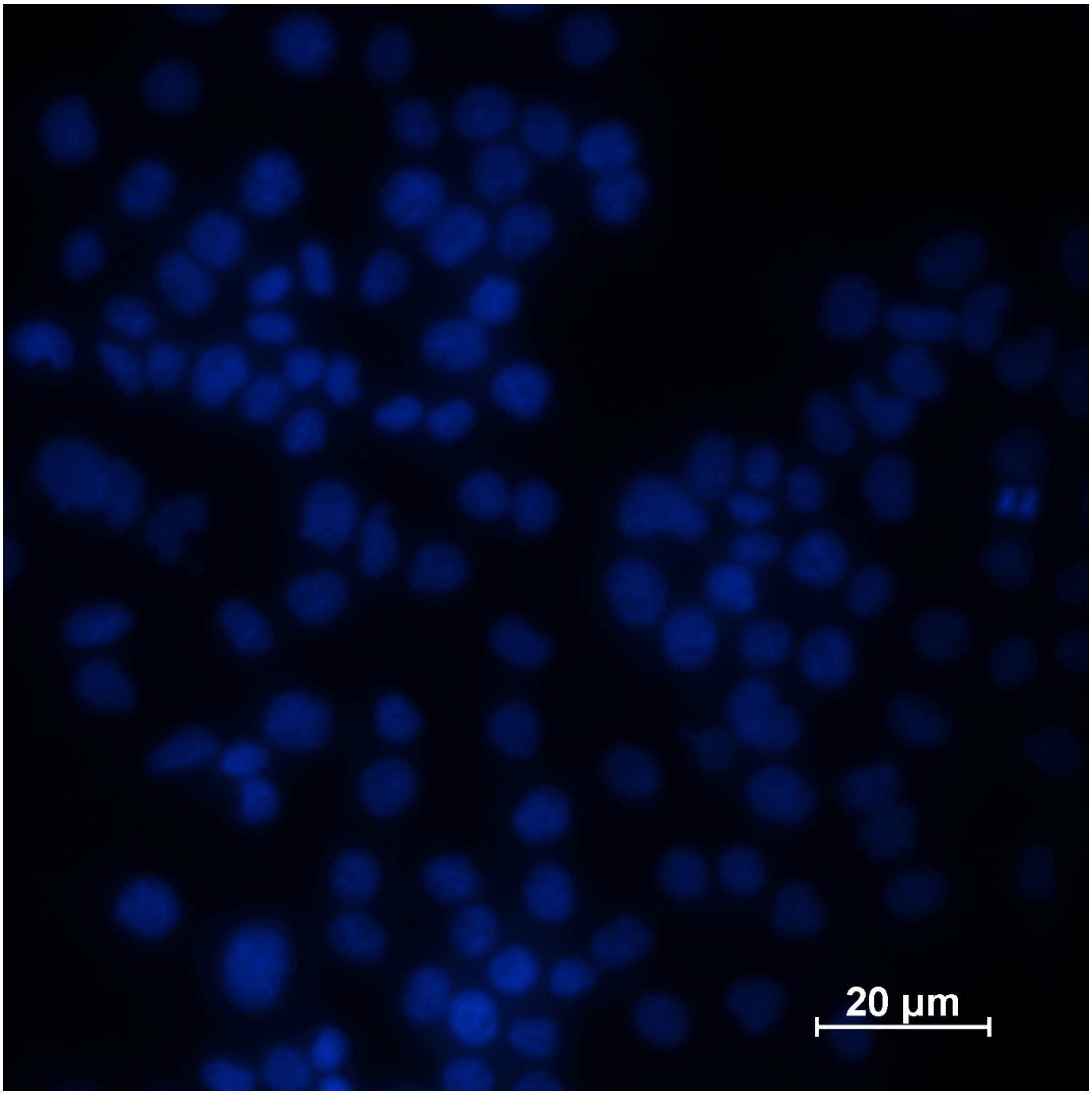

Fig.2C-Ishikawa-sh-IGF2BP3-3-Hoechest

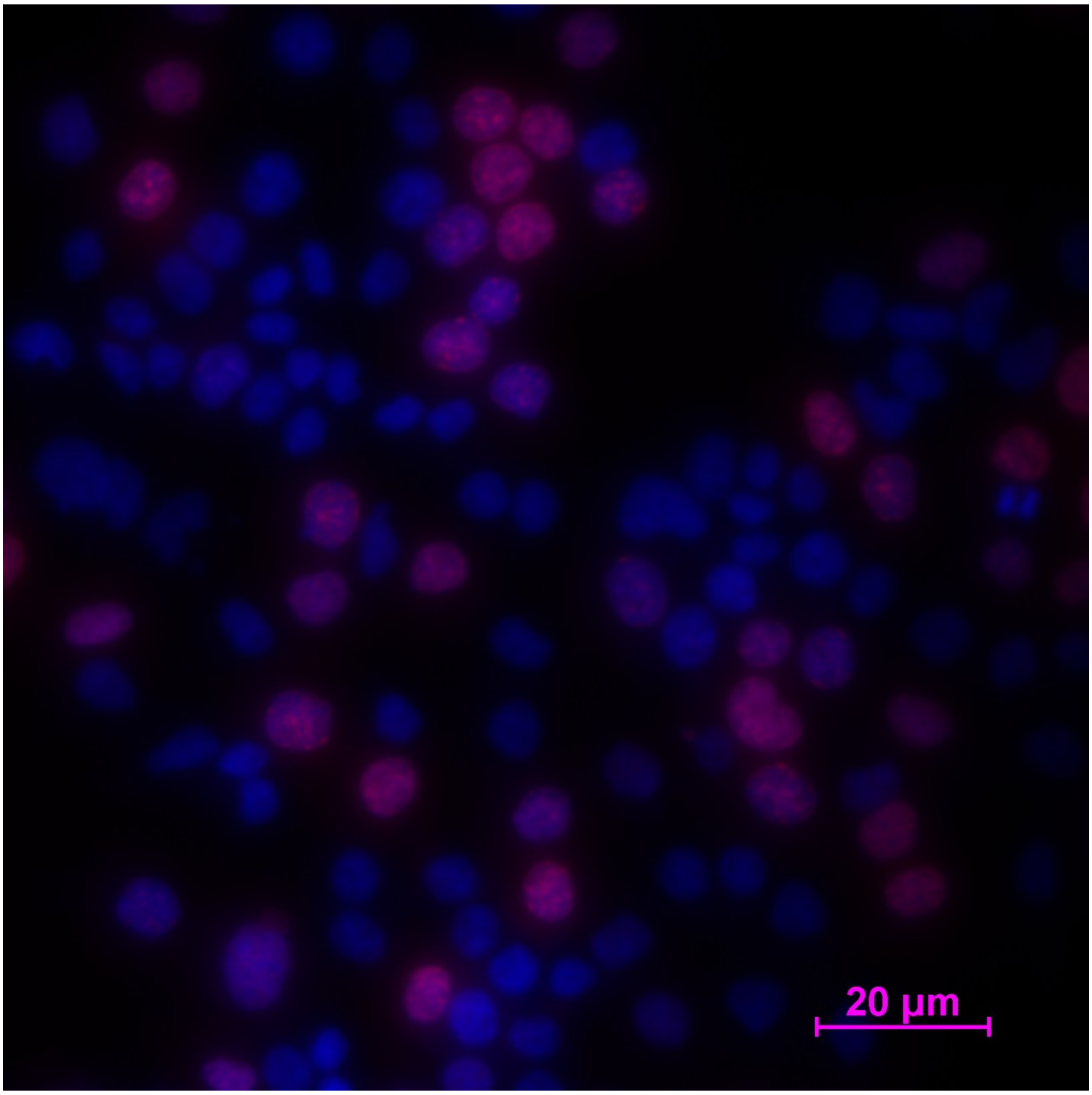

Fig.2C-Ishikawa-sh-IGF2BP3-3-merged

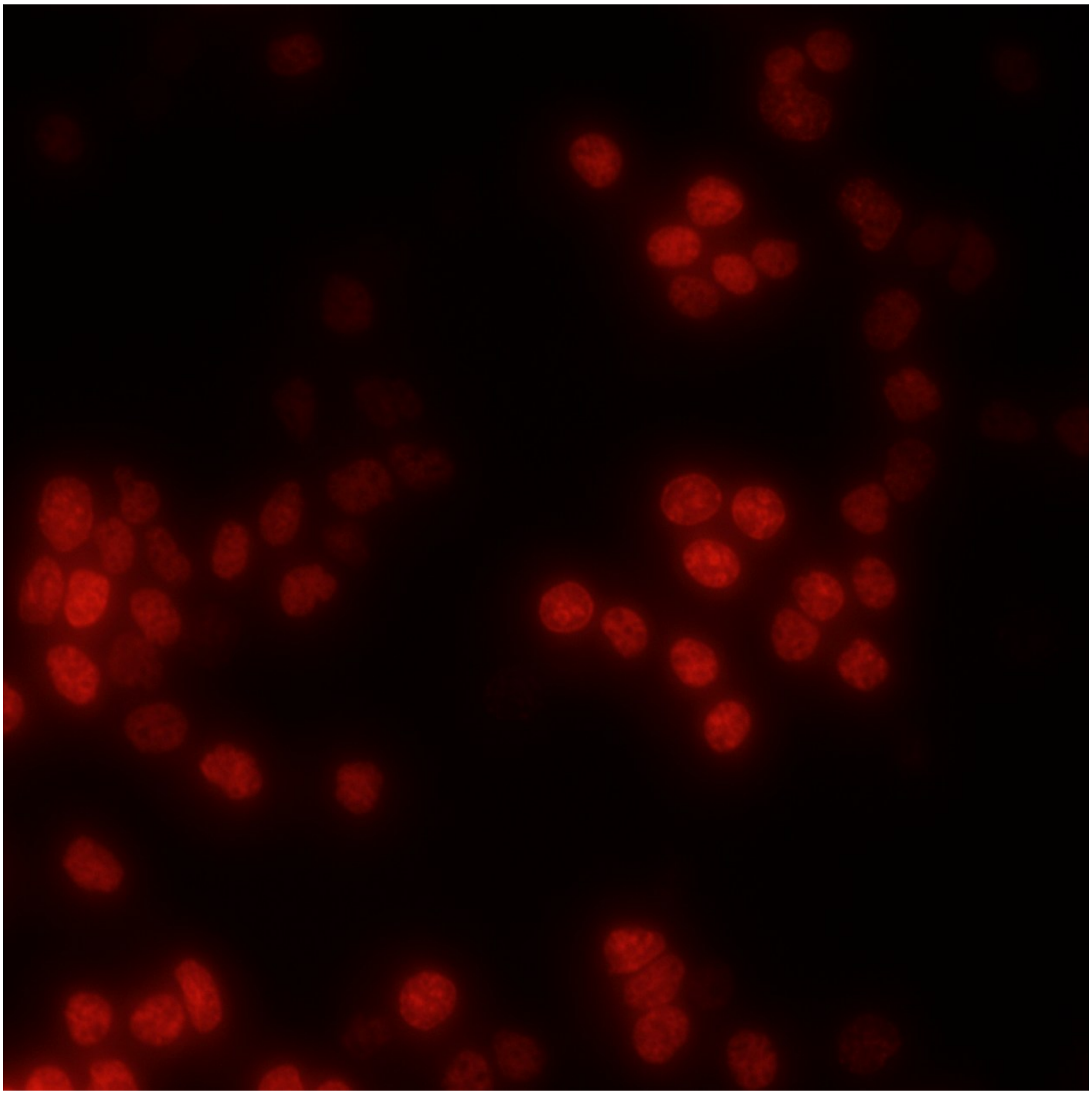

Fig.2C-Ishikawa-sh-NC-EdU

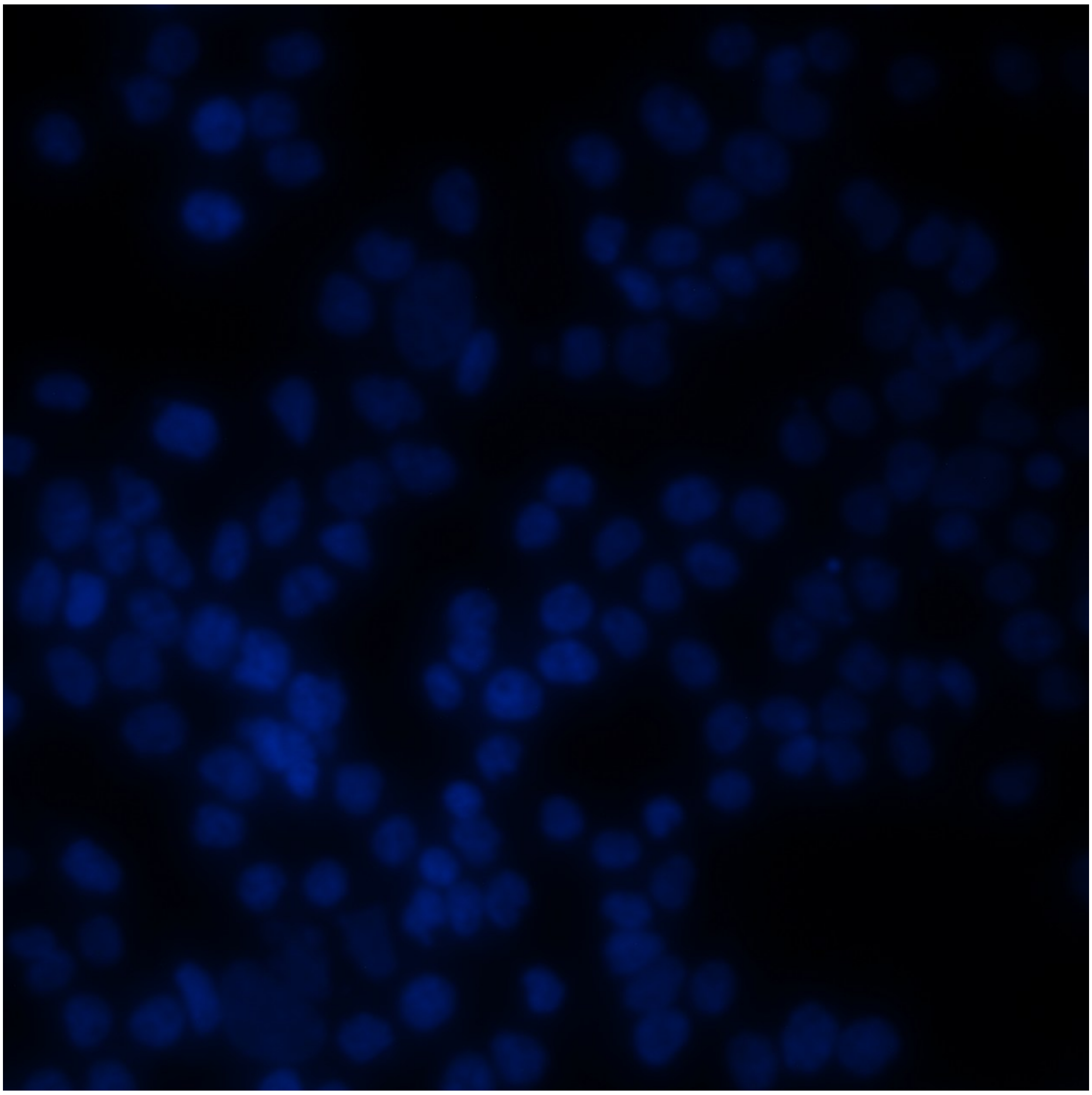

Fig.2C-Ishikawa-sh-NC-Hoechest

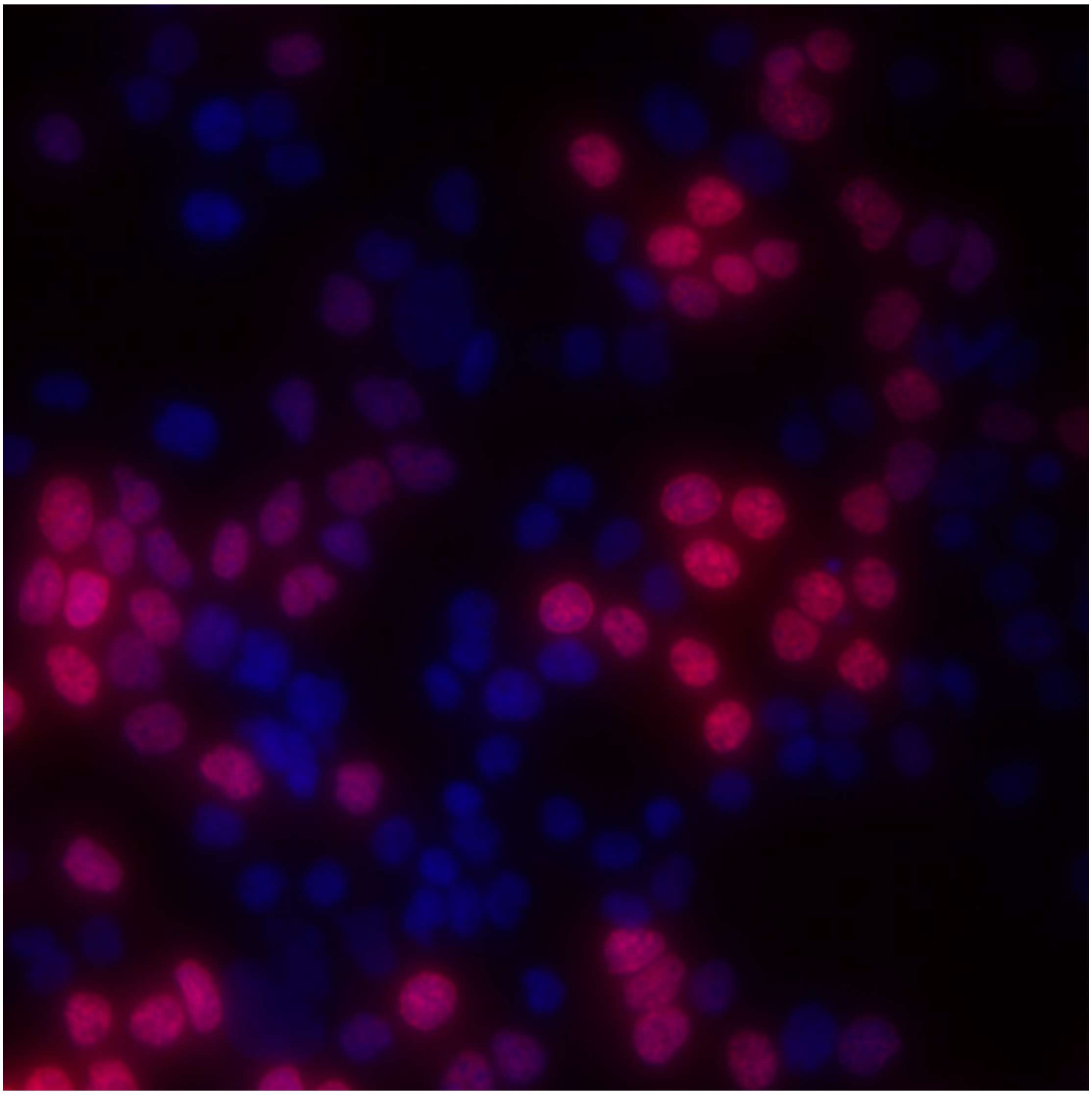
Fig.2C-Ishikawa-sh-NC-merged

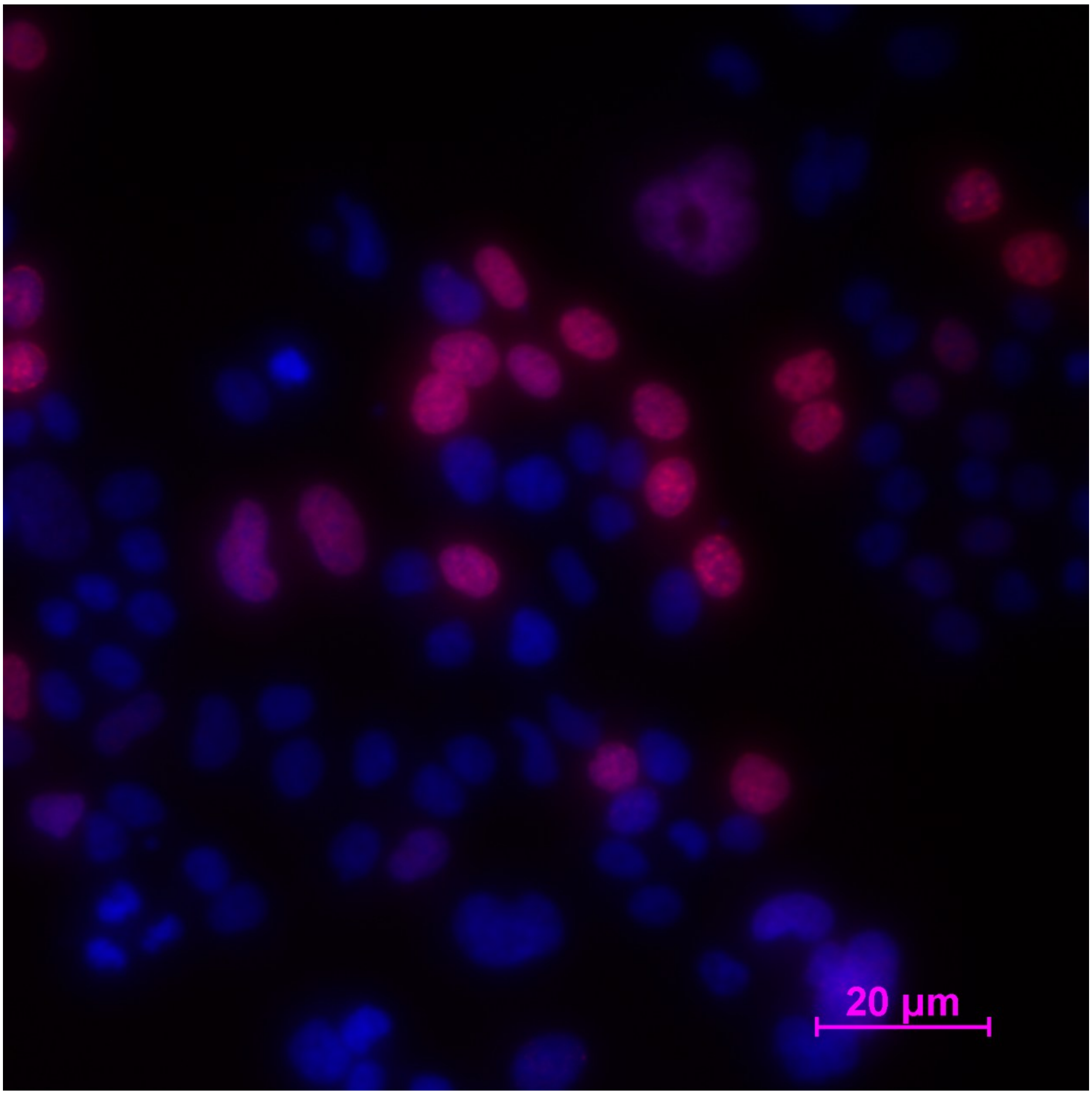

Fig.2C-Ishkawa-sh-IGF2BP3-1-merged

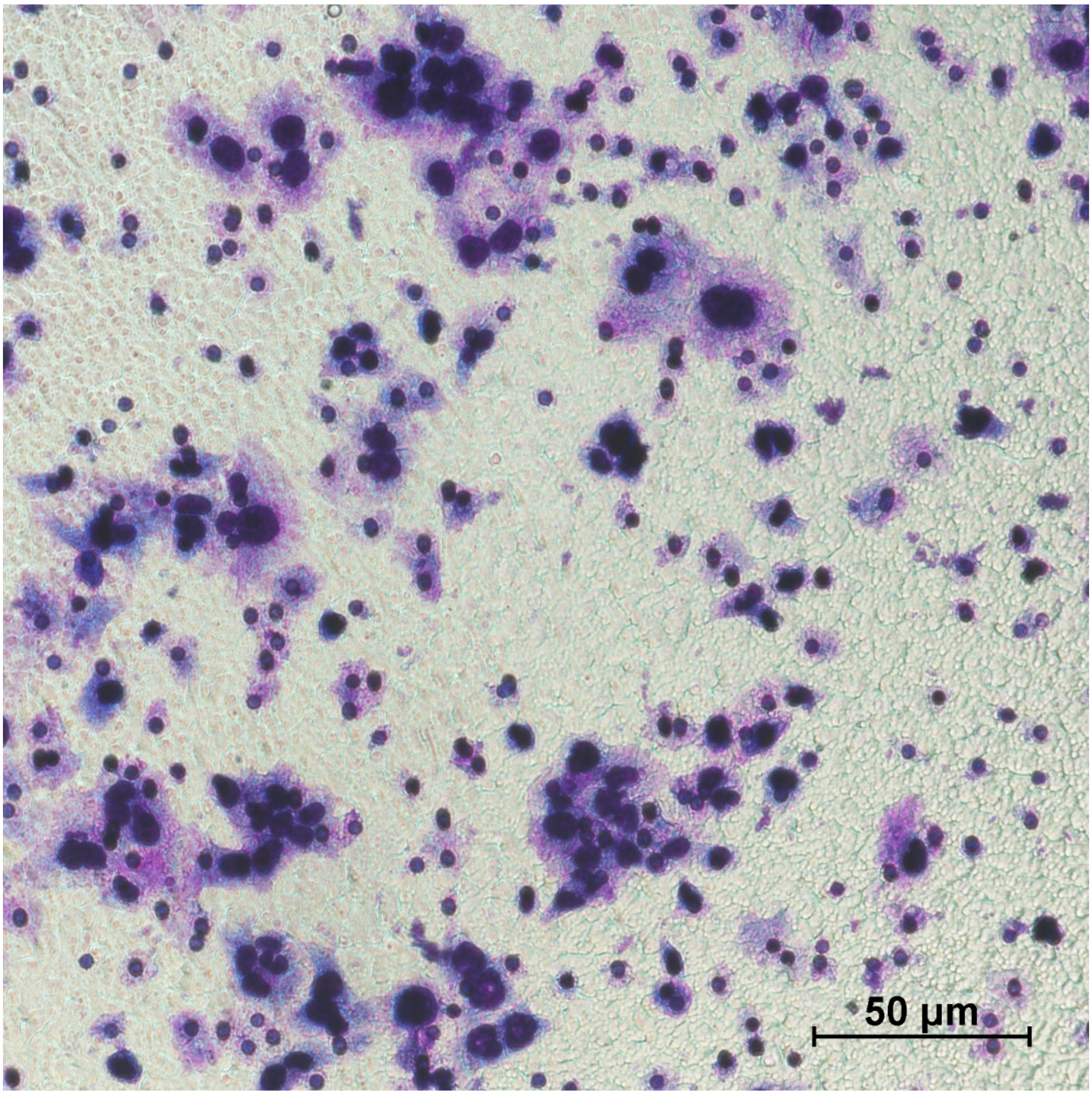

Fig.2D-HEC-1-A-invasion-sh-IGF2BP3-1

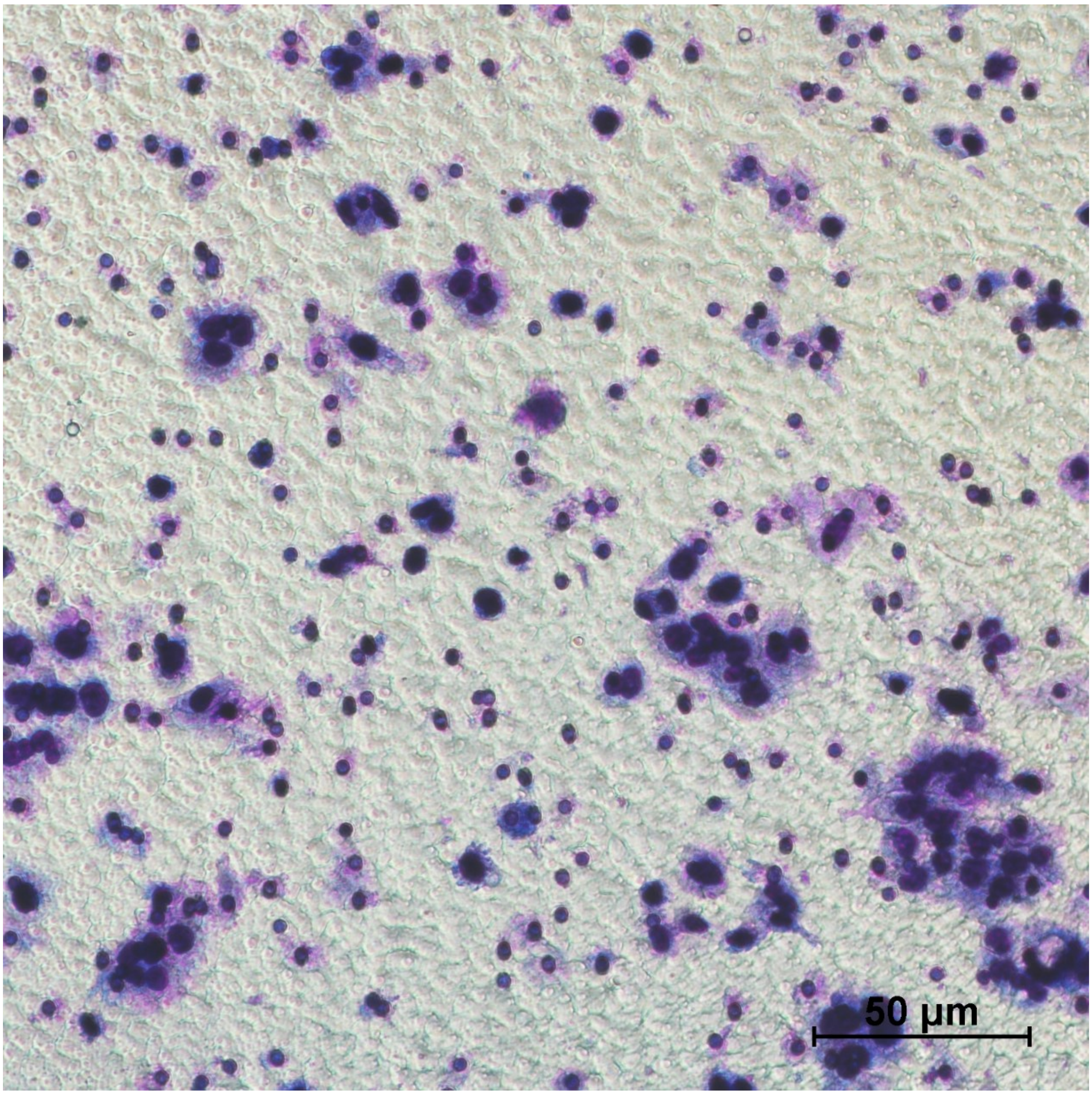

Fig.2D-HEC-1-A-invasion-sh-IGF2BP3-3

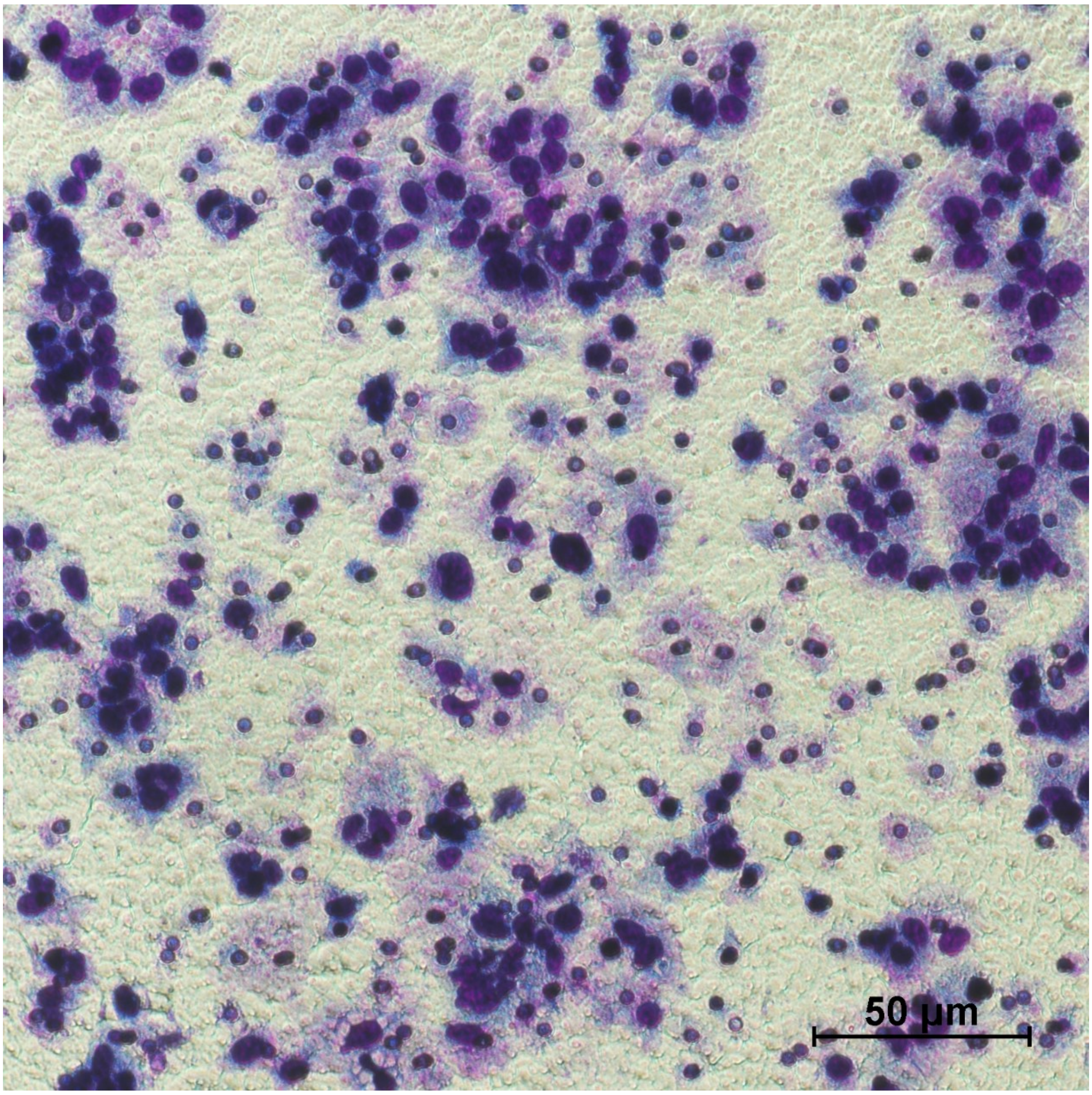

Fig.2D-HEC-1-A-invasion-sh-NC

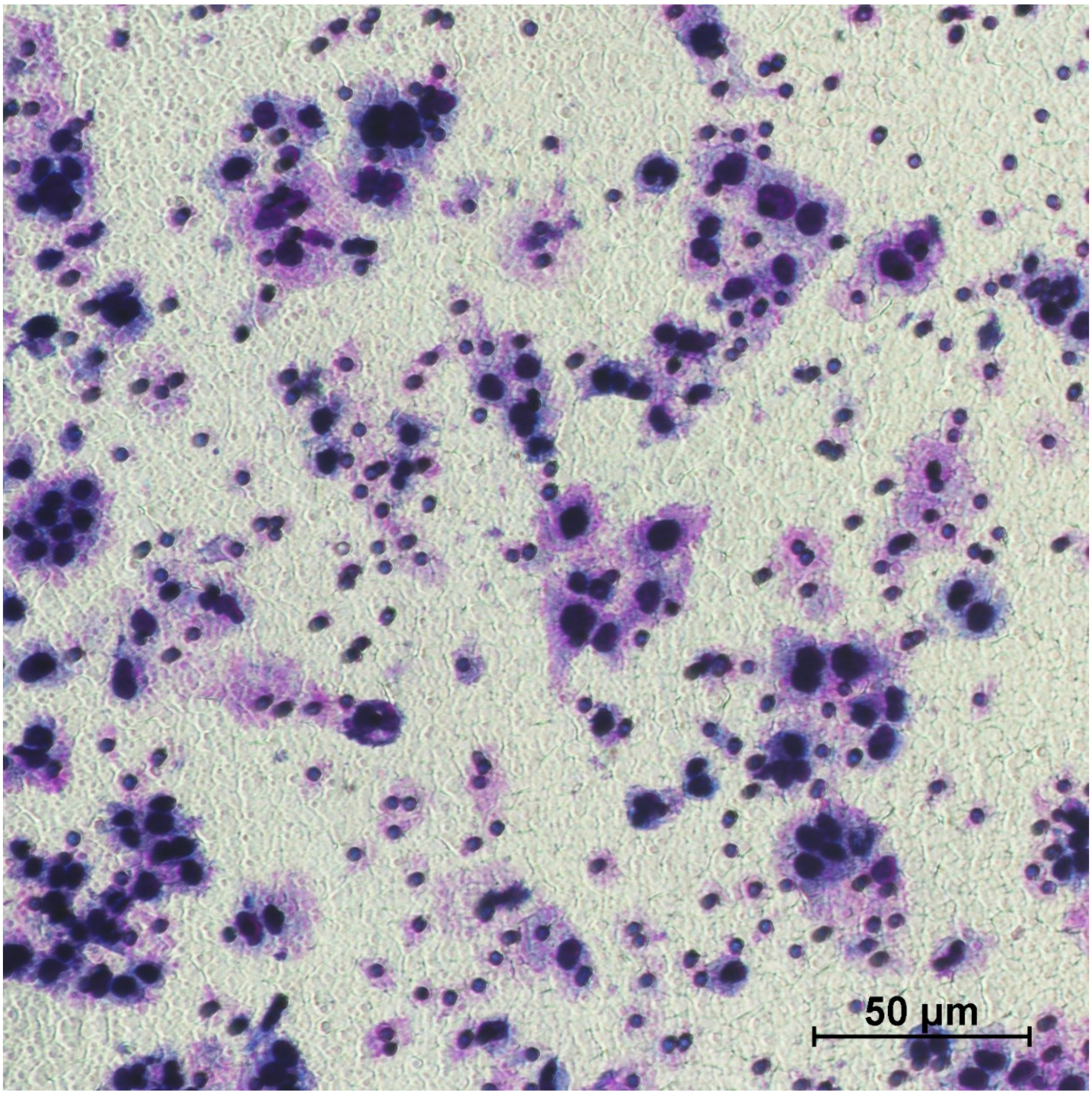

Fig.2D-HEC-1-A-migration-sh-IGF2BP3-1

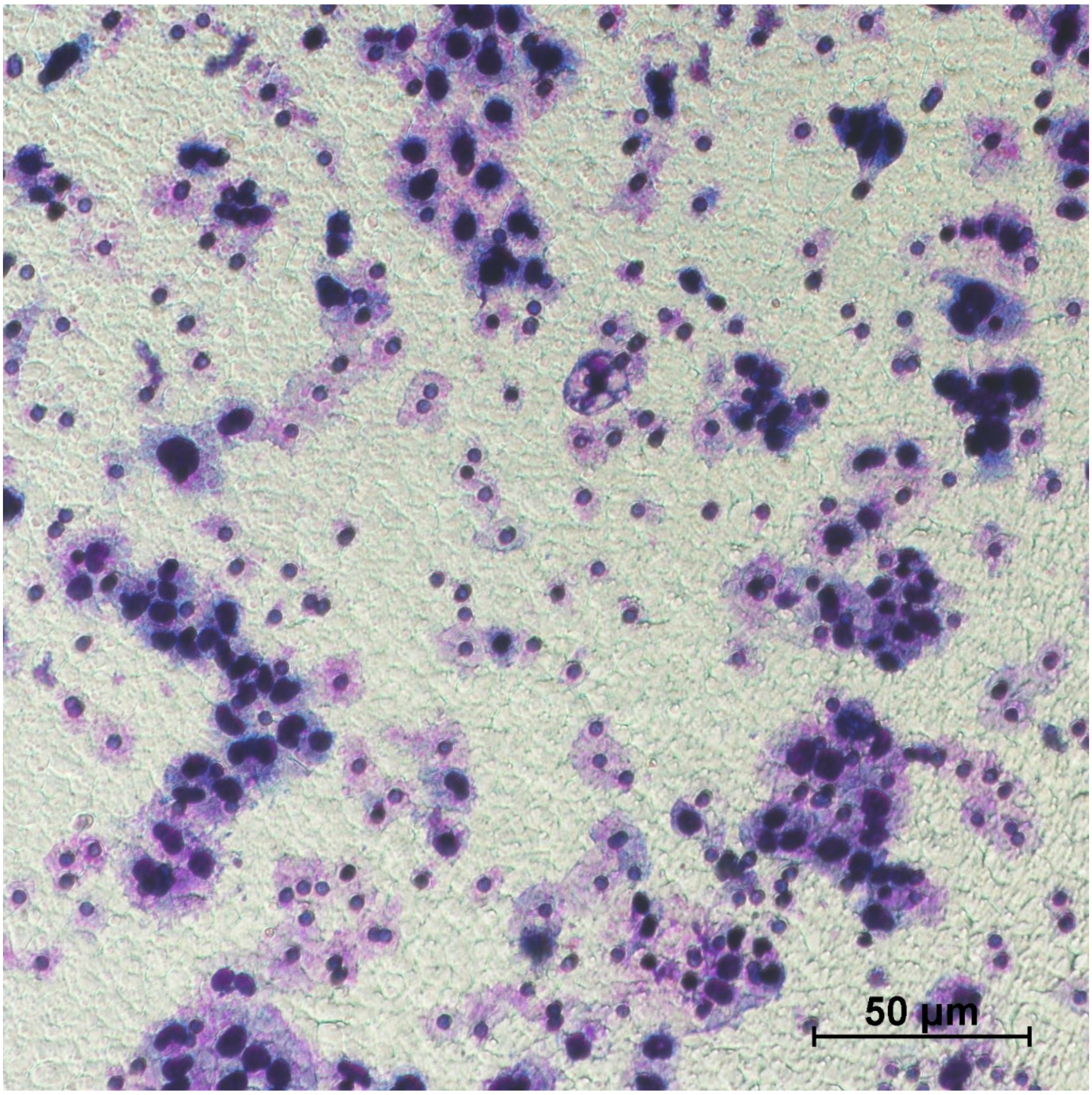

Fig.2D-HEC-1-A-migration-sh-IGF2BP3-3

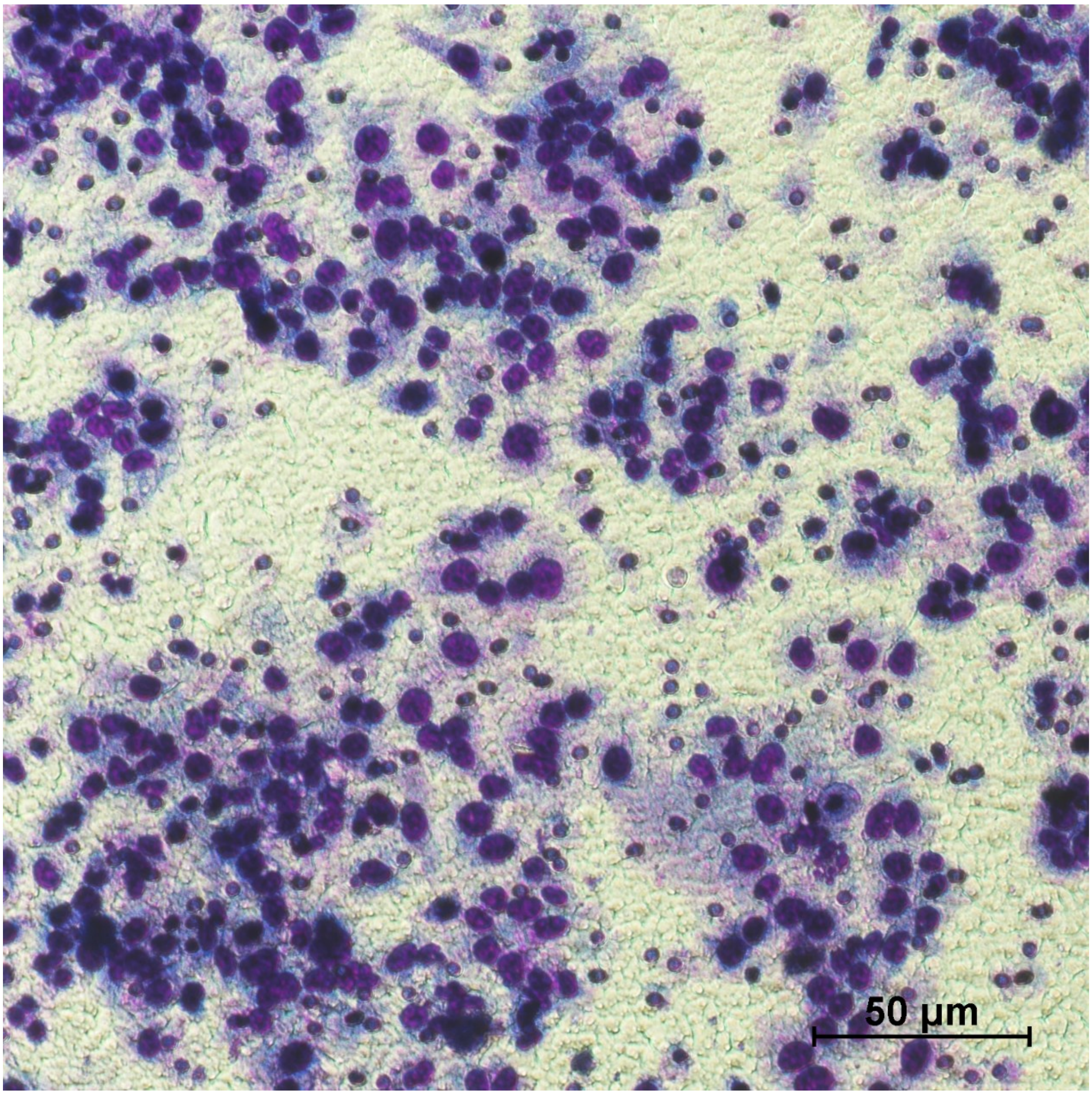

Fig.2D-HEC-1-A-migration-sh-NC

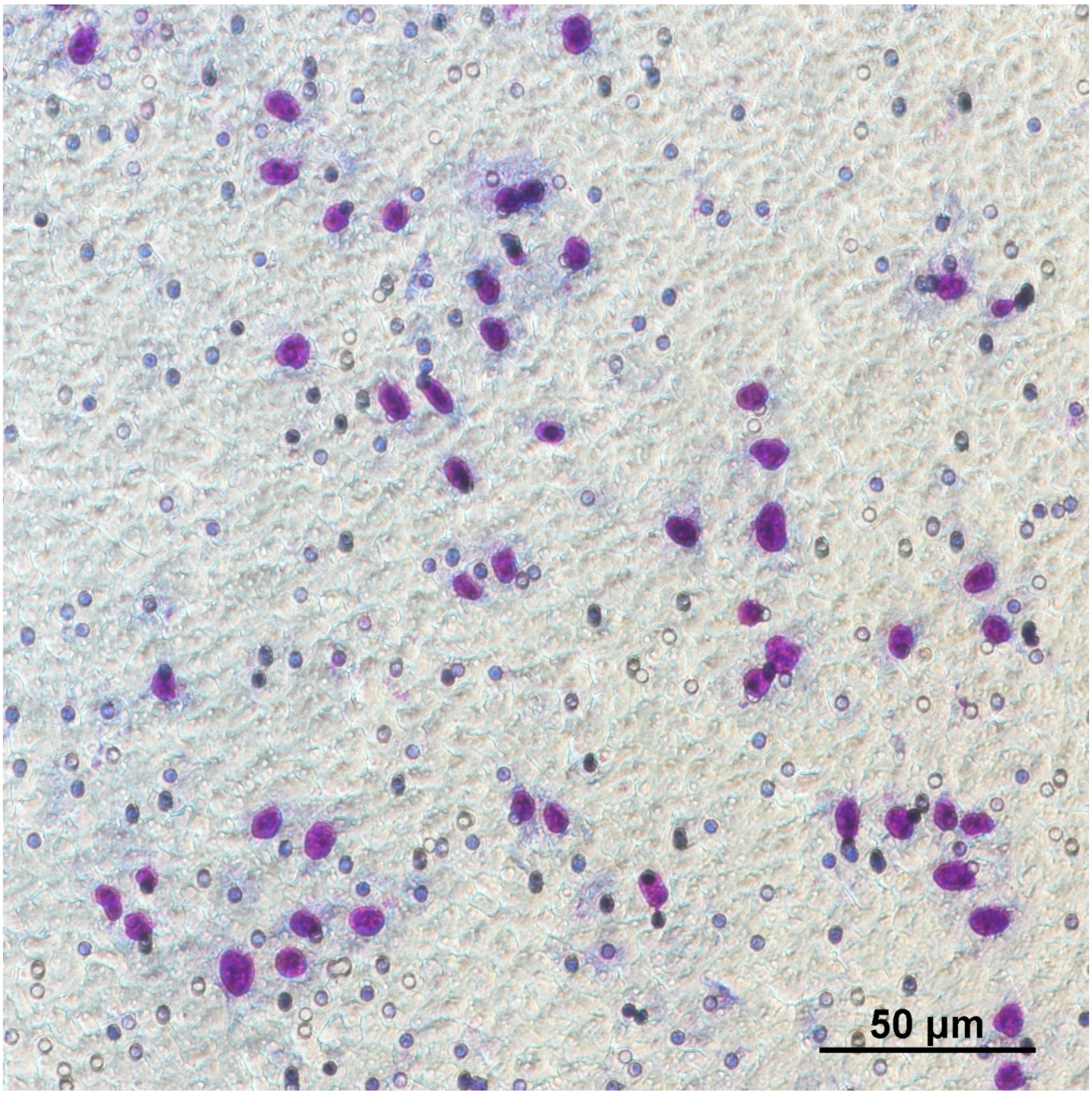

Fig.2D-Ishikawa-invasion-sh-IGF2BP3-1

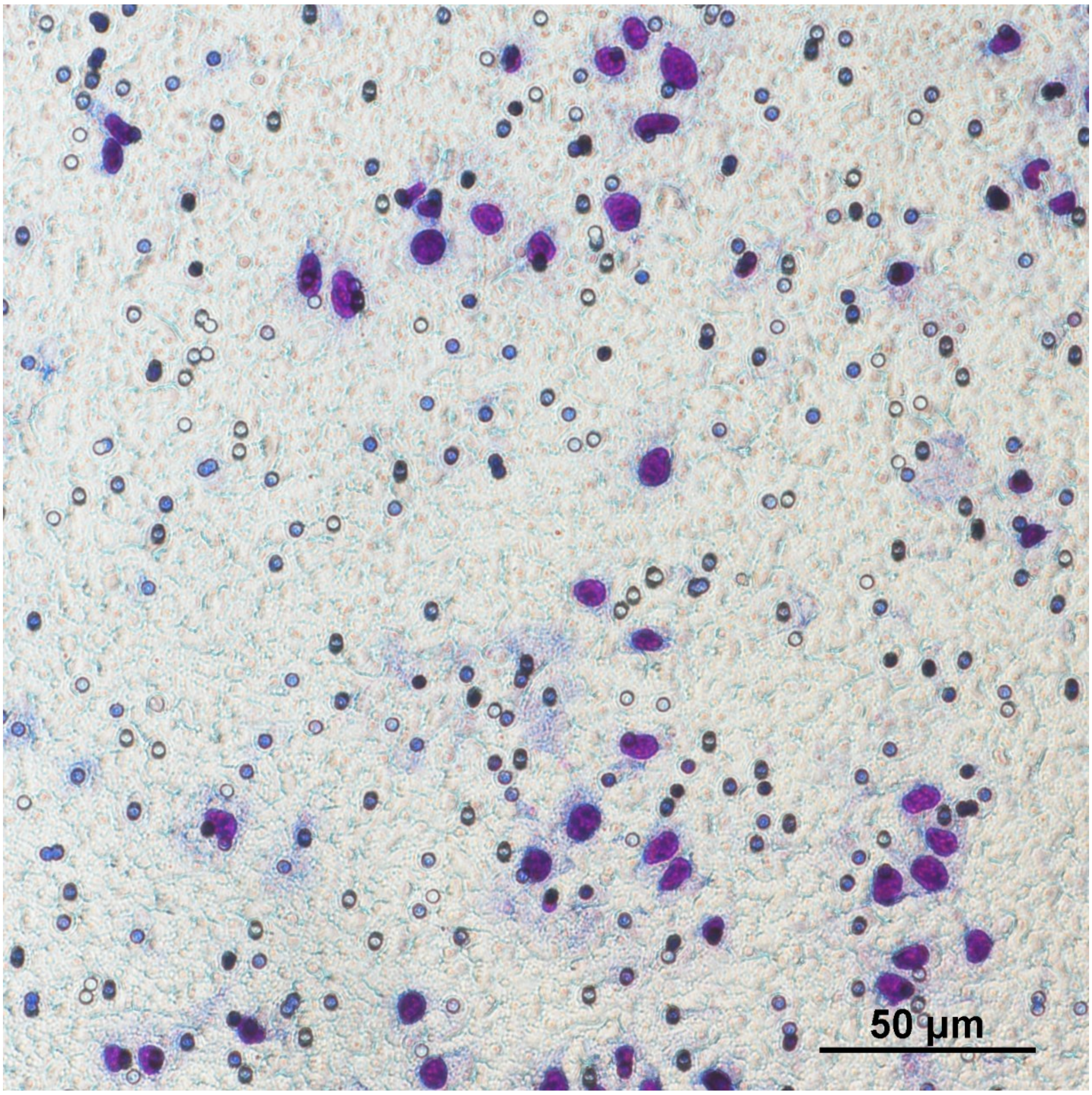

Fig.2D-Ishikawa-invasion-sh-IGF2BP3-3

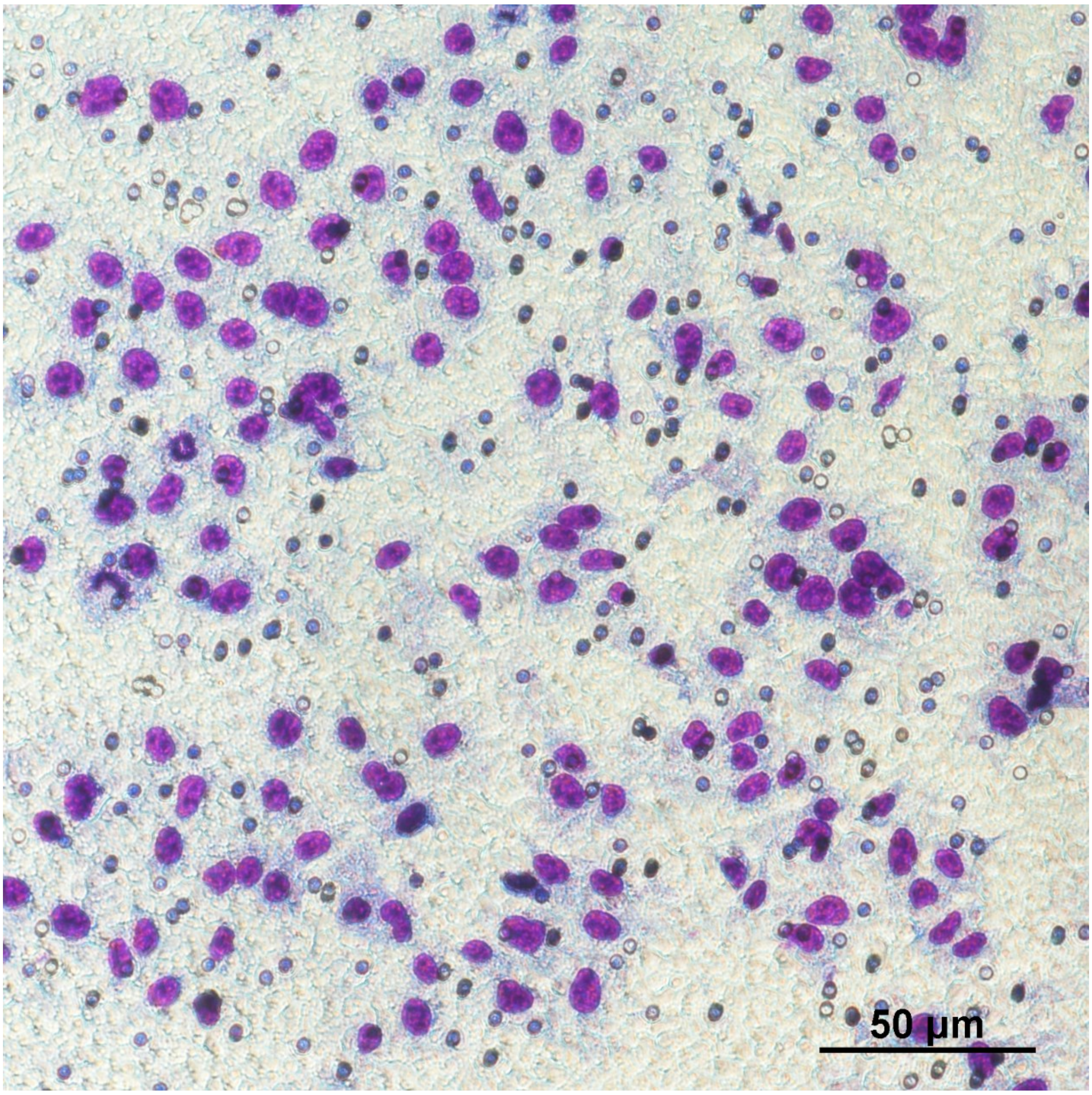

Fig.2D-Ishikawa-invasion-sh-NC

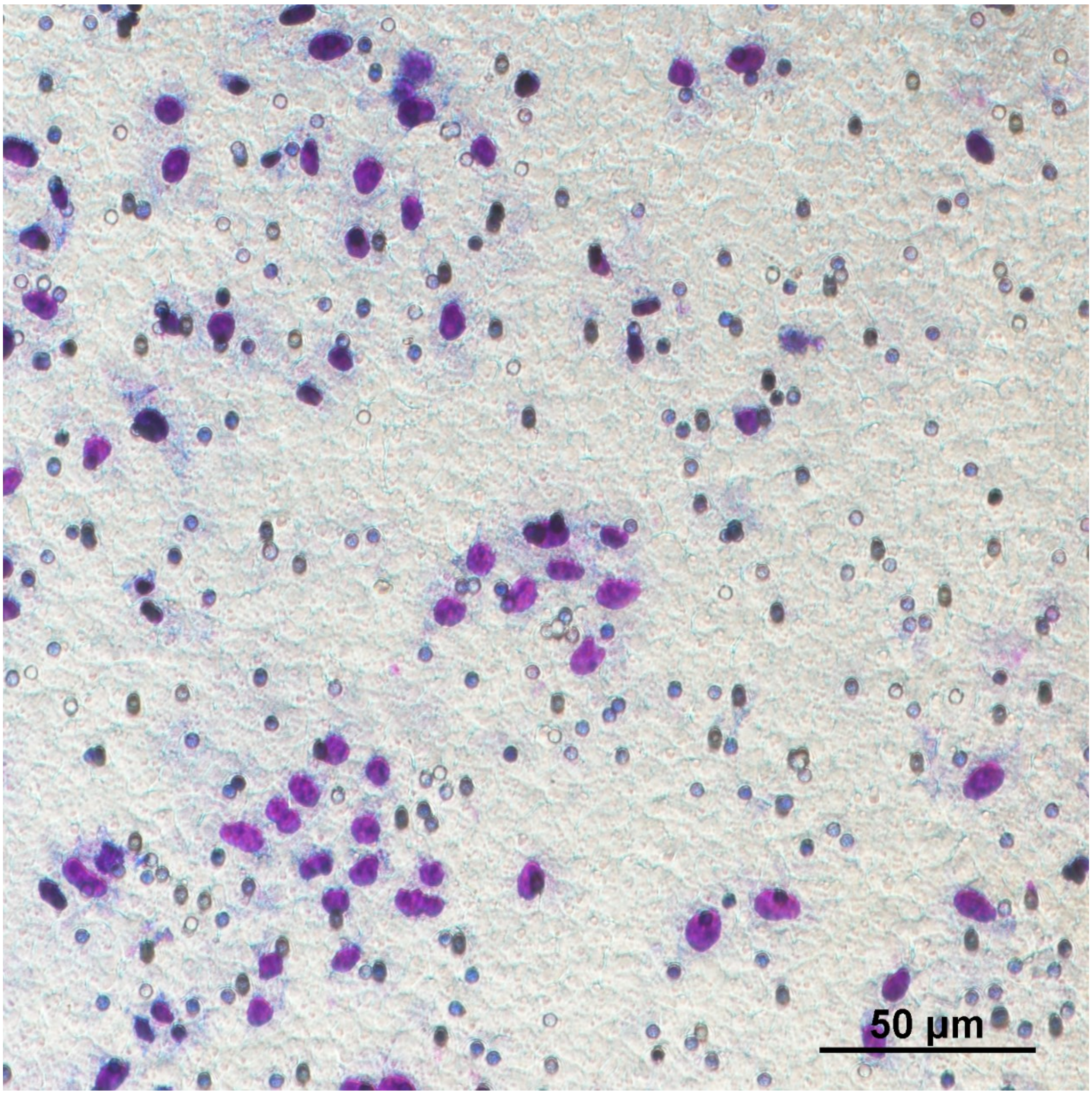

Fig.2D-Ishikawa-migration-sh-IGF2BP3-1

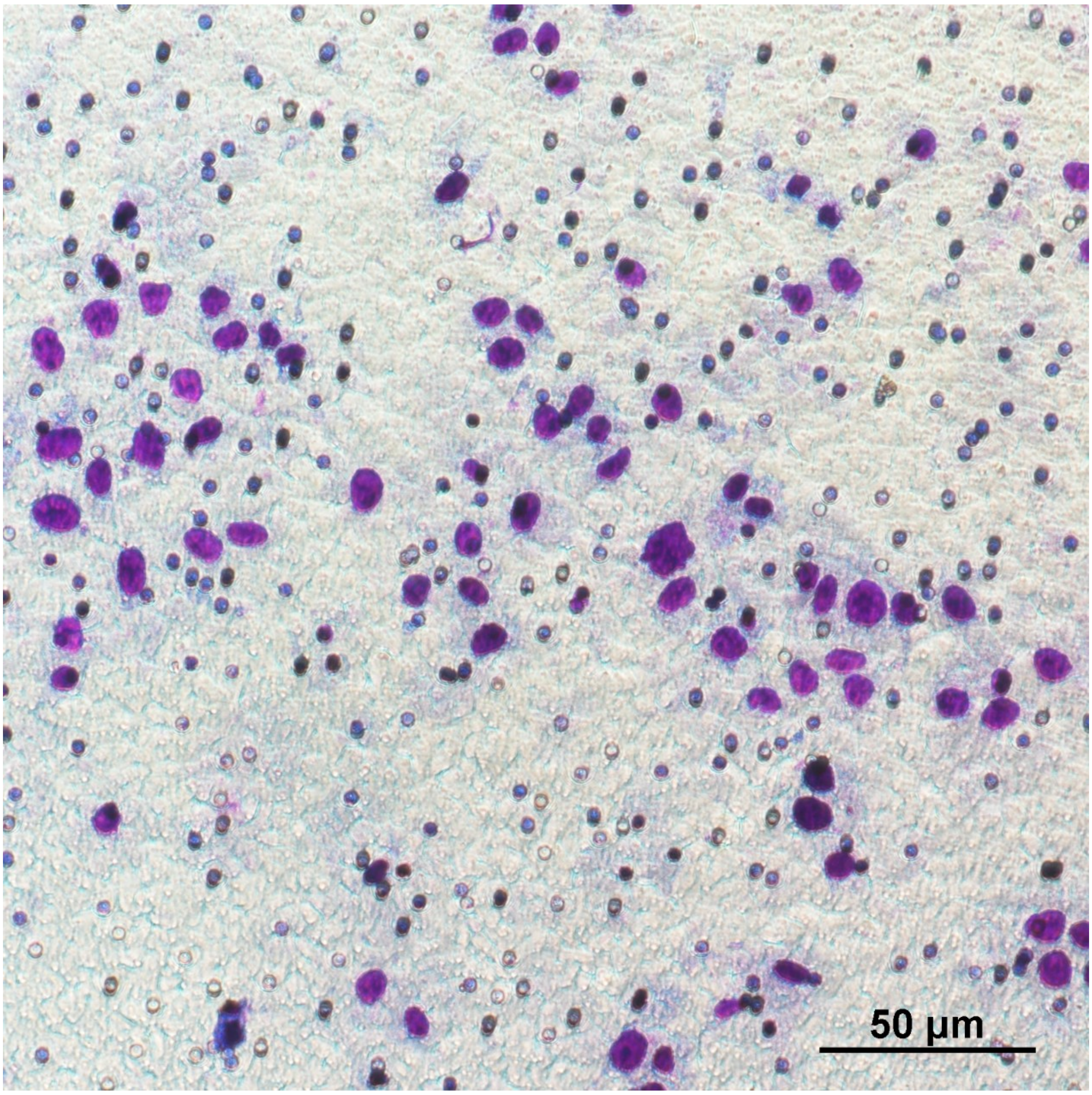

Fig.2D-Ishikawa-migration-sh-IGF2BP3-3

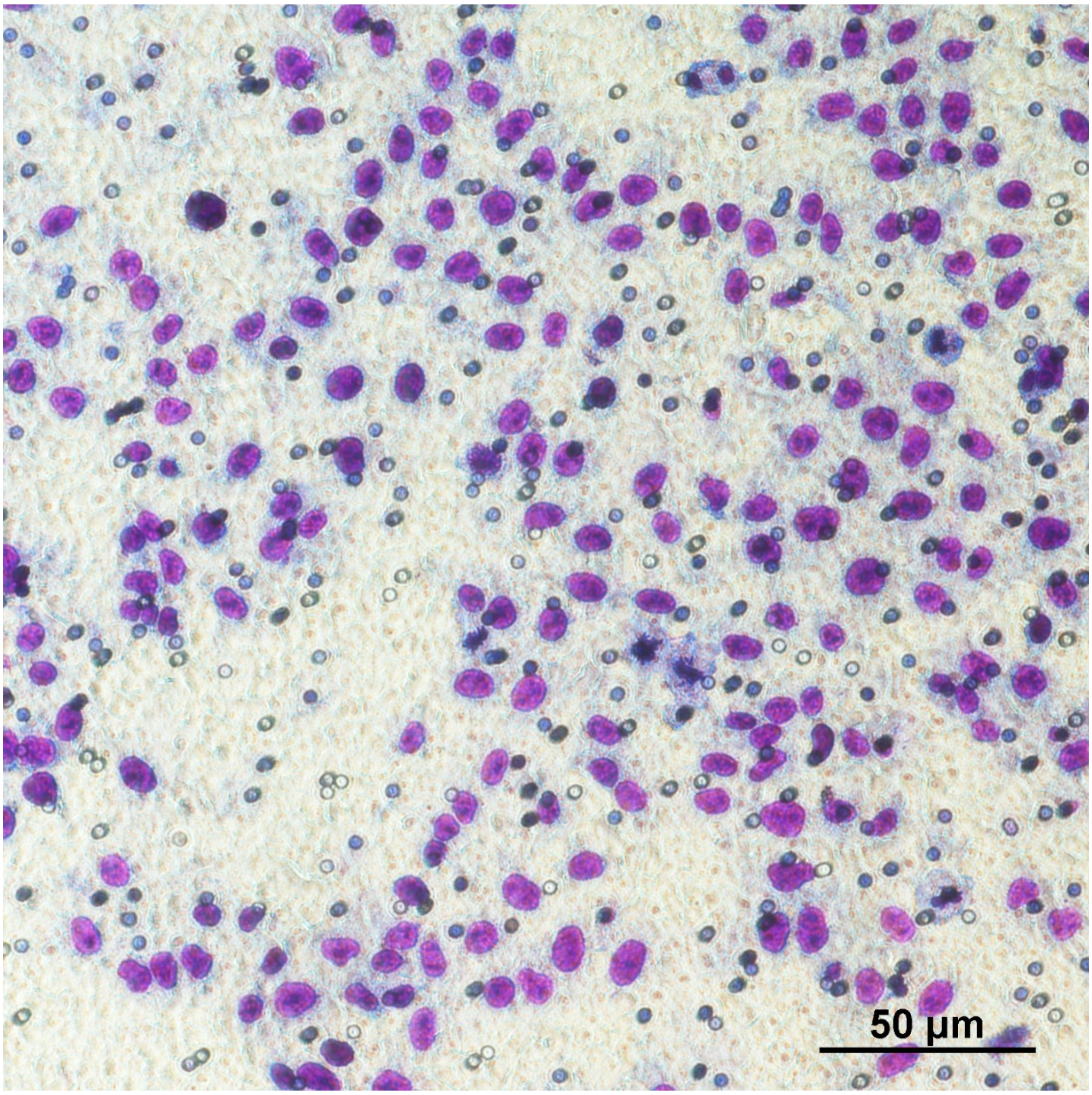

Fig.2D-Ishikawa-migration-sh-NC

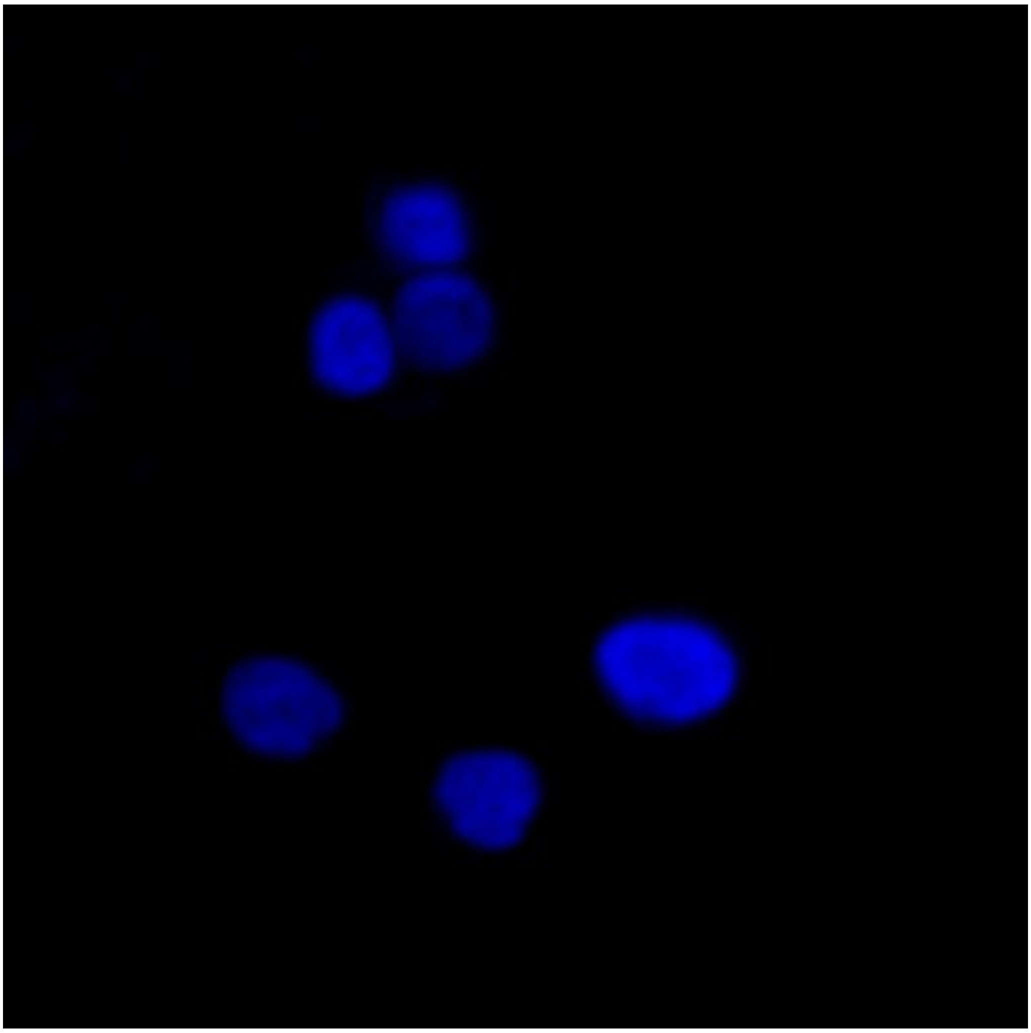

Fig.3L-Ishikawa-DAPI

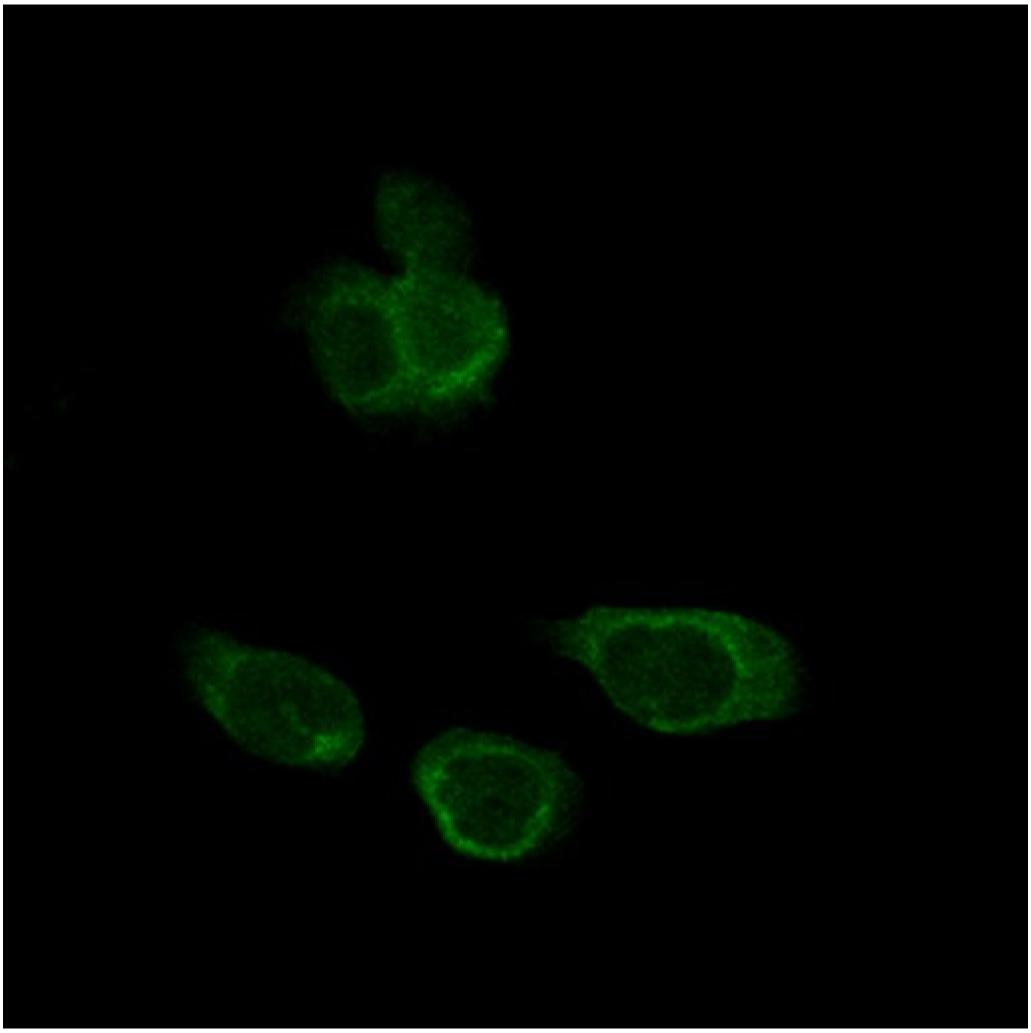

Fig.3L-Ishikawa-IGF2BP3

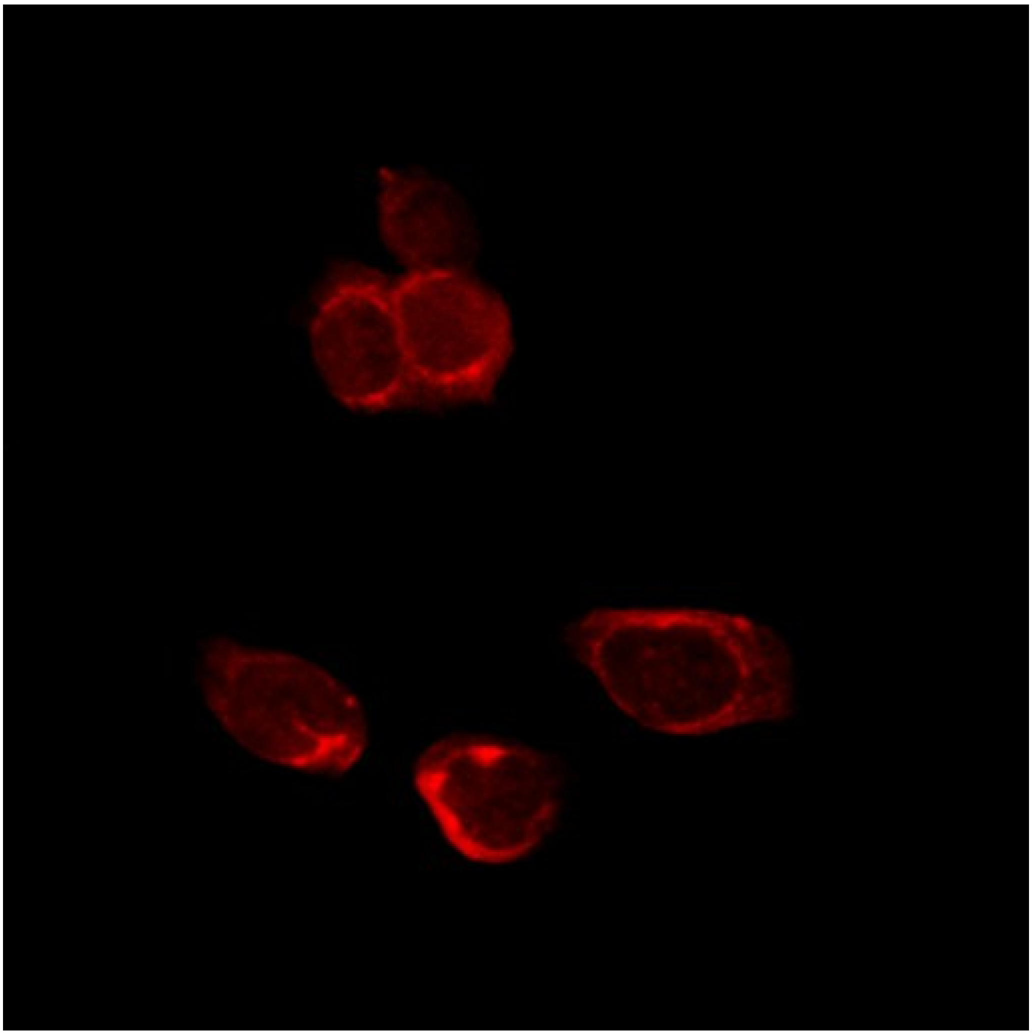

Fig.3L-Ishikawa-LINC00958

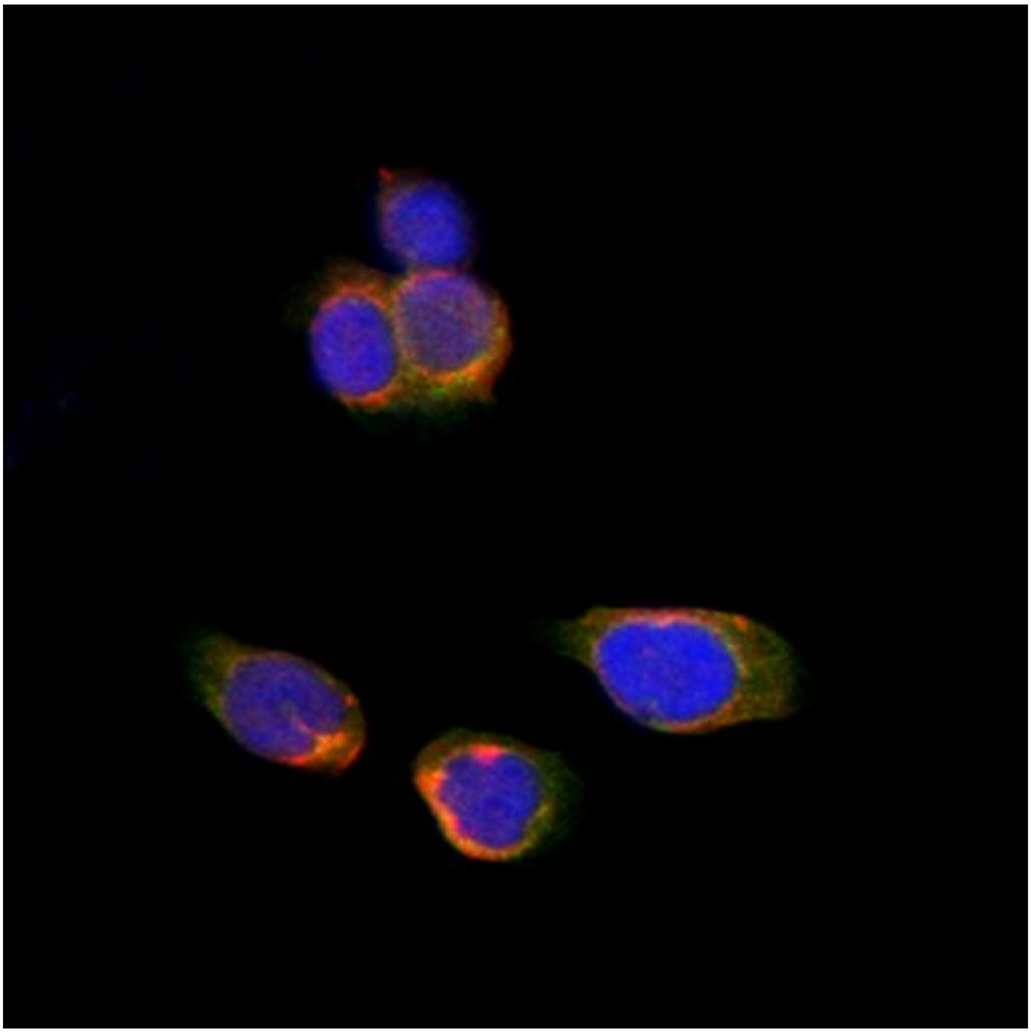

Fig.3L-Ishikawa-Merge

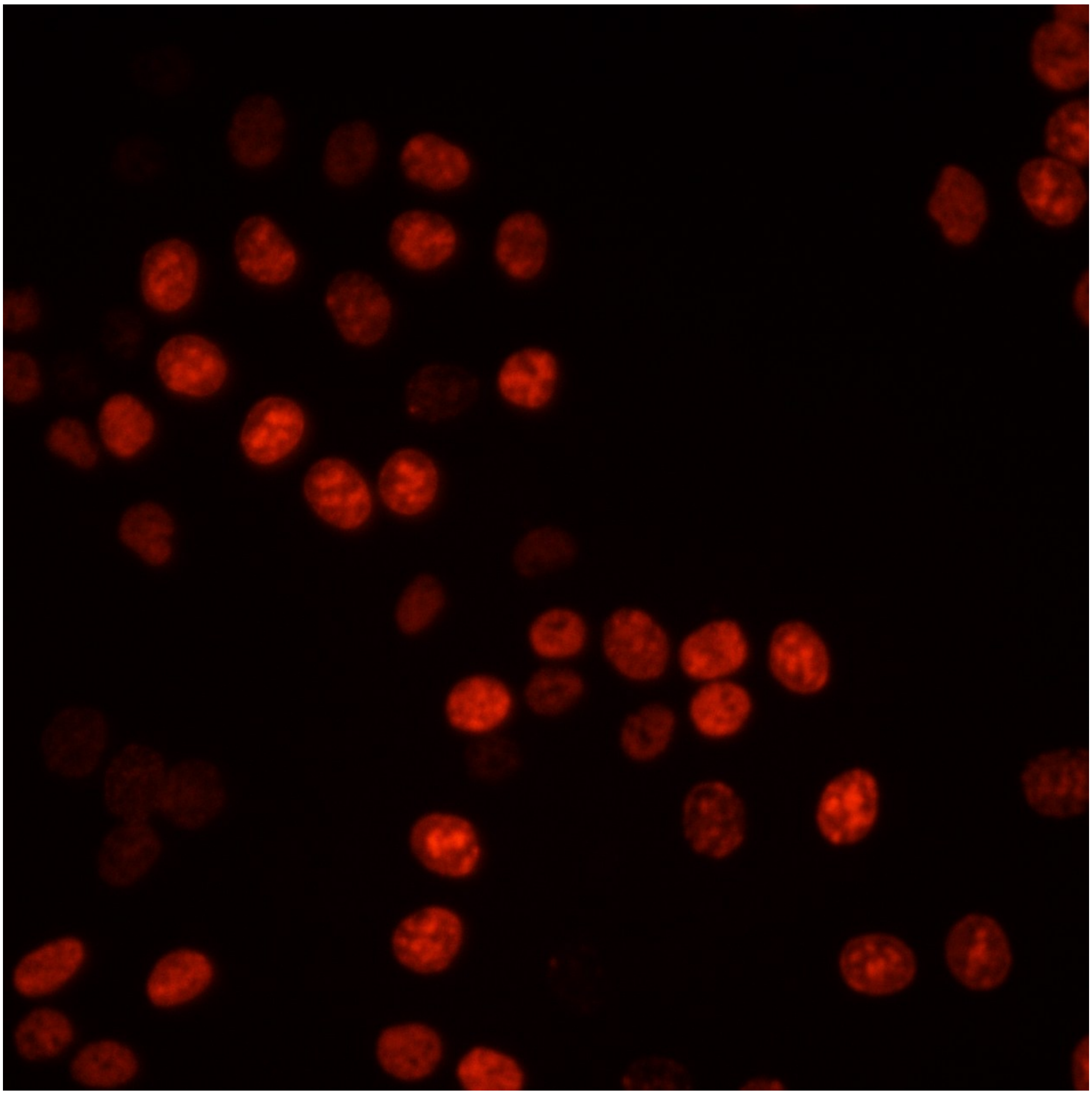

Fig.4C-Ishikawa-IGF2BP3-OE+sh-LINC00958-EdU

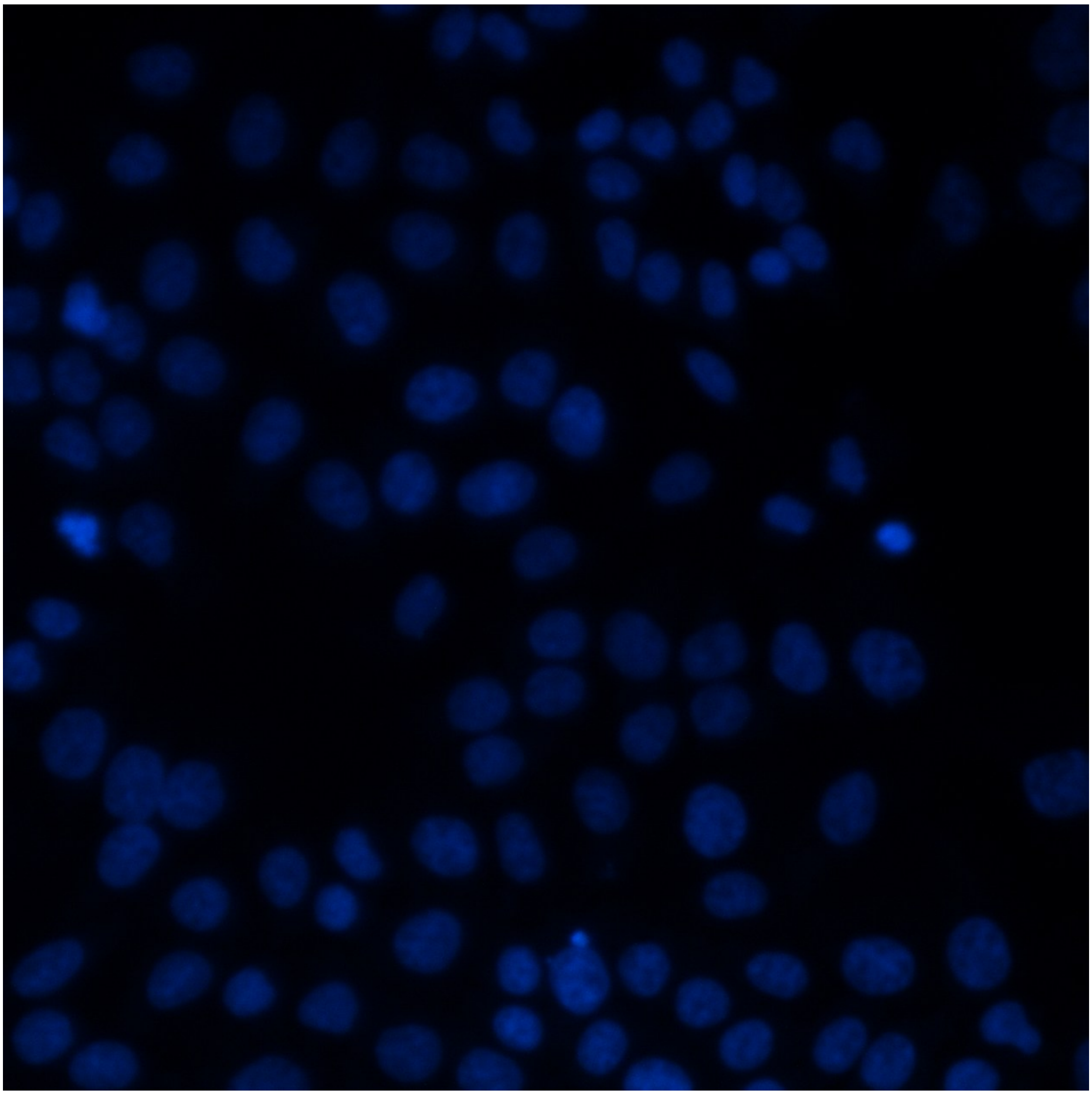

Fig.4C-Ishikawa-IGF2BP3-OE+sh-LINC00958-Hoechst

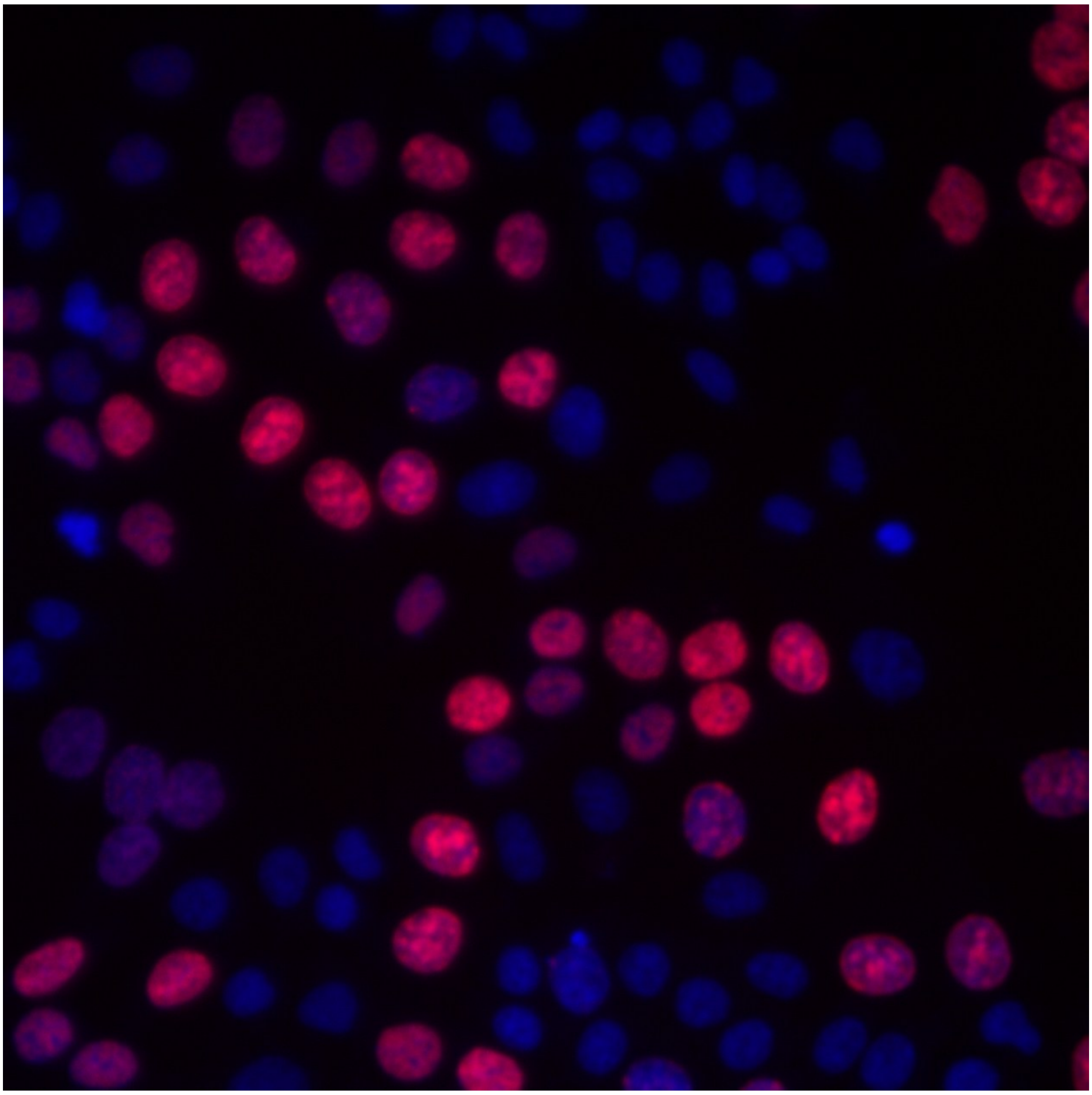
Fig.4C-Ishikawa-IGF2BP3-OE+sh-LINC00958-Merge

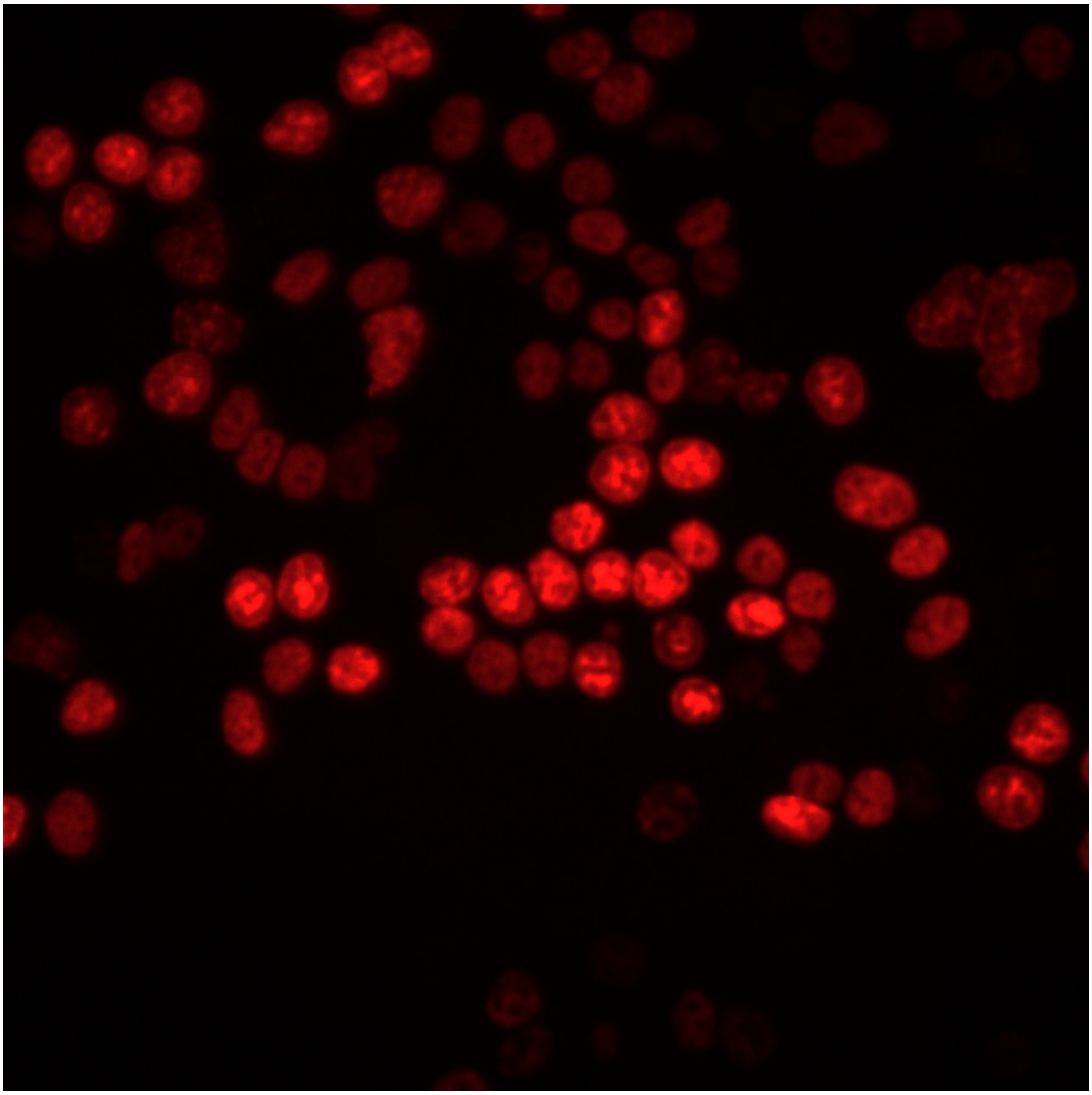

Fig.4C-Ishikawa-IGF2BP3-OE+sh-NC-EdU

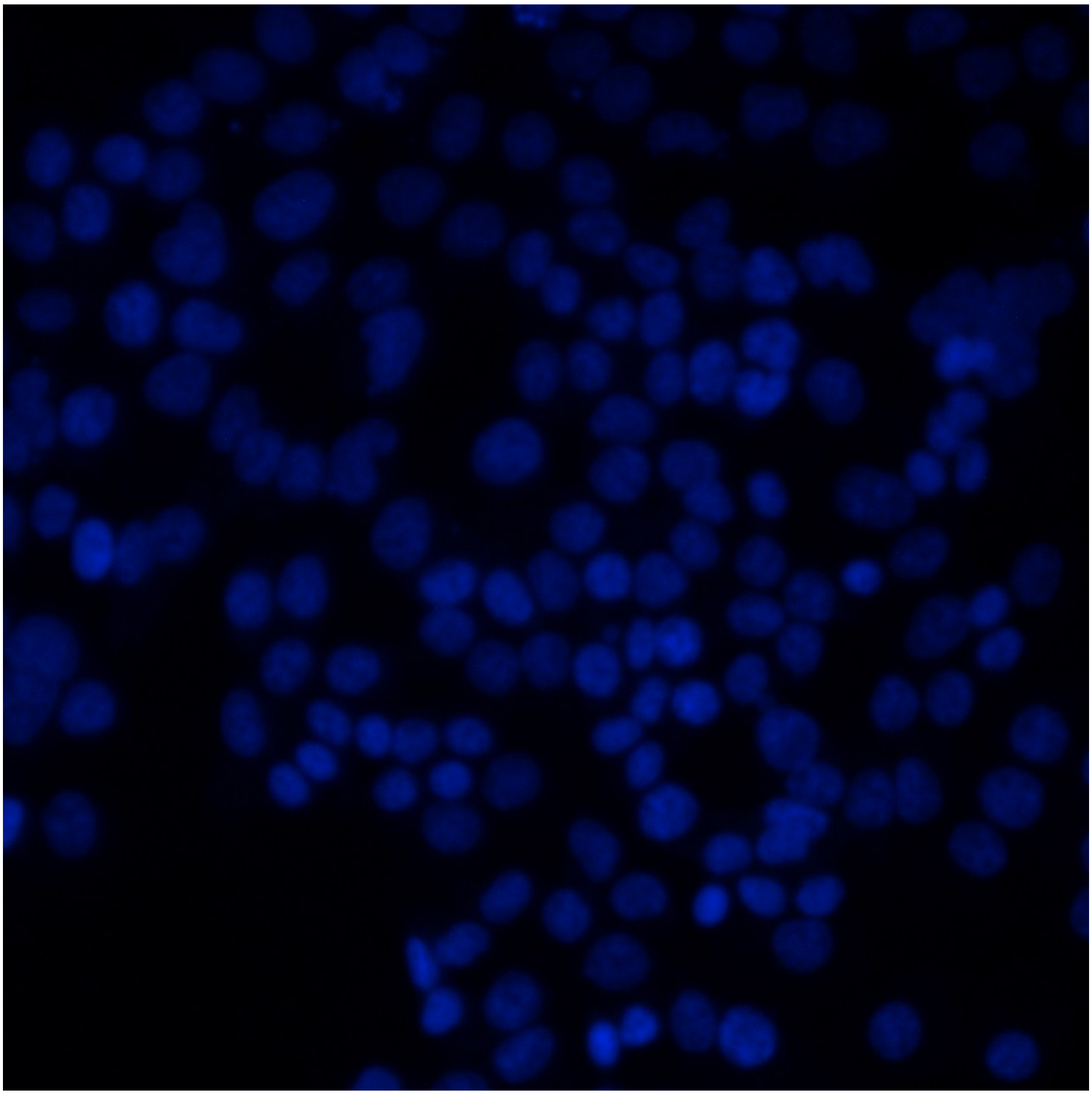
Fig.4C-Ishikawa-IGF2BP3-OE+sh-NC-Hoechst

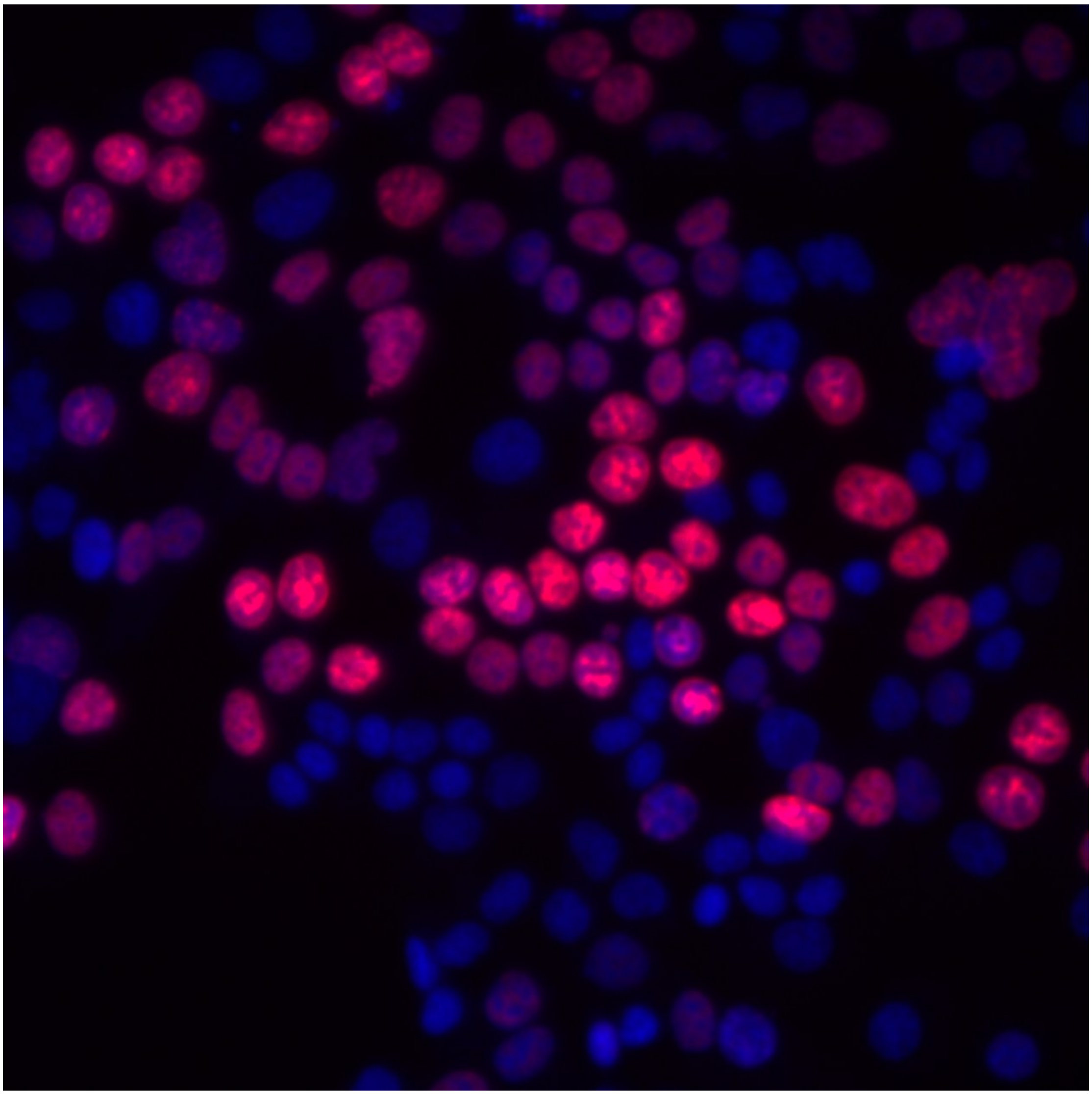

Fig.4C-Ishikawa-IGF2BP3-OE+sh-NC-Merge

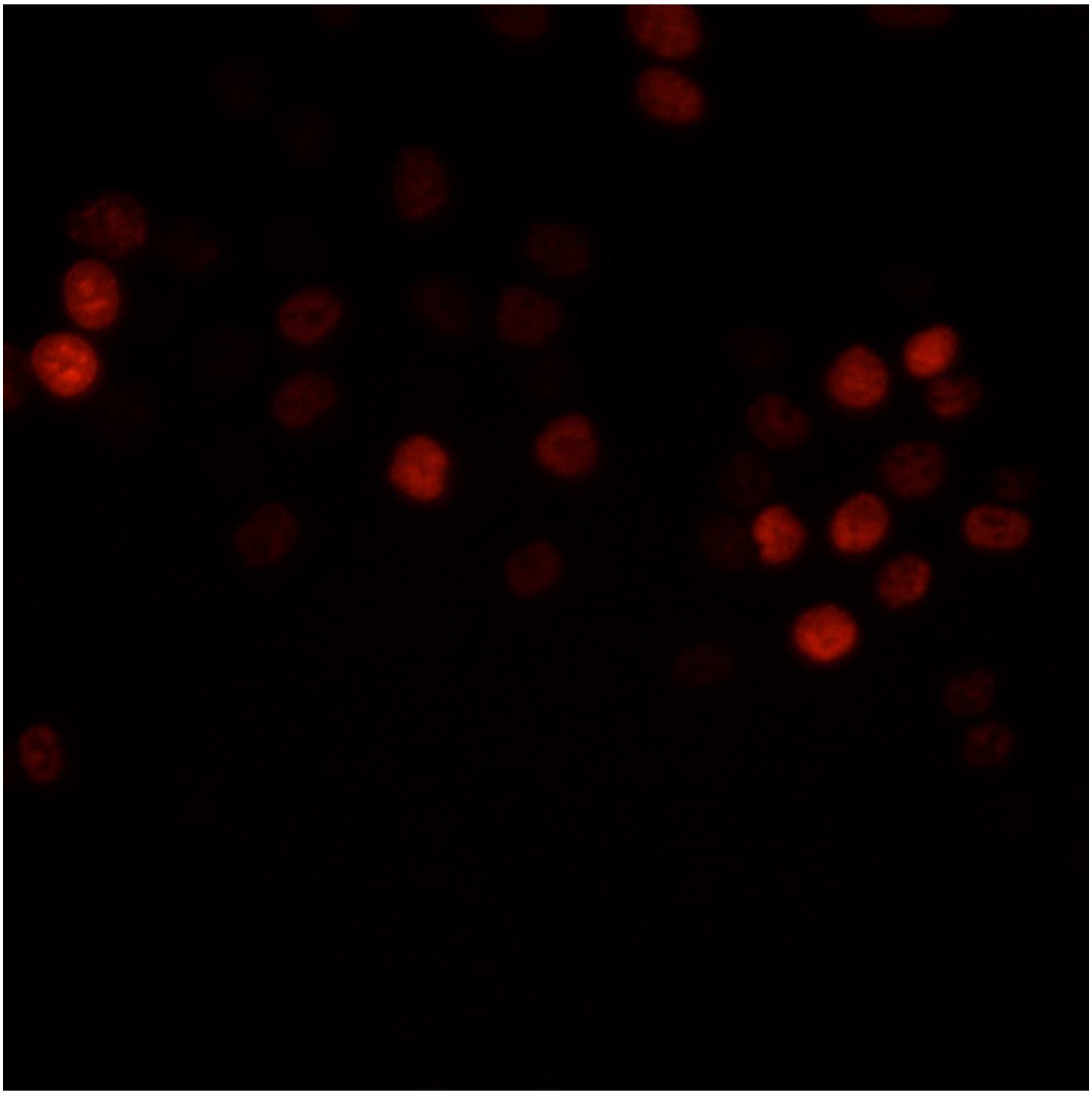

Fig.4C-Ishikawa-LV-NC+sh-LINC00958-EdU

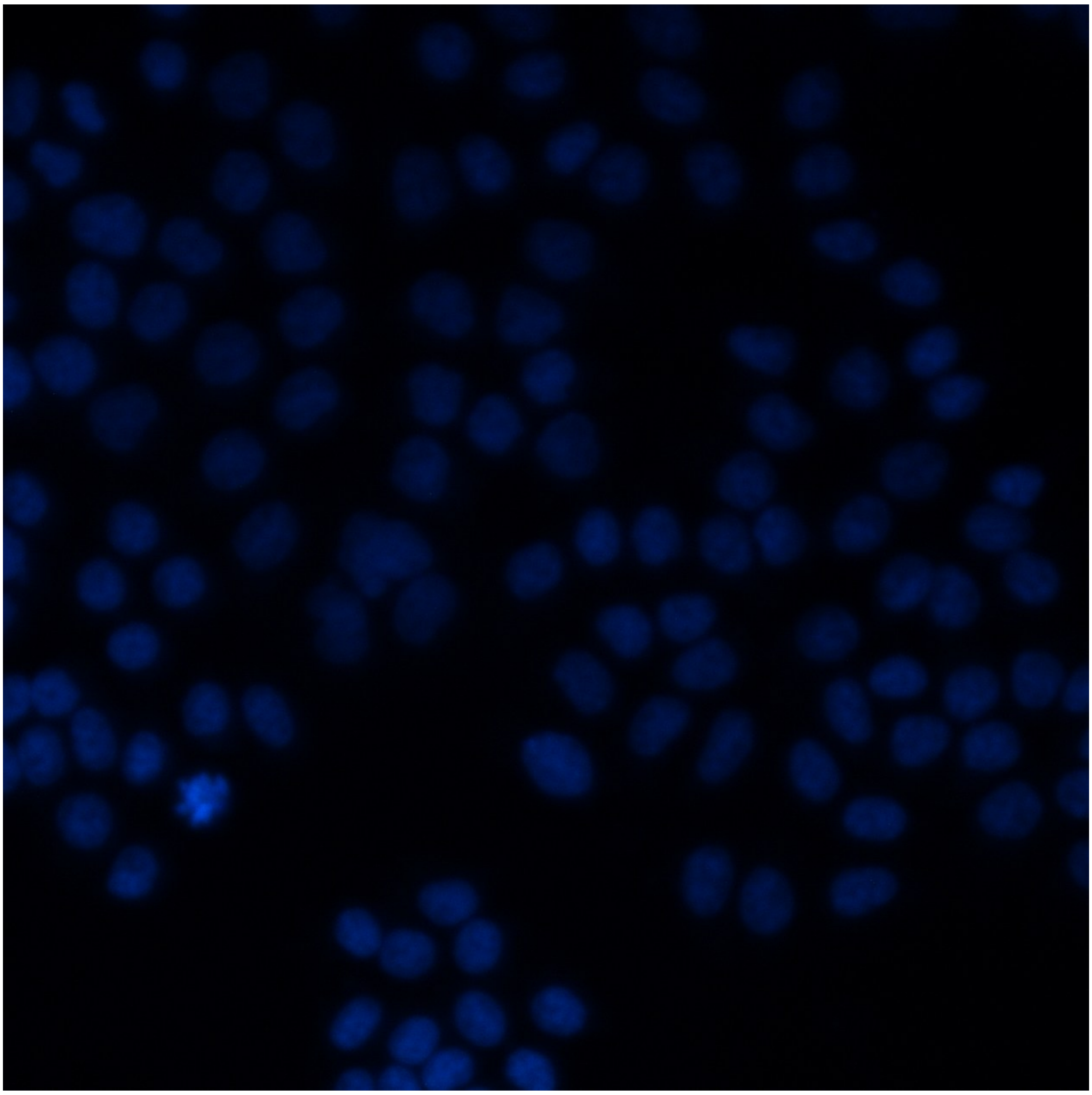

Fig.4C-Ishikawa-LV-NC+sh-LINC00958-Hoechst

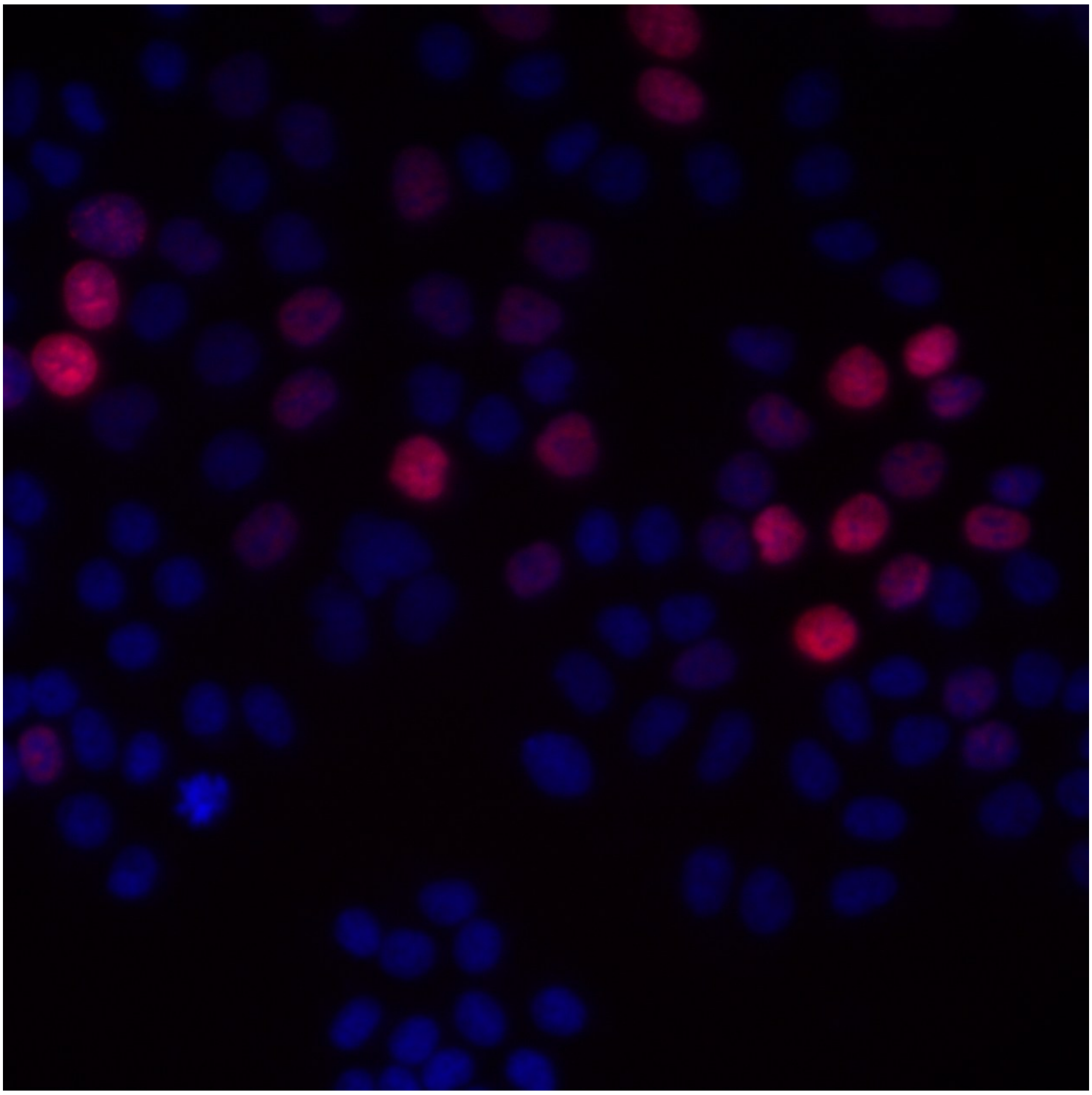

Fig.4C-Ishikawa-LV-NC+sh-LINC00958-Merge

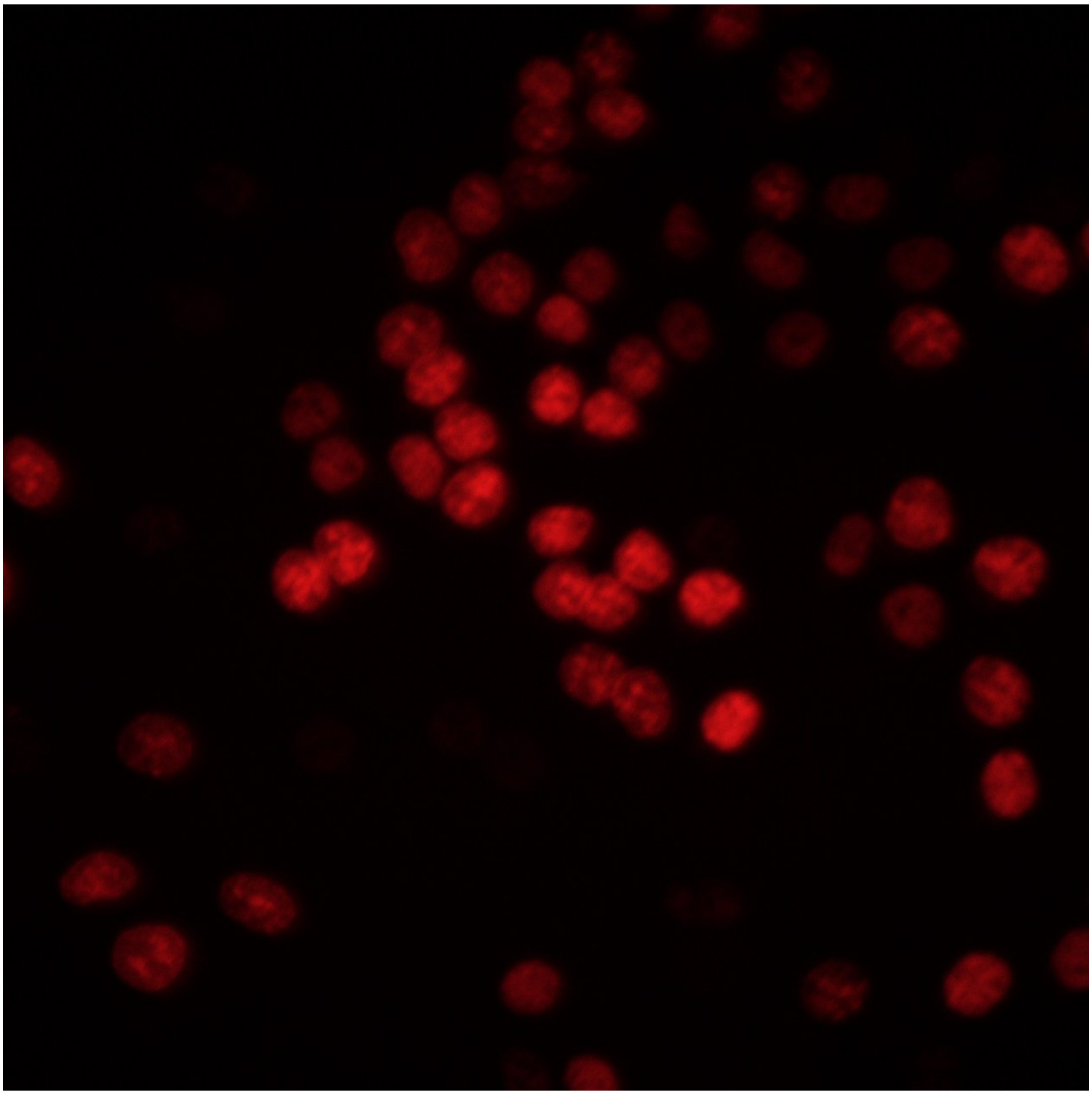

Fig.4C-Ishikawa-LV-NC+sh-NC-EdU

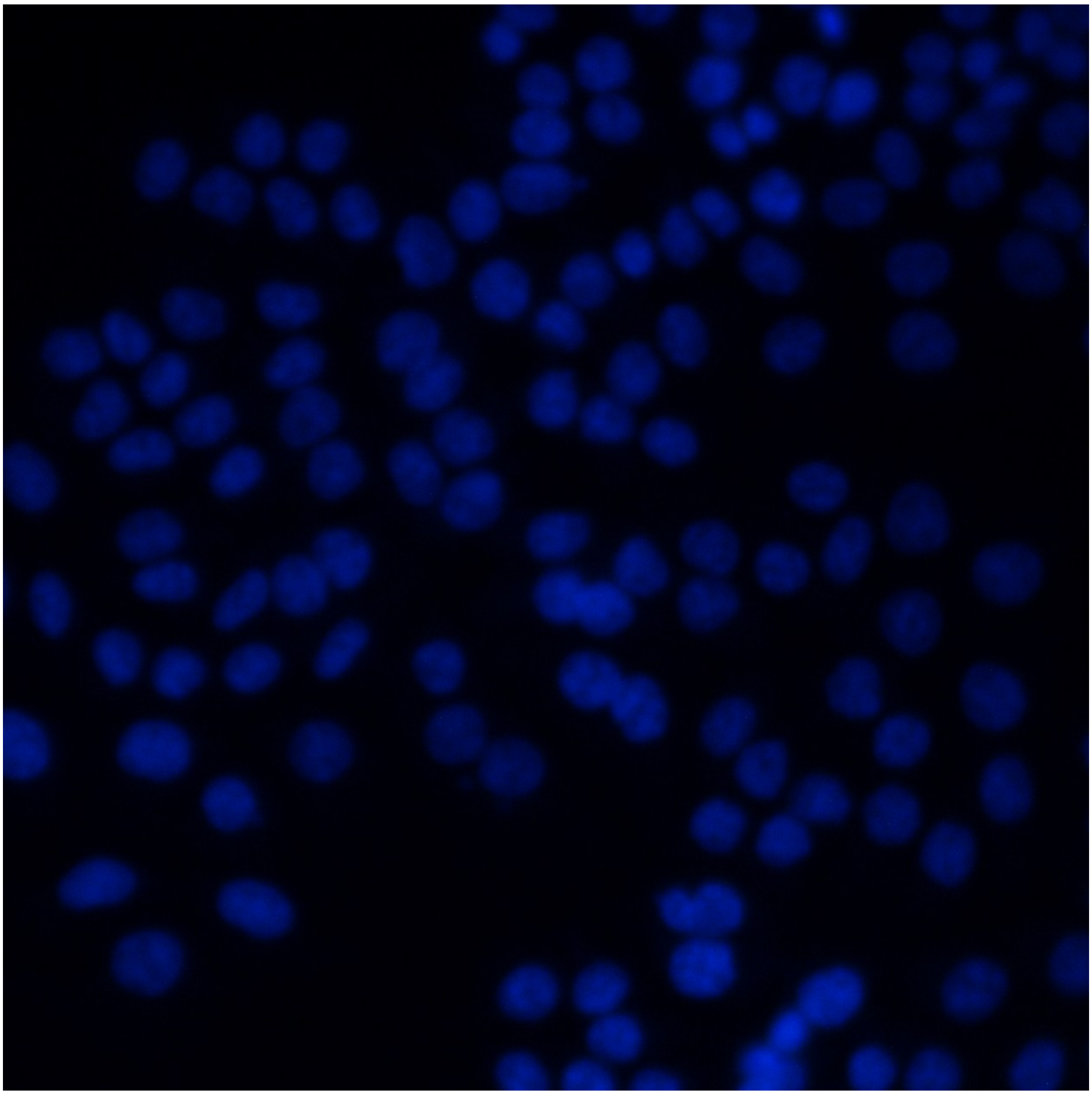

Fig.4C-Ishikawa-LV-NC+sh-NC-Hoechst

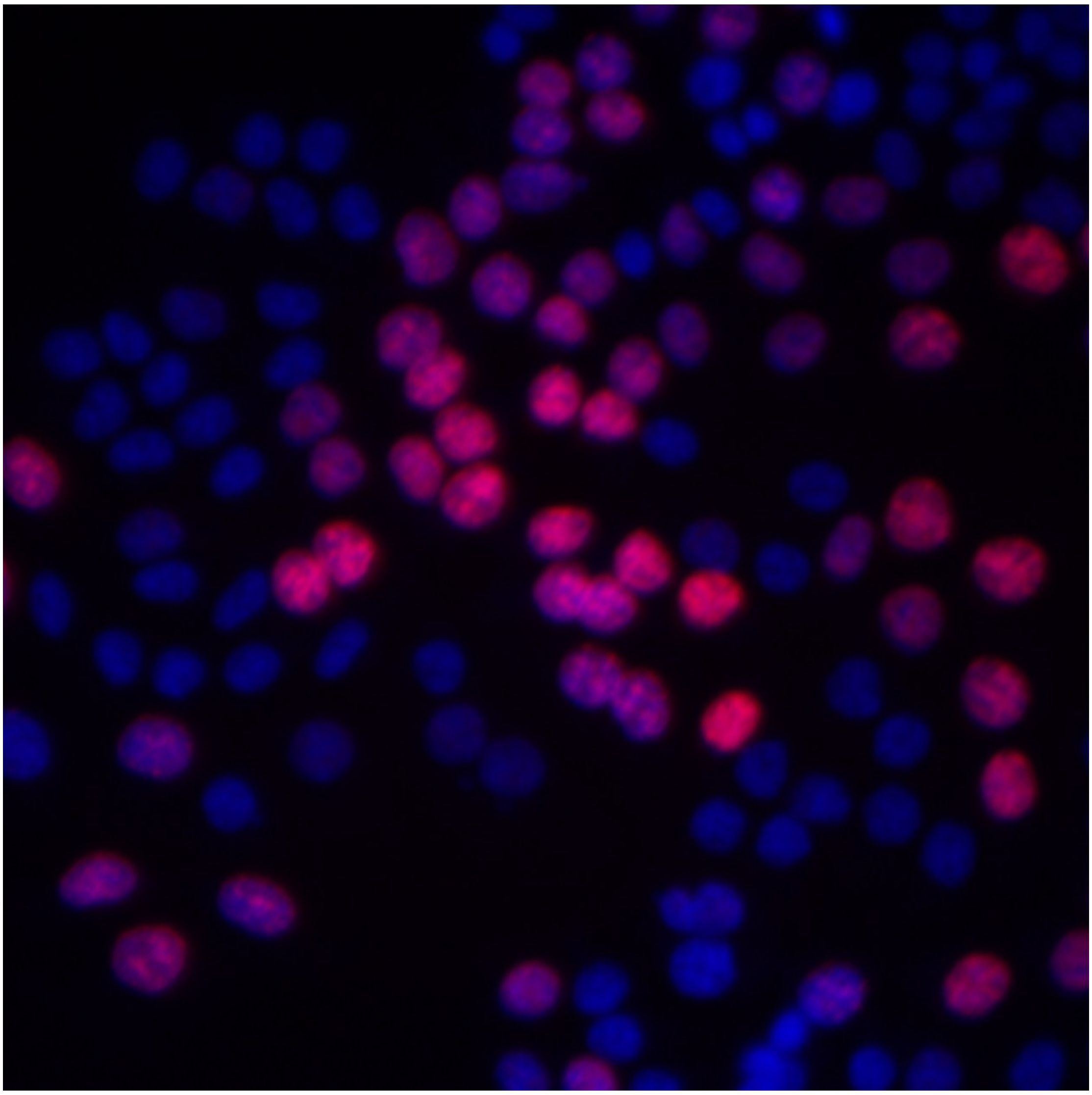

Fig.4C-Ishikawa-LV-NC+sh-NC-Merge

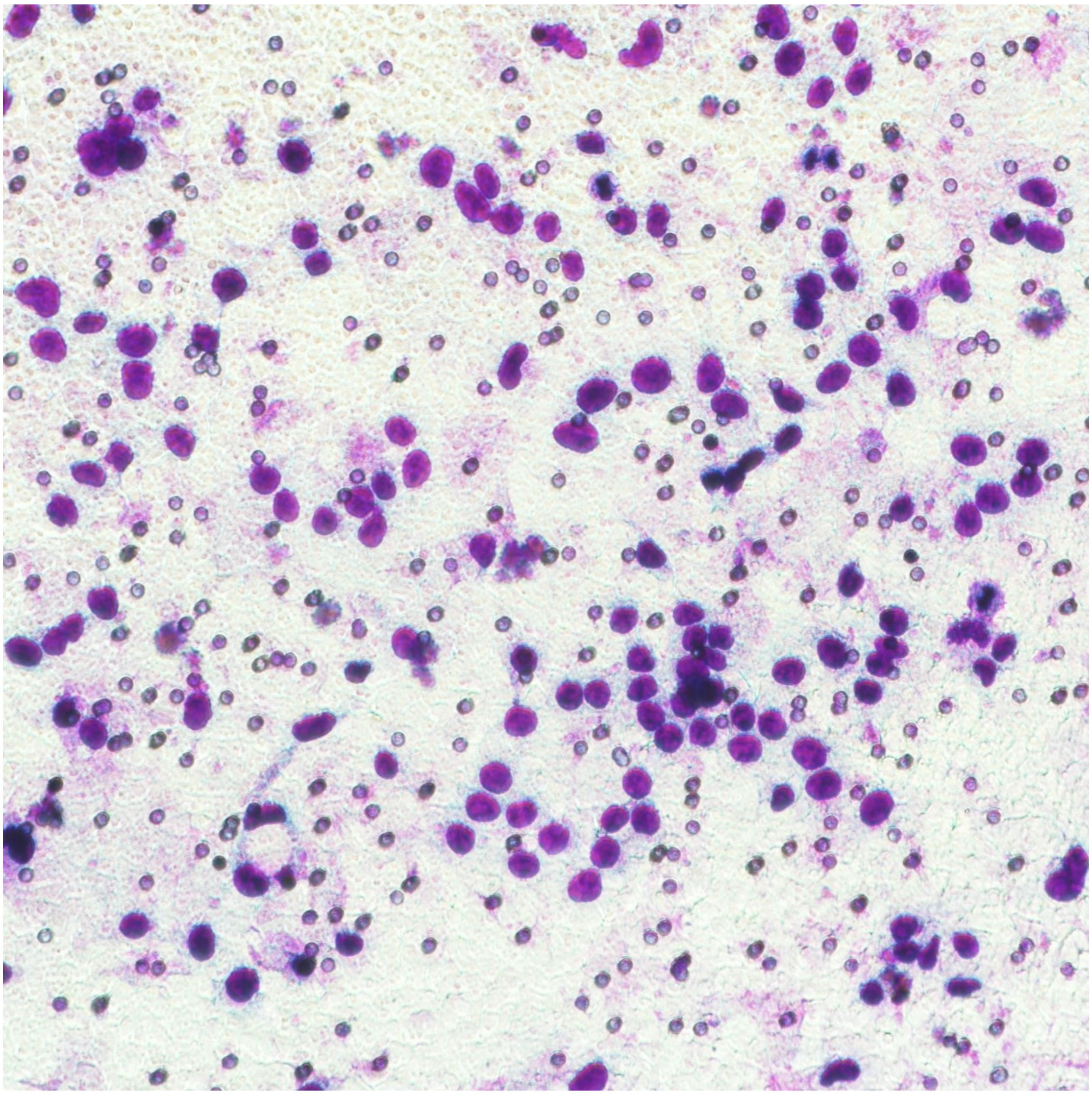

Fig.4D-HEC-1-A-Invasion-IGF2BP3-OE+sh-LINC00958

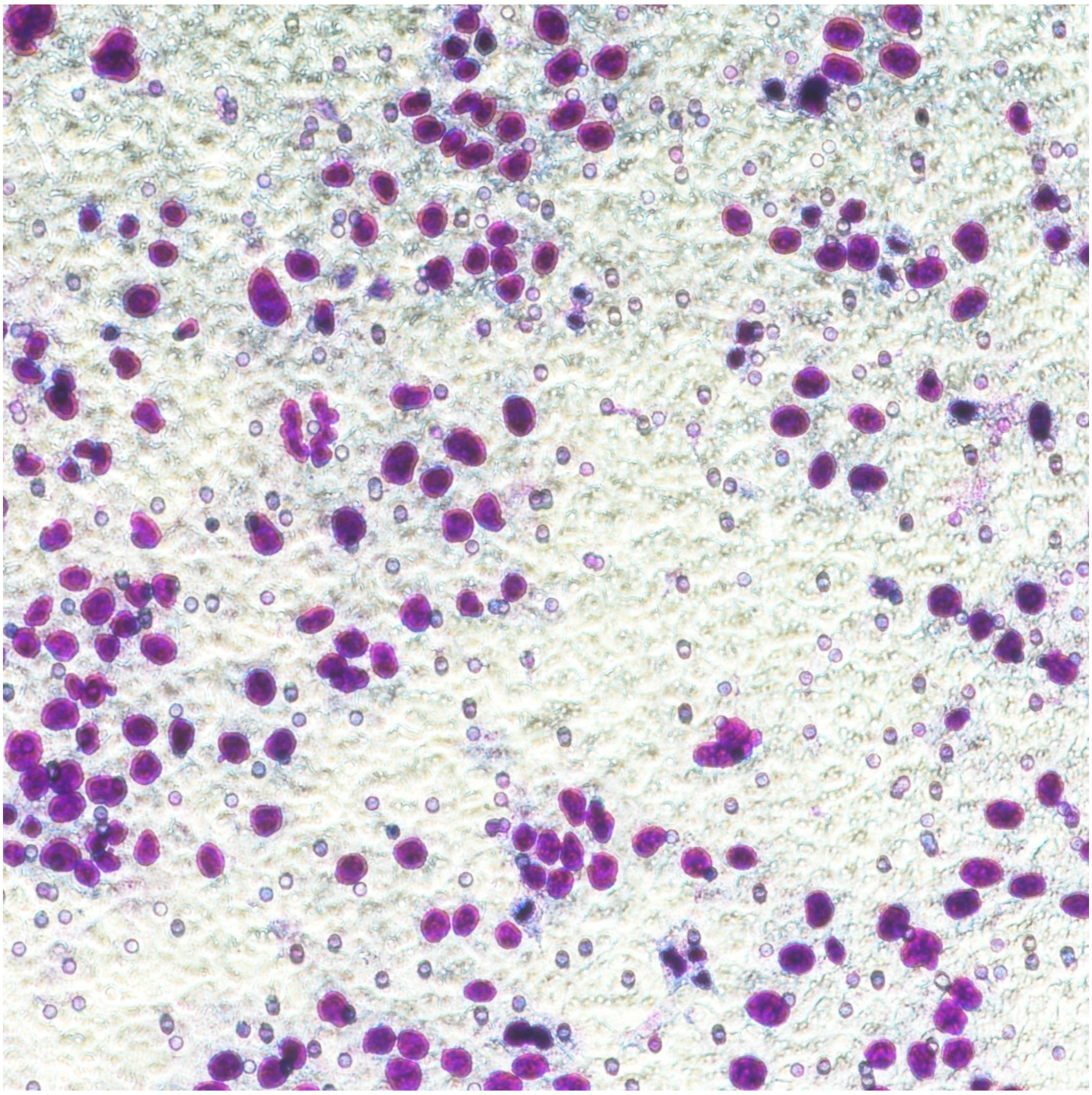

Fig.4D–Ishikawa-Invasion-IGF2BP3-OE+sh-NC

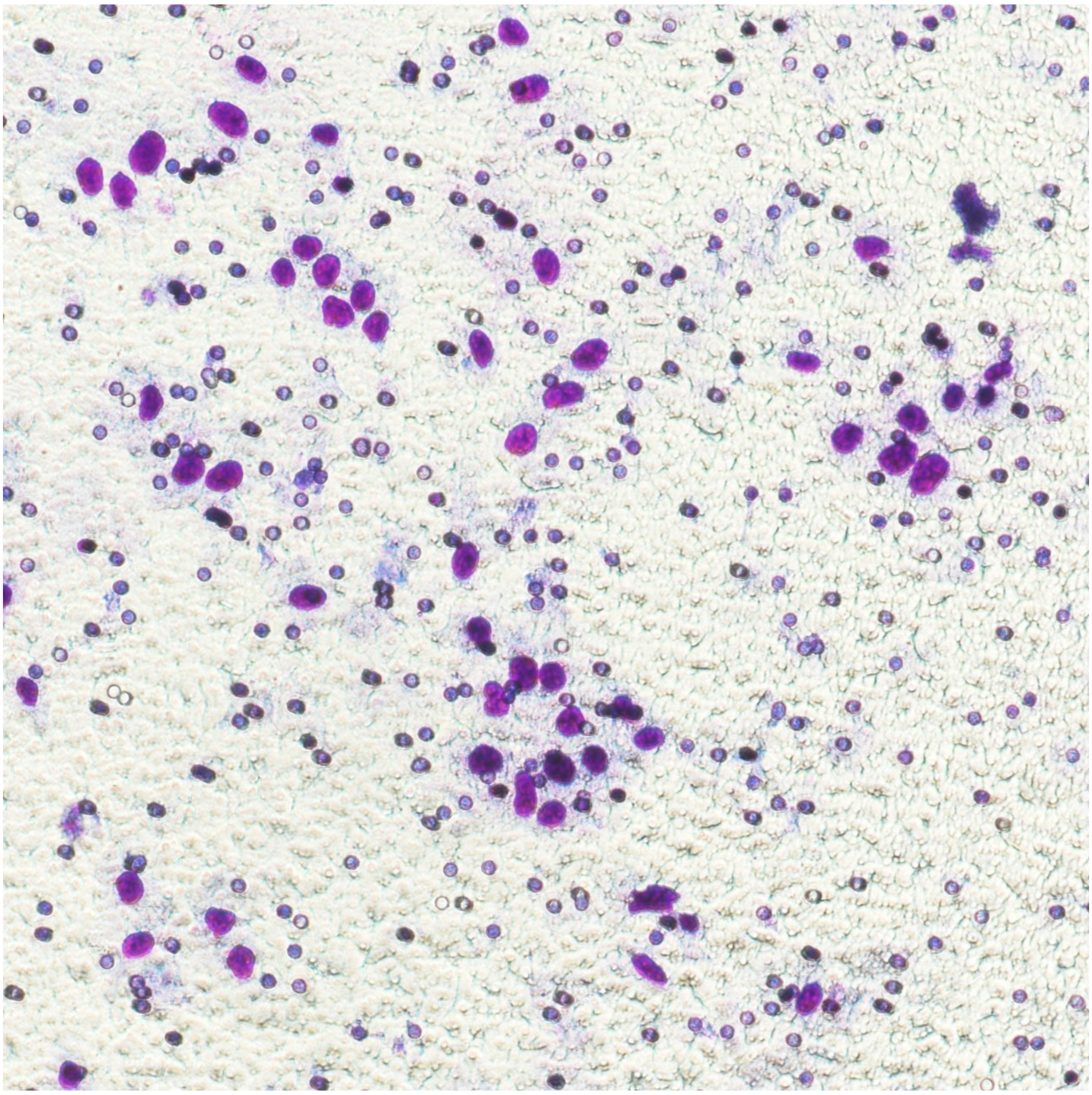
Fig.4D-Ishikawa-Invasion-LV-NC+sh-LINC00958

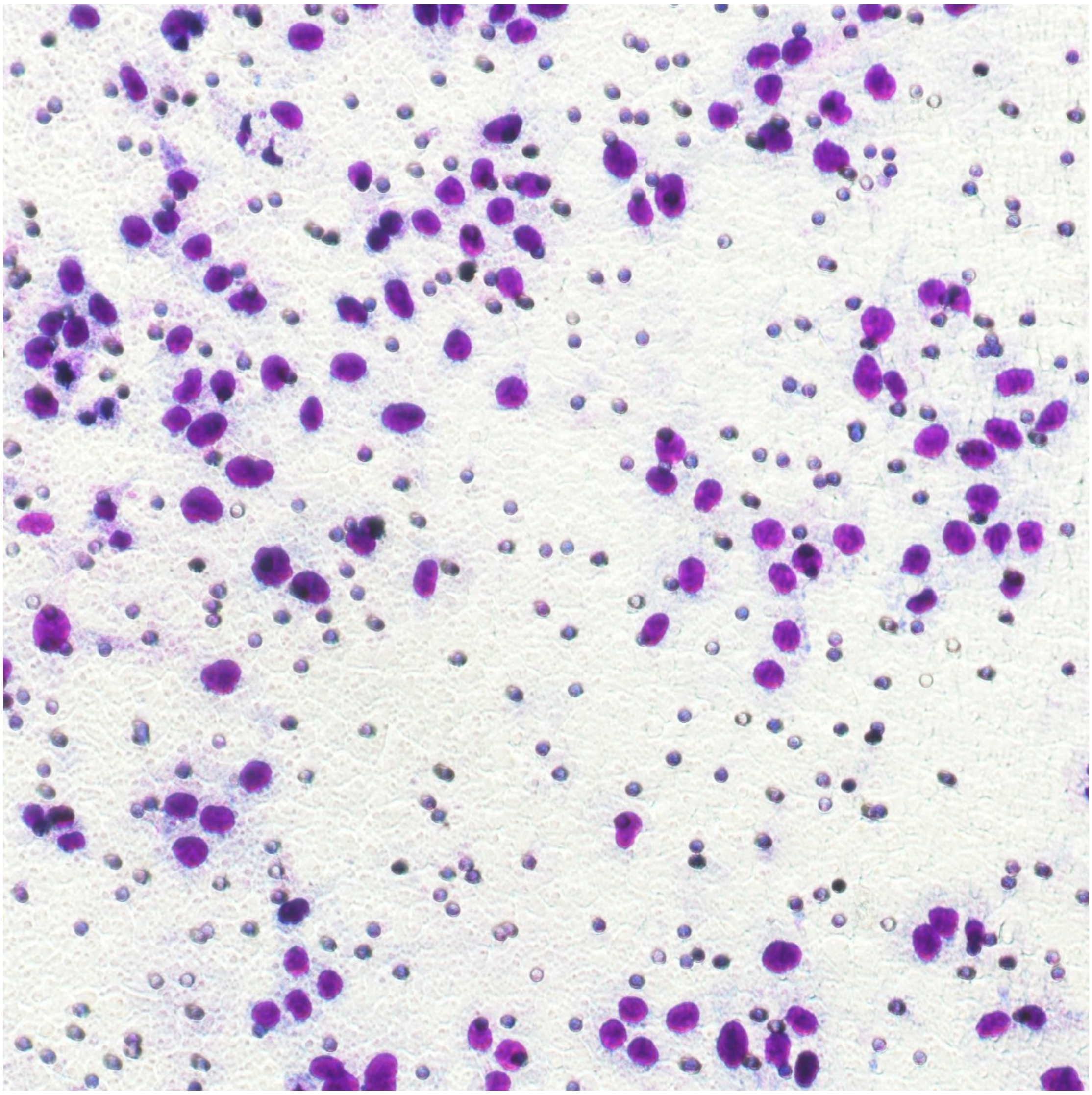

Fig.4D-Ishikawa-Invasion-LV-NC+sh-NC

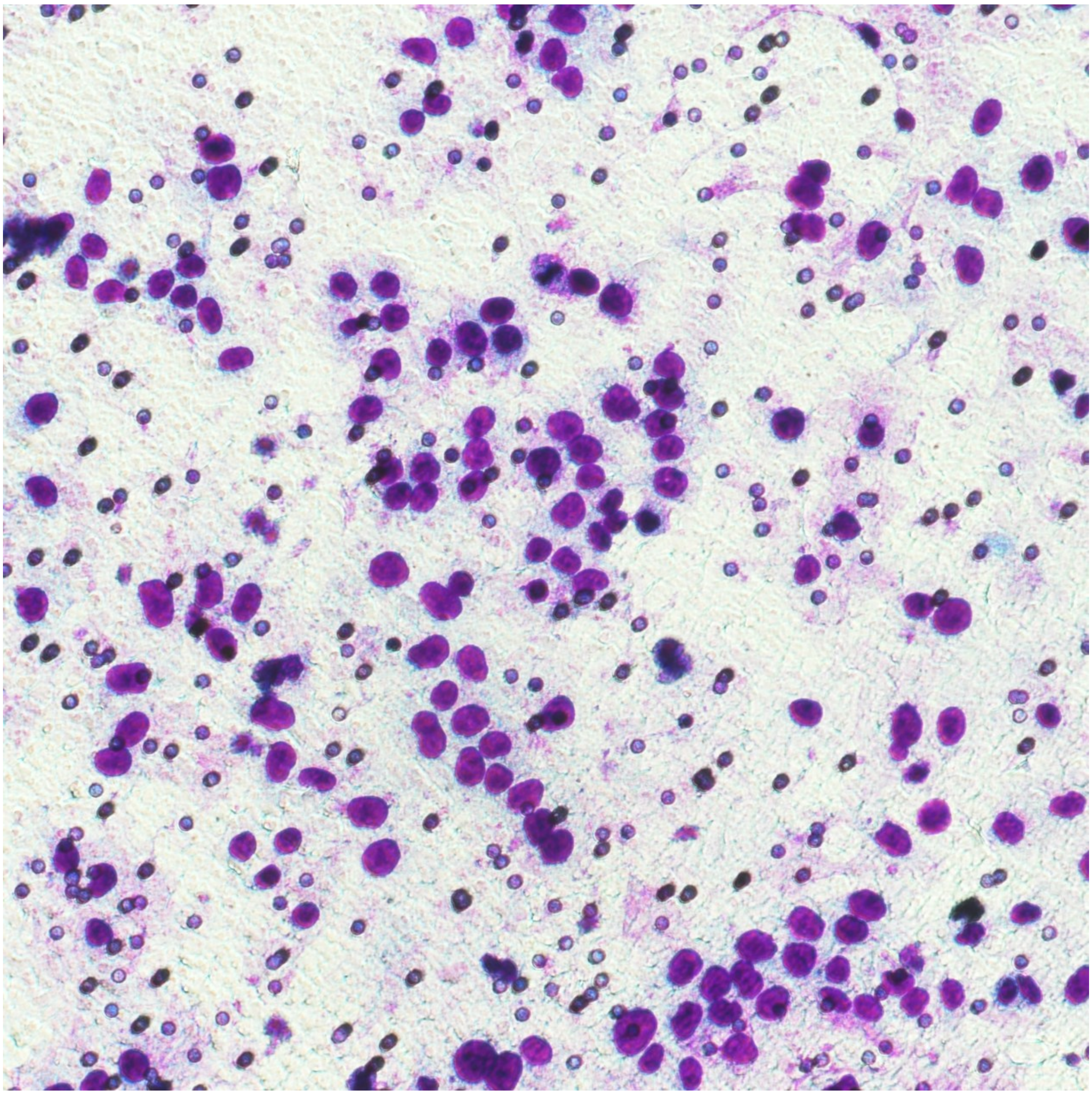

Fig.4D-Ishikawa-Migration-IGF2BP3-OE+sh-LINC00958

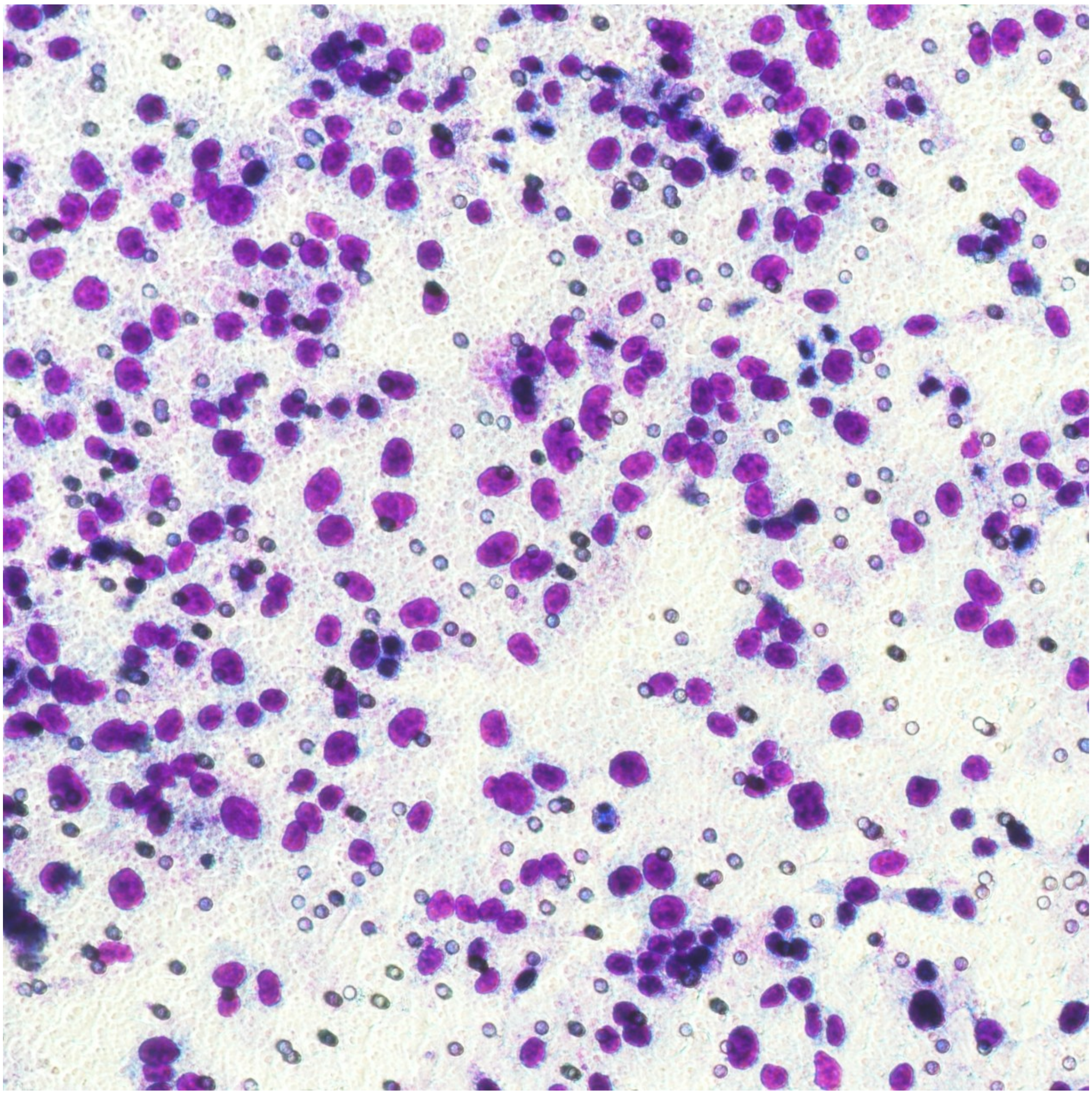

Fig.4D-Ishikawa-Migration-IGF2BP3-OE+sh-NC

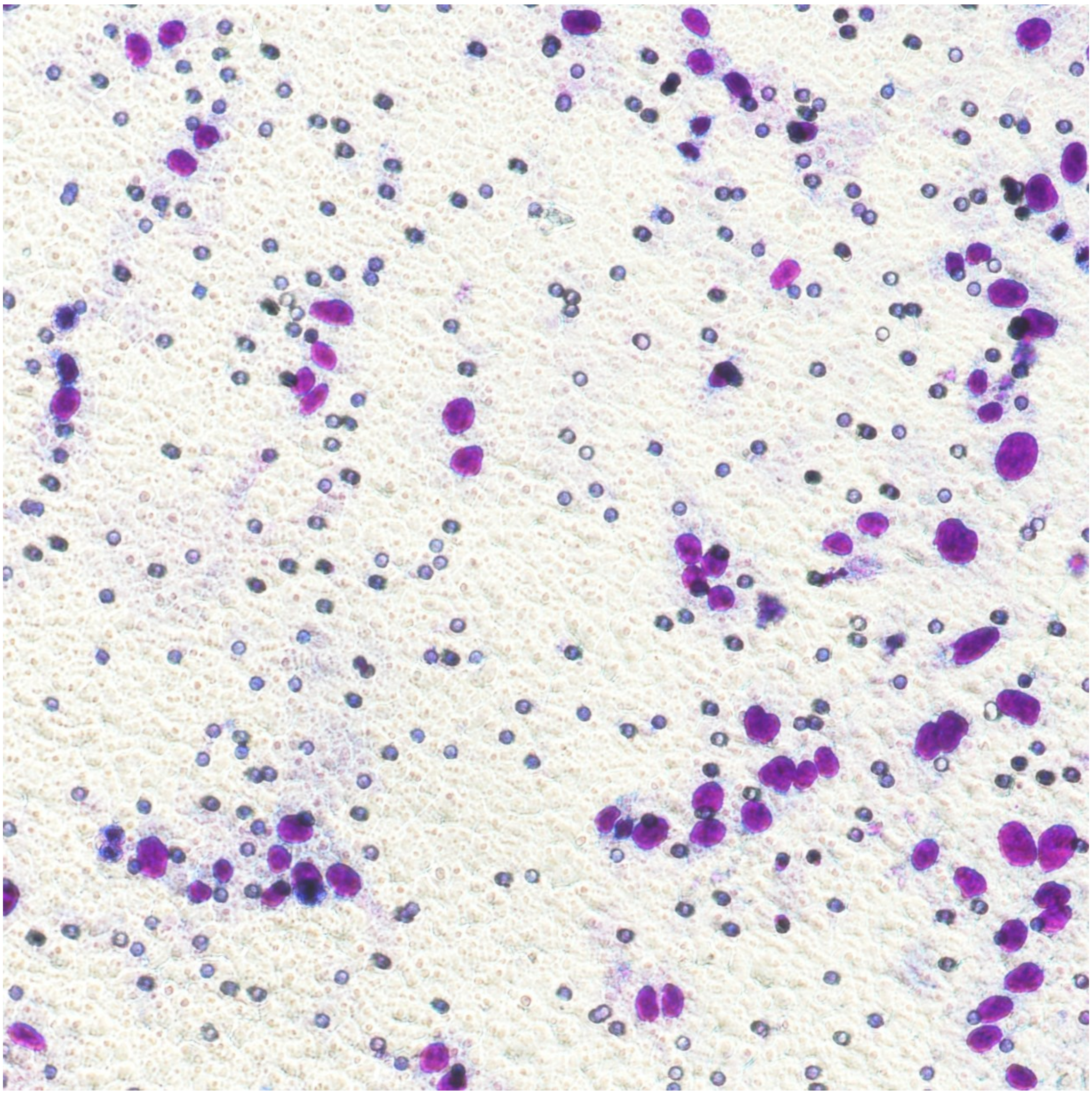

Fig.4D-Ishikawa-Migration-LV-NC+sh-LINC00958

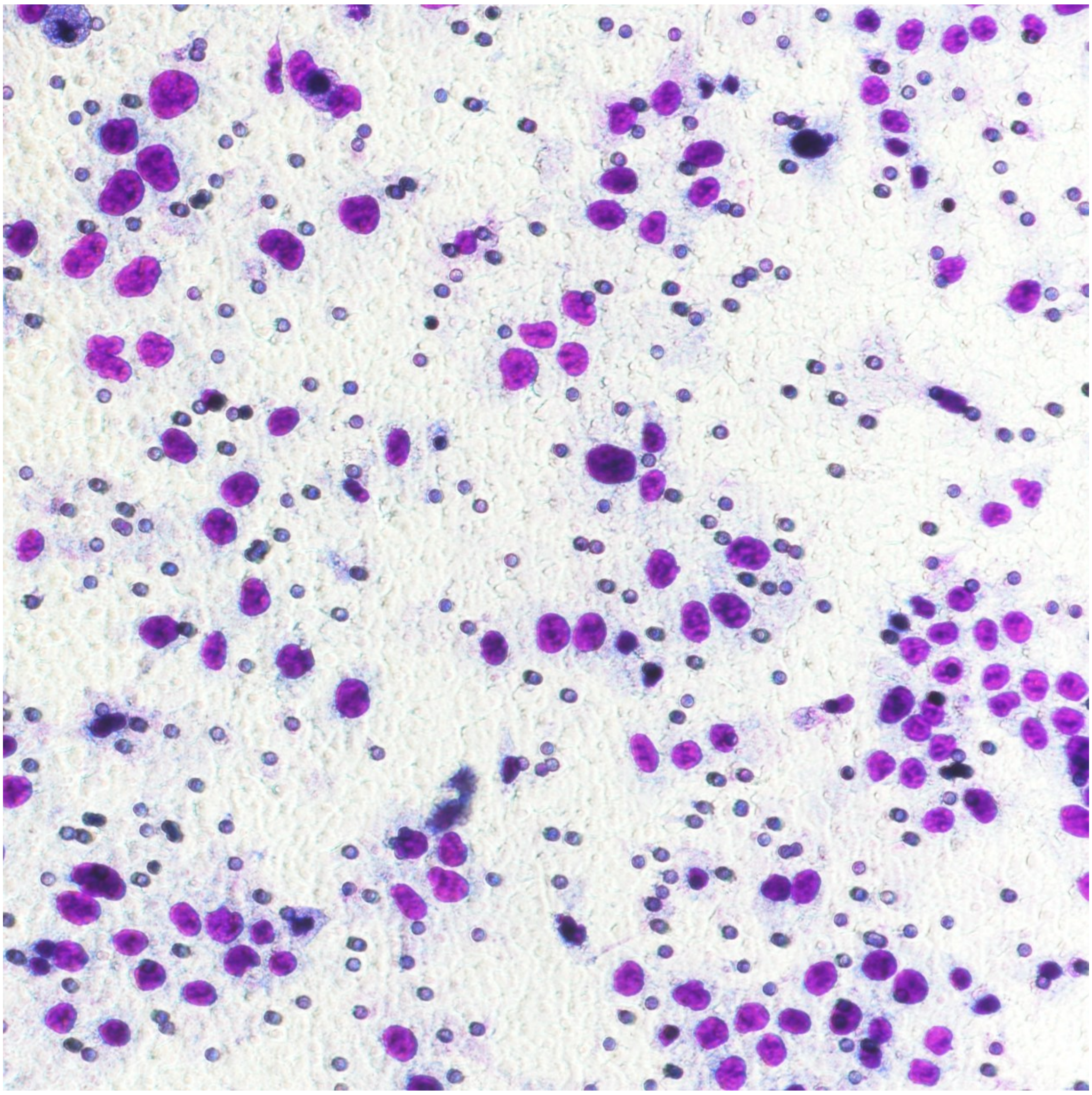

Fig.4D-Ishikawa-Migration-LV-NC+sh-NC

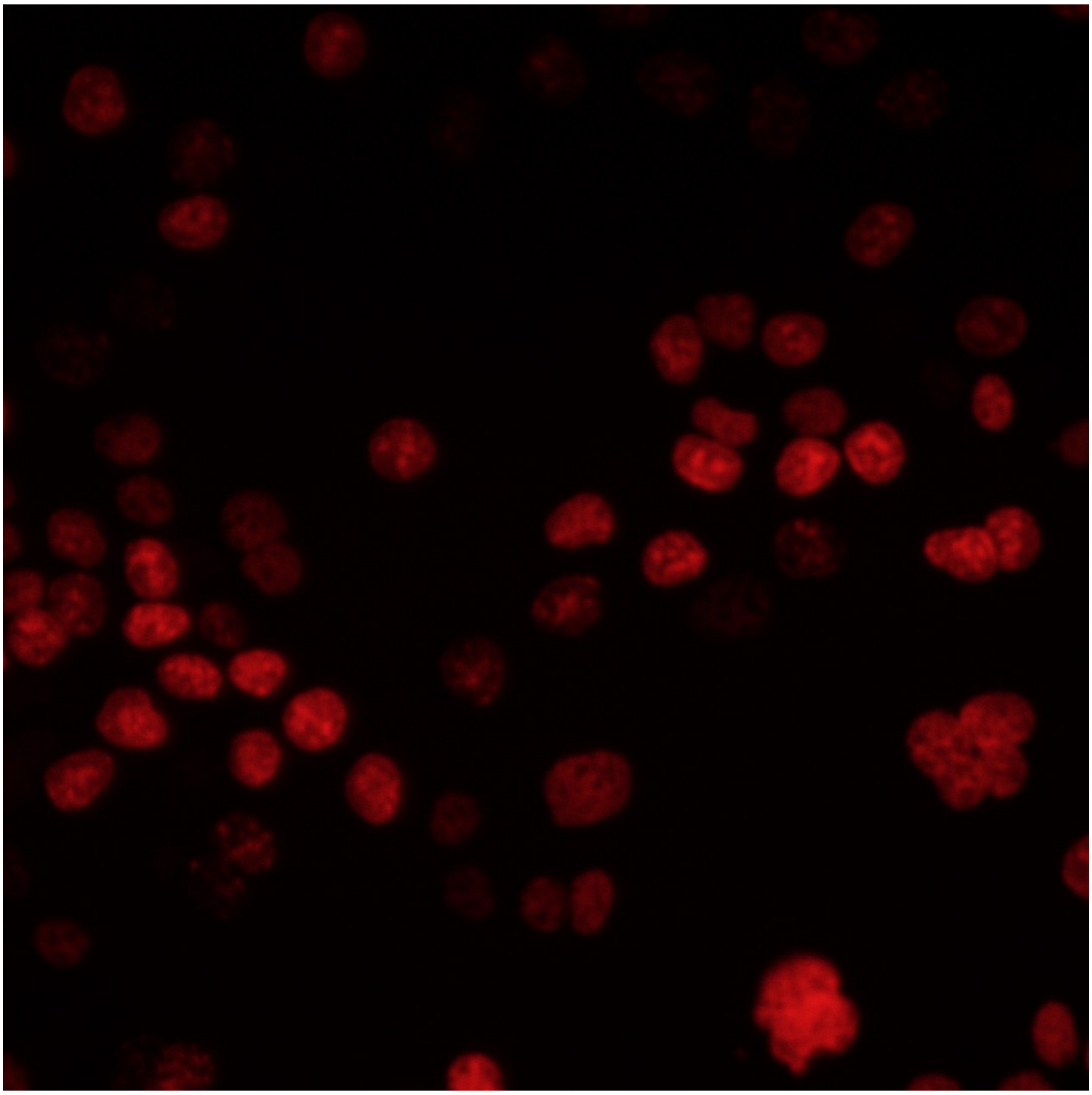

Fig.6C-Ishikawa-IGF2BP3-OE+si-E2F3-EdU

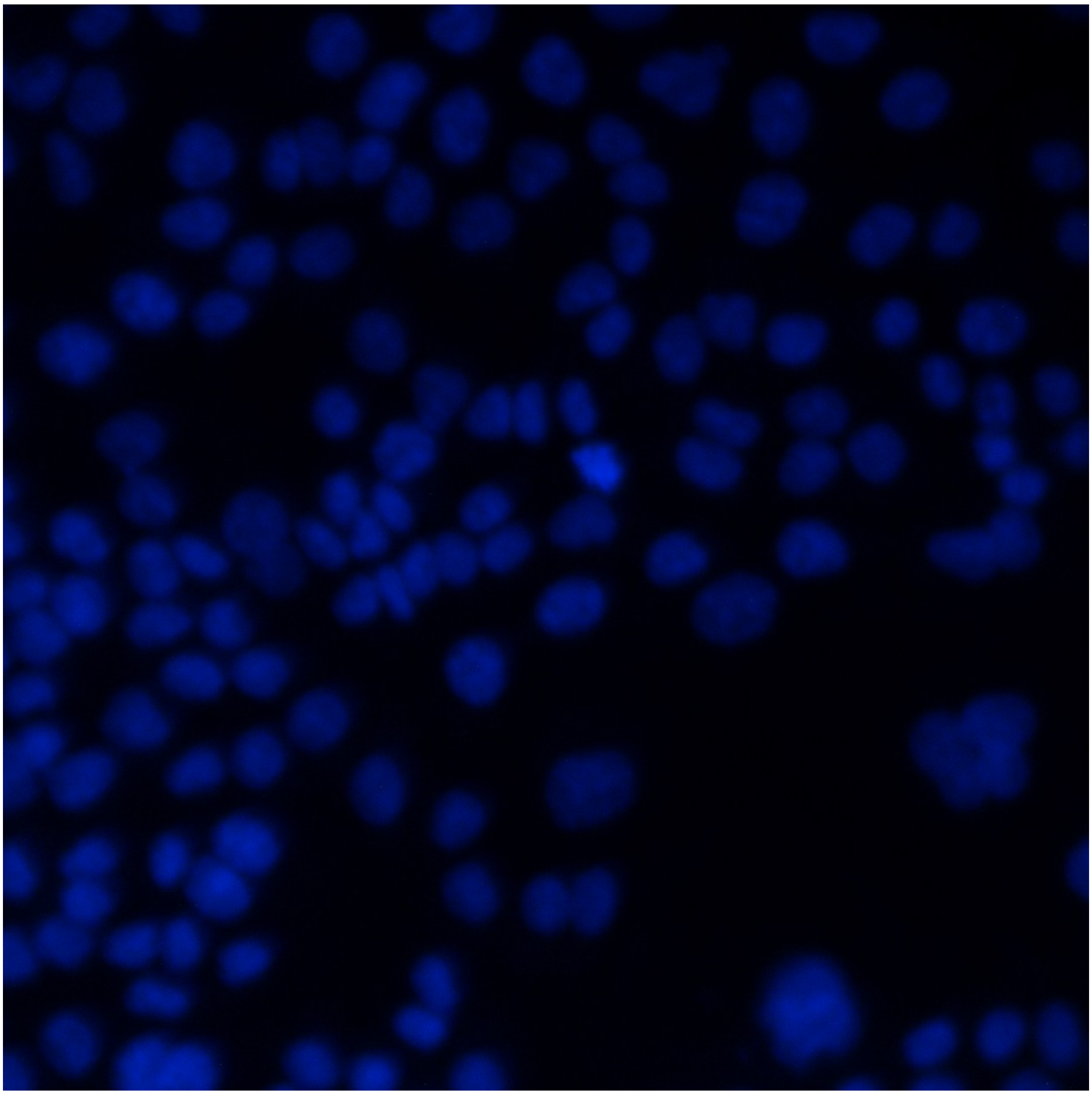

Fig.6C-Ishikawa-IGF2BP3-OE+si-E2F3-Hoechst

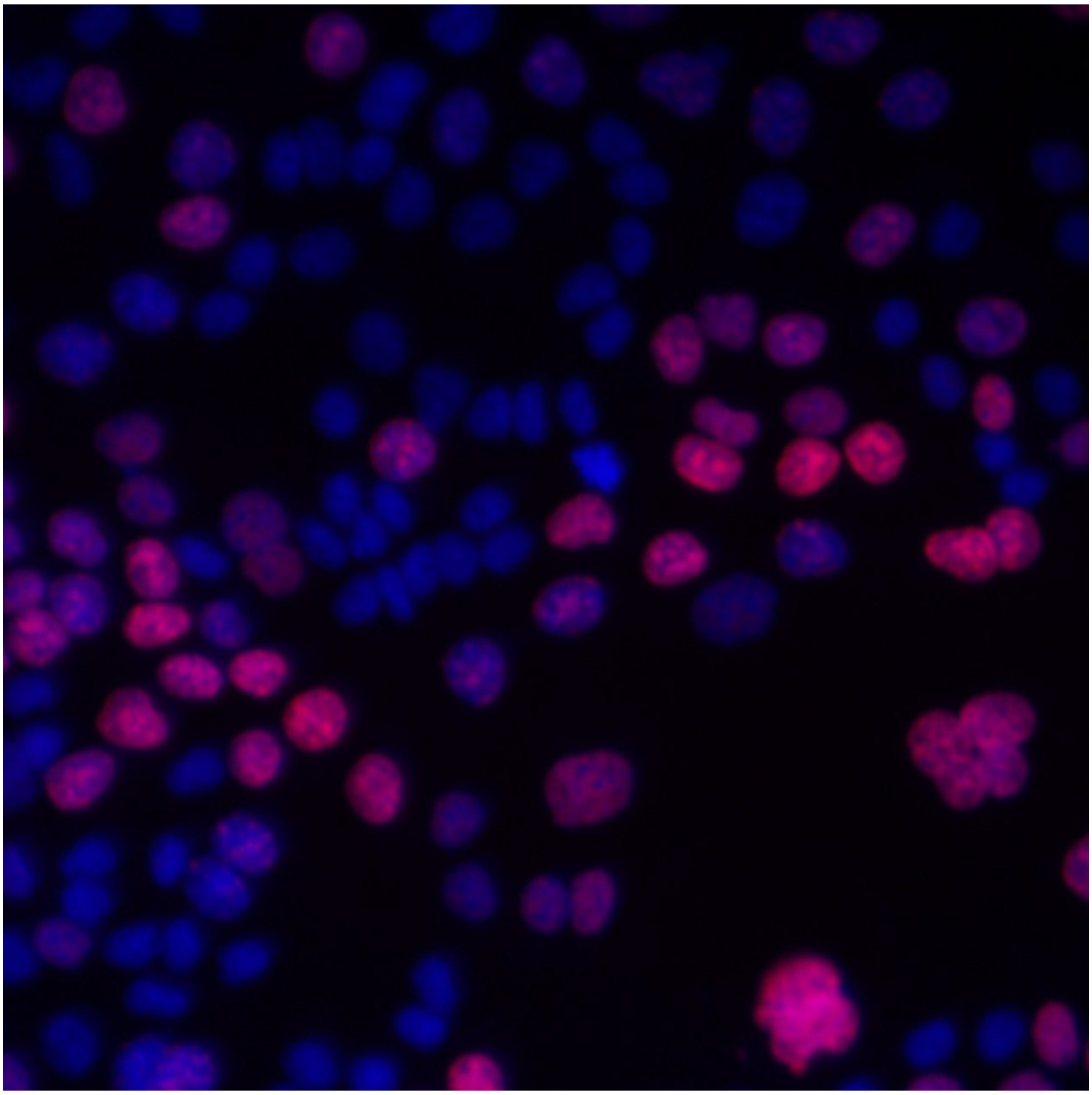

Fig.6C-Ishikawa-IGF2BP3-OE+si-E2F3-Merge

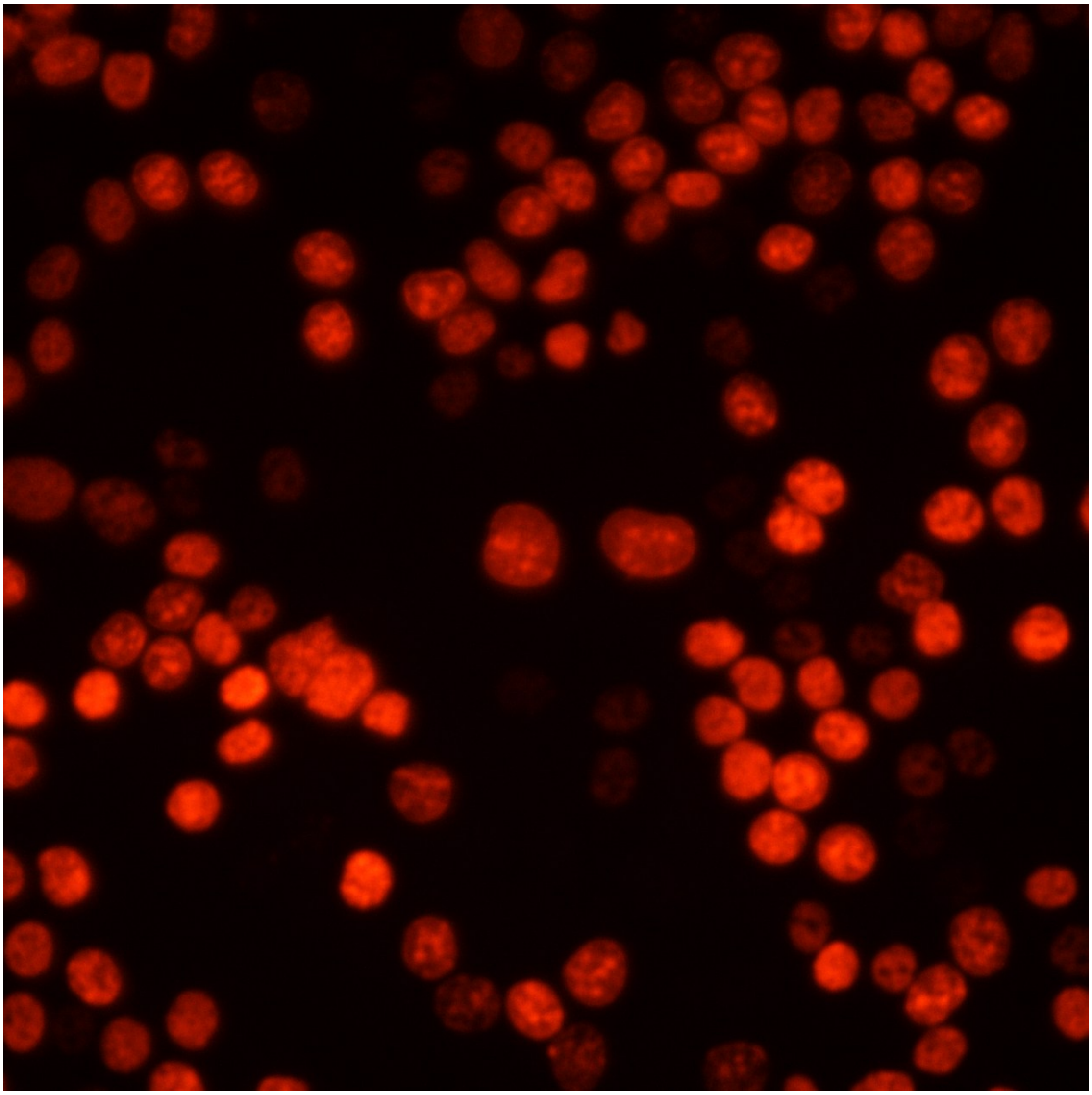

Fig.6C-Ishikawa-IGF2BP3-OE+si-NC-EdU

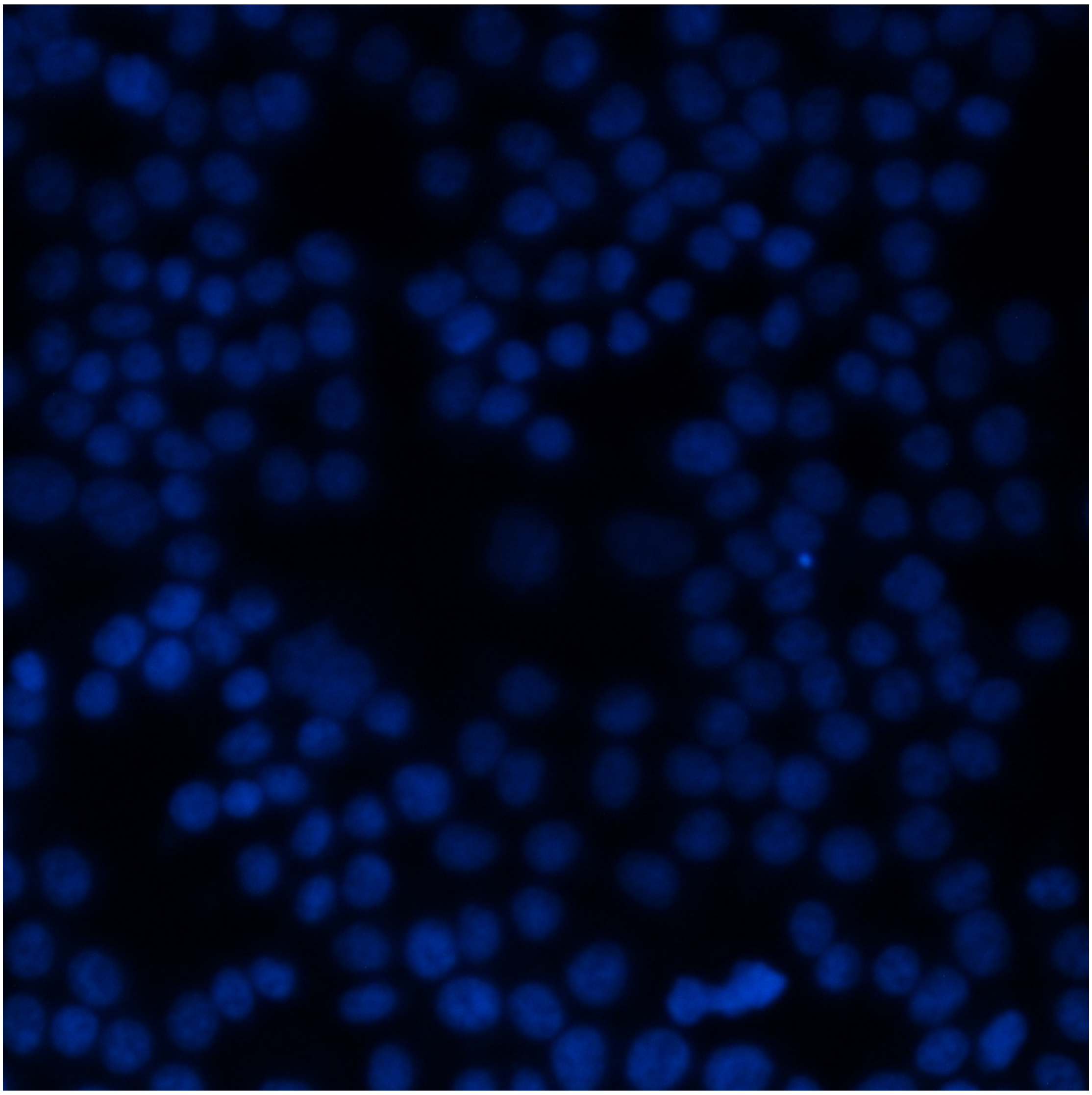

Fig.6C-Ishikawa-IGF2BP3-OE+si-NC-Hoechst

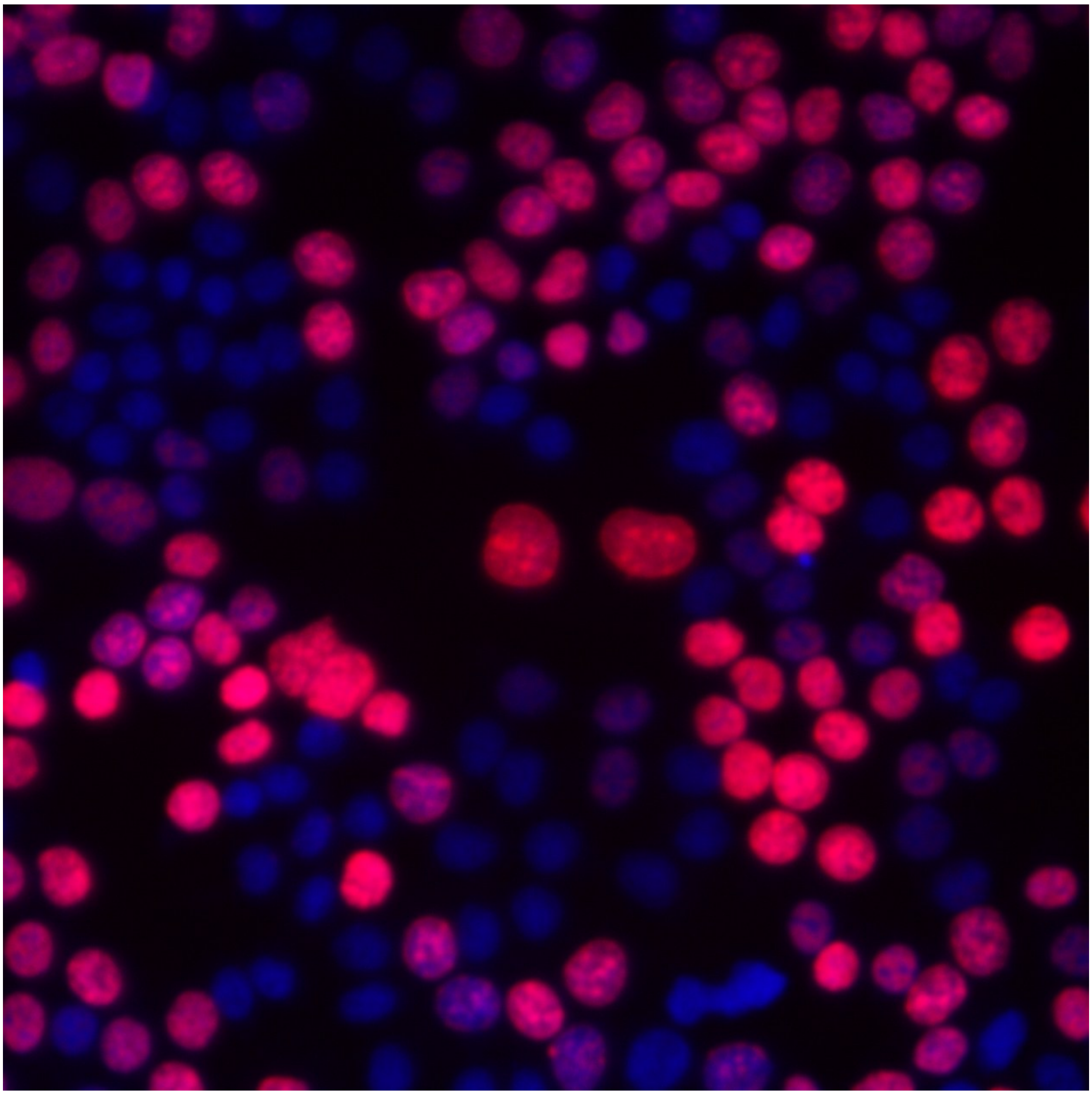

Fig.6C-Ishikawa-IGF2BP3-OE+si-NC-merge

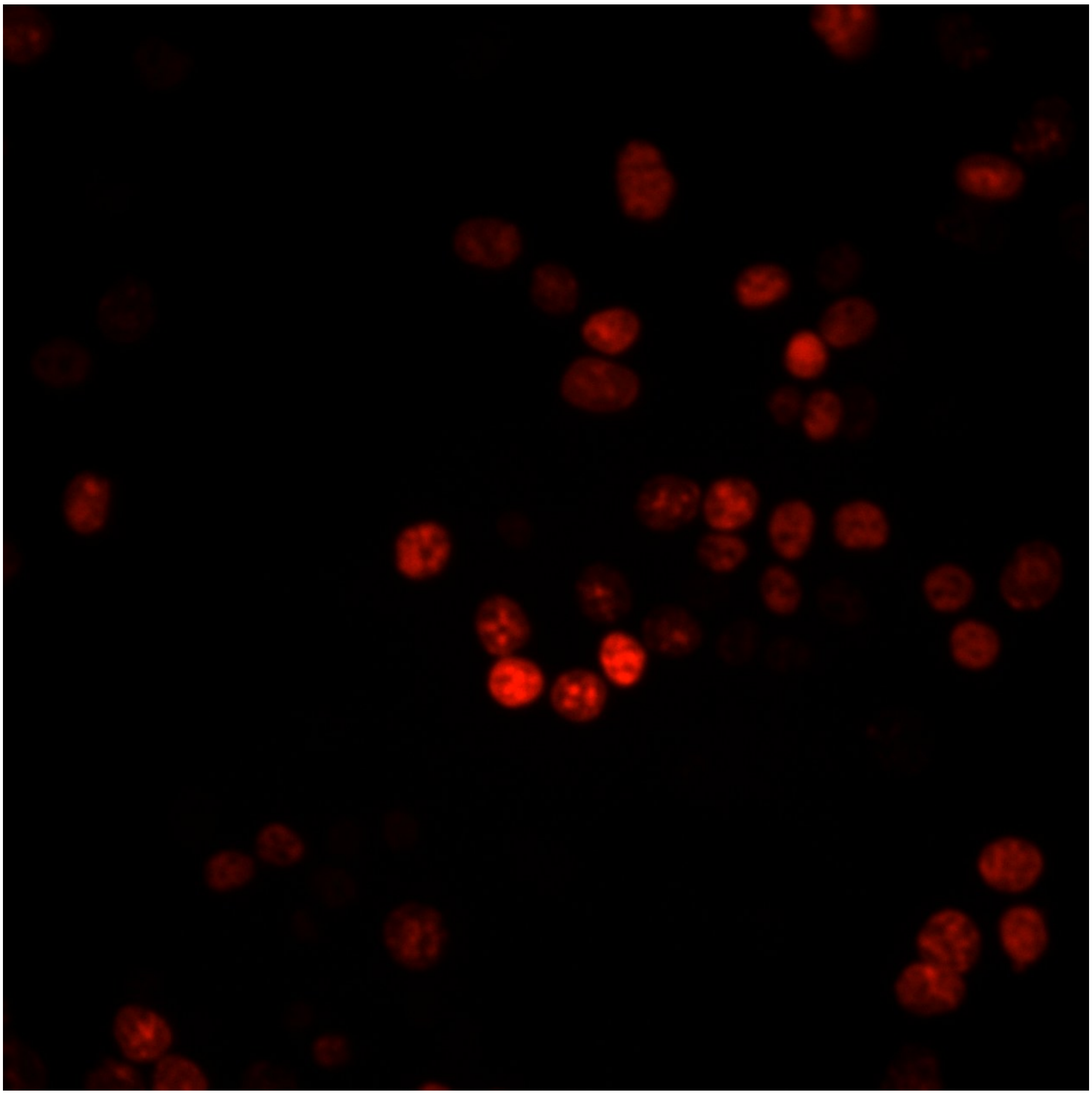

Fig.6C-Ishikawa-LV-NC+si-E2F3-EdU

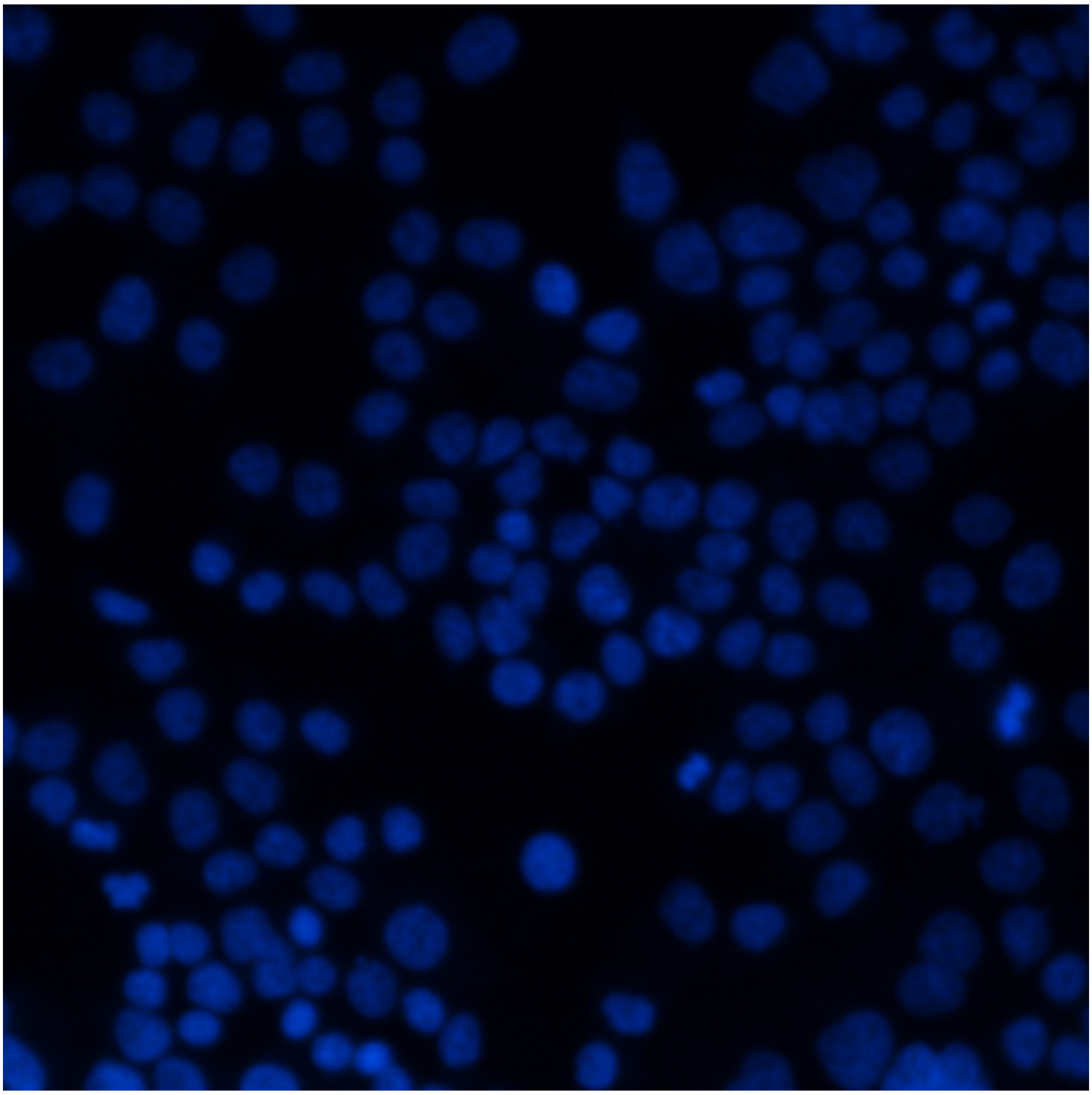

Fig.6C-Ishikawa-LV-NC+si-E2F3-Hoechst

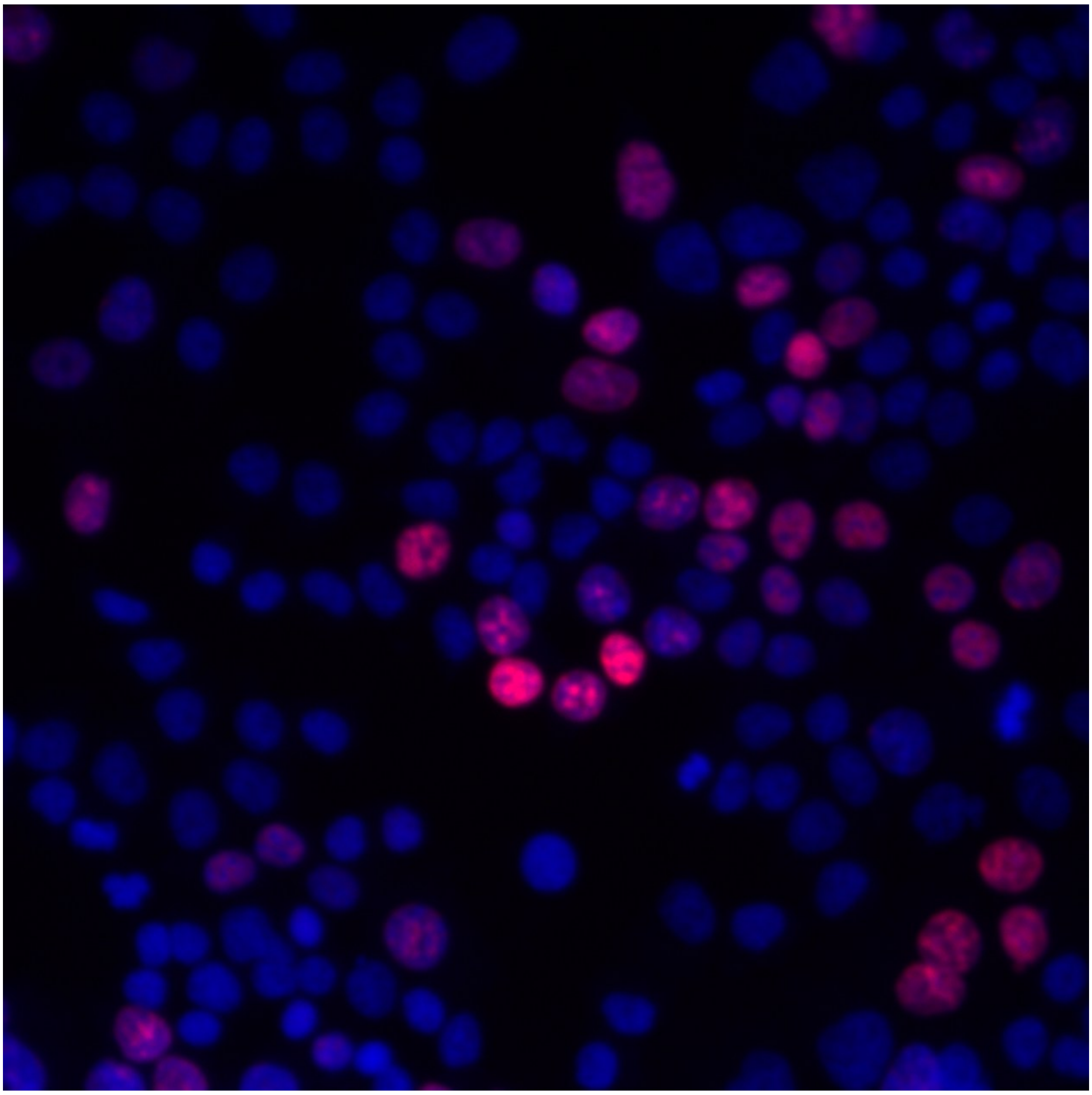

Fig.6C-Ishikawa-LV-NC+si-E2F3-Merge

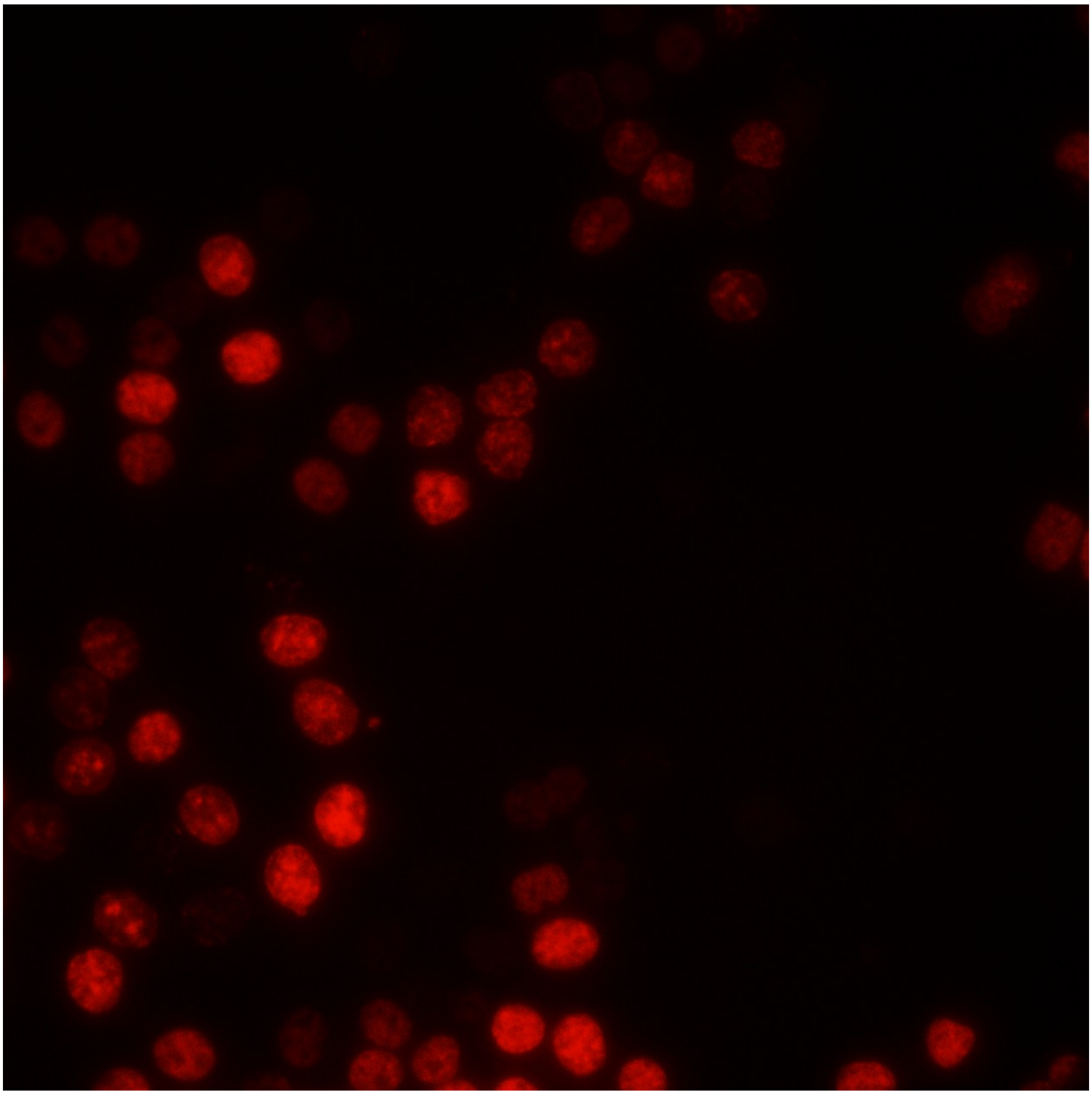

Fig.6C-Ishikawa-LV-NC+si-NC-EdU

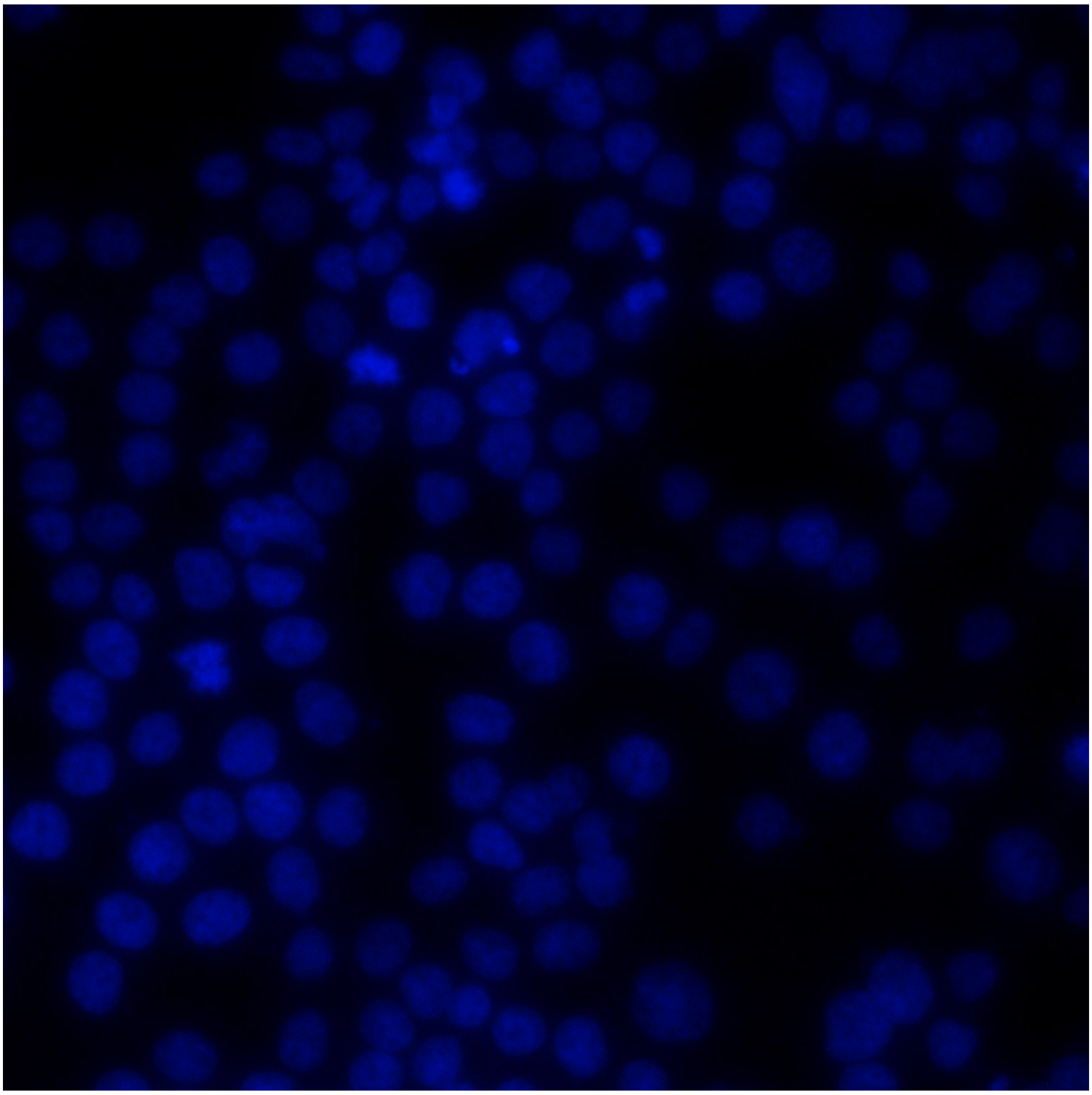

Fig.6C-Ishikawa-LV-NC+si-NC-Hoechst

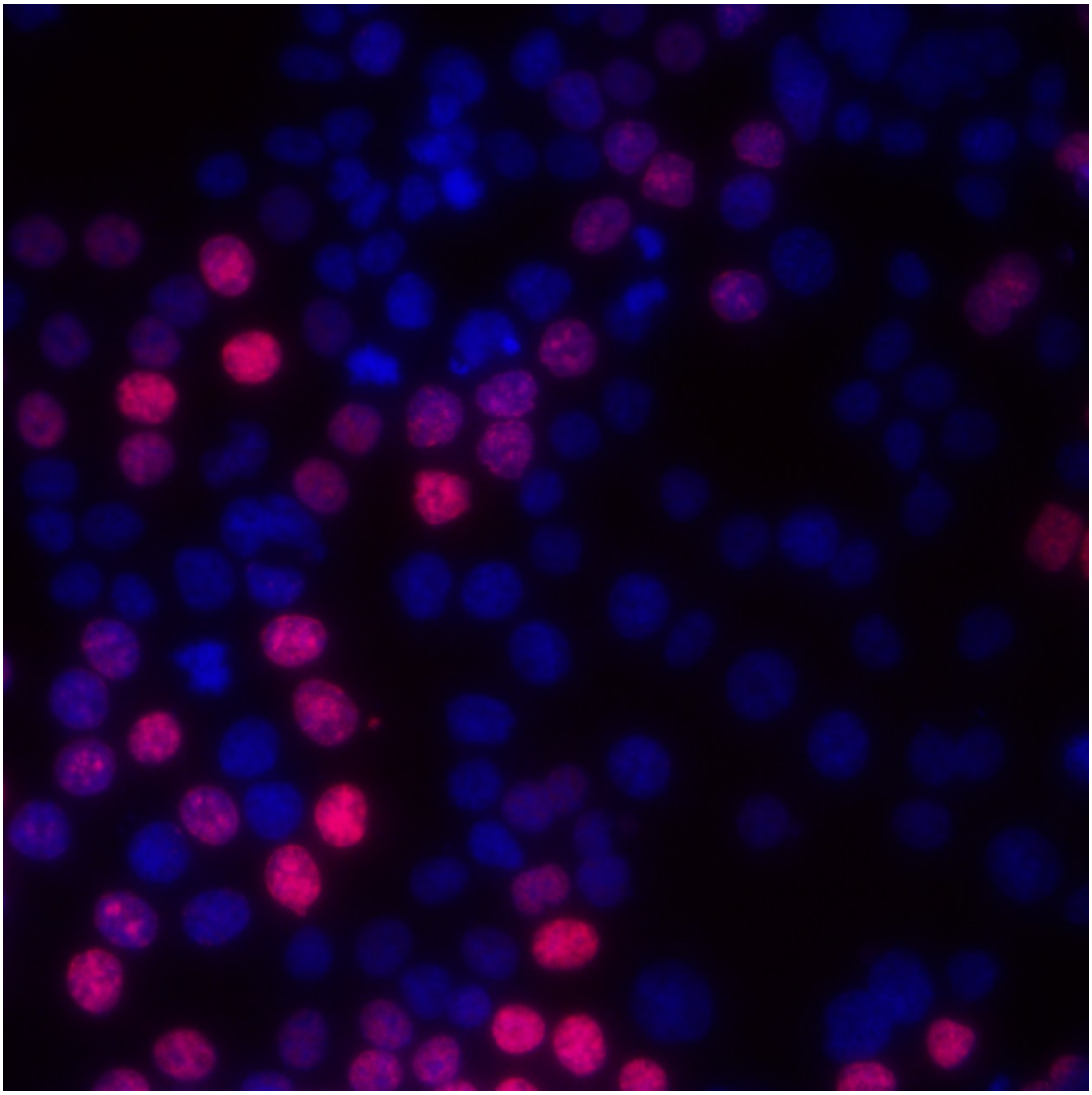

Fig.6C-Ishikawa-LV-NC+si-NC-Merge

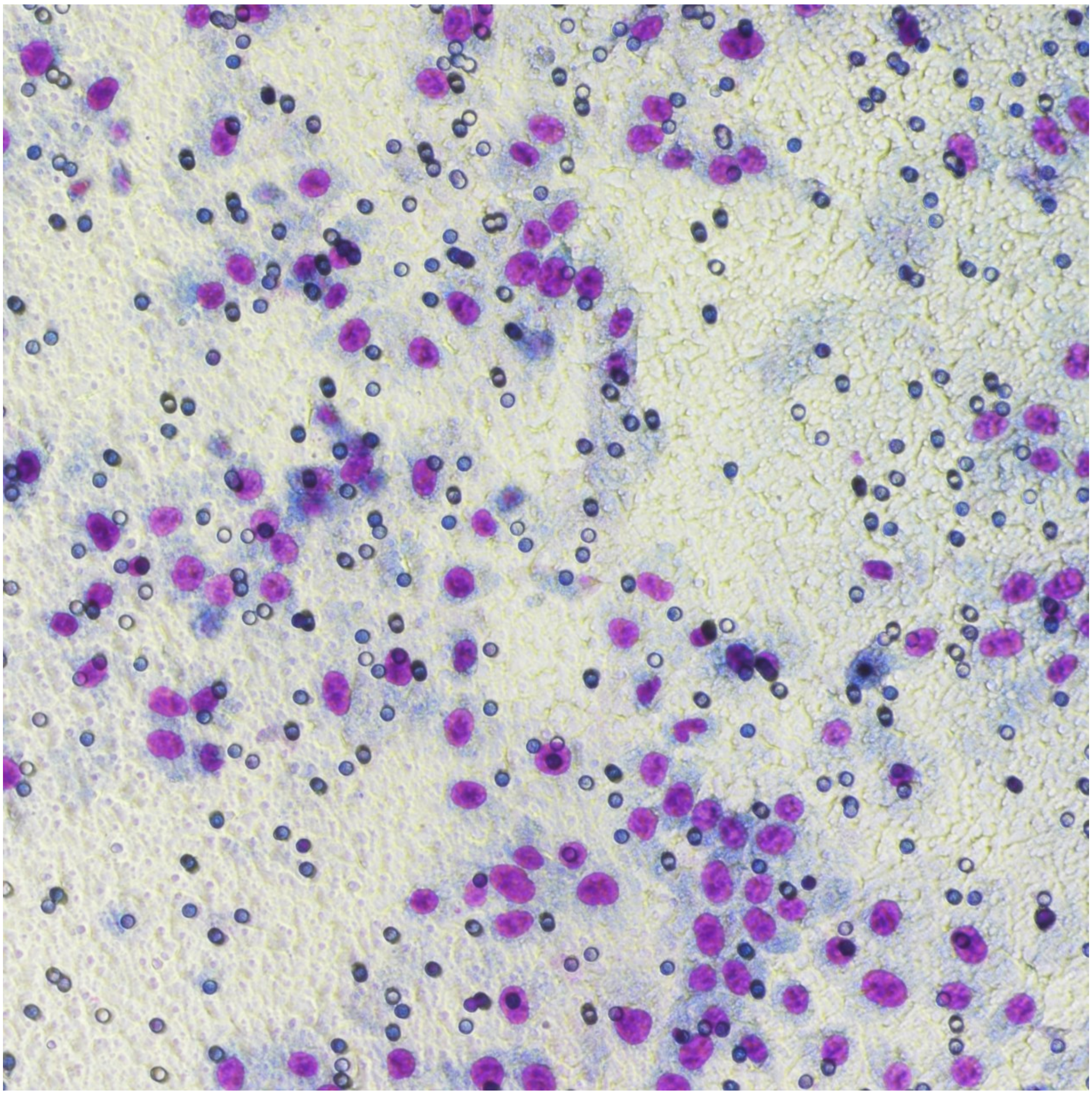

Fig.6D-Ishikawa-invasion-IGF2BP3-OE+si-E2F3

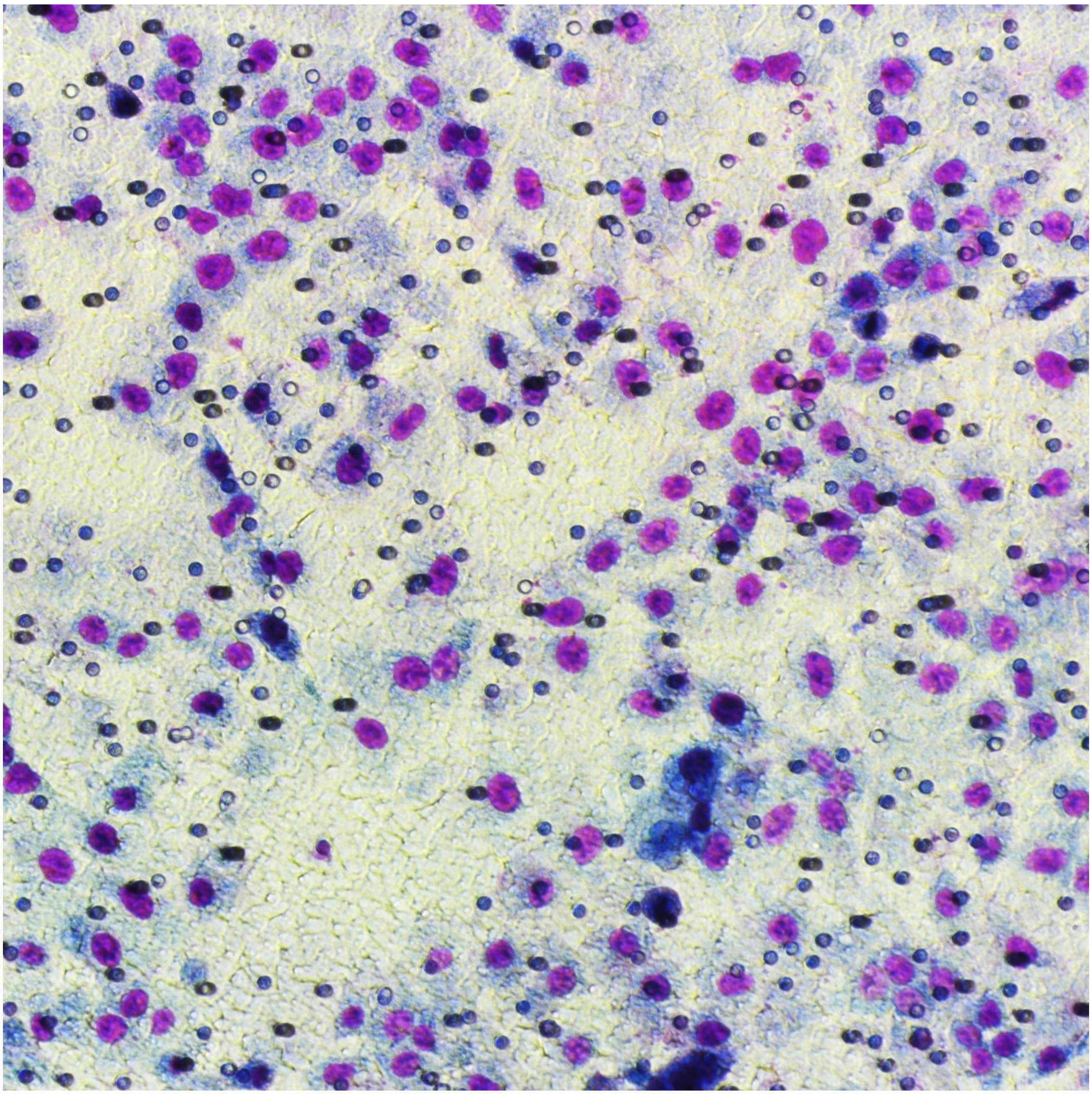

Fig.6D-Ishikawa-invasion-IGF2BP3-OE+si-NC

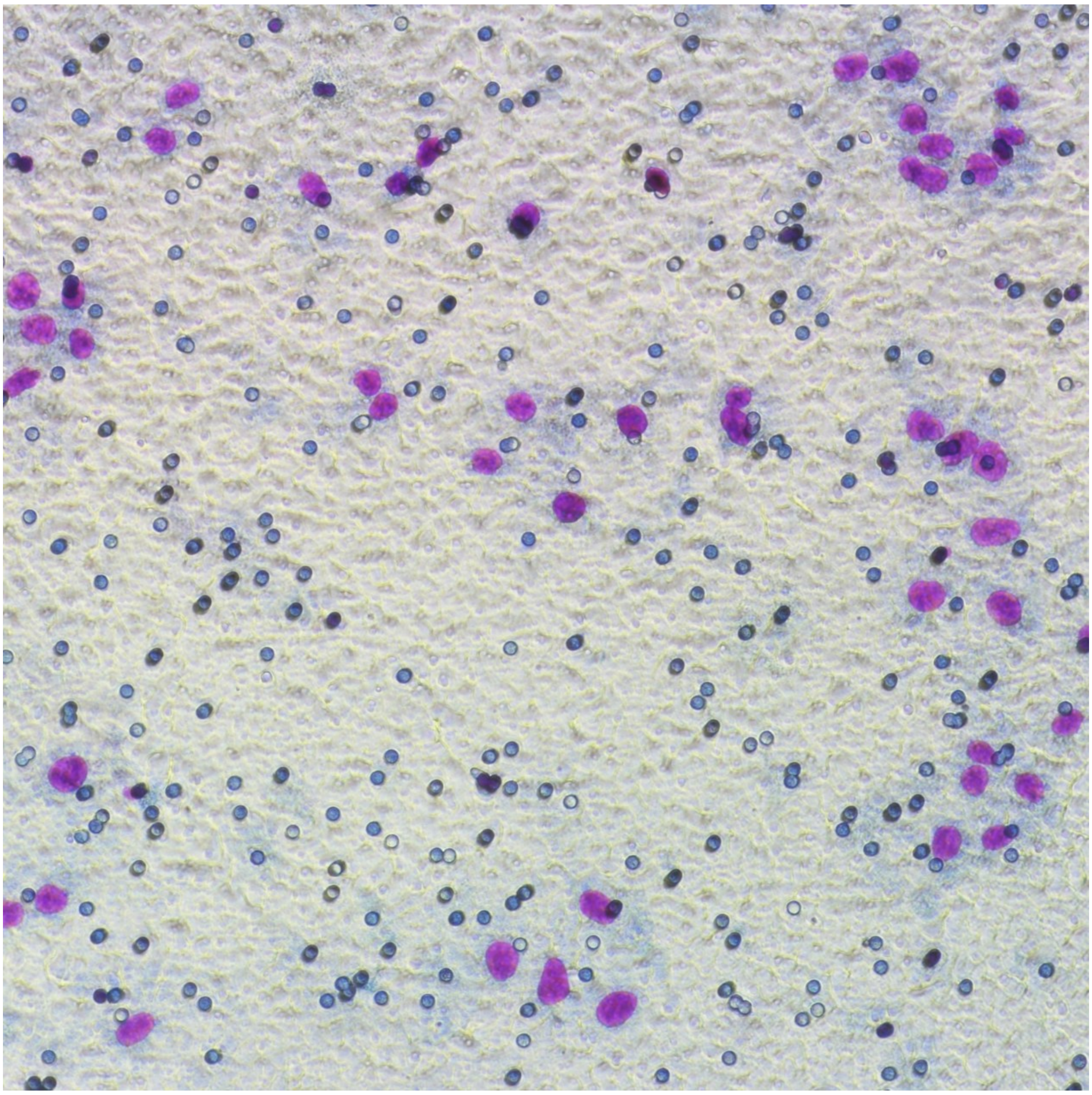

Fig.6D-Ishikawa-invasion-LV-NC+si-E2F3

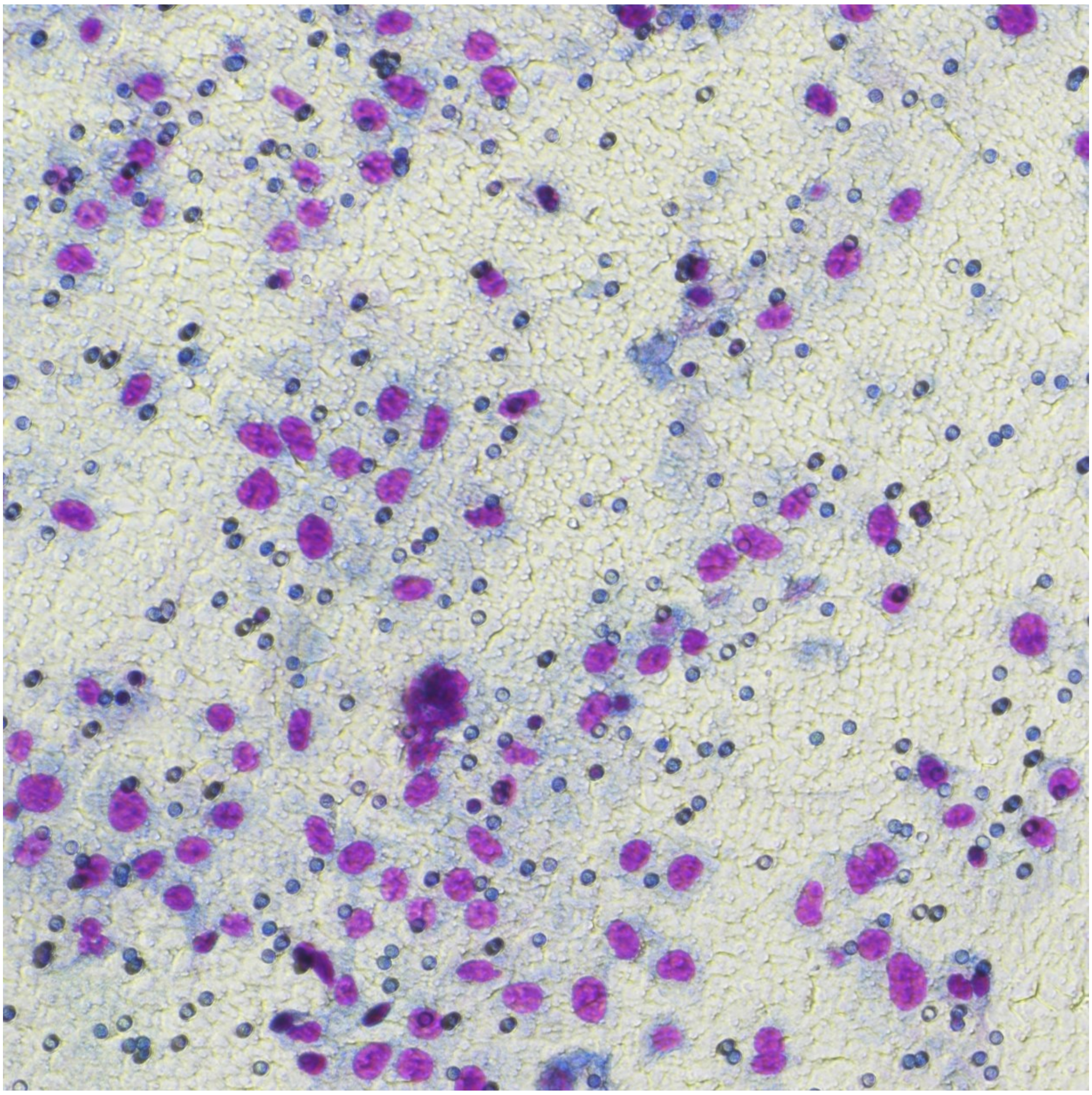

Fig.6D-Ishikawa-invasion-LV-NC+si-NC

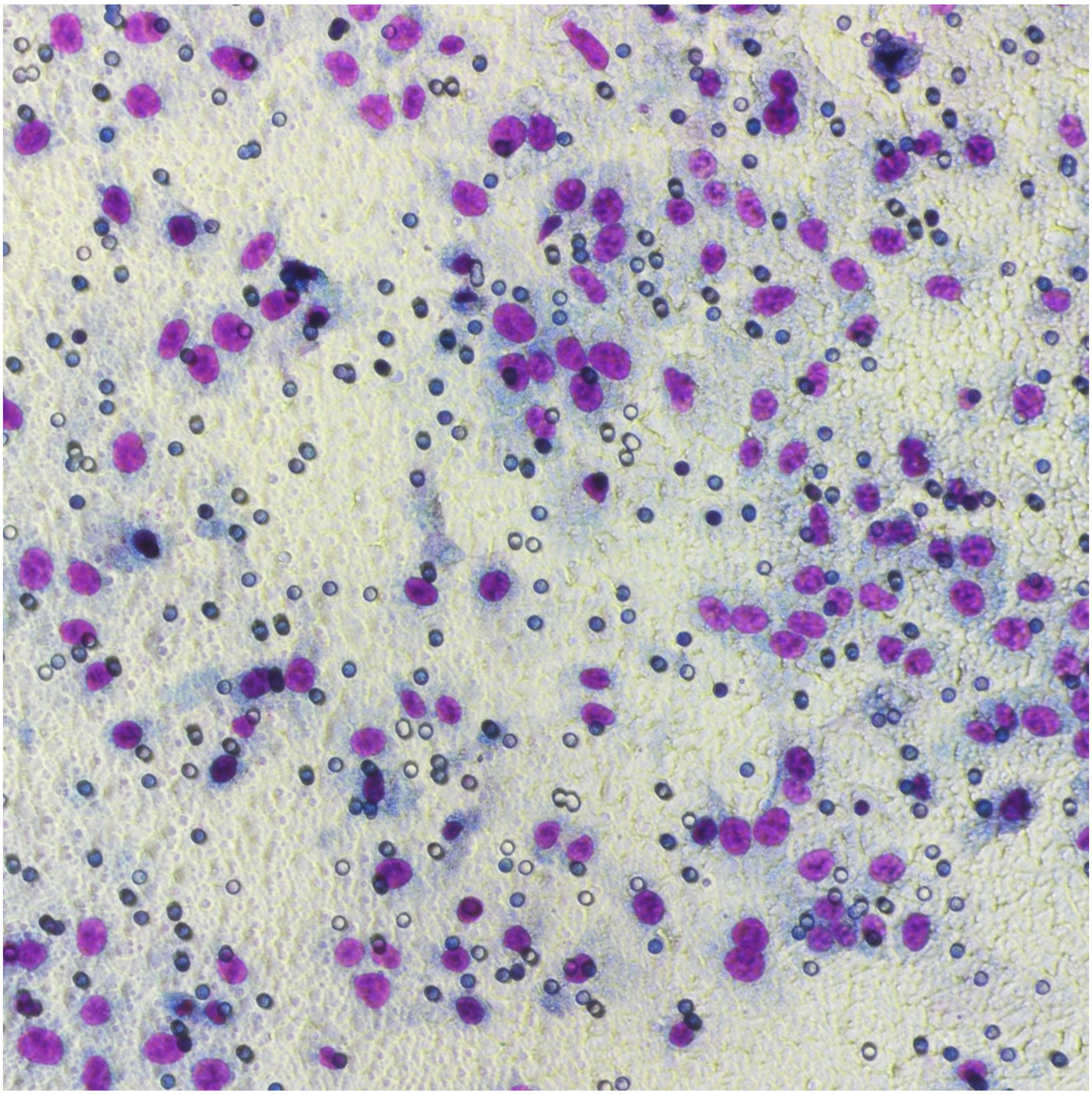

Fig.6D-Ishikawa-migration-IGF2BP3-OE+si-E2F3

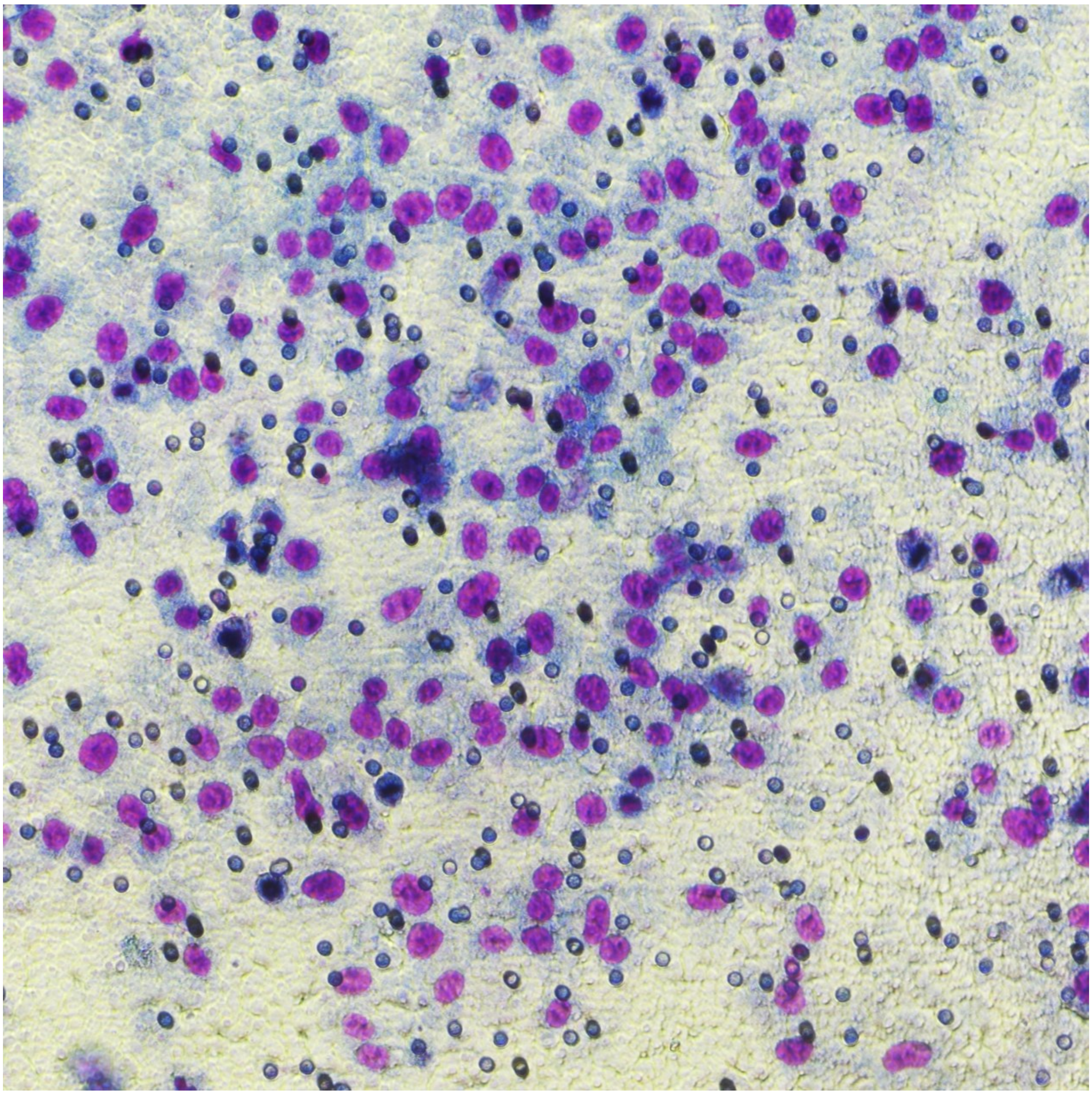

Fig.6D-Ishikawa-migration-IGF2BP3-OE+si-NC

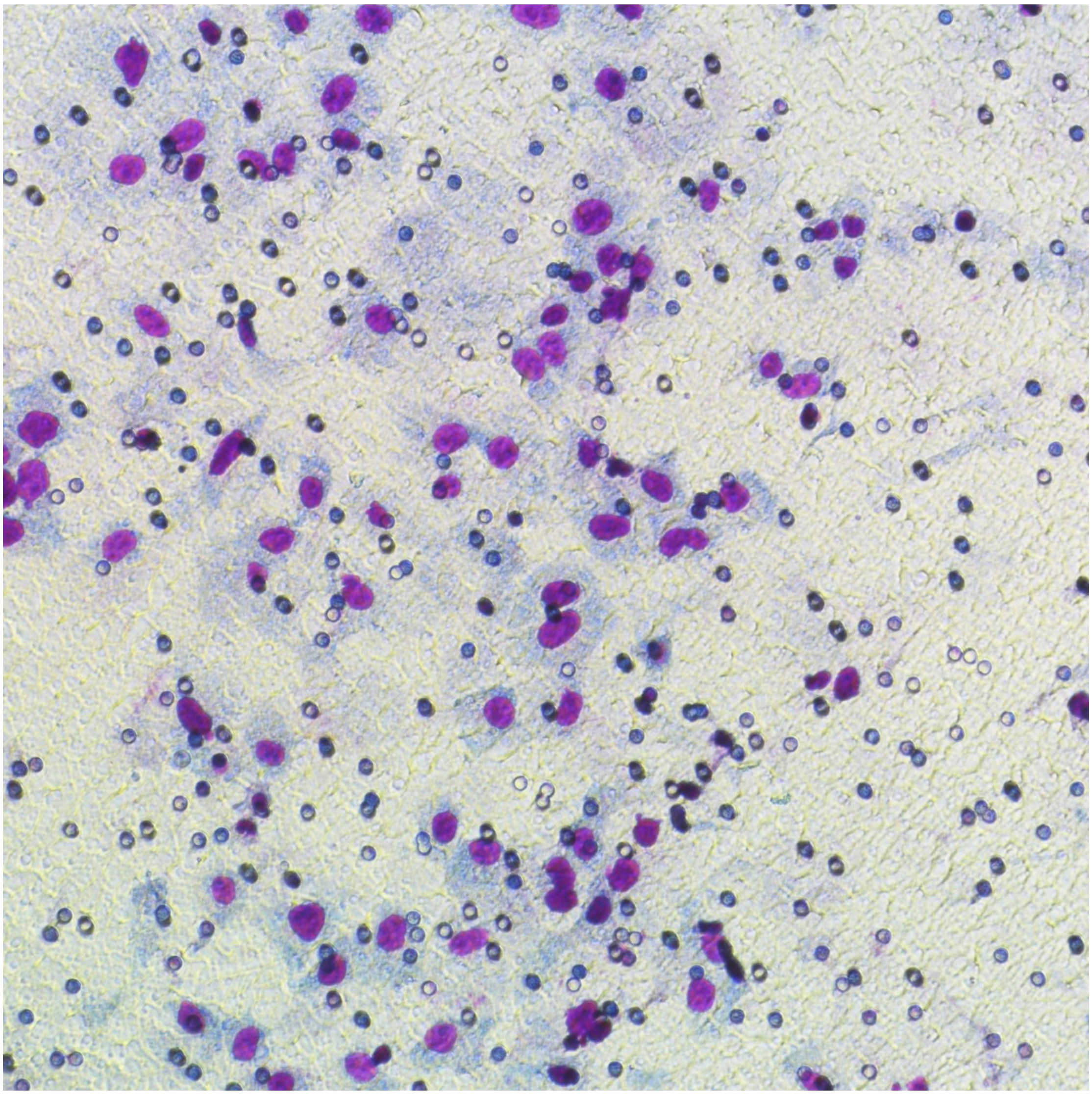

Fig.6D-Ishikawa-migration-LV-NC+si-E2F3

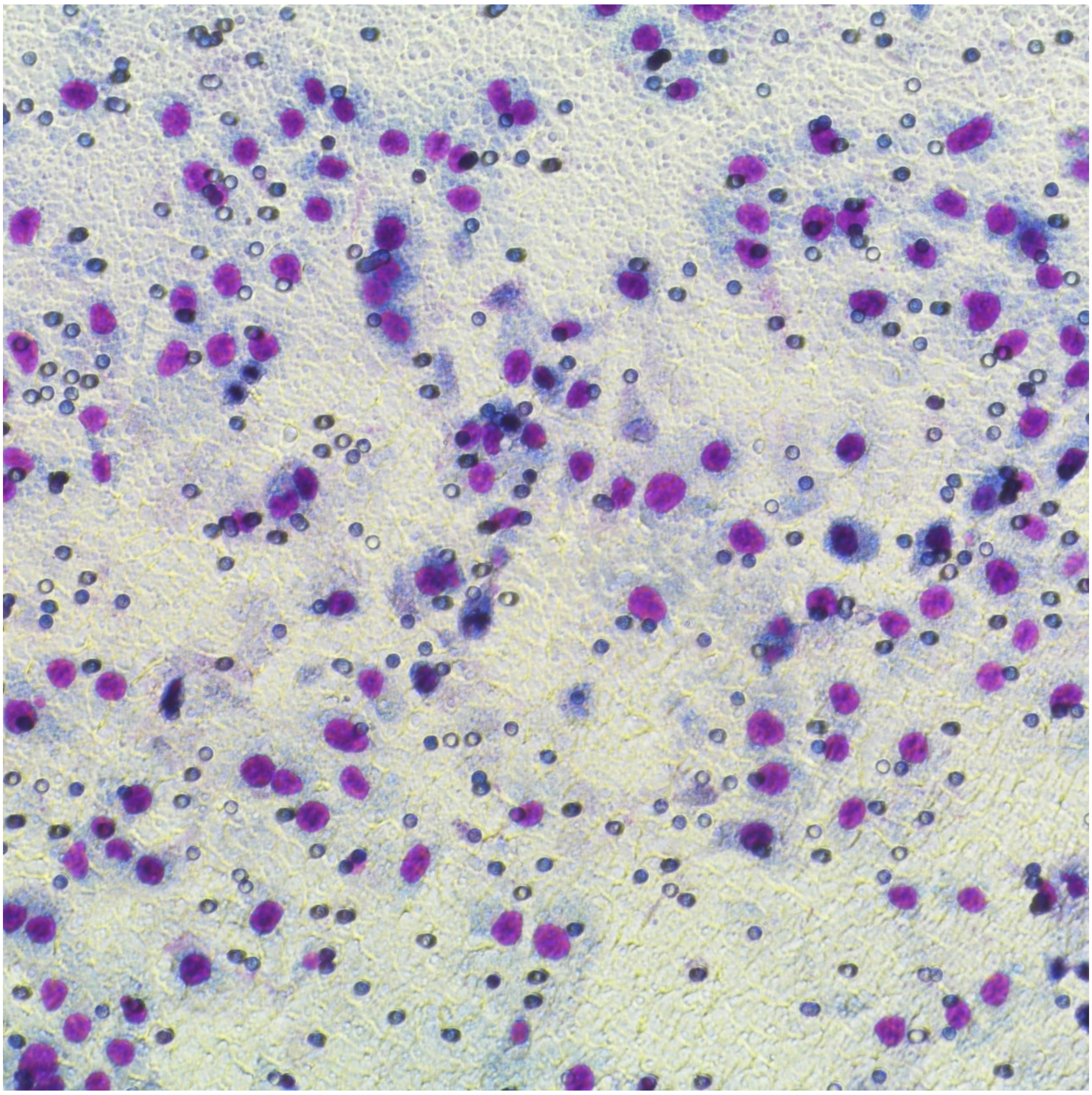

Fig.6D-Ishikawa-migration-LV-NC+si-NC

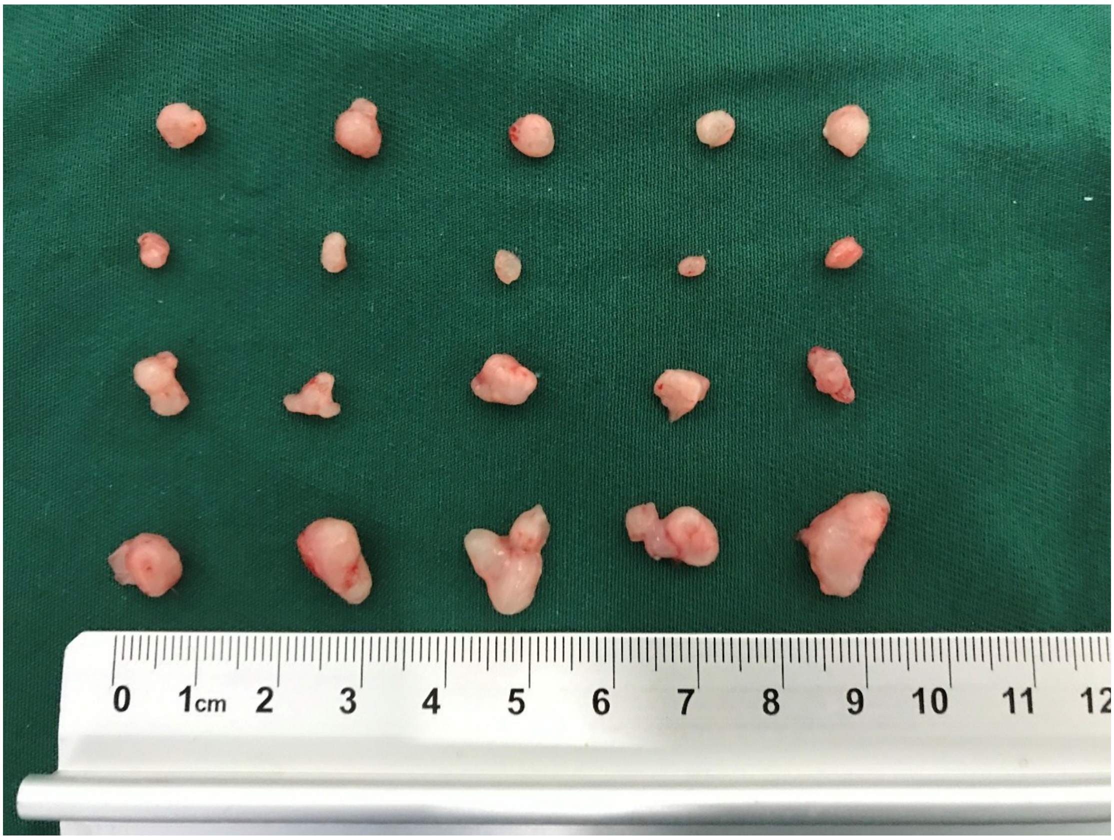

Fig.7A

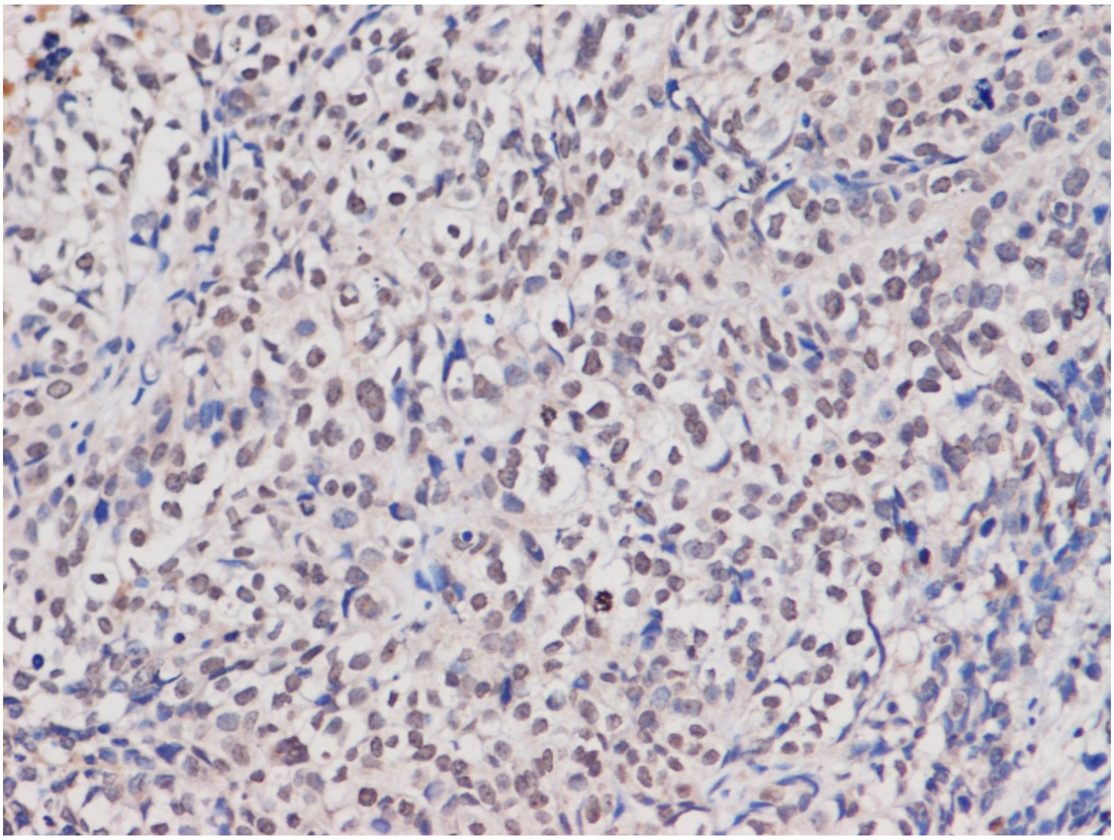

Fig.7B-E2F3-IGF2BP3-OE

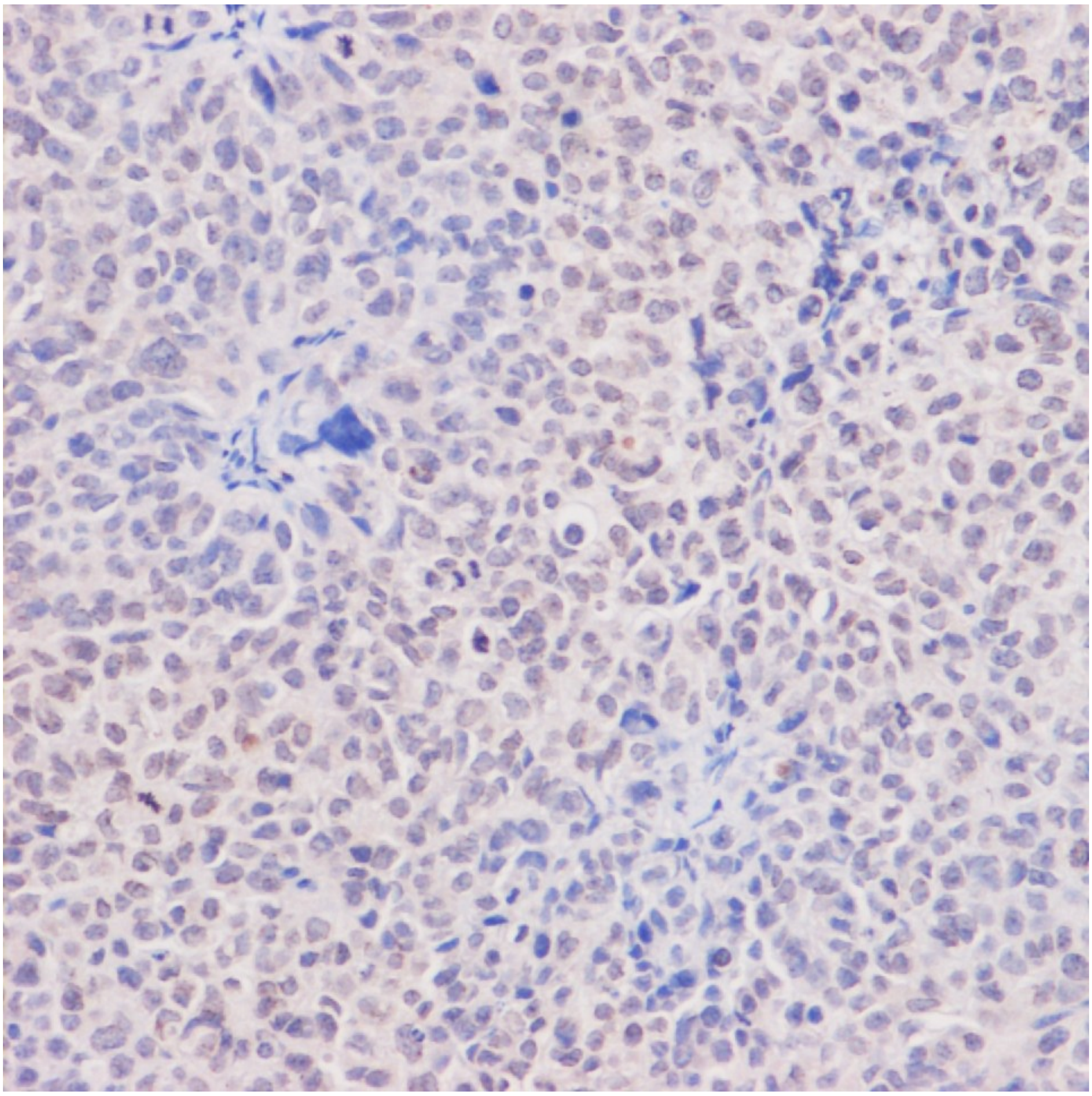

Fig.7B-E2F3-LV-NC

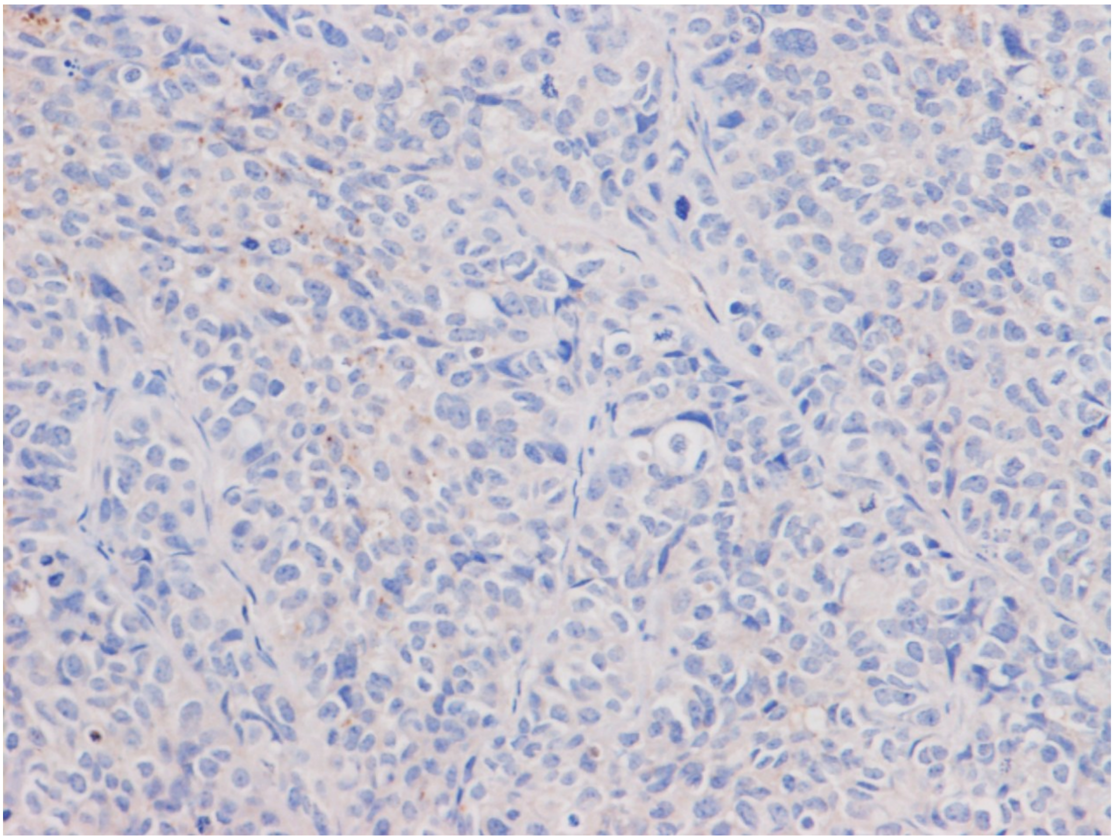

Fig.7B-E2F3-sh-IGF2BP3

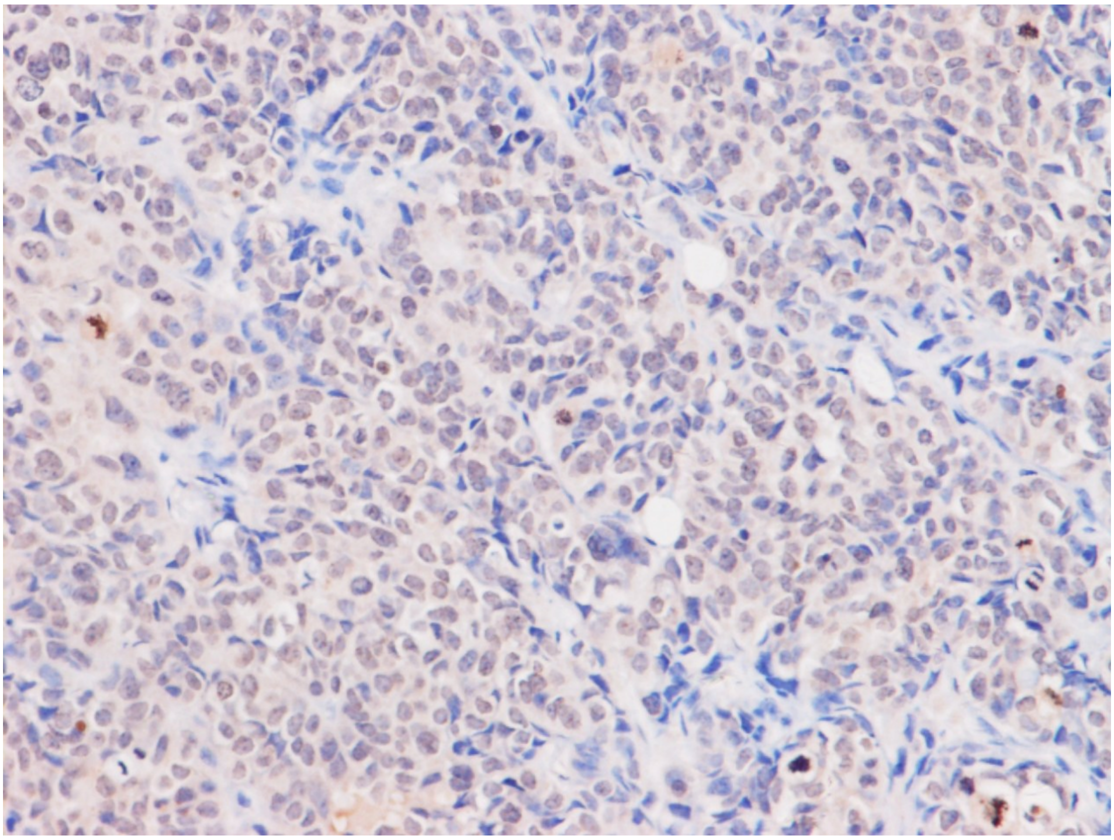

Fig.7B-E2F3-sh-NC

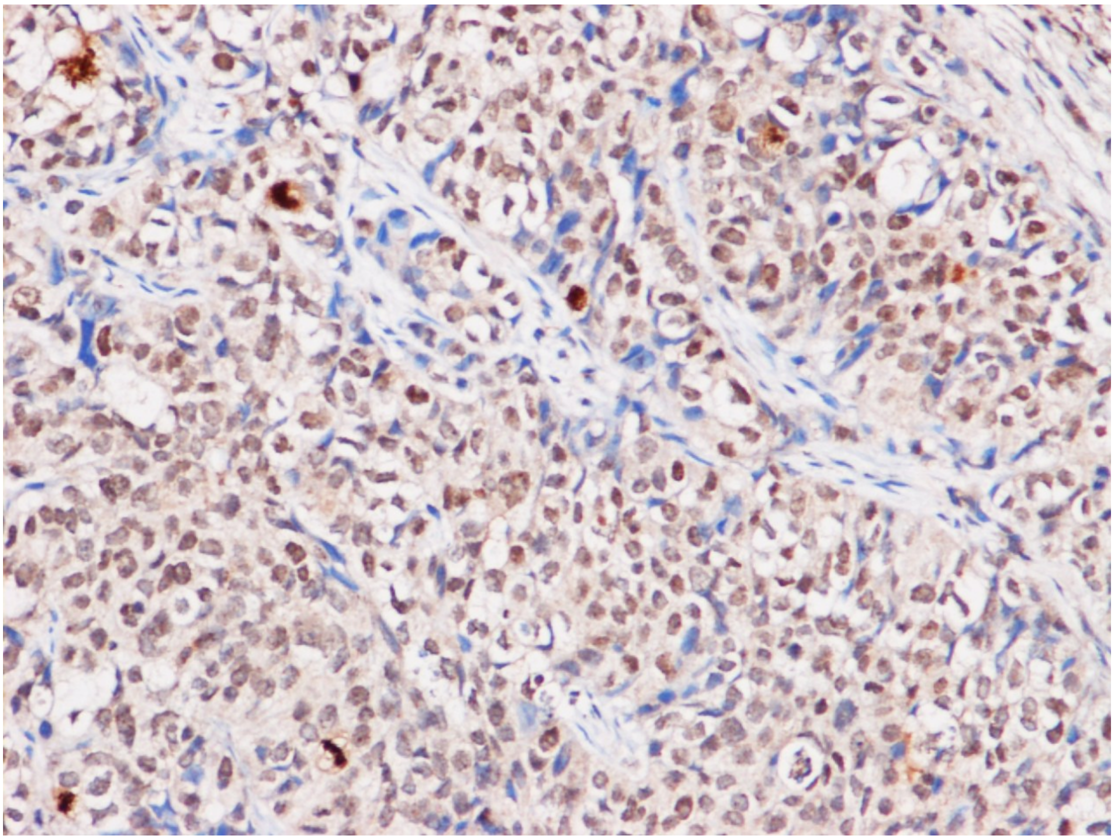

Fig.7B-Ki67-IGF2BP3-OE

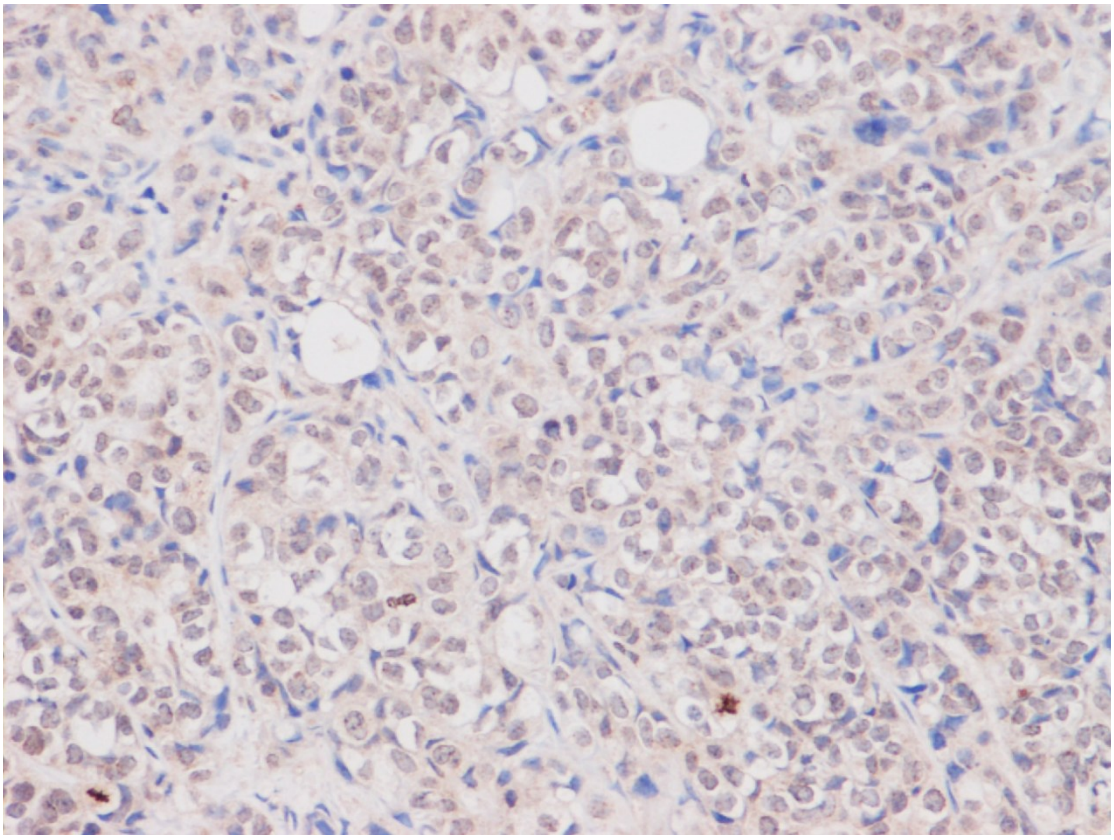

Fig.7B-Ki67-LV-NC

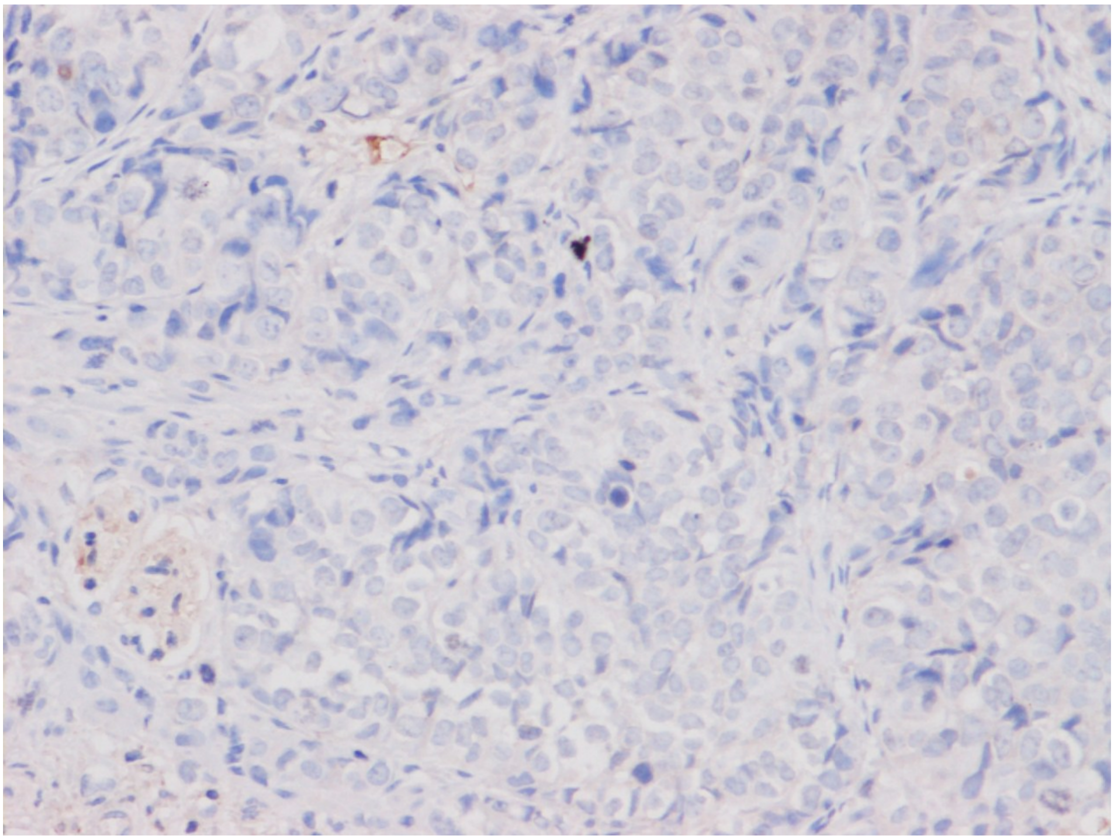

Fig.7B-Ki67-sh-IGF2BP3

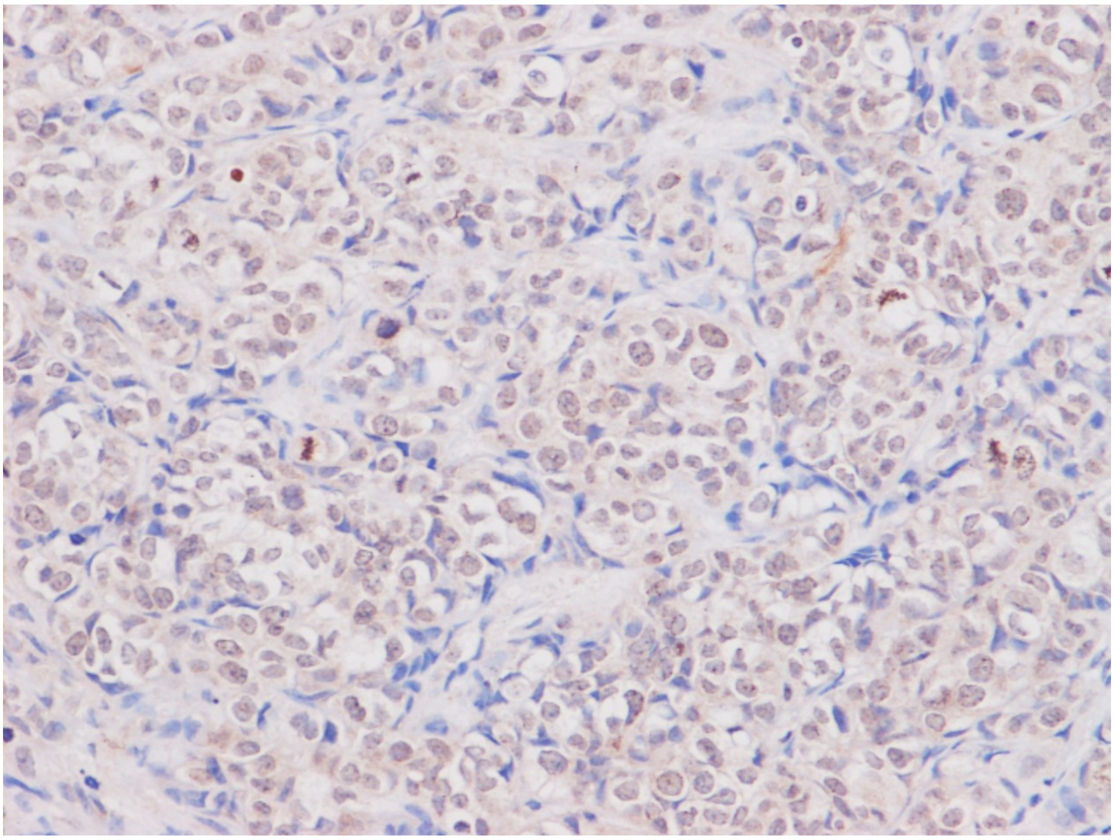

Fig.7B-Ki67-sh-NC

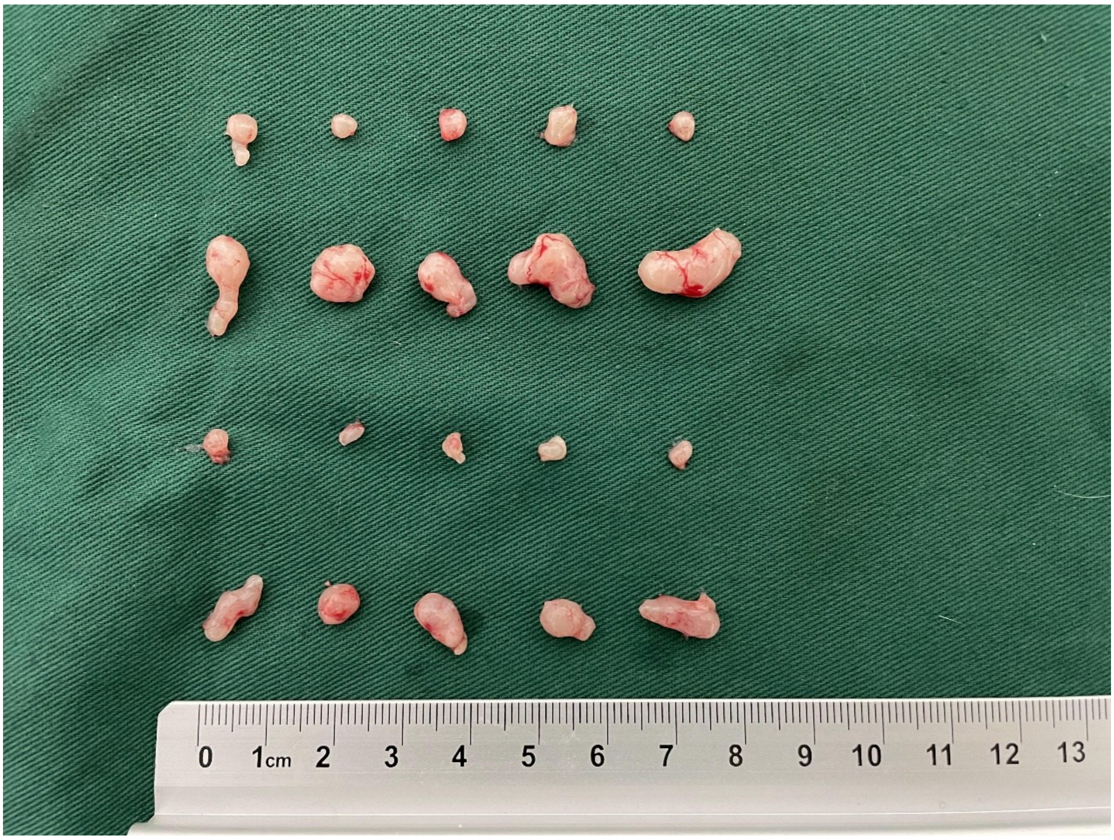

Fig.7C

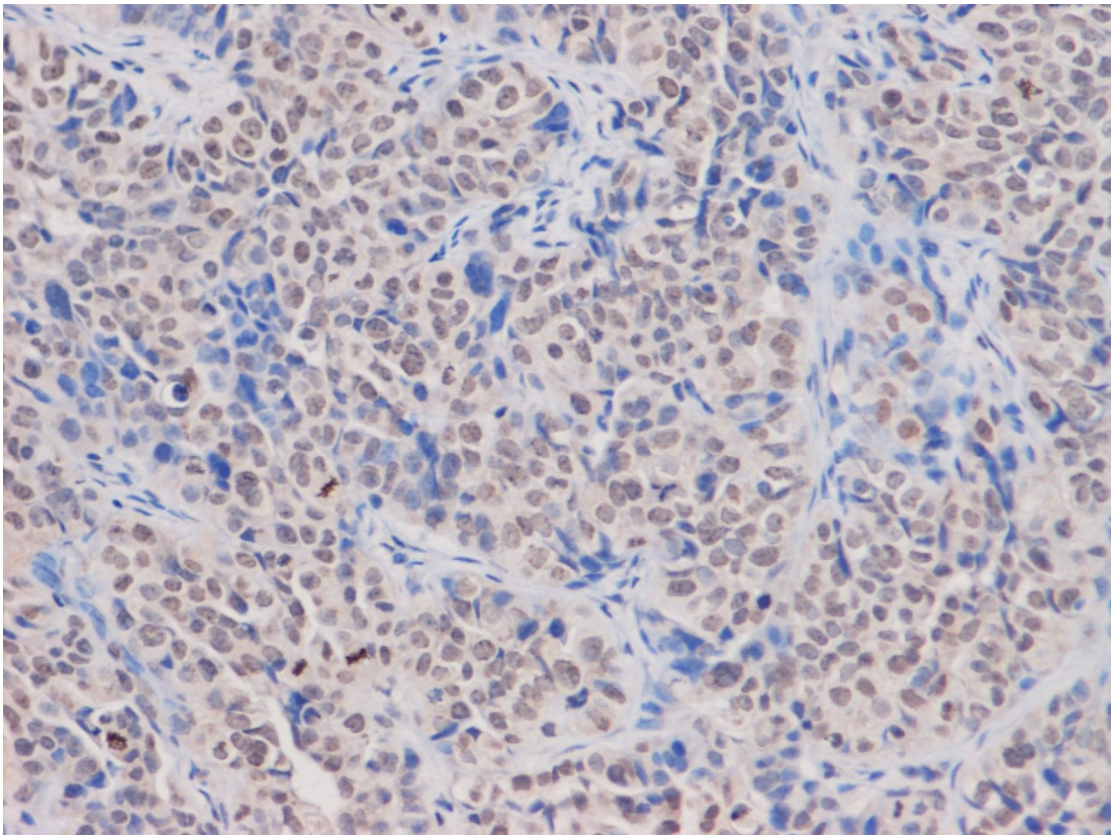

Fig.7D-E2F3-IGF2BP3-OE+sh-LINC00958

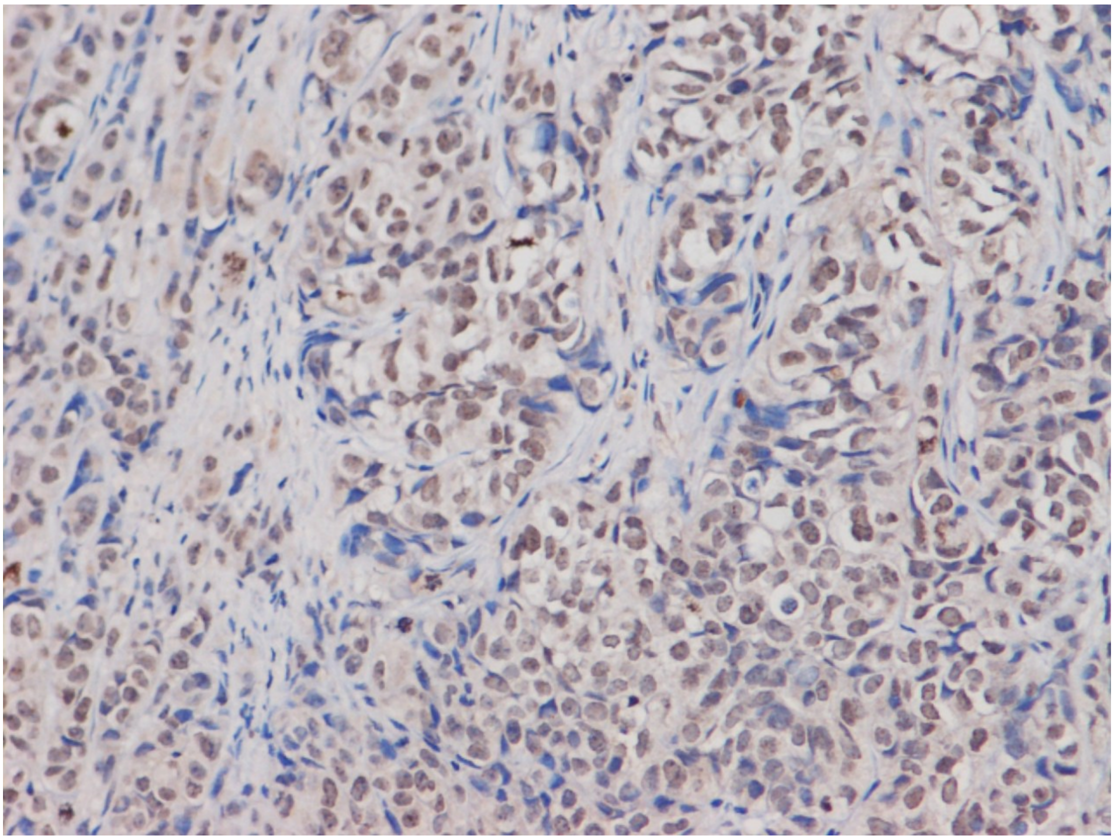

Fig.7D-E2F3-IGF2BP3-OE+sh-NC

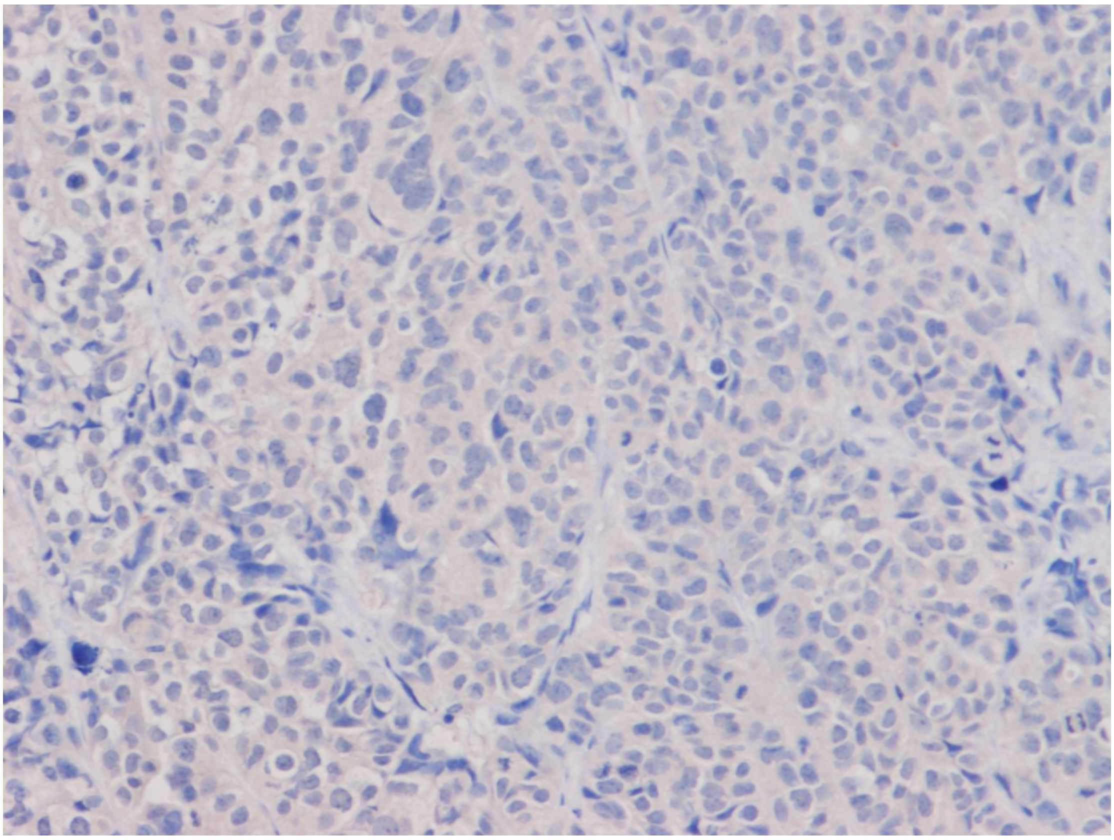

Fig.7D-E2F3-LV-NC+sh-LINC00958

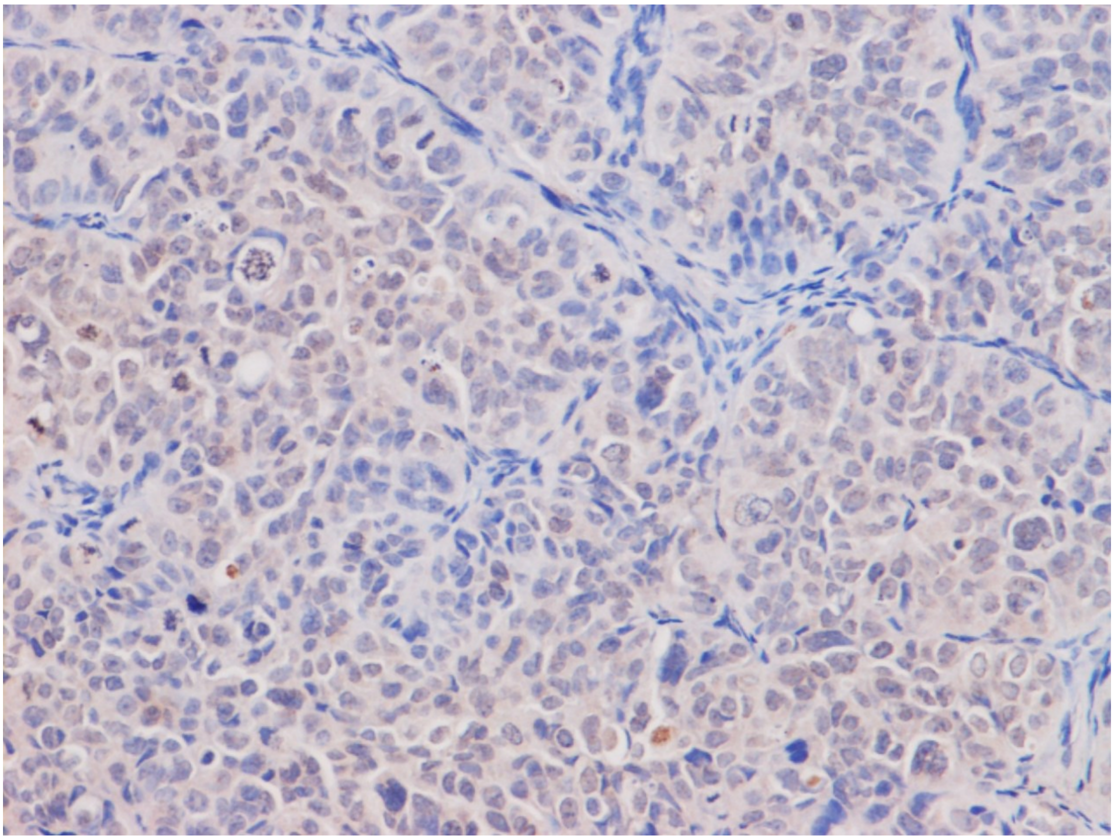

Fig.7D-E2F3-LV-NC+sh-NC

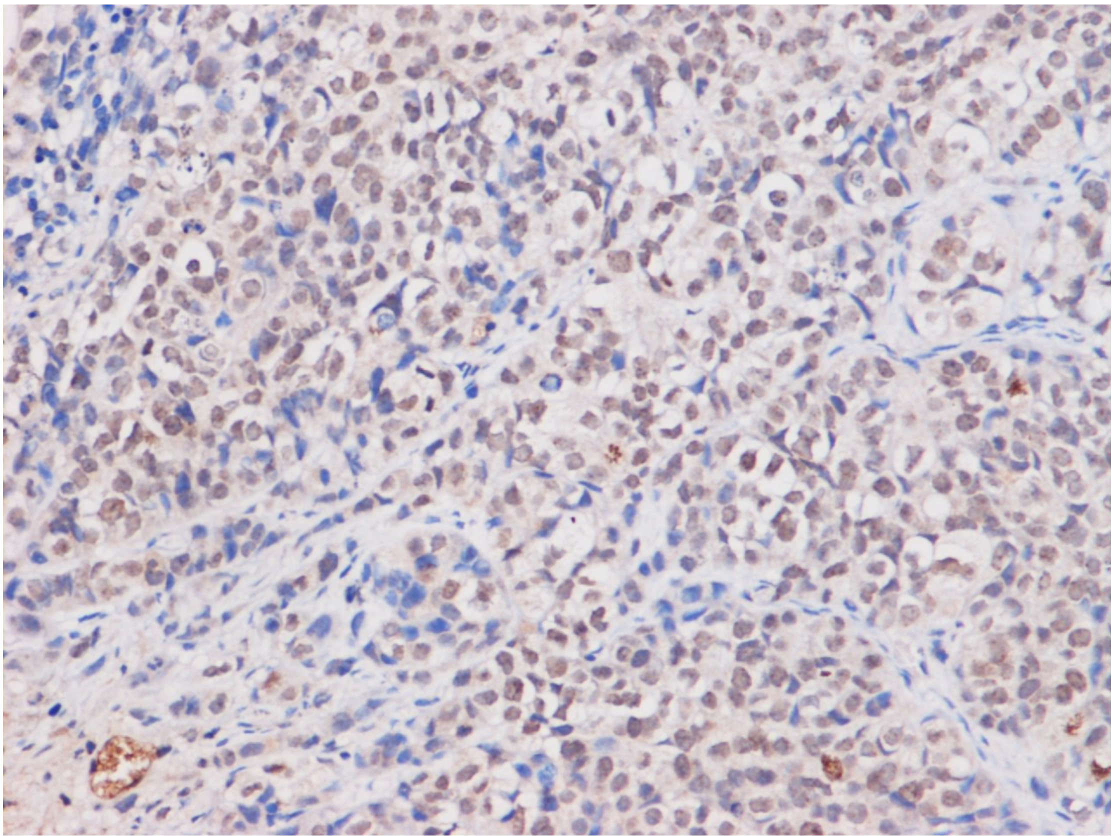

Fig.7D-Ki67-IGF2BP3-OE+sh-LINC00958

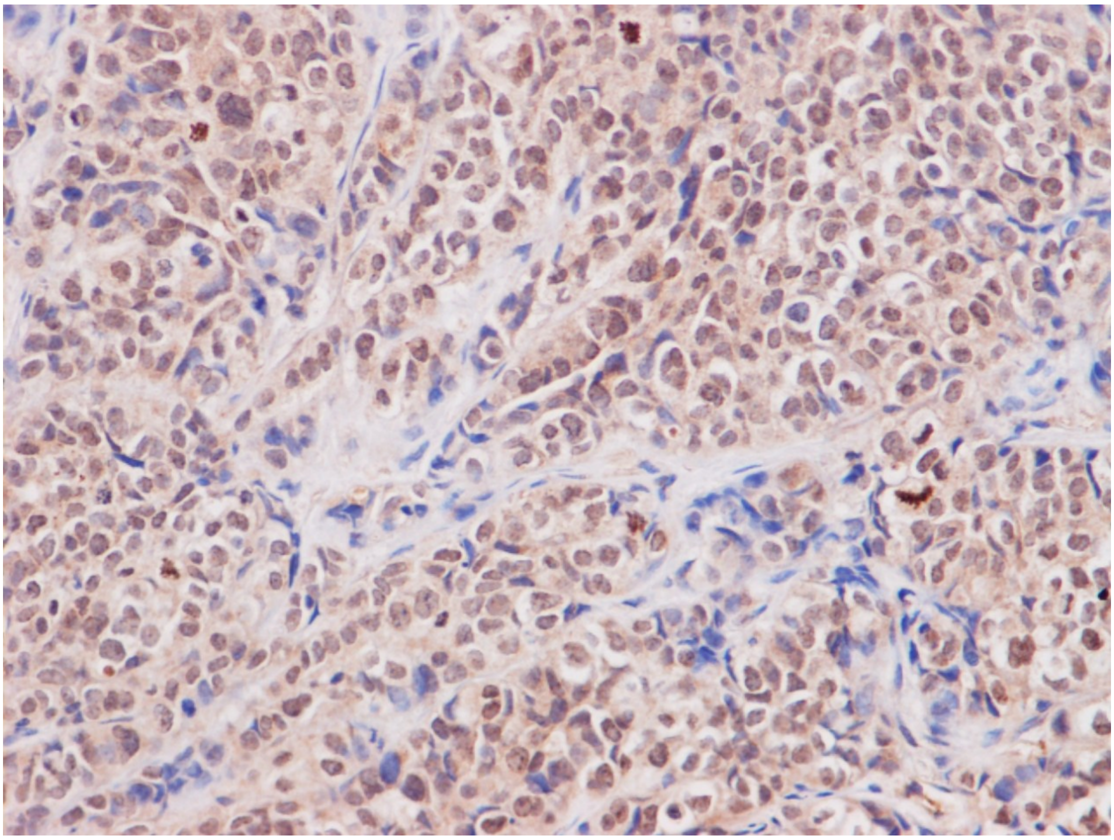

Fig.7D-Ki67-IGF2BP3-OE+sh-NC

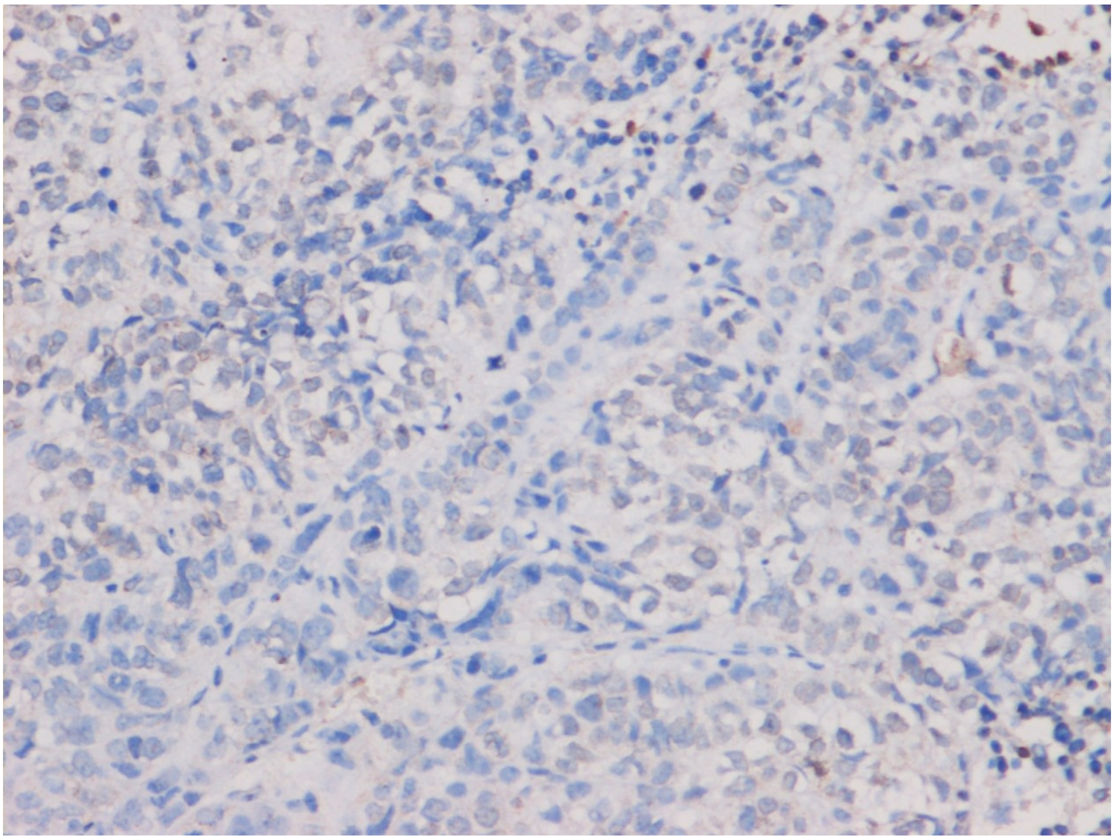

Fig.7D-Ki67-LV-NC+sh-LINC00958

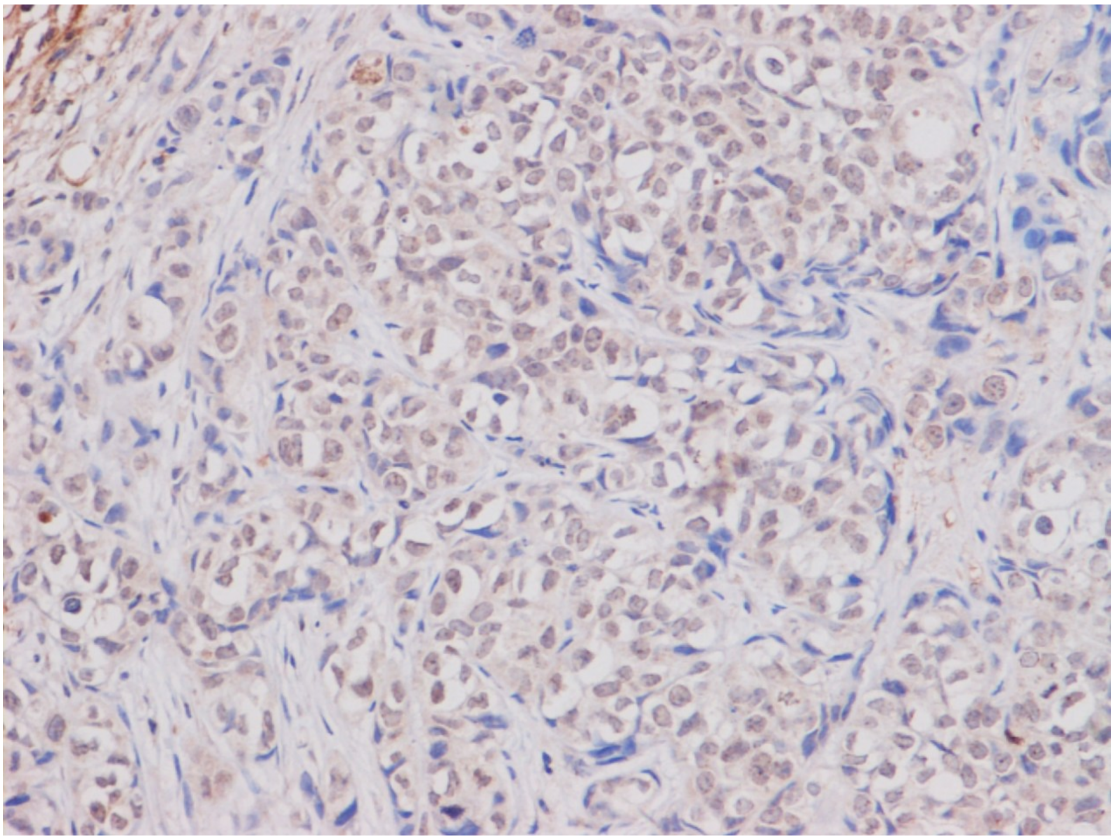

Fig.7D-Ki67-LV-NC+sh-NC

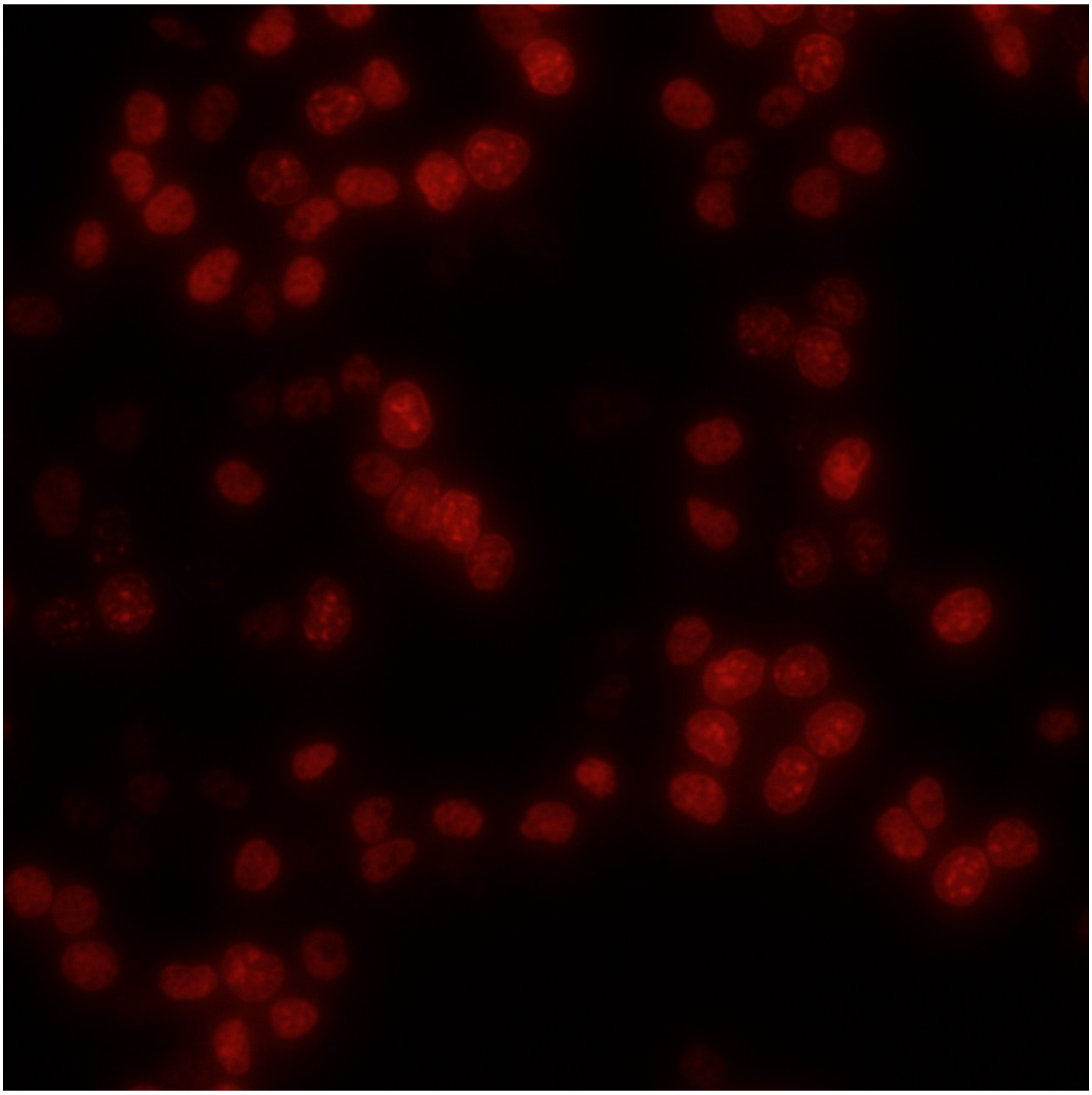

Fig.S1E-HEC-1-A-IGF2BP3-OE-EdU

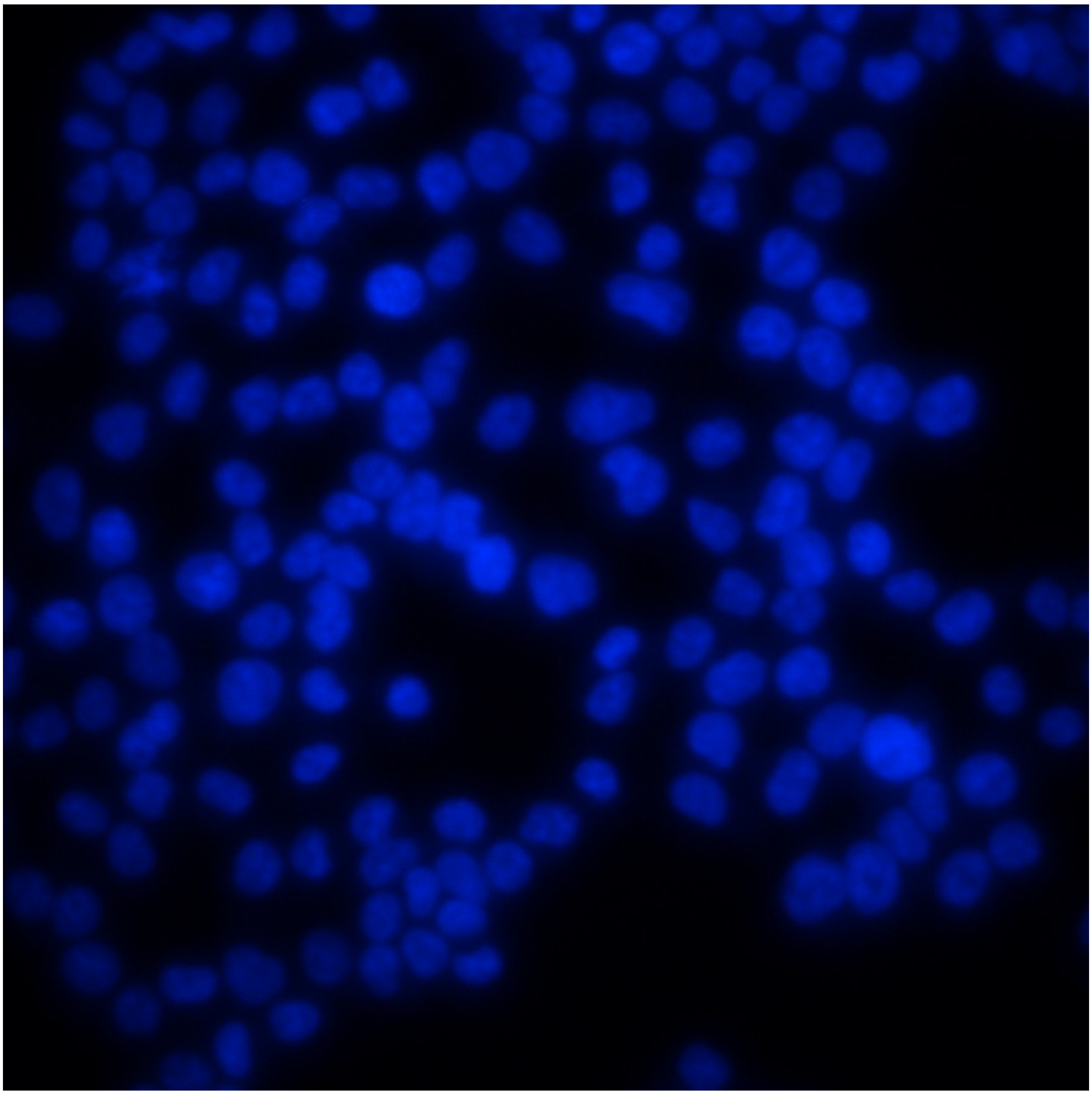

Fig.S1E-HEC-1-A-IGF2BP3-OE-Hoechst

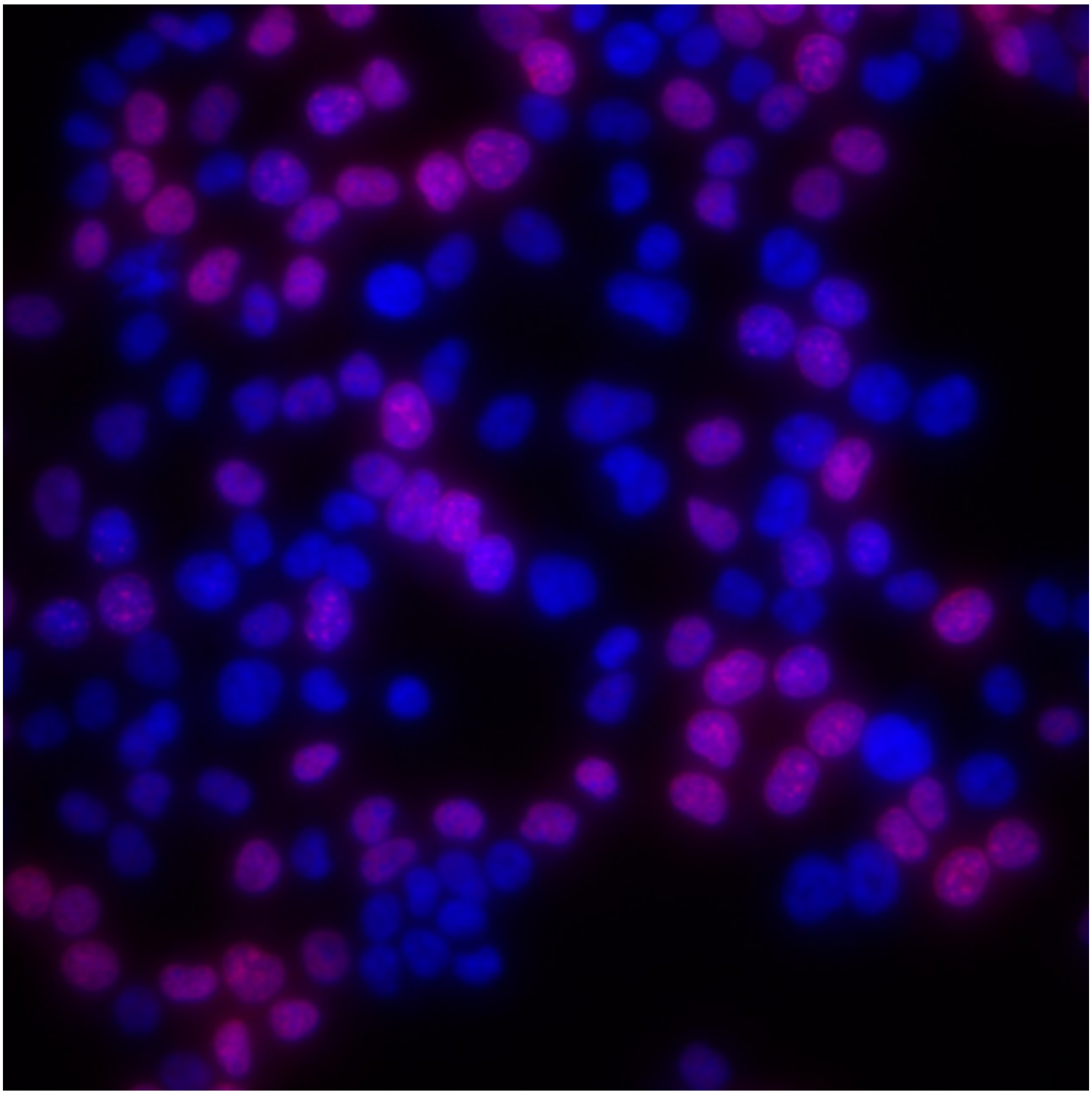

Fig.S1E-HEC-1-A-IGF2BP3-OE-Merged

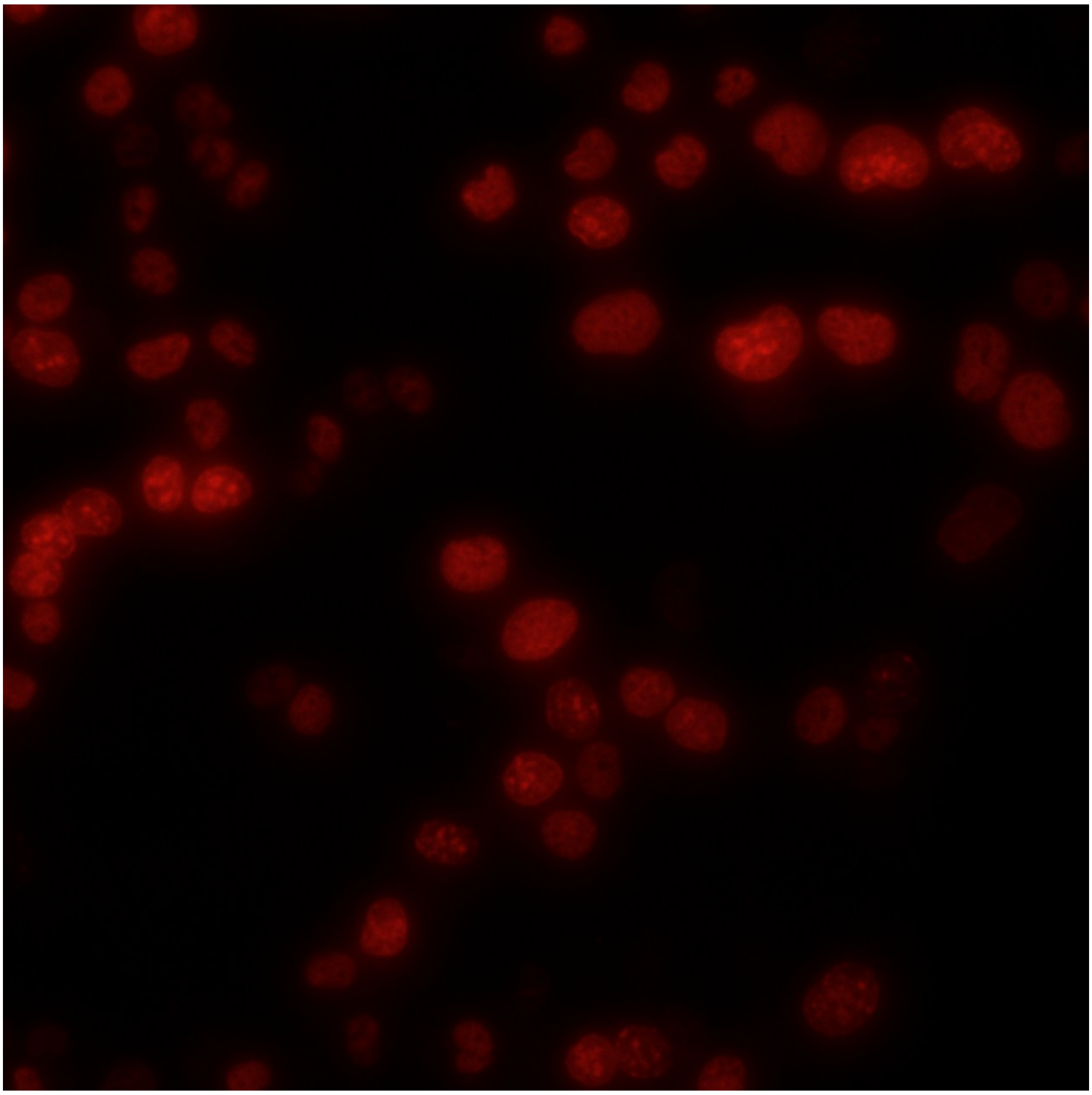

Fig.S1E-HEC-1-A-LV-NC-EdU

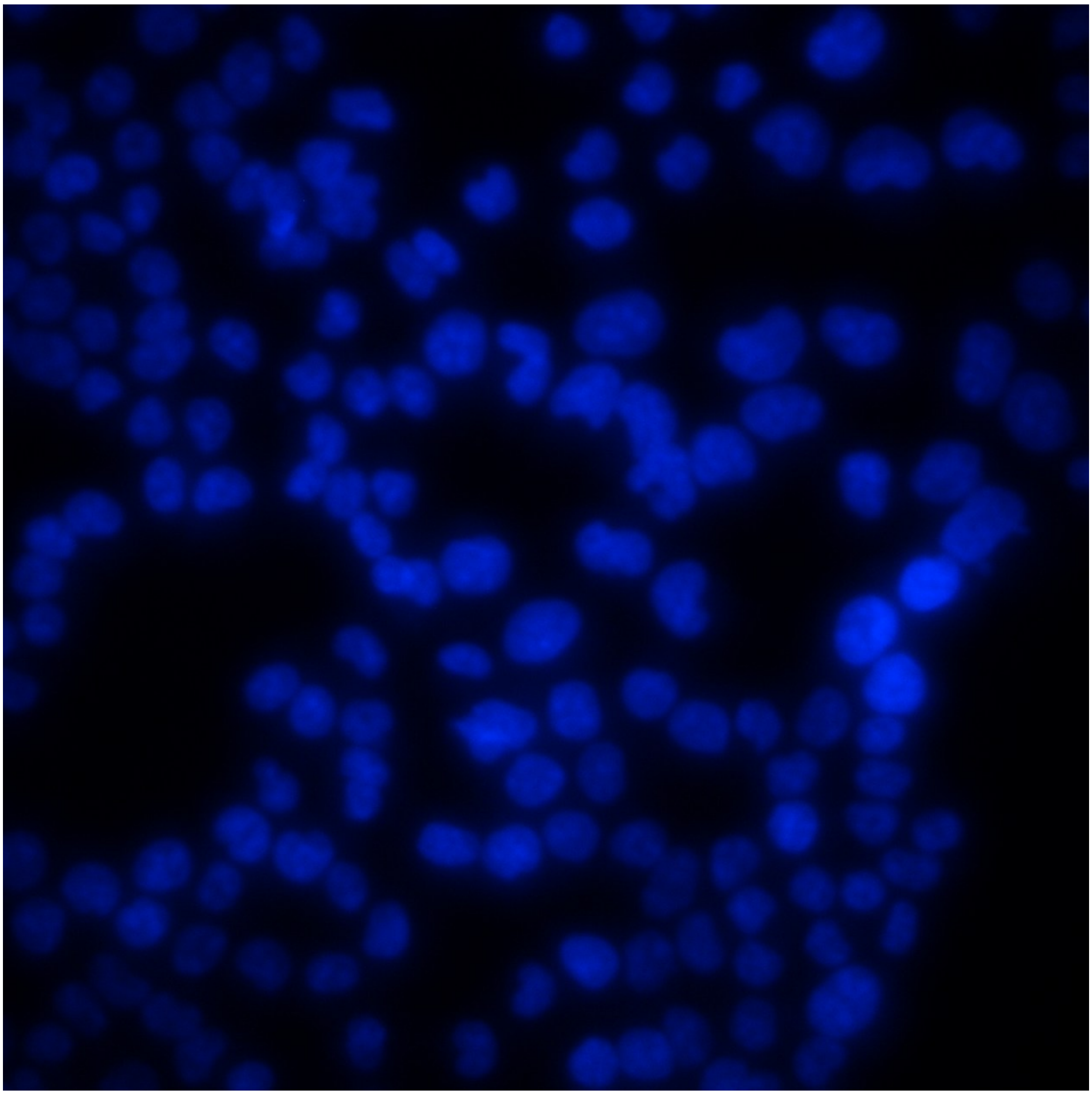

Fig.S1E-HEC-1-A-LV-NC-Hoechst

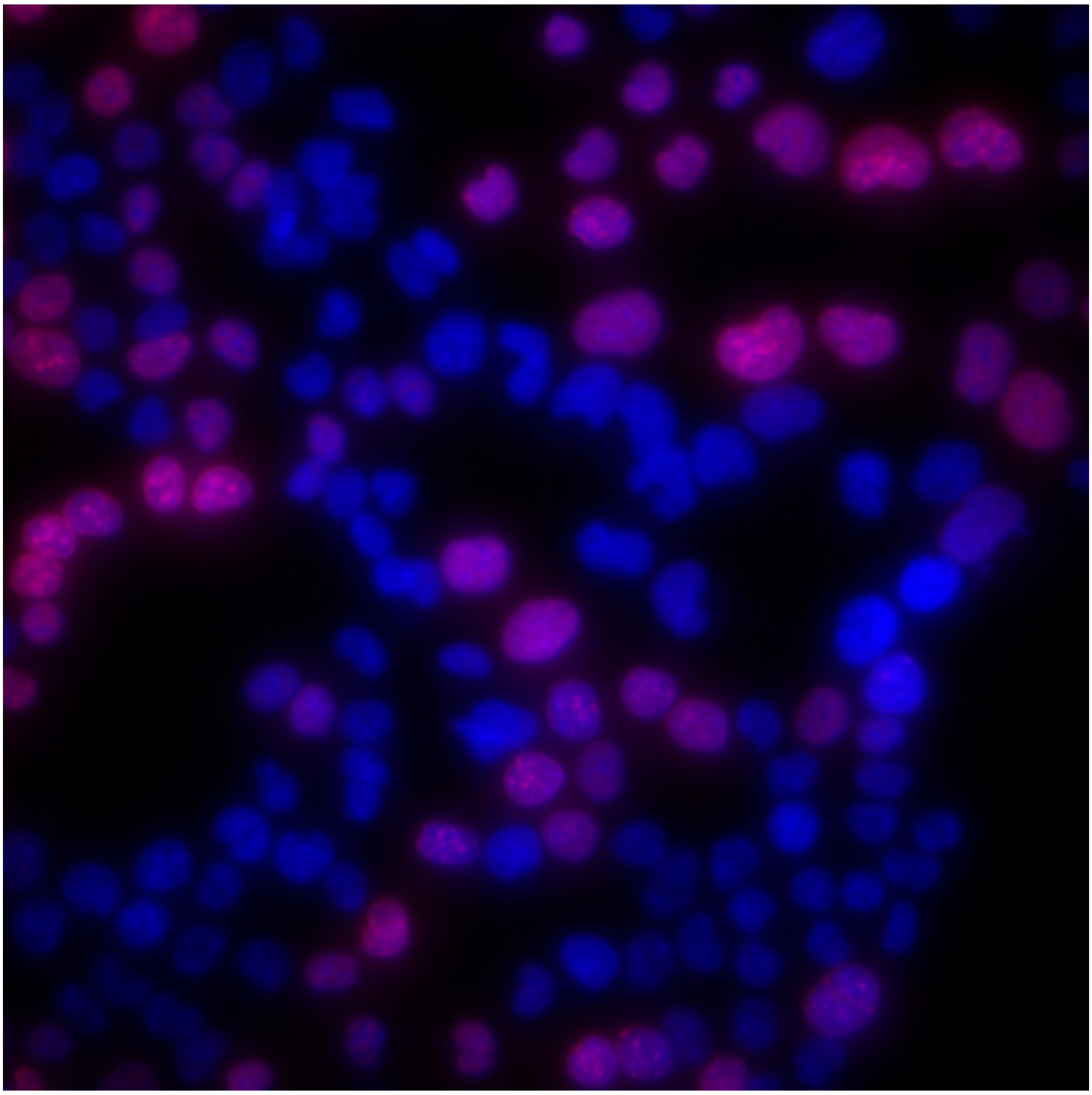

Fig.S1E-HEC-1-A-LV-NC-Merged

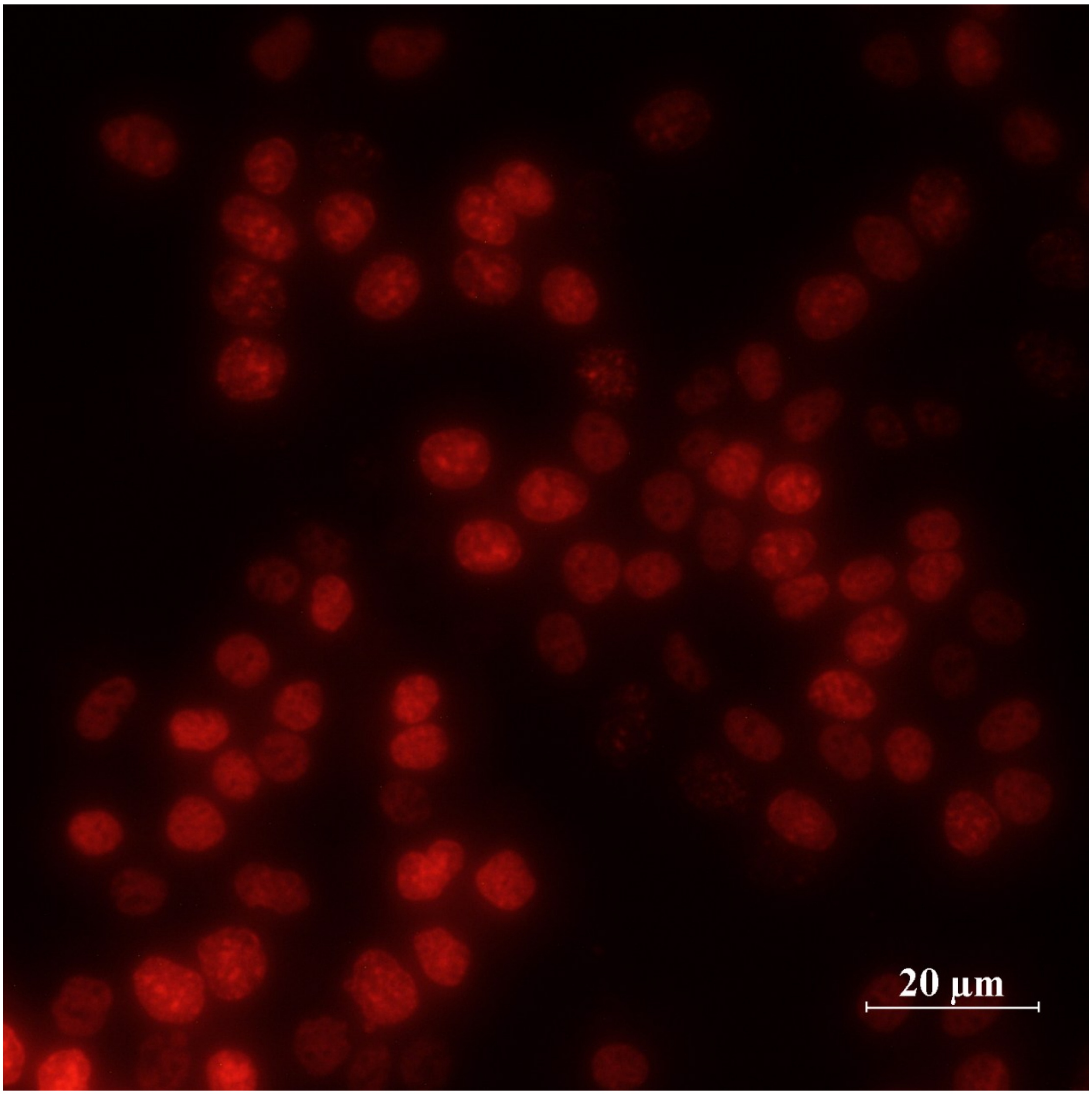

Fig.S1E-Ishikawa-IGF2BP3-OE-EdU

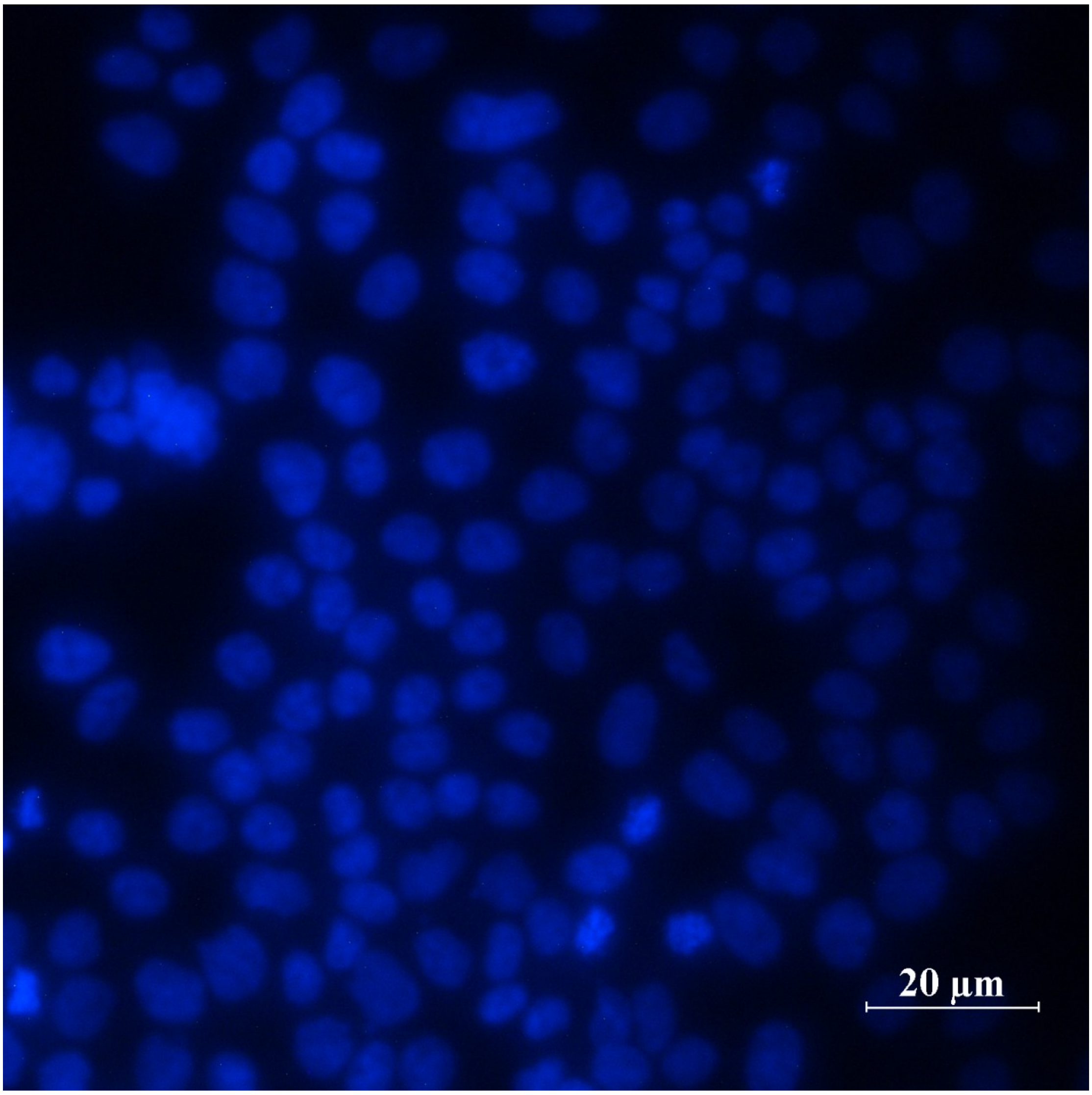

Fig.S1E-Ishikawa-IGF2BP3-OE-Hoechst

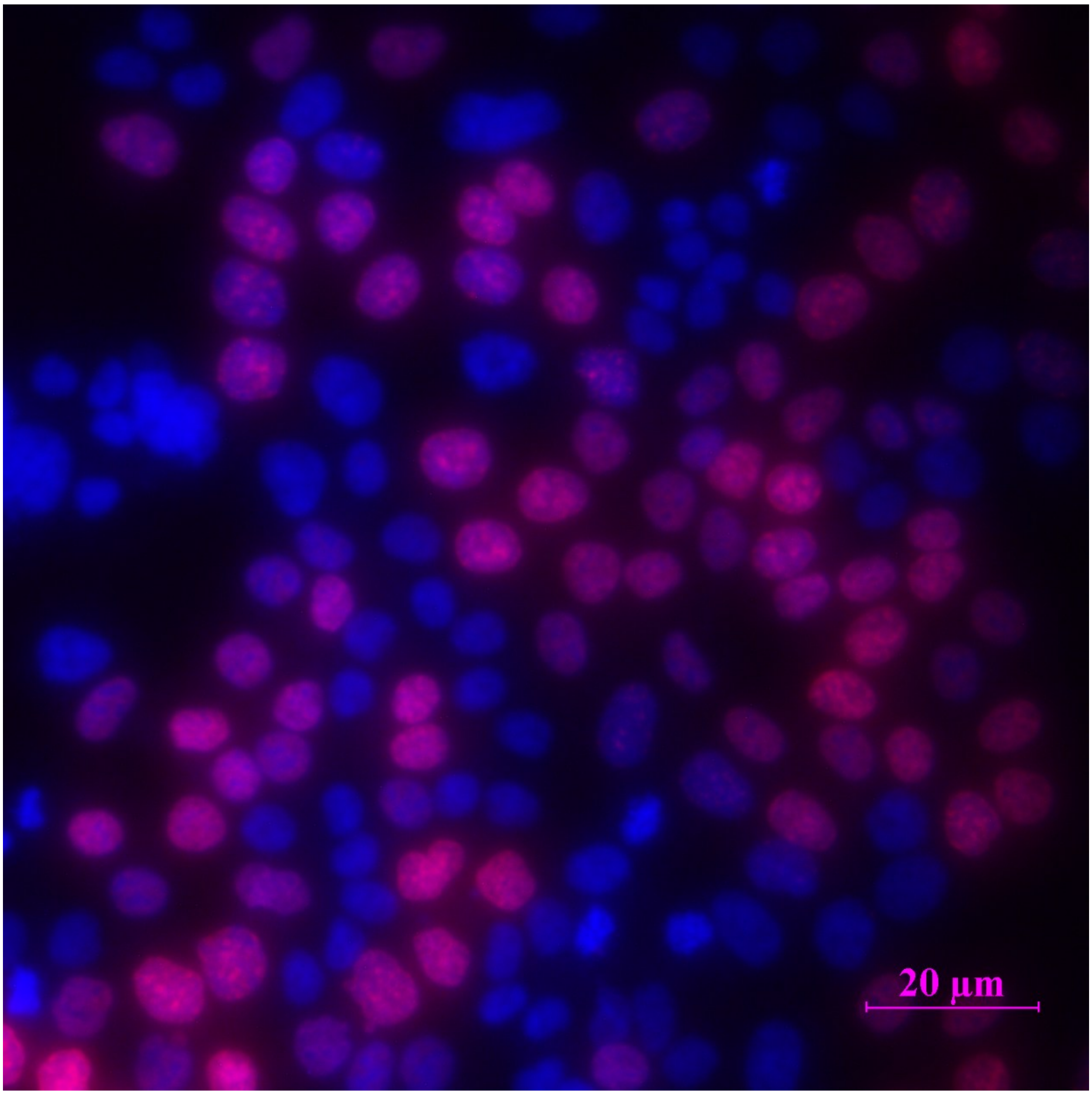

Fig.S1E-Ishikawa-IGF2BP3-OE-Merged

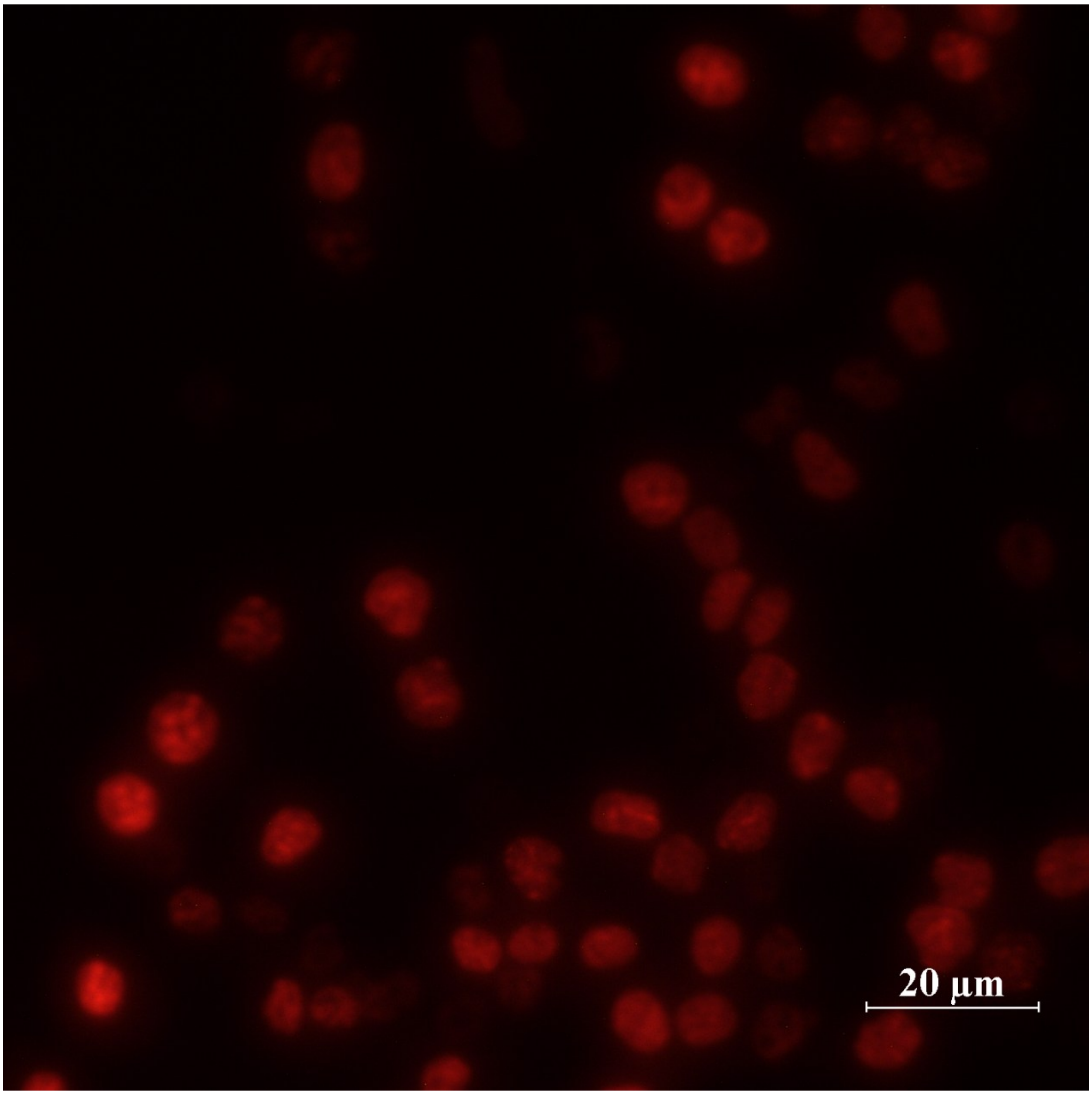

Fig.S1E-Ishikawa-LV-NC-EdU

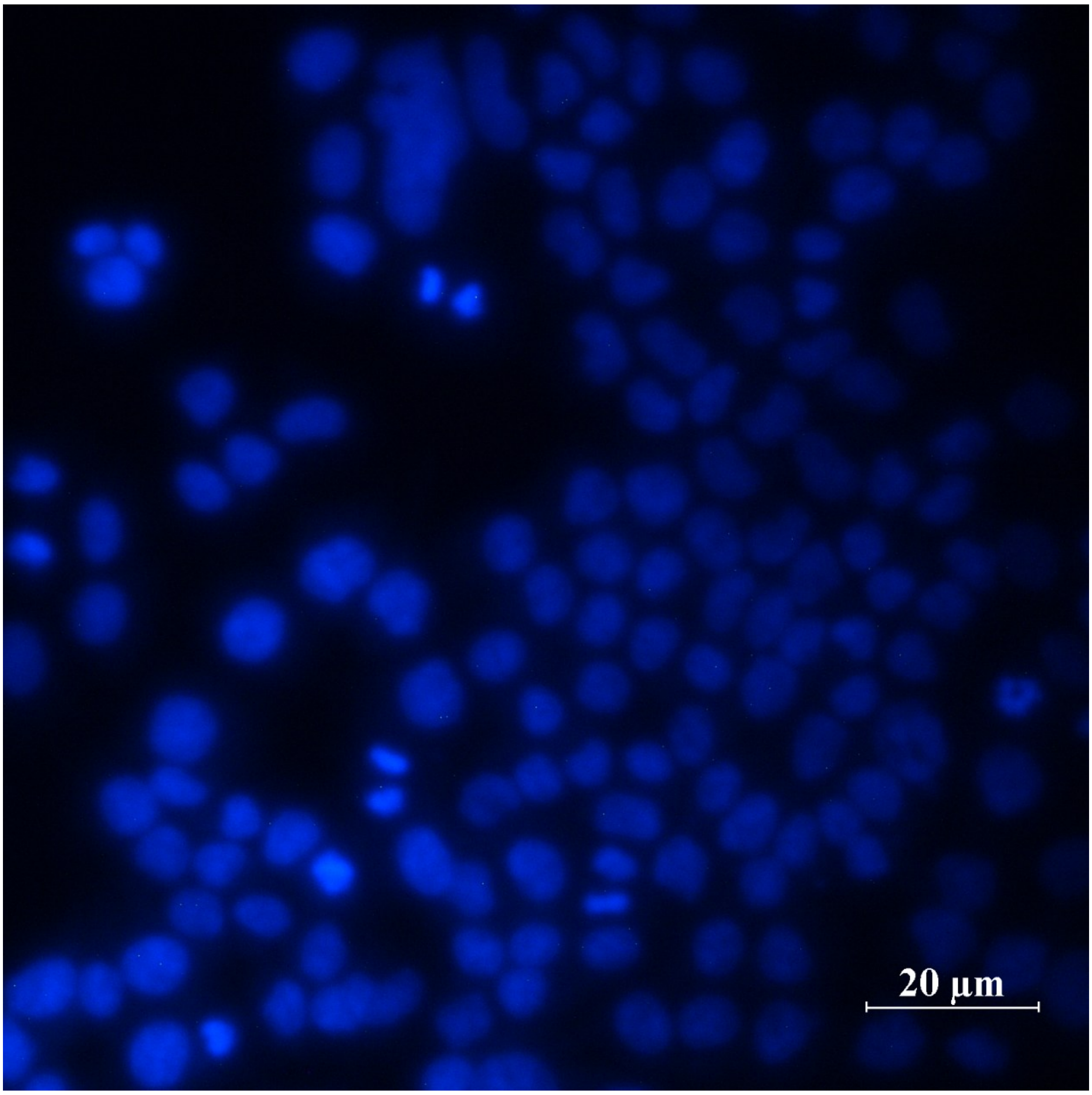

Fig.S1E-Ishikawa-LV-NC-Hoechst

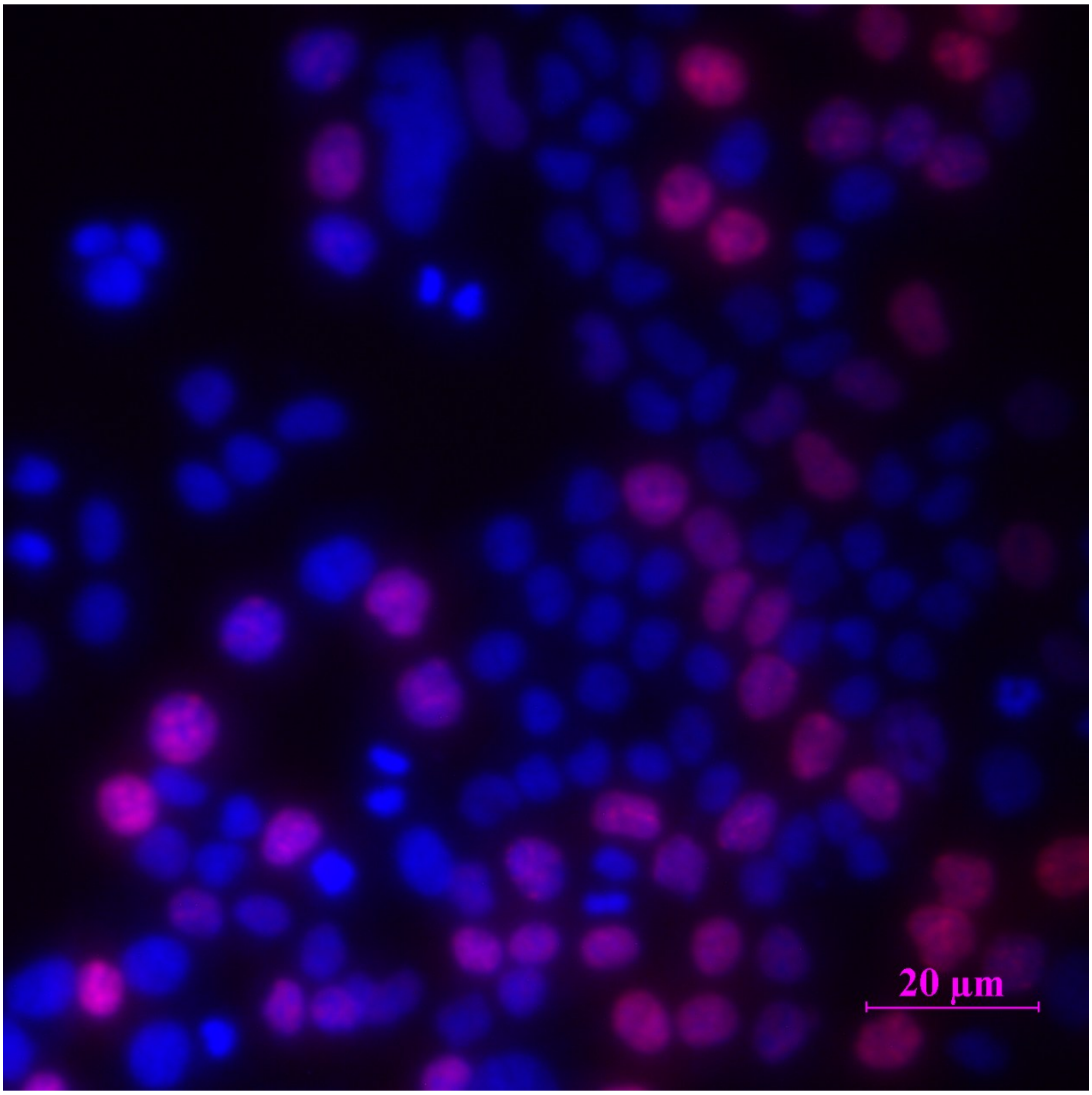

Fig.S1E-Ishikawa-LV-NC-Merge

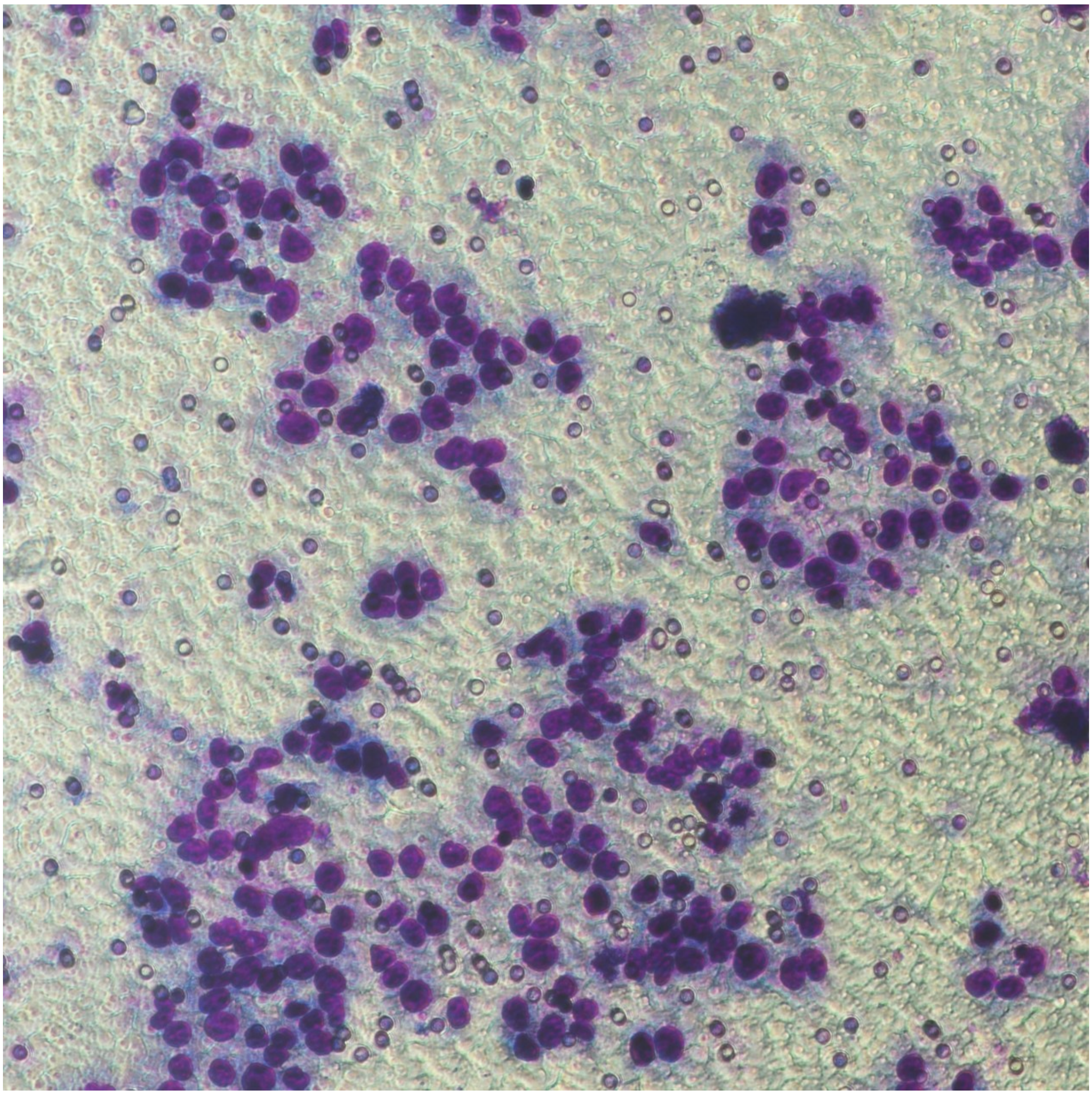

Fig.S1F-HEC-1-A-invasion-IGF2BP3-OE

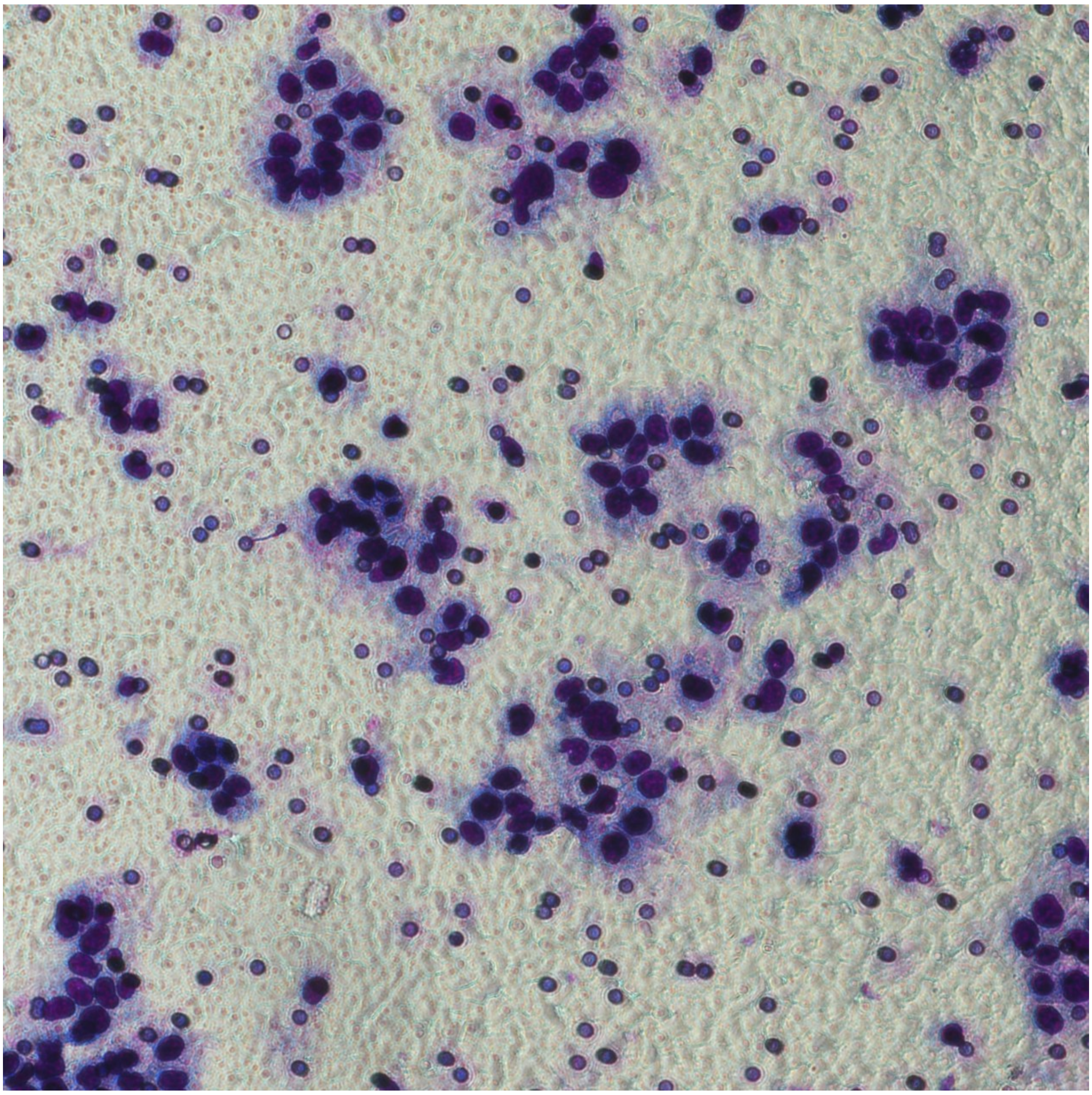

Fig.S1F-HEC-1-A-invasion-LV-NC

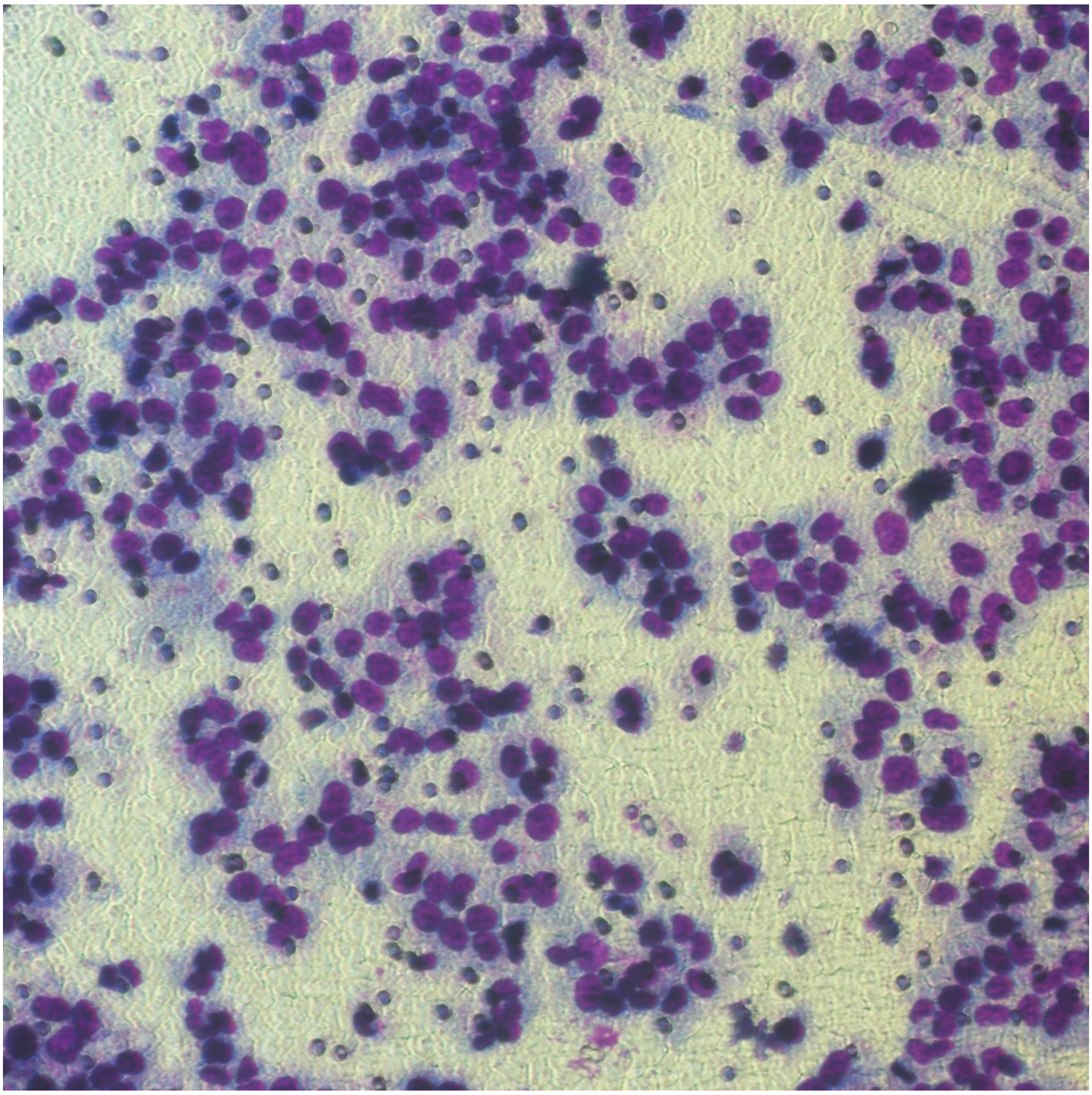

Fig.S1F-HEC-1-A-migration-IGF2BP3-OE

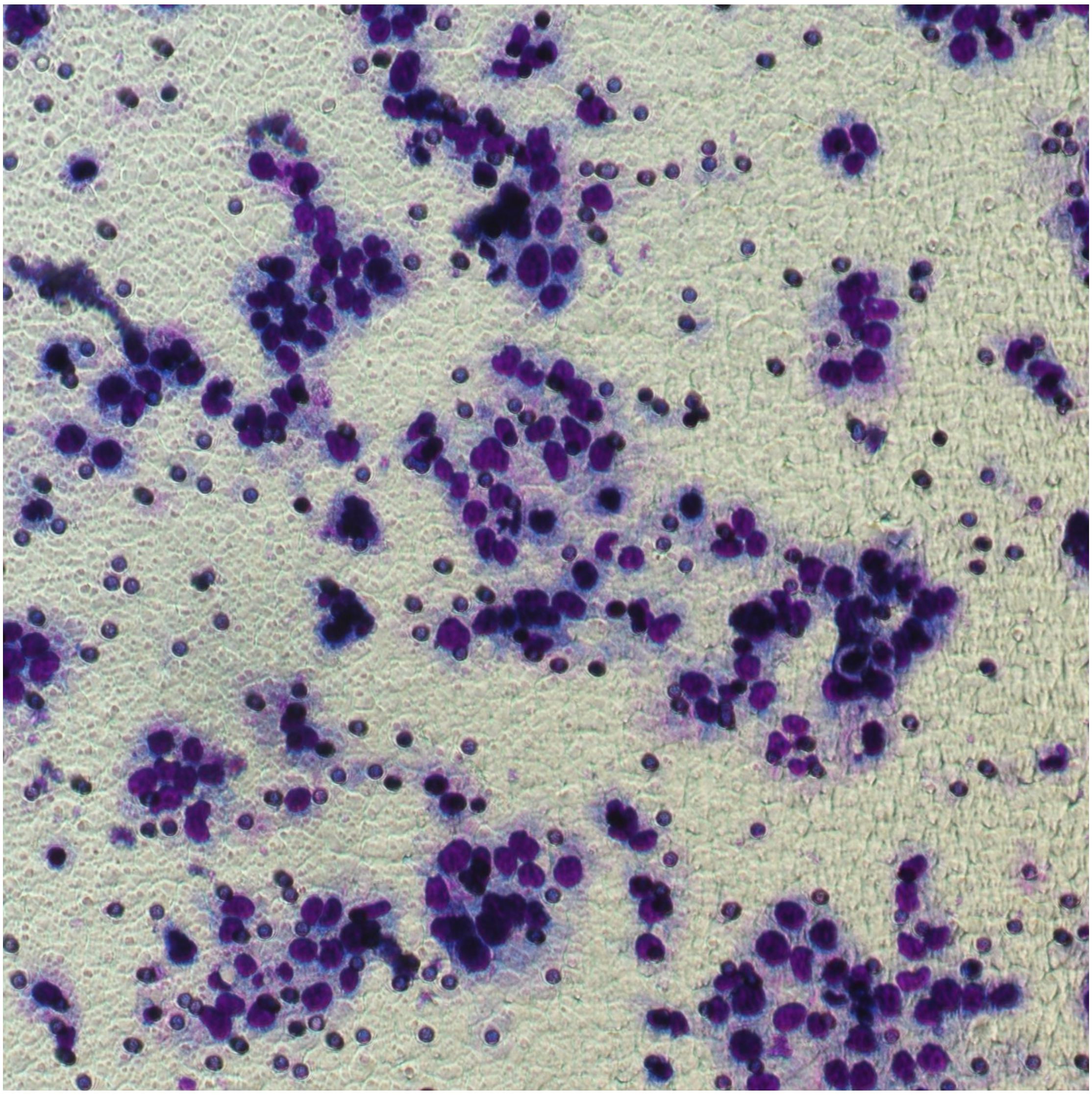

Fig.S1F-HEC-1-A-migration-LV-NC

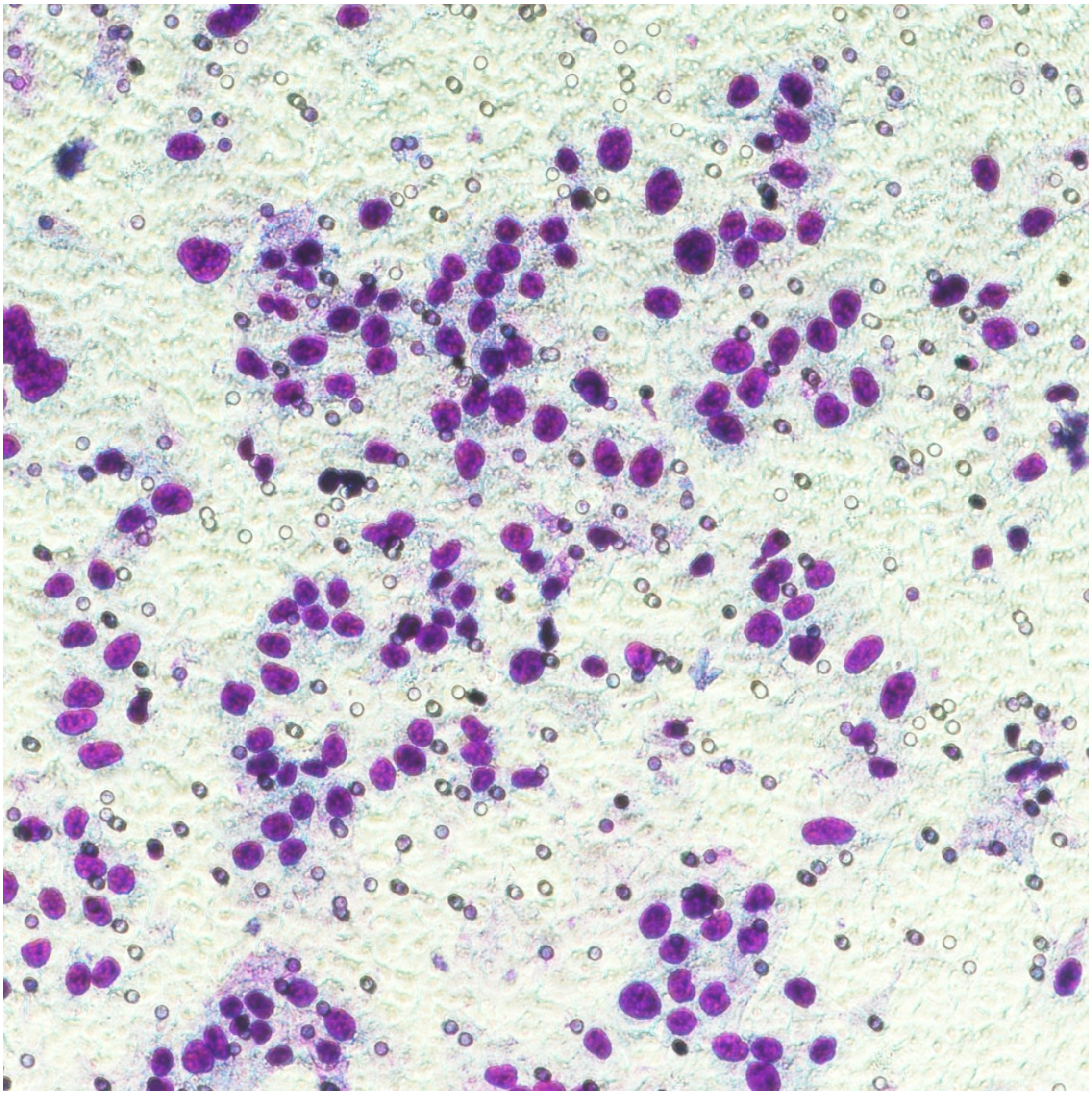

Fig.S1F-Ishikawa-invasion-IGF2BP3-OE

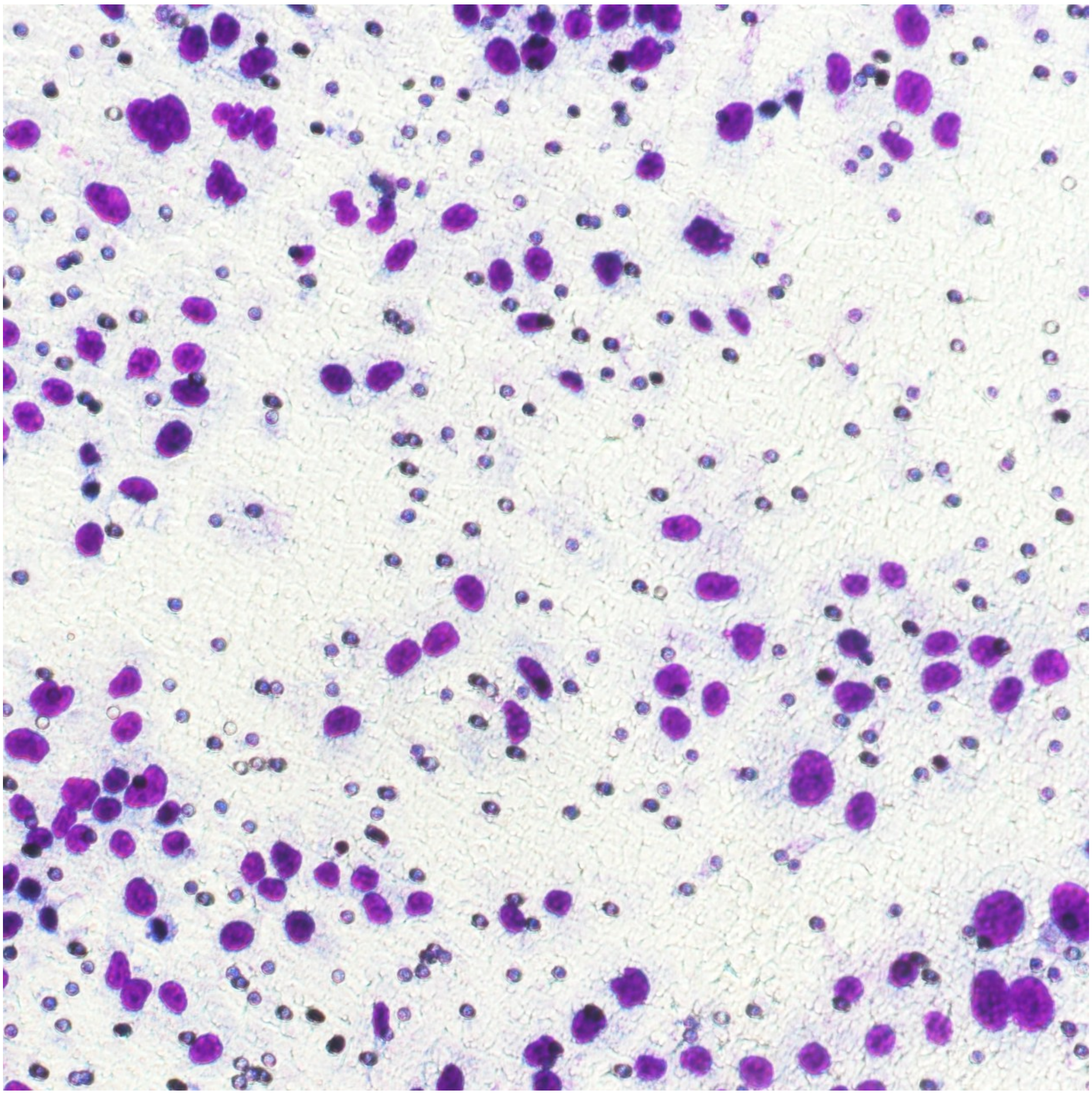

Fig.S1F-Ishikawa-invasion-LV-NC

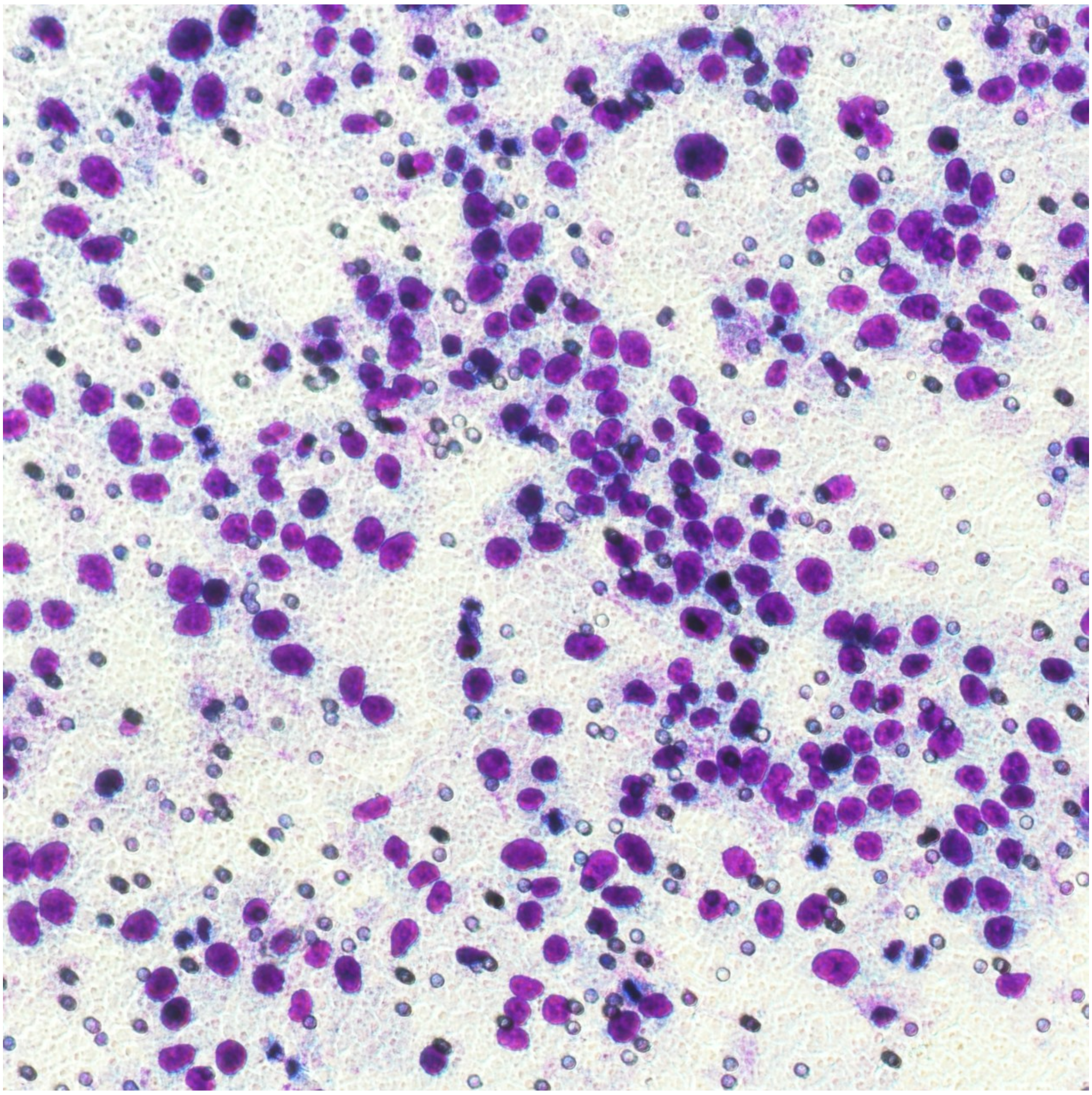

Fig.S1F-Ishikawa-migration-IGF2BP3-OE

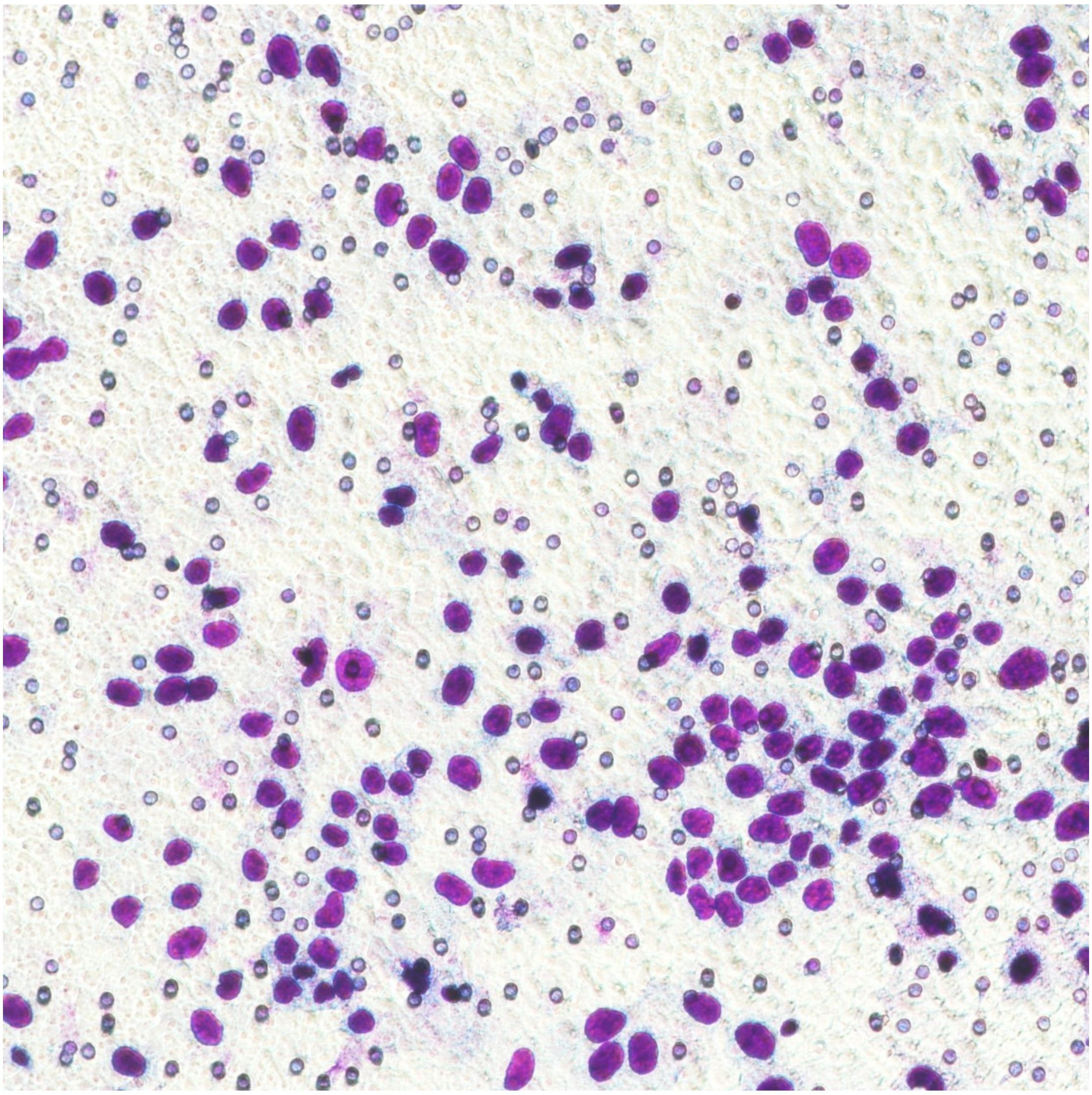

Fig.S1F-Ishikawa-migration-LV-NC

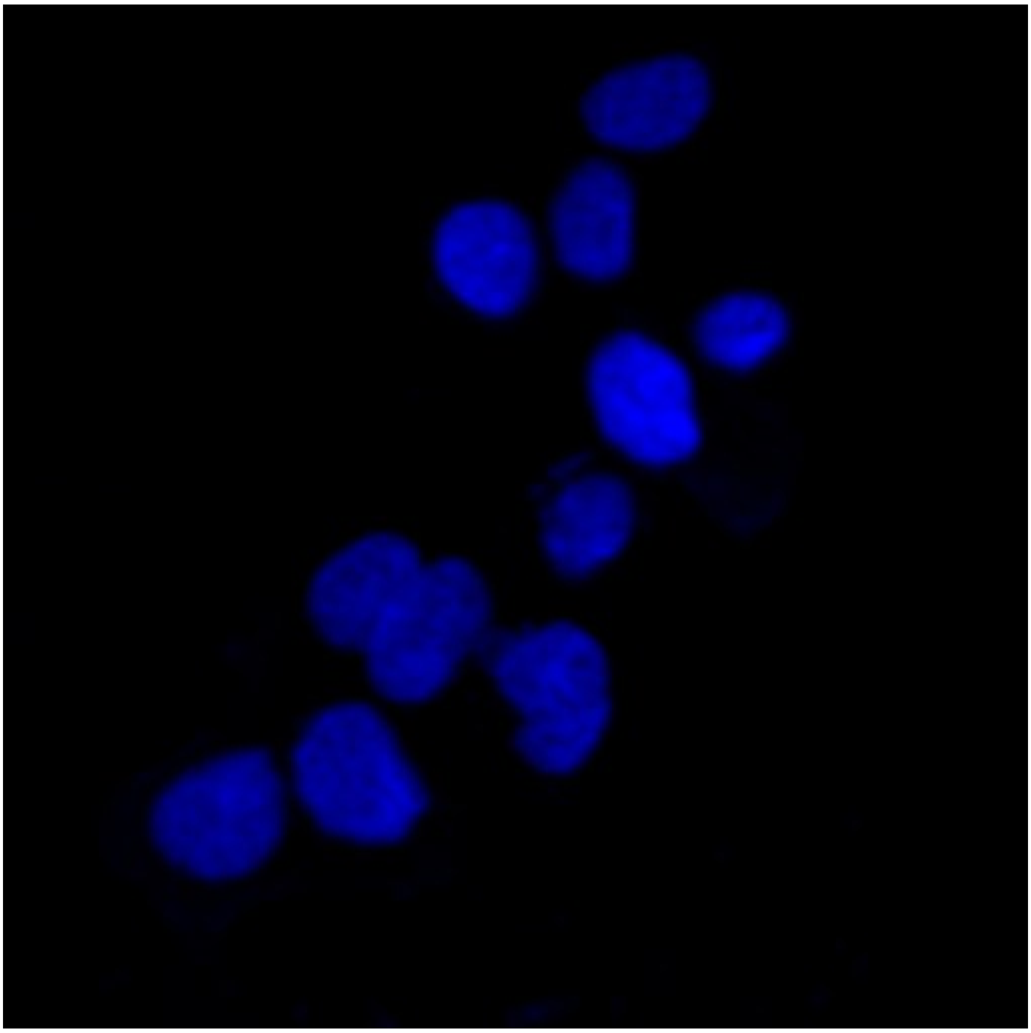

Fig.S2F-HEC-1-A-DAPI

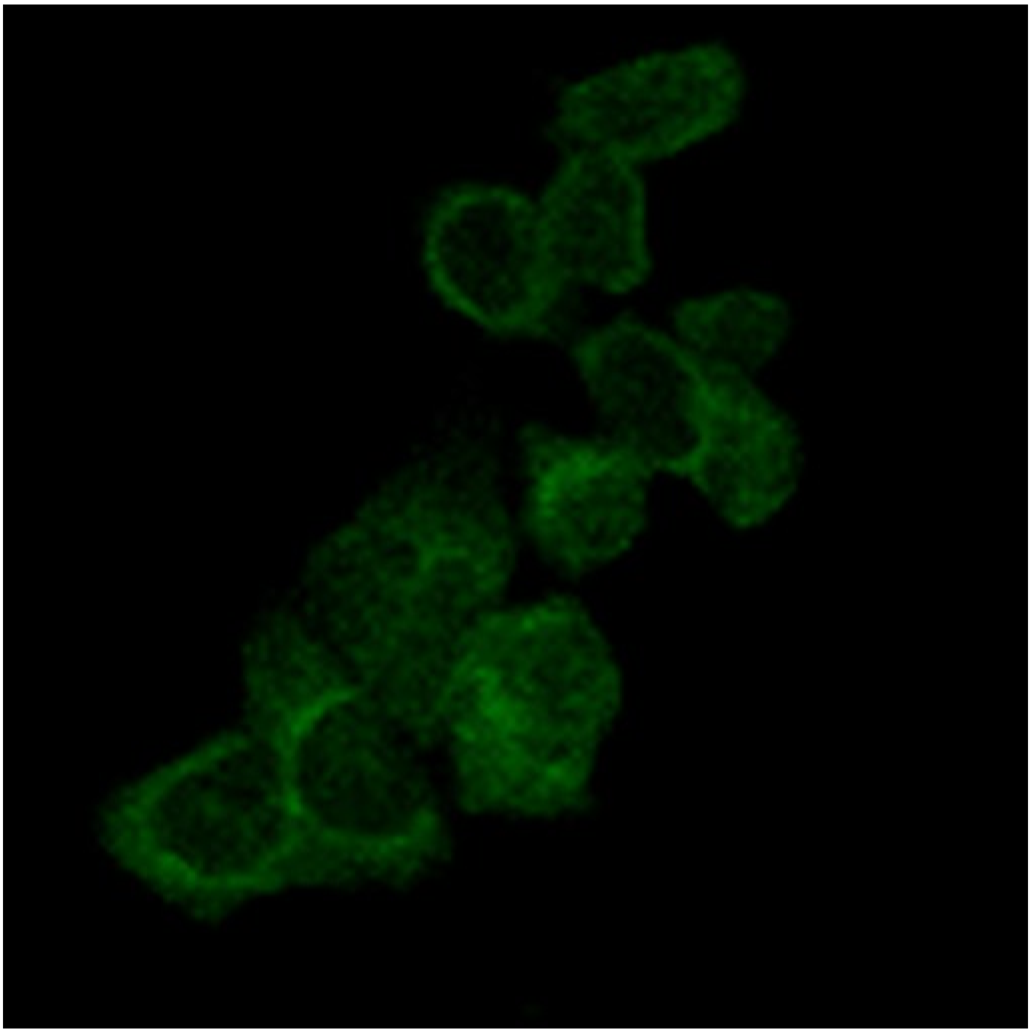

Fig.S2F-HEC-1-A-IGF2BP3

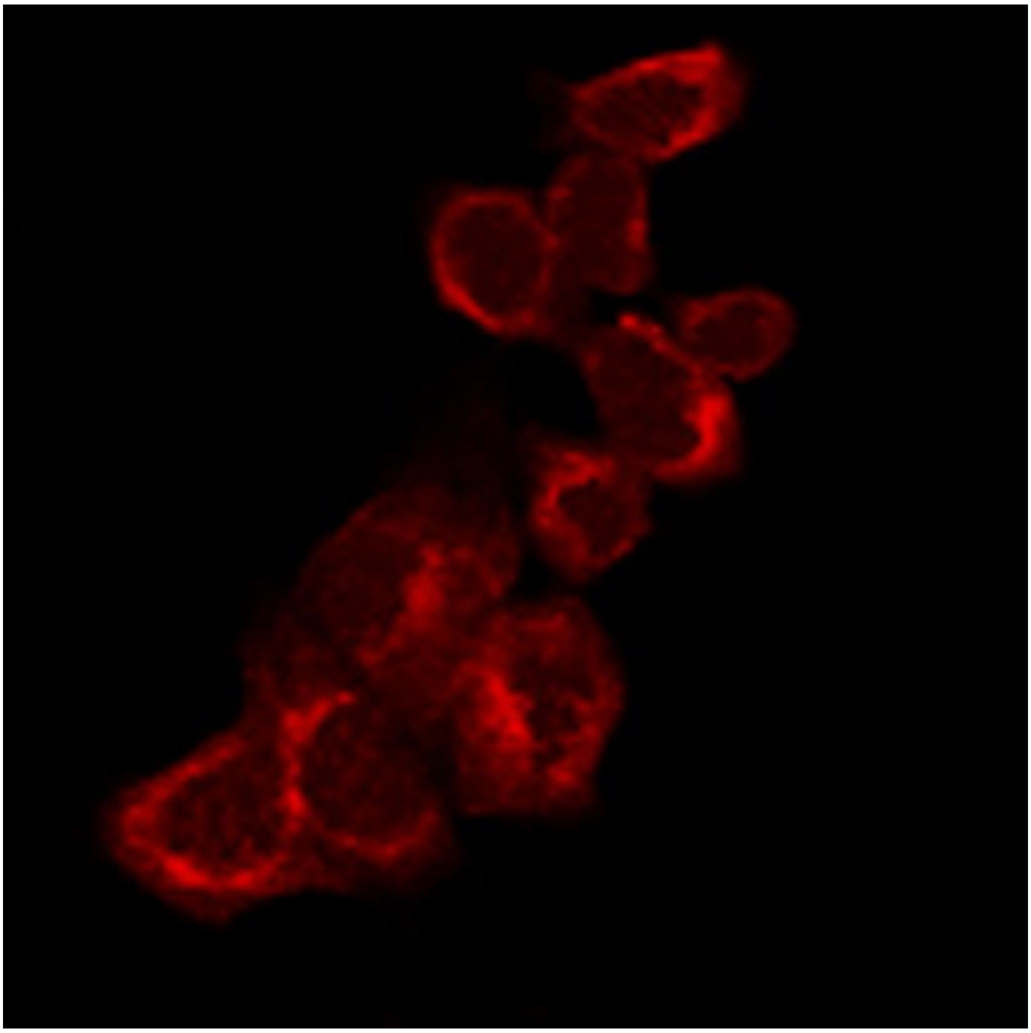

Fig.S2F-HEC-1-A-LINC00958

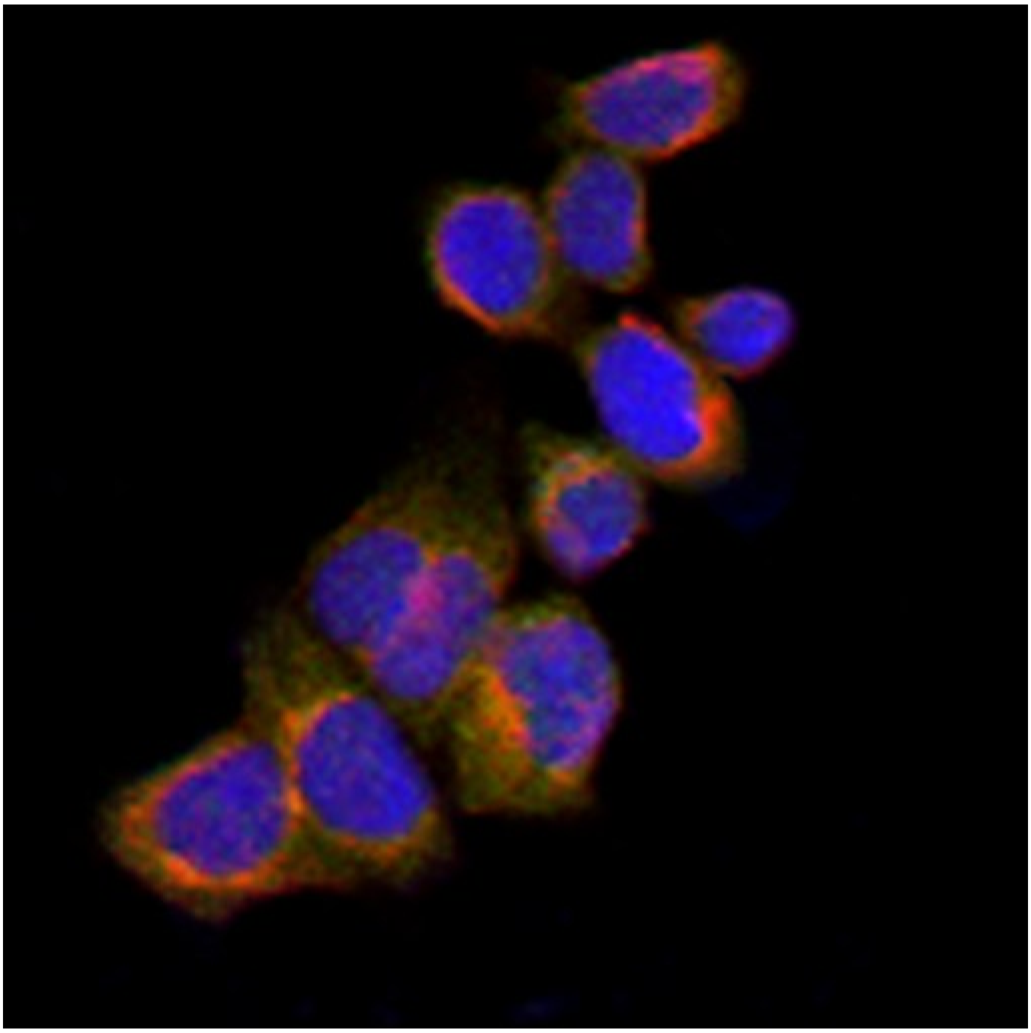

Fig.S2F-HEC-1-A-Merge

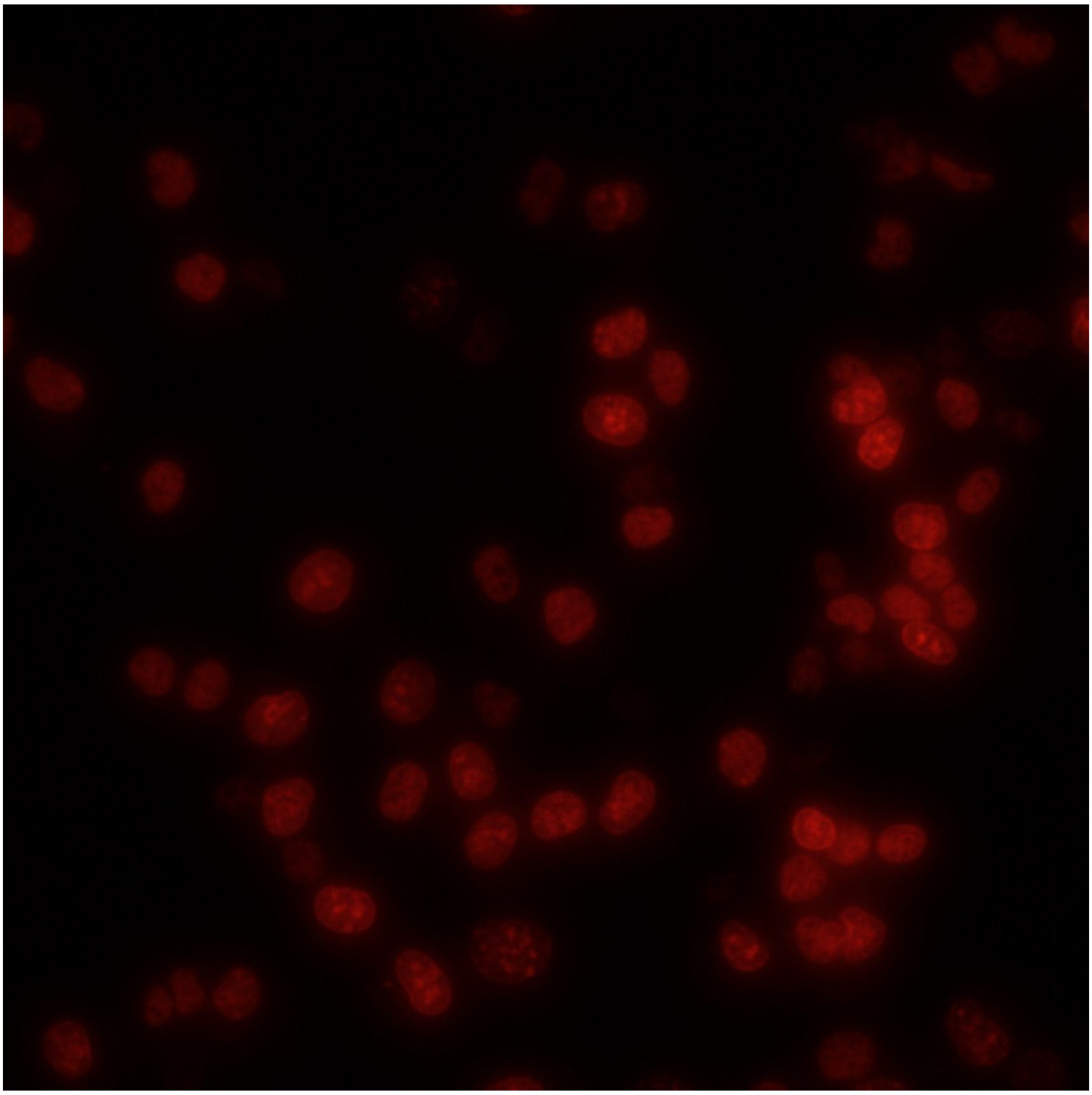

Fig.S3A-HEC-1-A-IGF2BP3-OE+sh-LINC00958-EdU

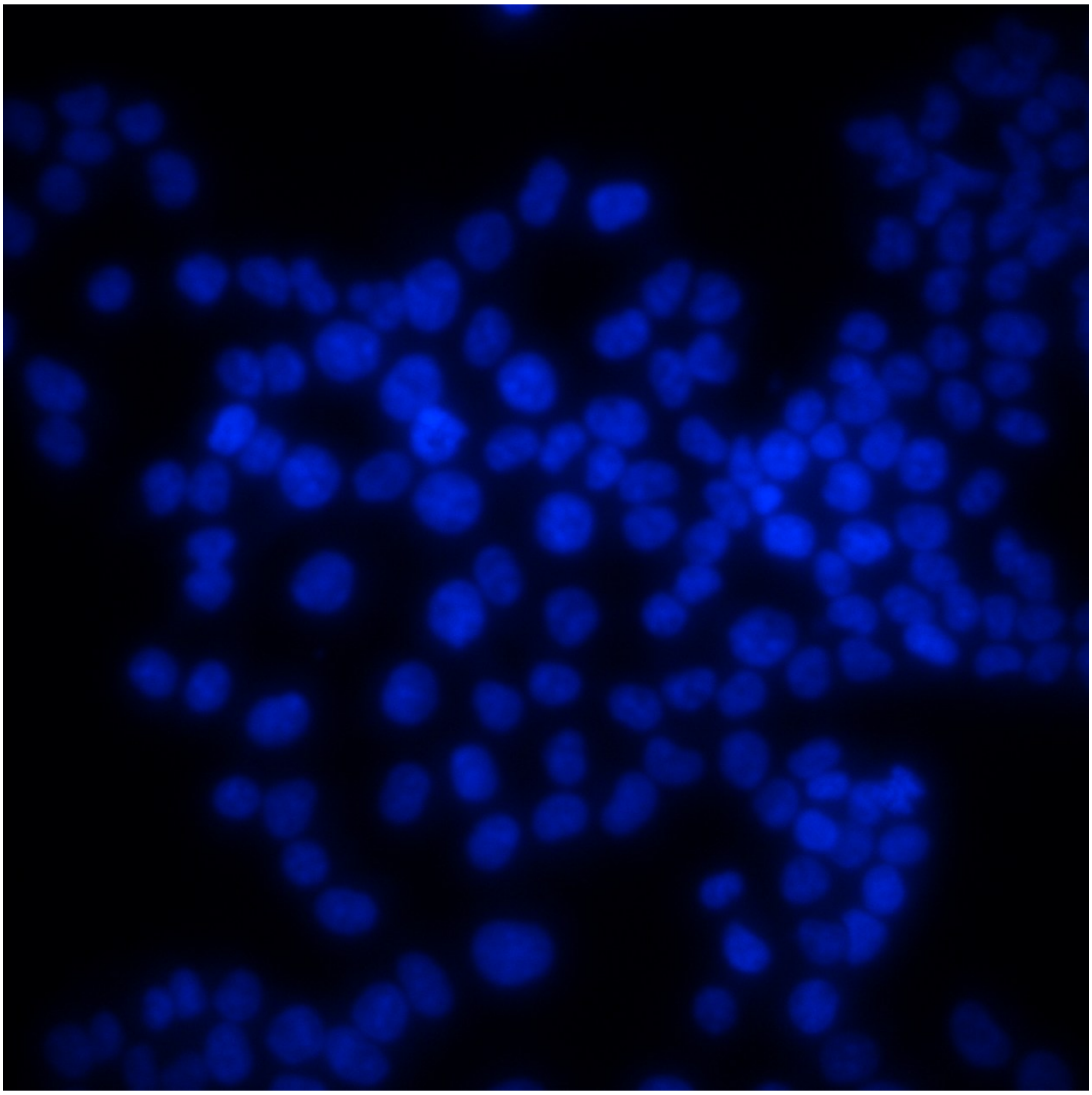

Fig.S3A-HEC-1-A-IGF2BP3-OE+sh-LINC00958-Hoechst

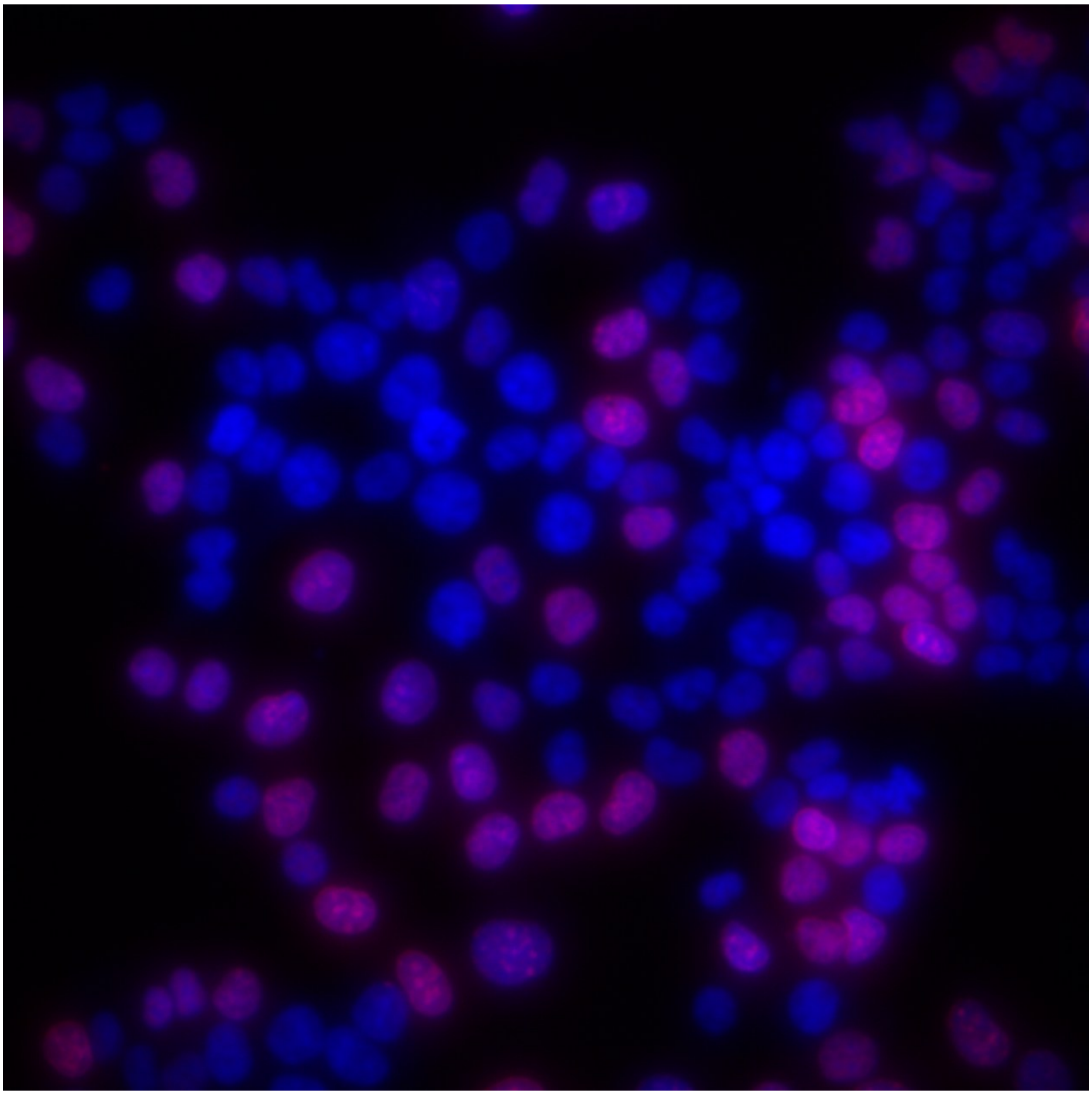

Fig.S3A-HEC-1-A-IGF2BP3-OE+sh-LINC00958-Merge

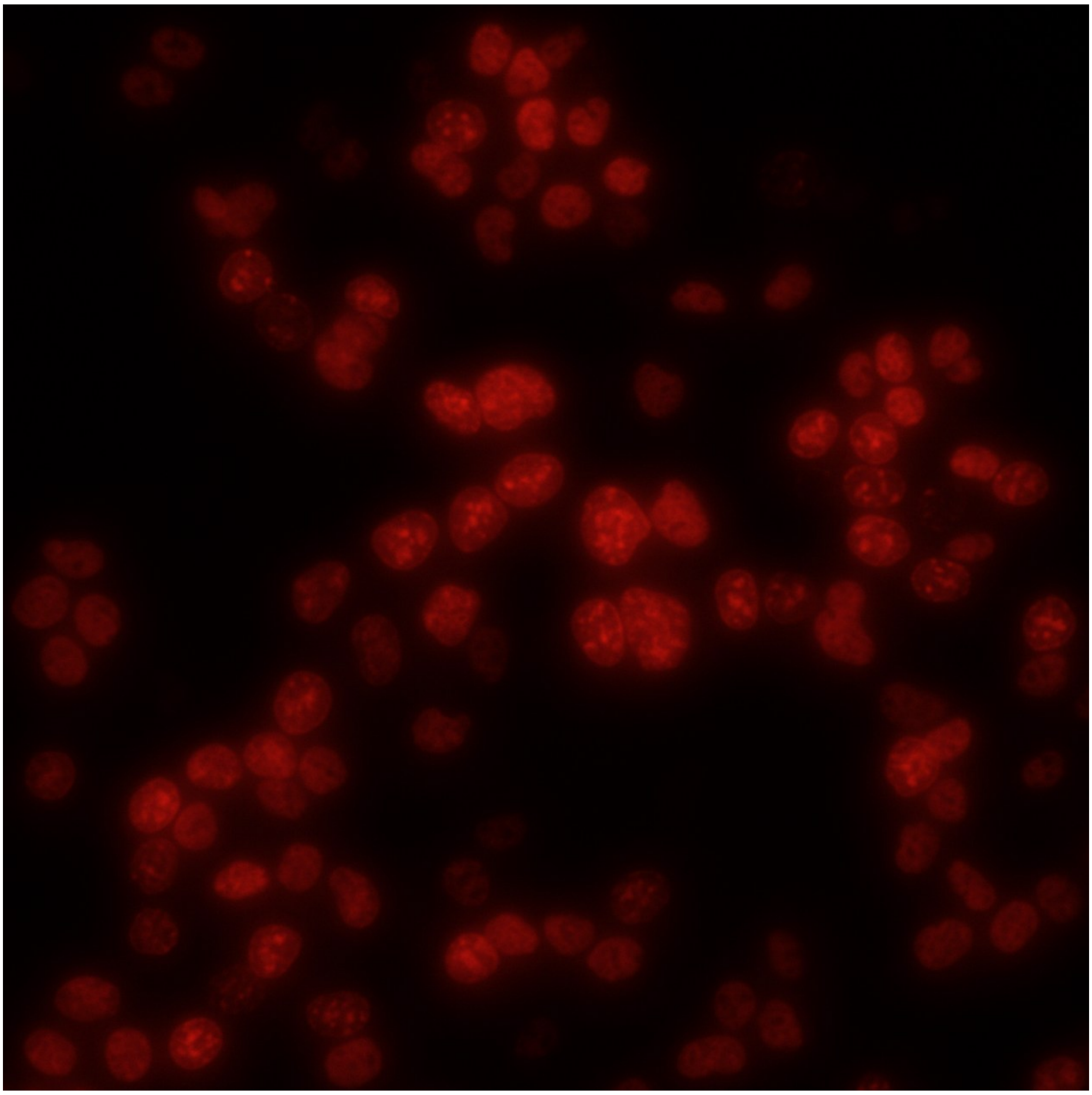

Fig.S3A-HEC-1-A-IGF2BP3-OE+sh-NC+EdU

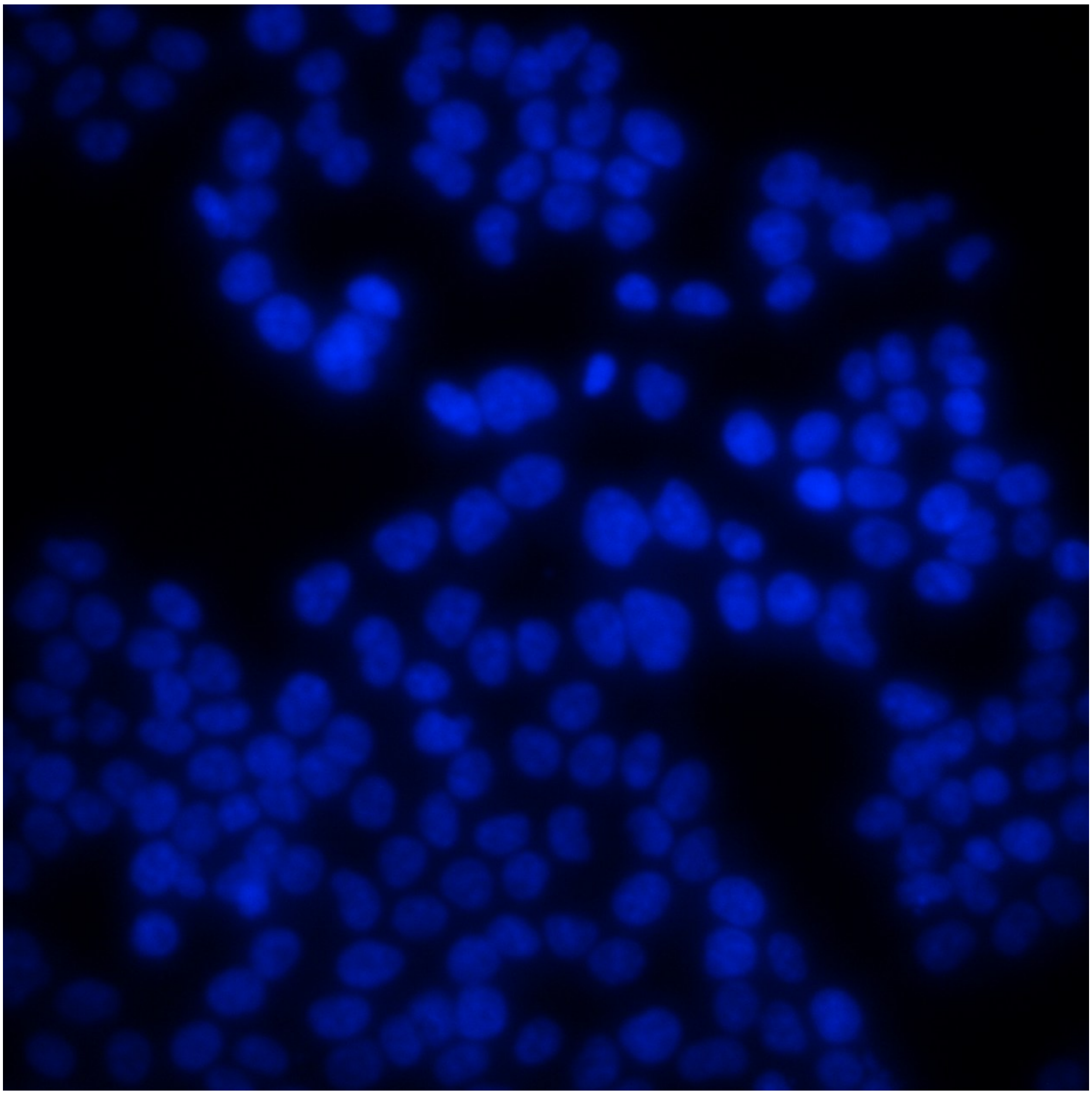

Fig.S3A-HEC-1-A-IGF2BP3-OE+sh-NC-Hoechst

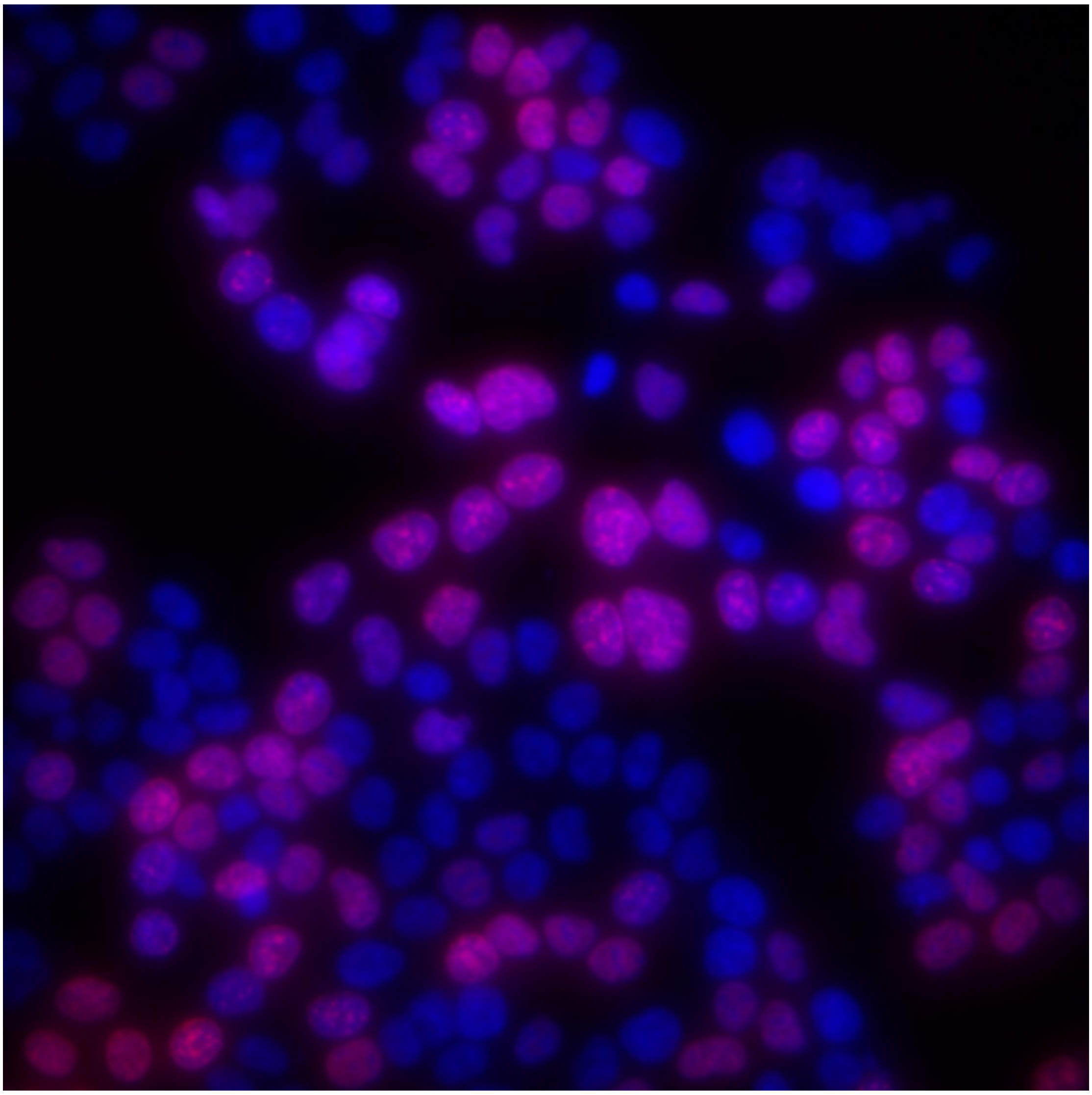

Fig.S3A-HEC-1-A-IGF2BP3-OE+sh-NC-Merge

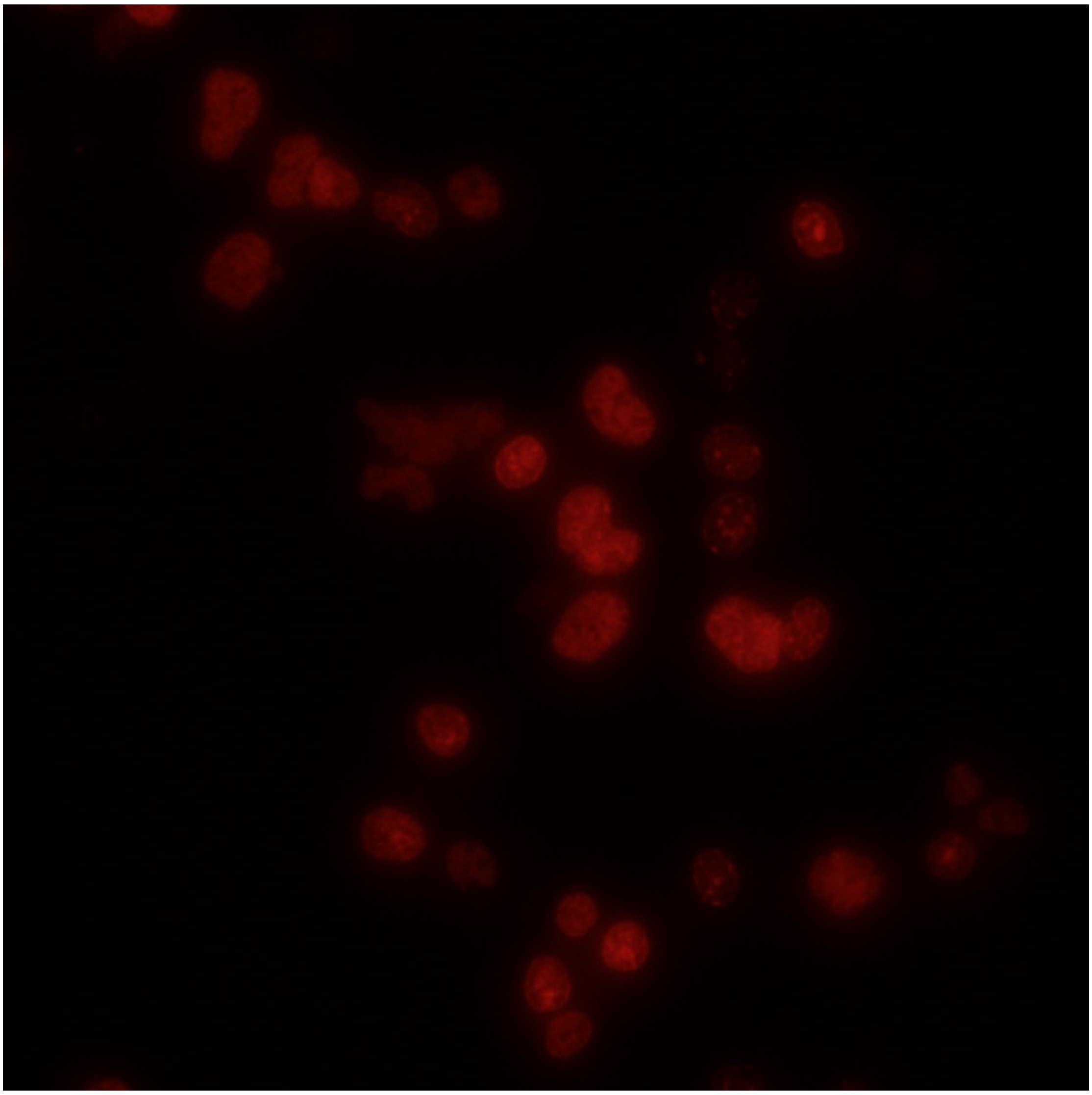

Fig.S3A-HEC-1-A-LV-NC+sh-LINC00958-EdU

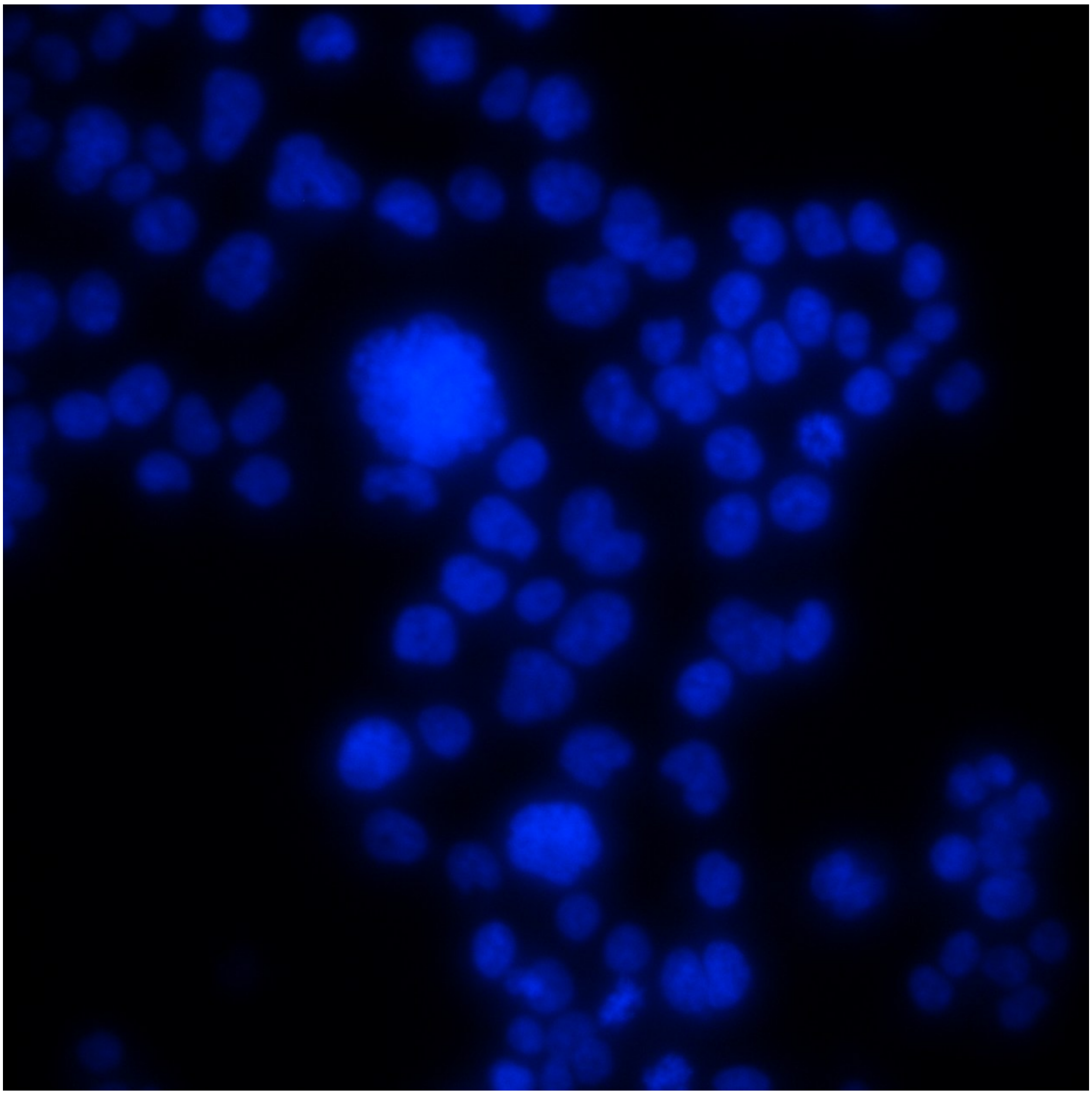

Fig.S3A-HEC-1-A-LV-NC+sh-LINC00958-Hoechst

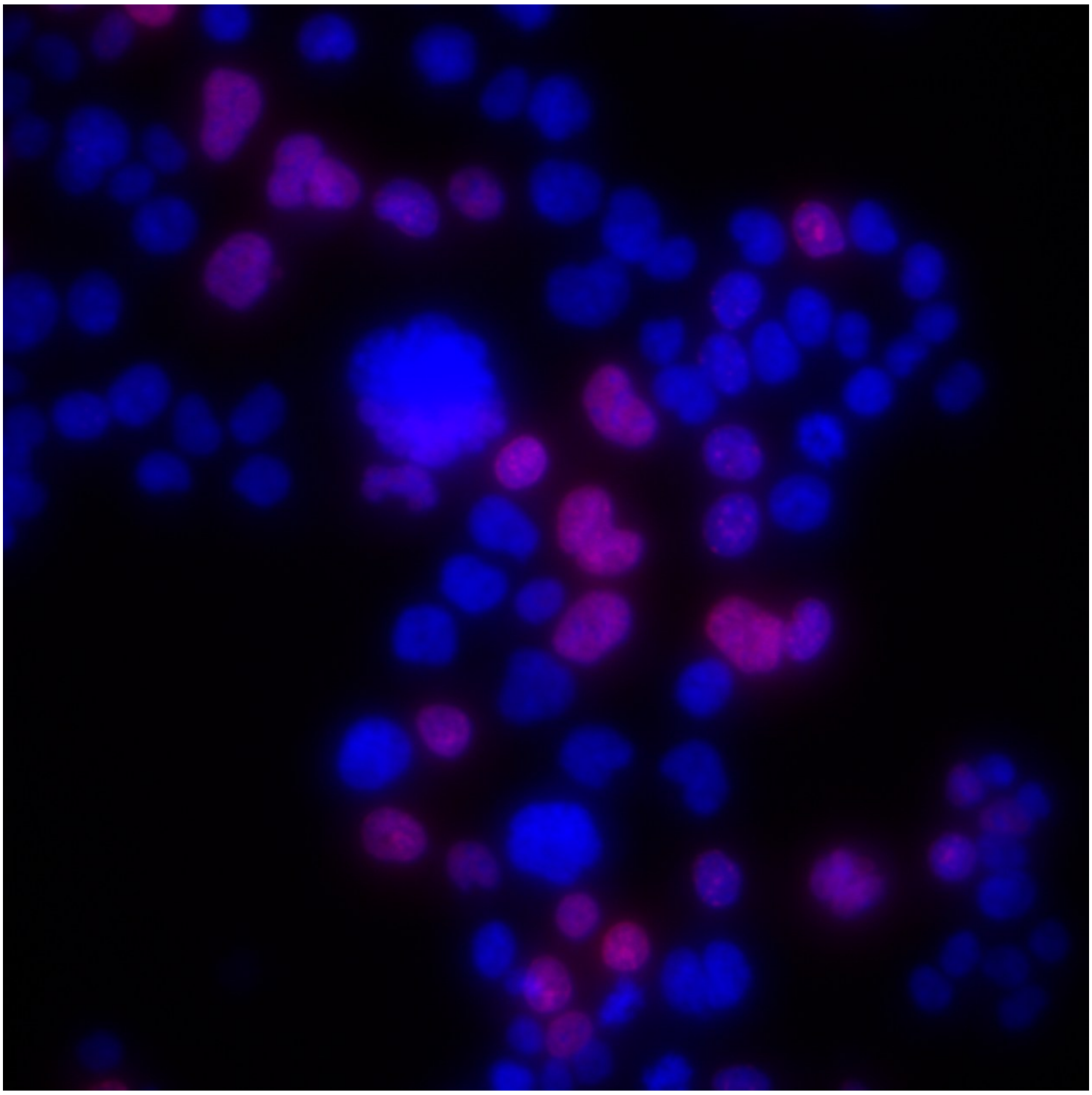

Fig.S3A-HEC-1-A-LV-NC+sh-LINC00958-Merge

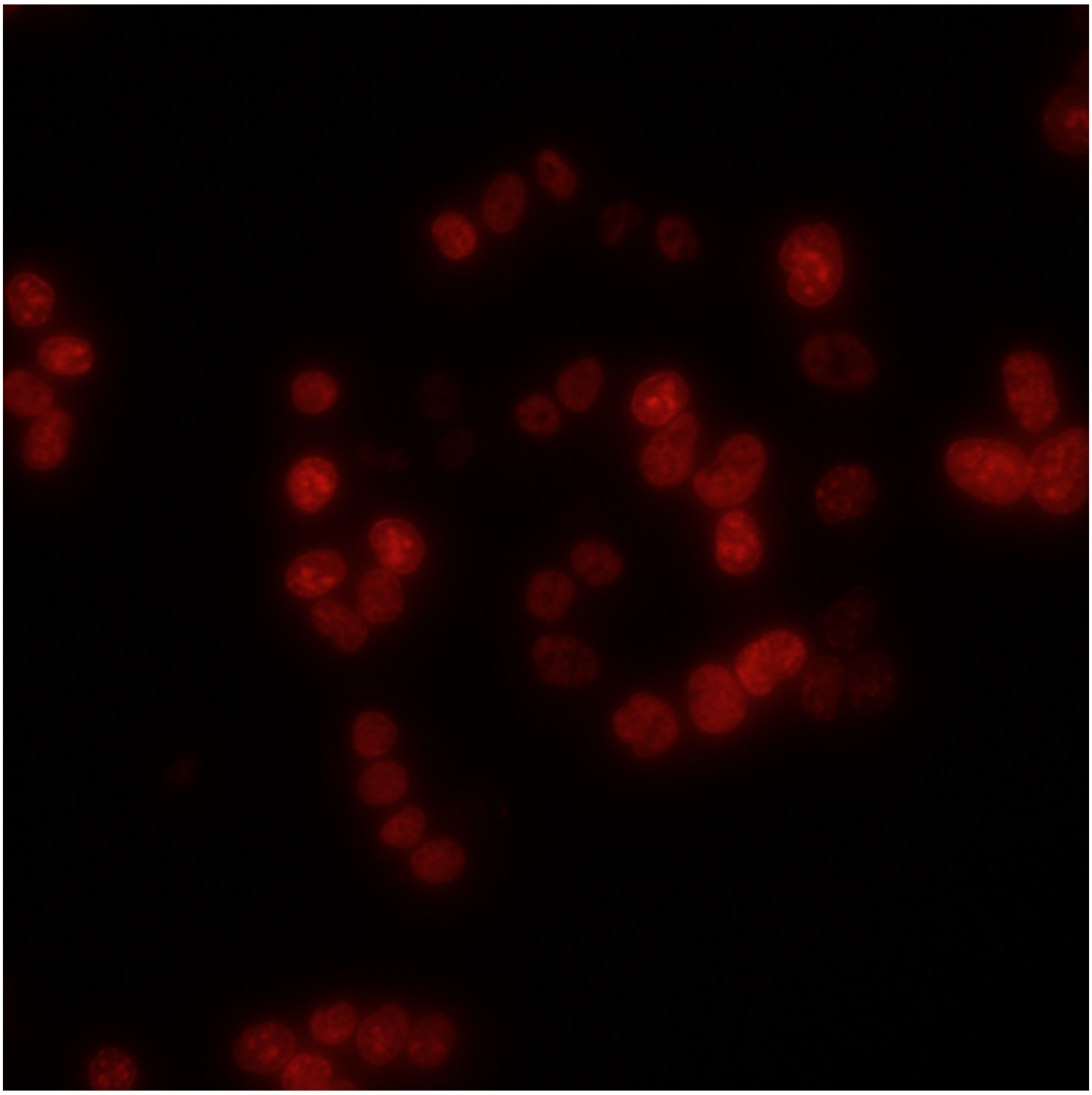

Fig.S3A-HEC-1-A-LV-NC+sh-NC-EdU

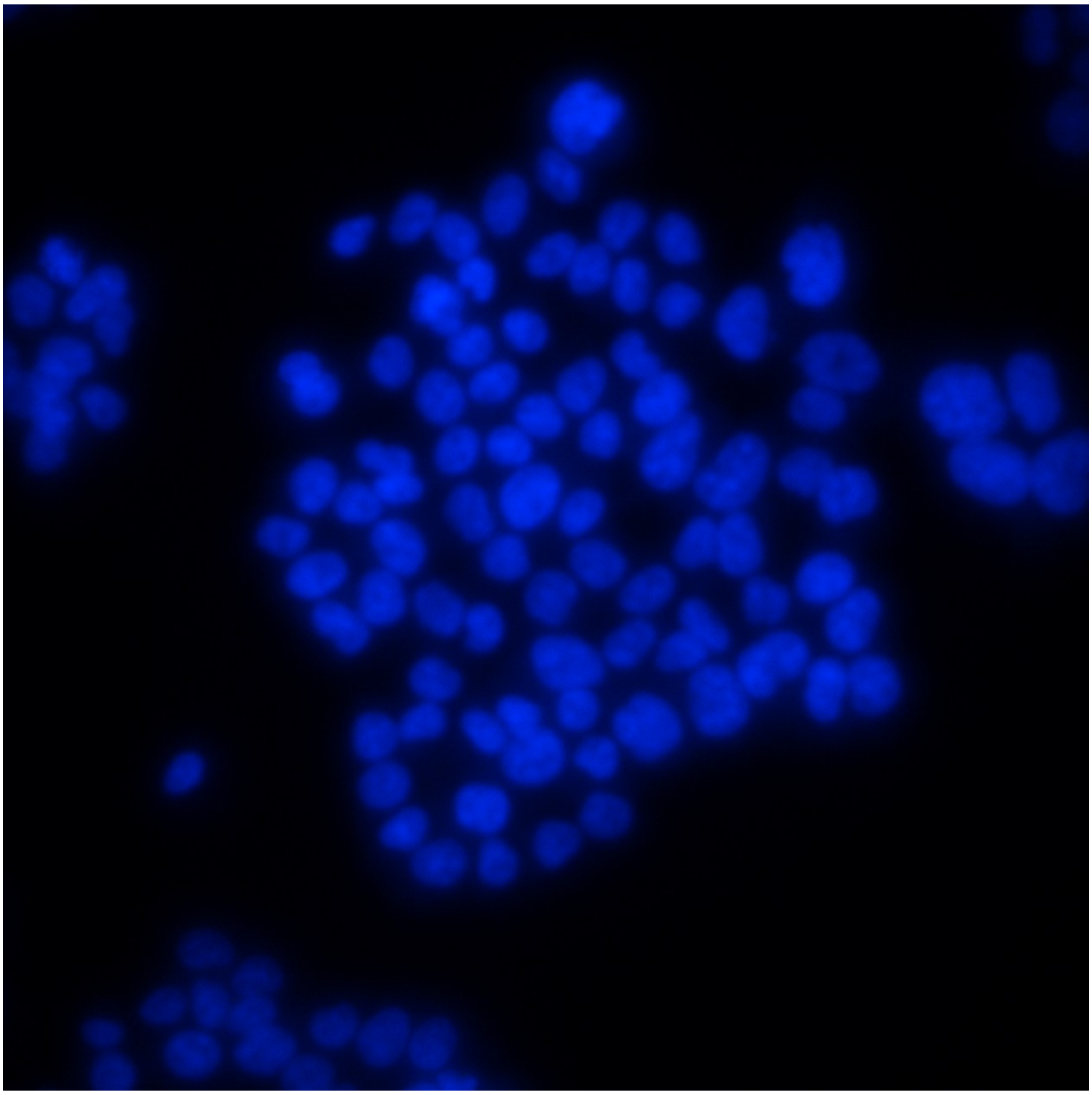

Fig.S3A-HEC-1-A-LV-NC+sh-NC-Hoechst

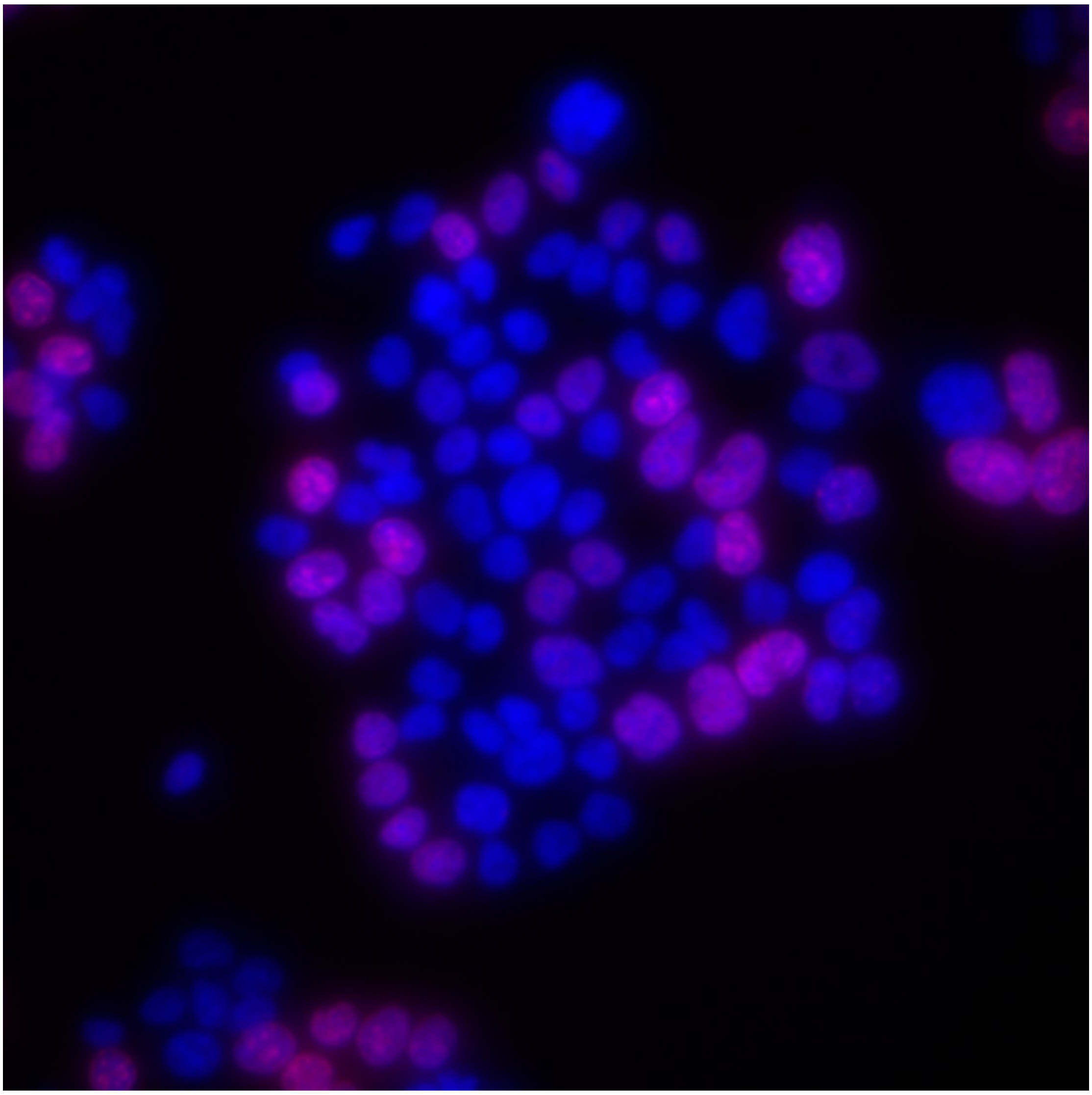

Fig.S3A-HEC-1-A-LV-NC+sh-NC-Merge

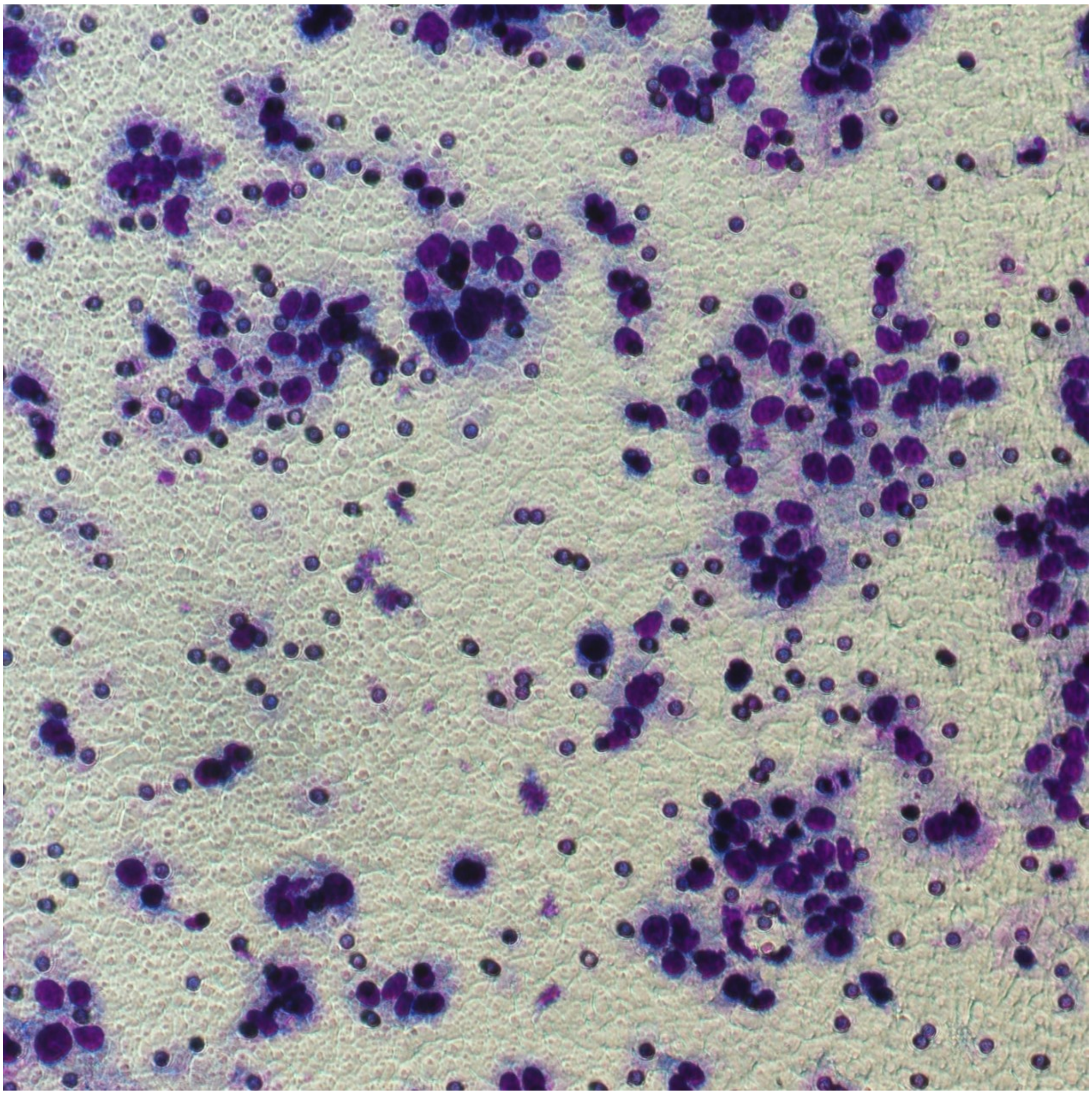

Fig.S3B-HEC-1-A-Invasion-IGF2BP3-OE+sh-LINC00958

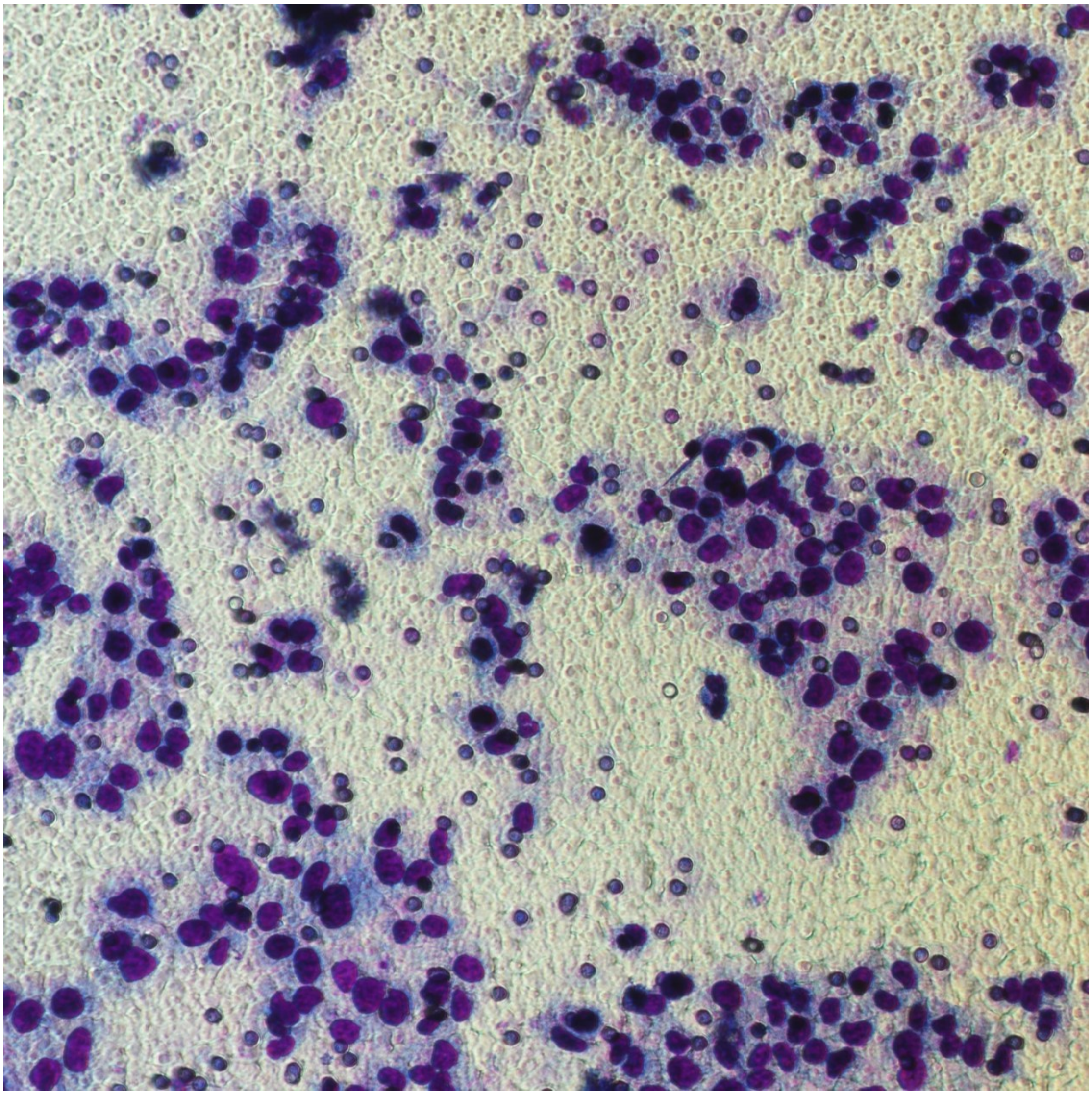

Fig.S3B-HEC-1-A-Invasion-IGF2BP3-OE+sh-NC

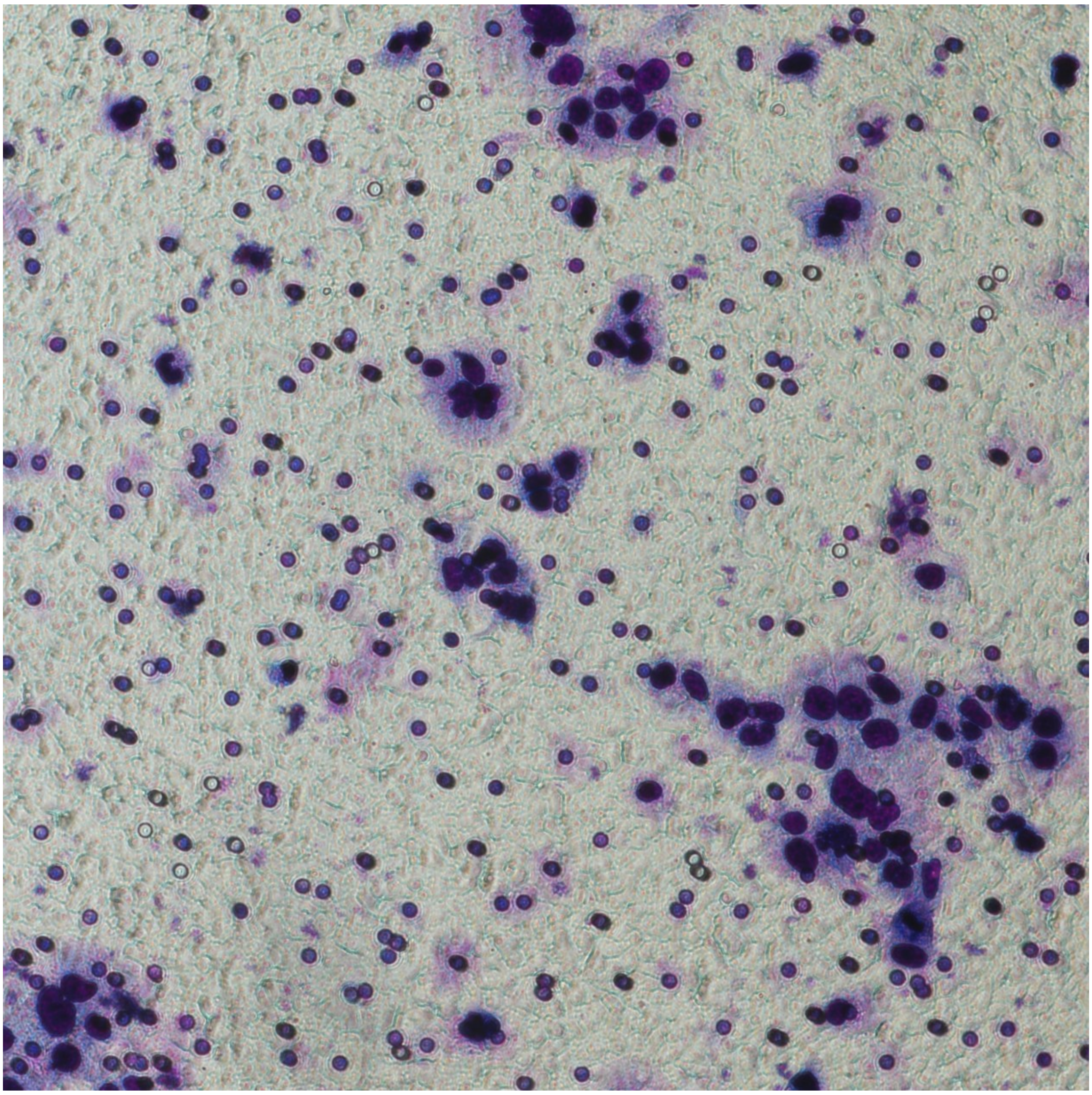

Fig.S3B-HEC-1-A-Invasion-LV-NC+sh-LINC00958

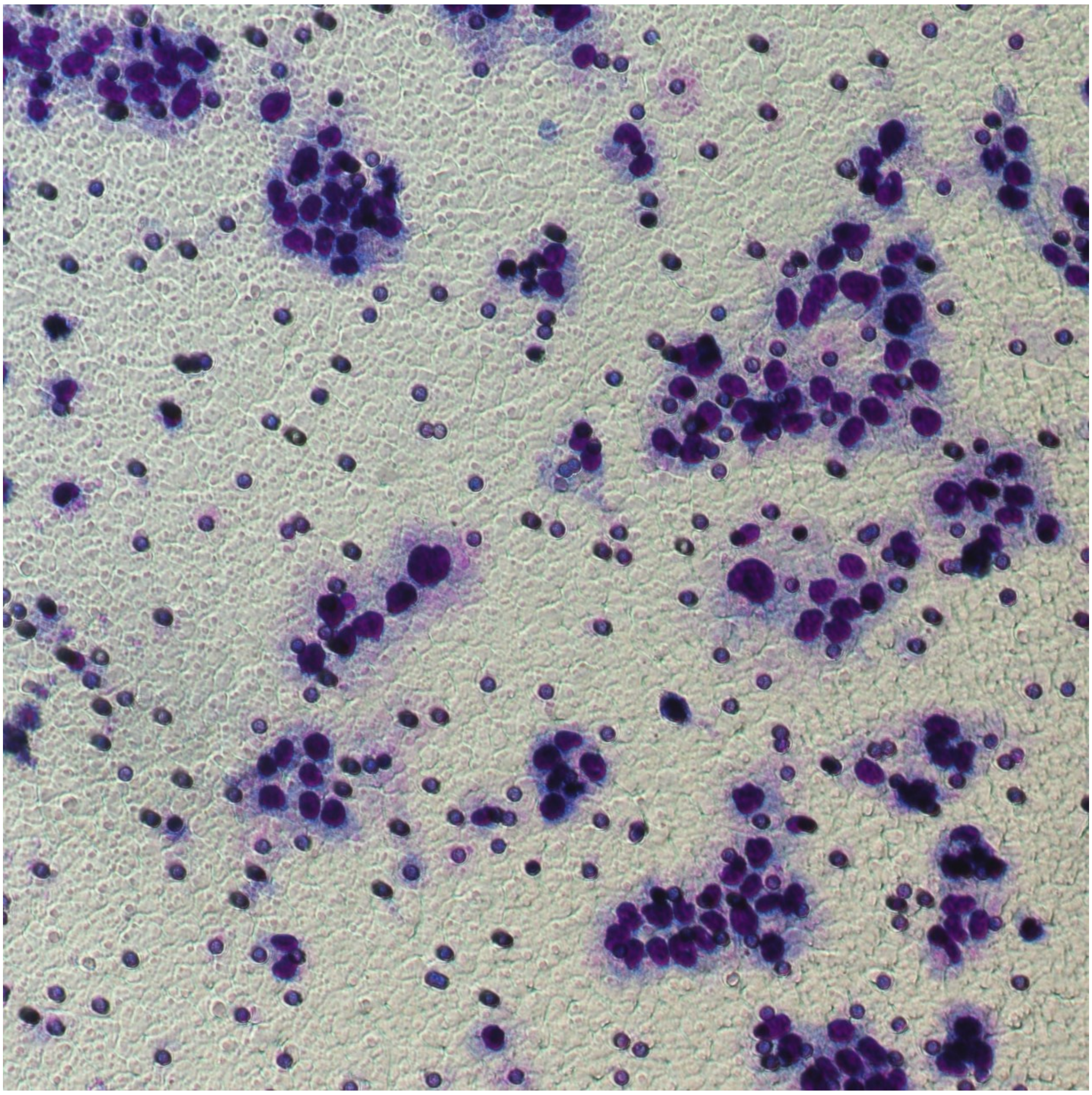

Fig.S3B-HEC-1-A-Invasion-LV-NC+sh-NC

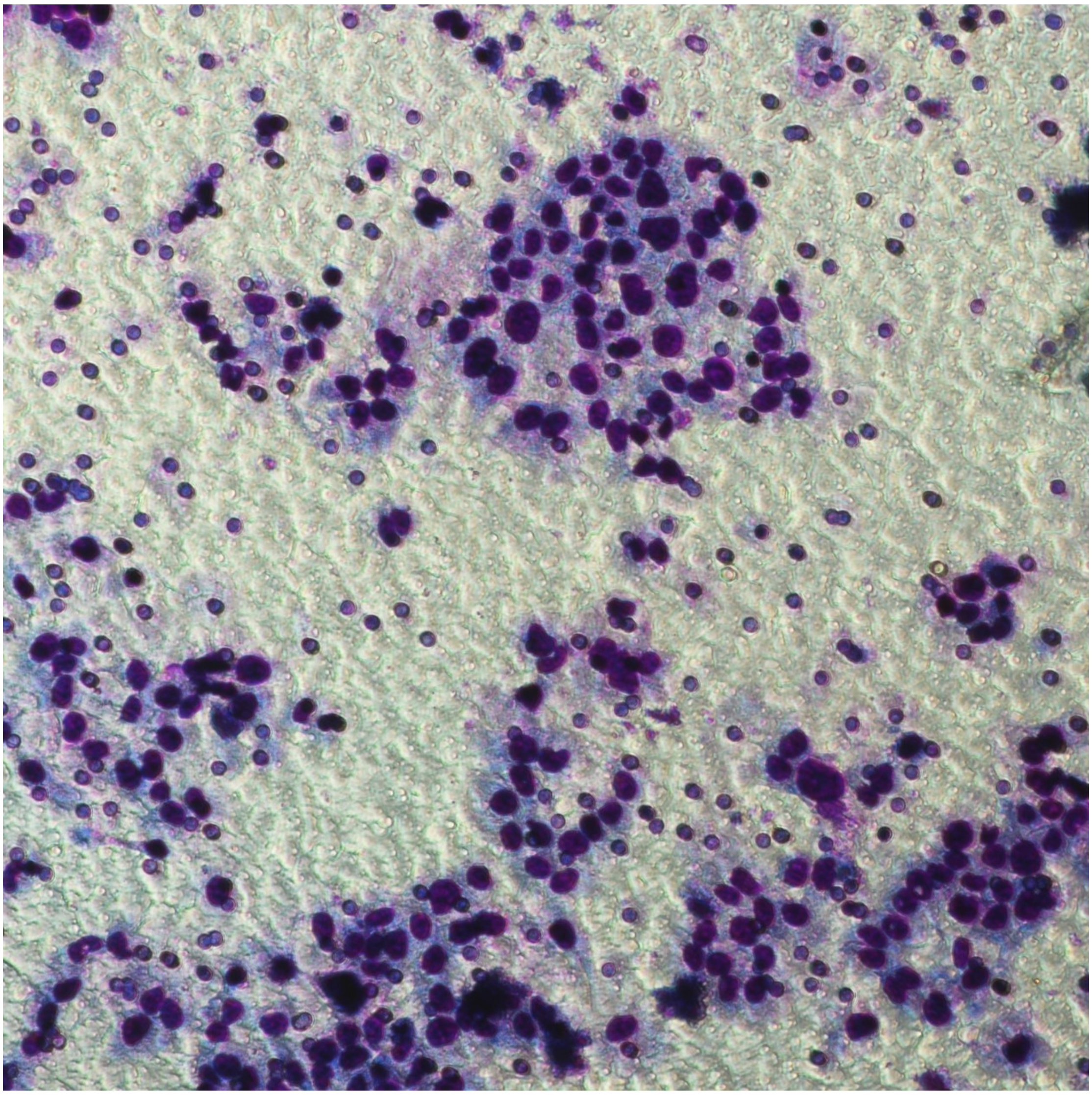

Fig.S3B-HEC-1-A-Migration-IGF2BP3-OE+sh-LINC00958

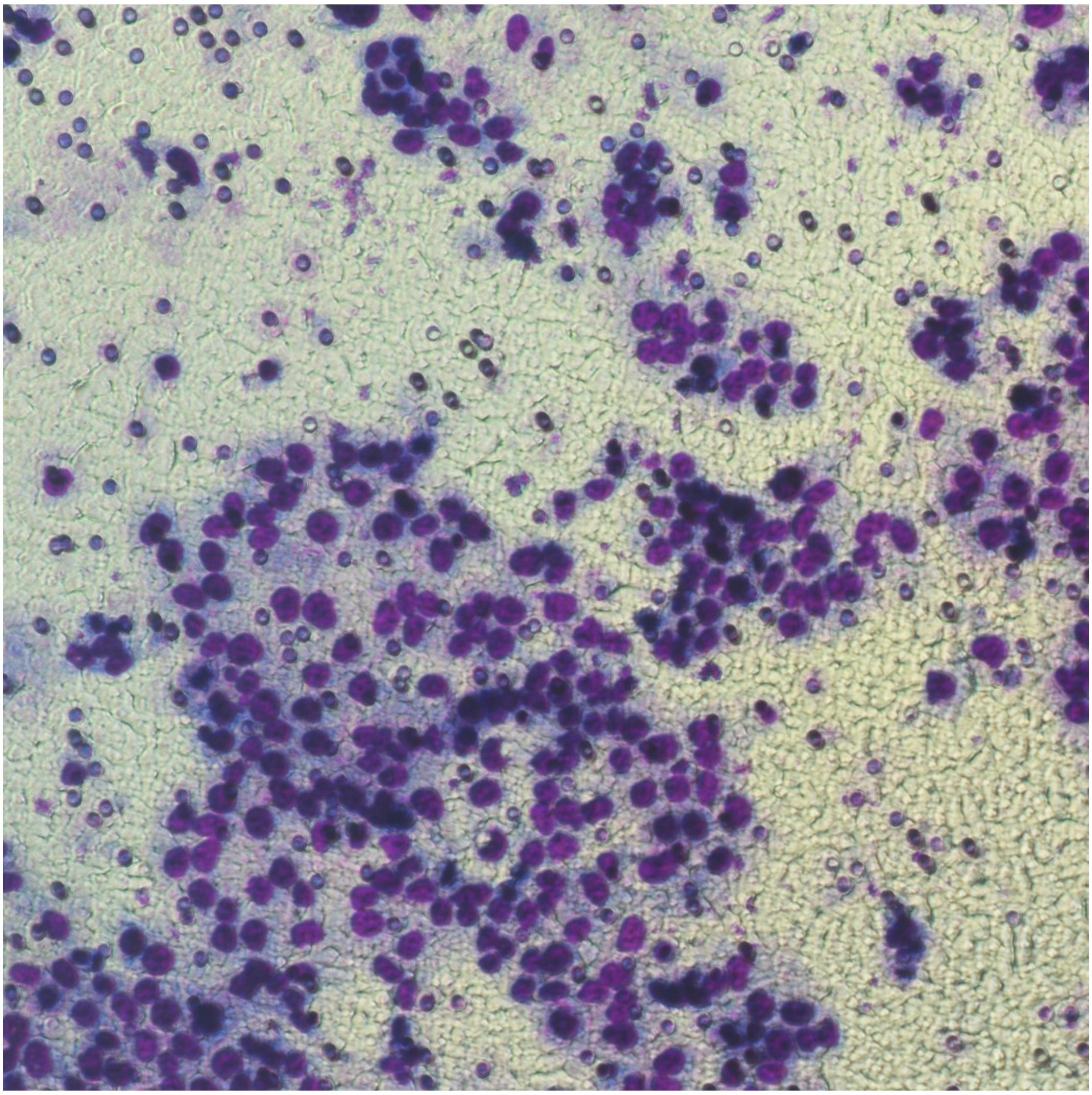

Fig.S3B-HEC-1-A-Migration-IGF2BP3-OE+sh-NC

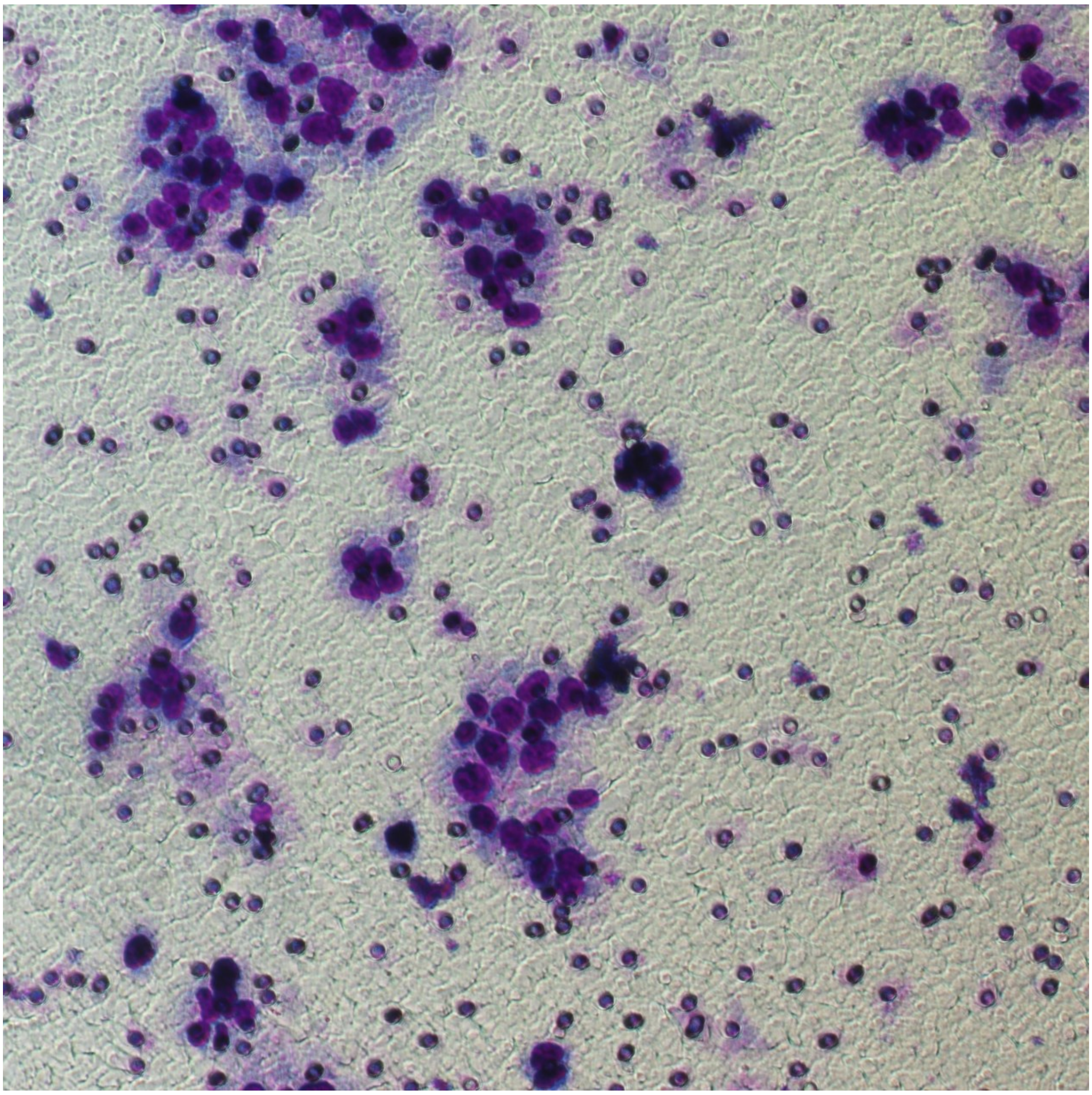

Fig.S3B-HEC-1-A-Migration-LV-NC+sh-LINC00958

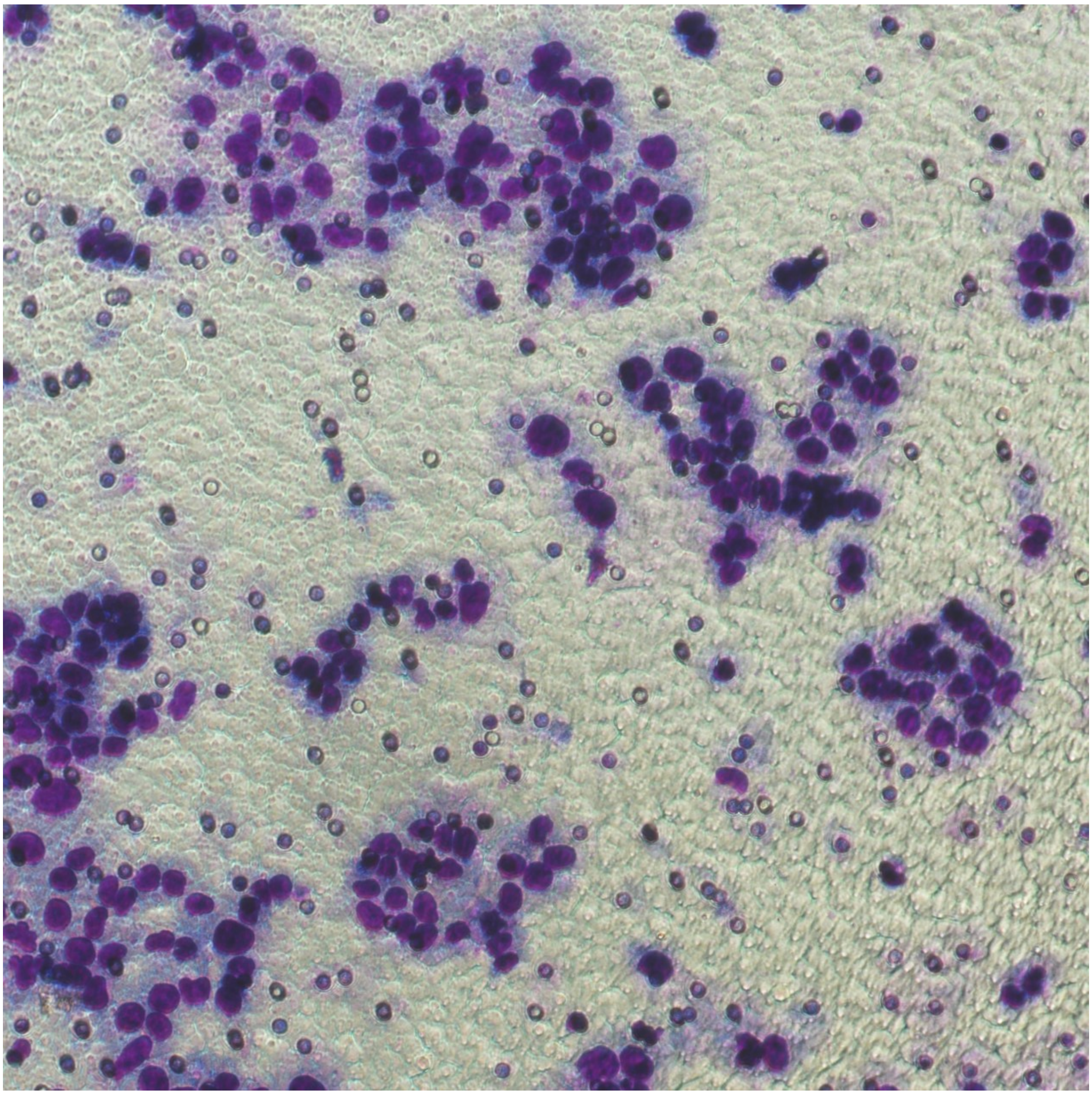

Fig.S3B-HEC-1-A-Migration-LV-NC+sh-NC

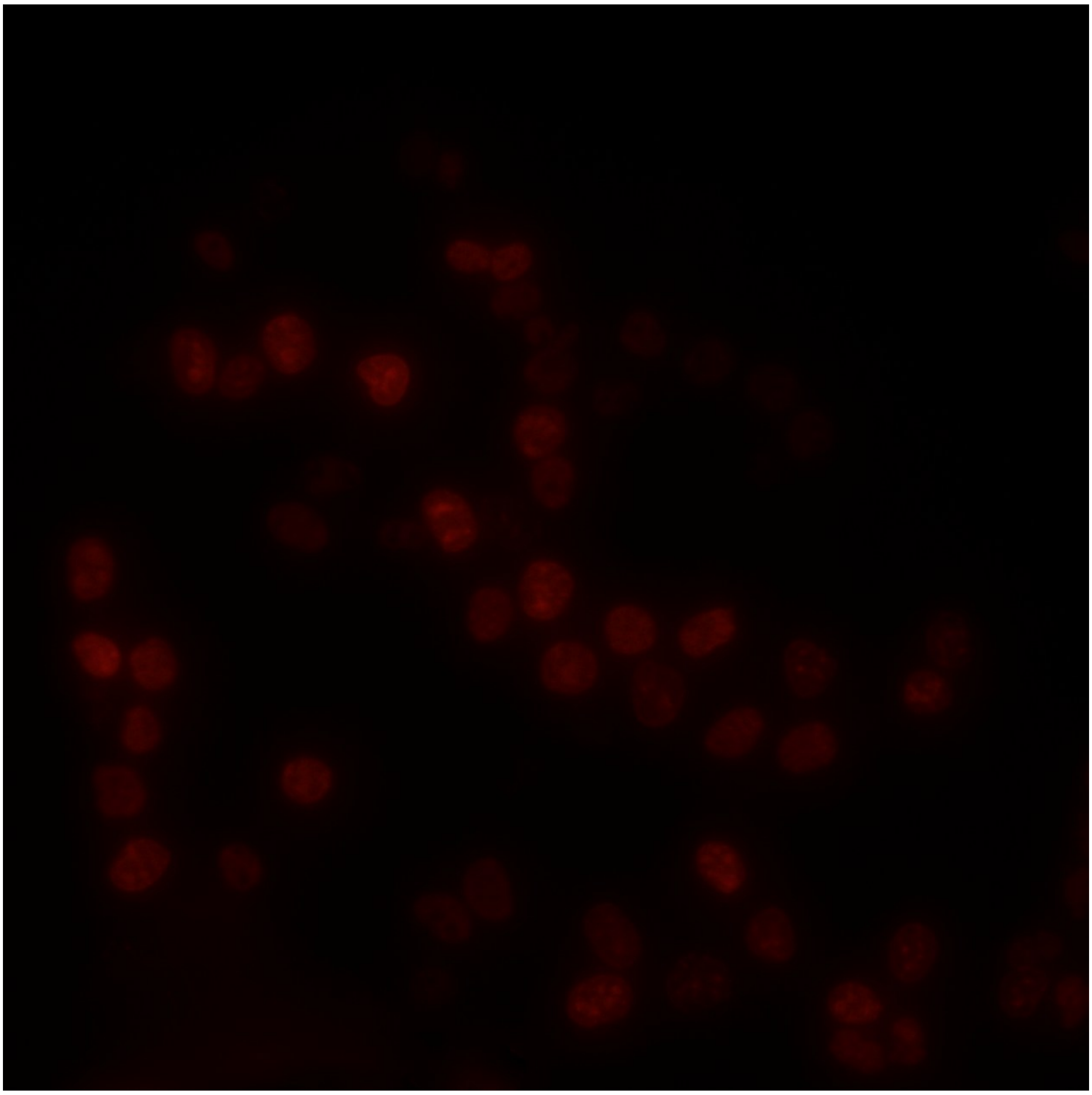

Fig.S4C-HEC-1-A-si-E2F3-1-EdU

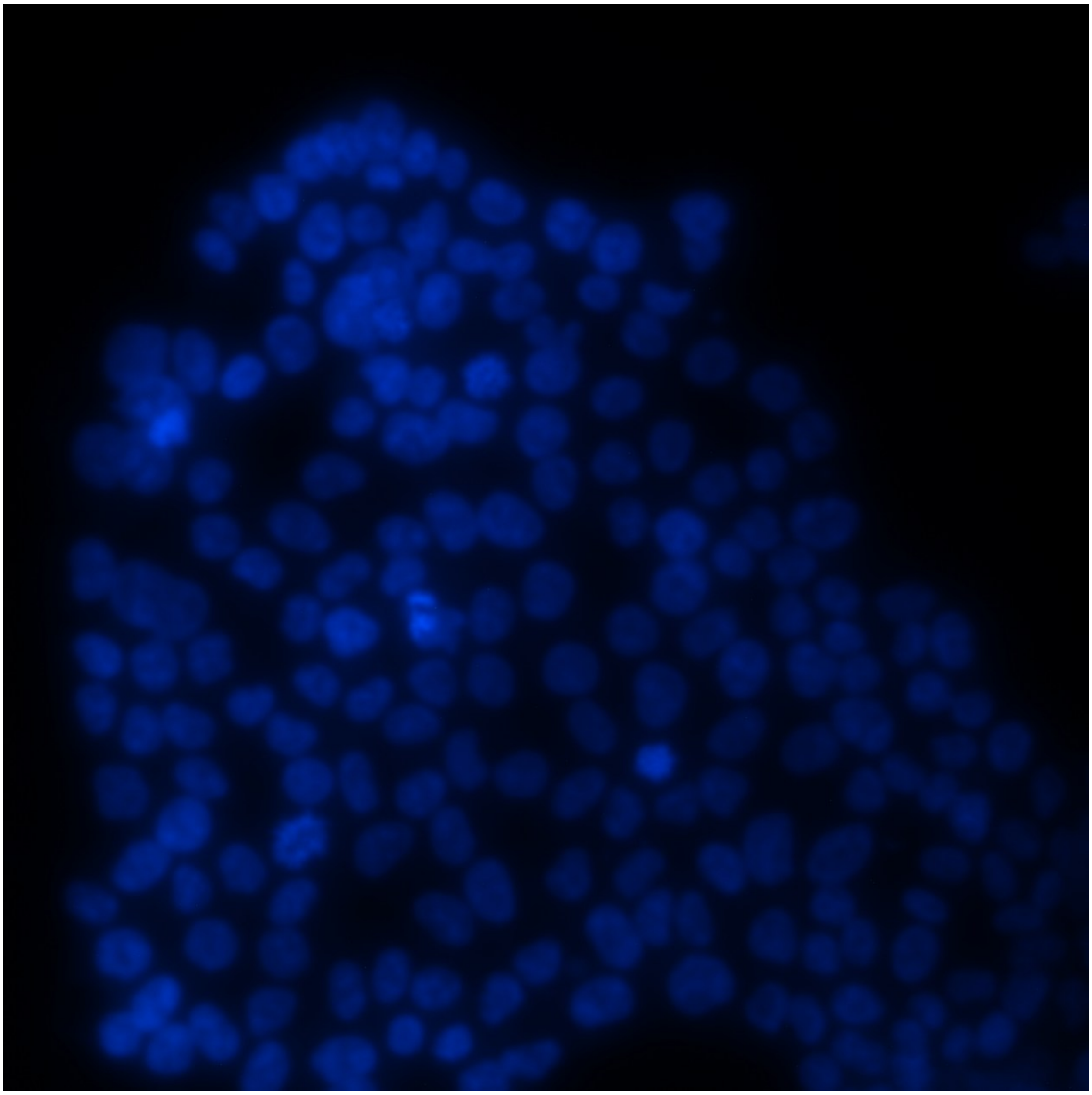

Fig.S4C-HEC-1-A-si-E2F3-1-Hoechest

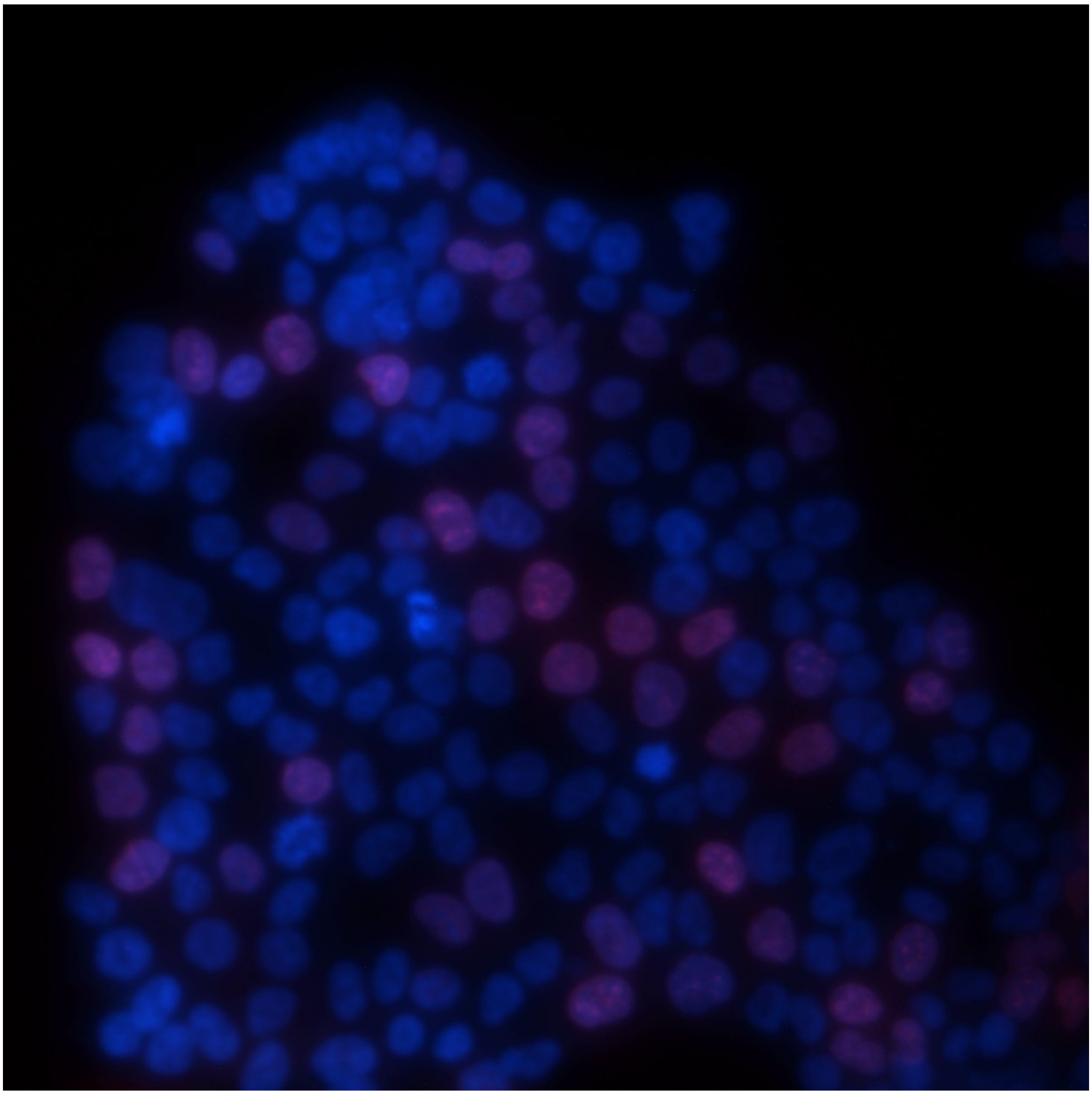

Fig.S4C-HEC-1-A-si-E2F3-1-merge

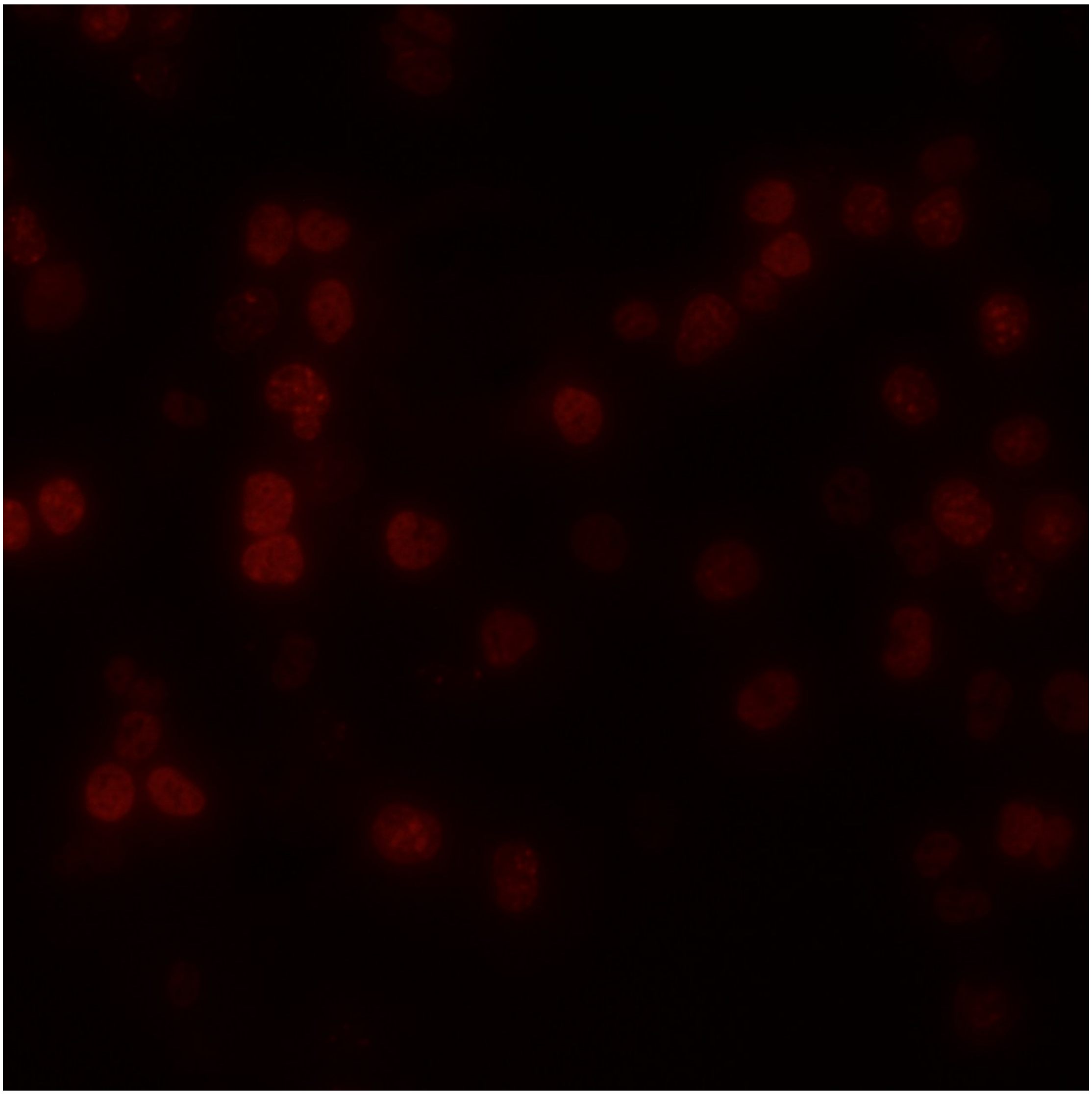

Fig.S4C-HEC-1-A-si-E2F3-2-EdU

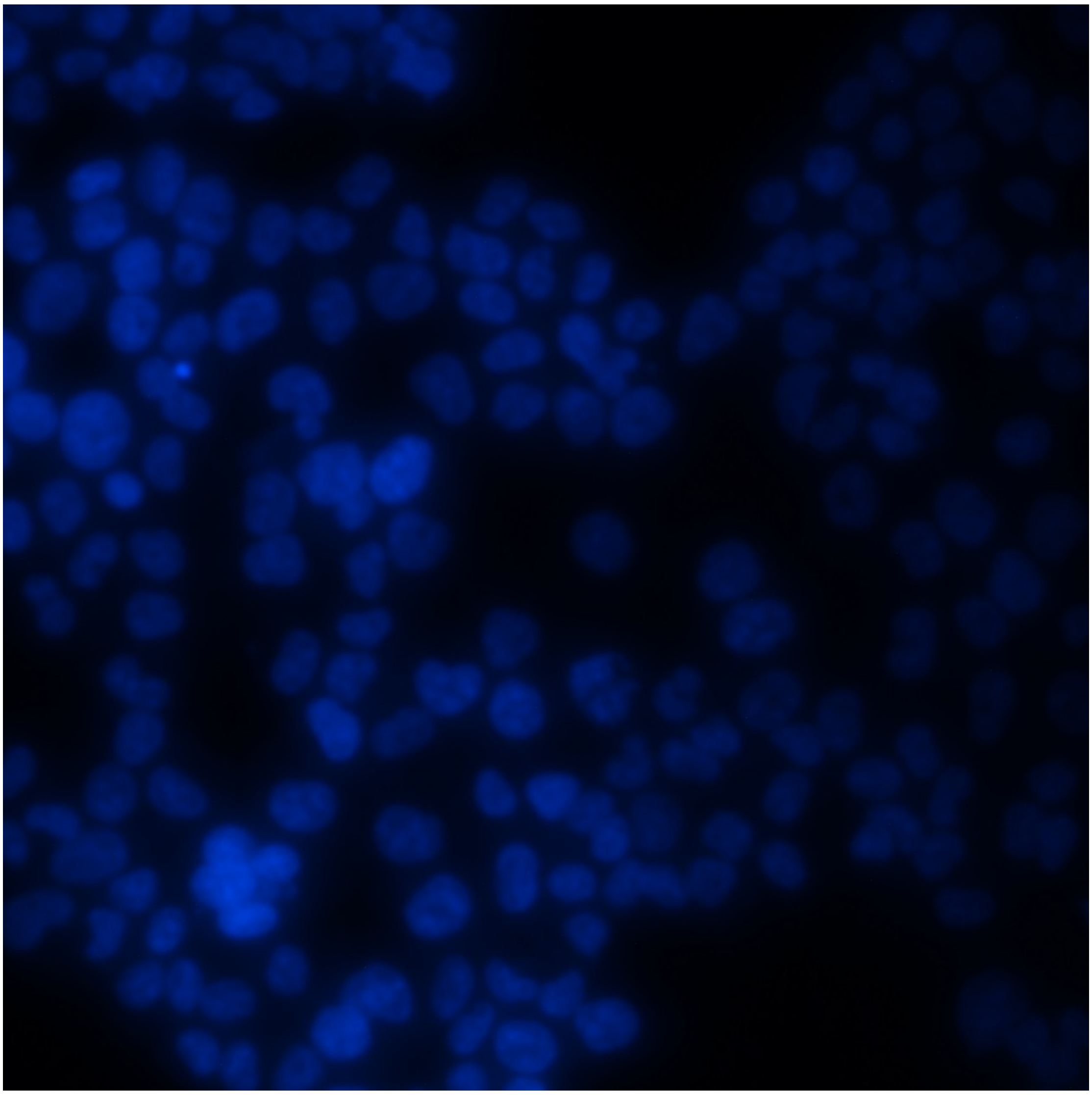

Fig.S4C-HEC-1-A-si-E2F3-2-Hoechest

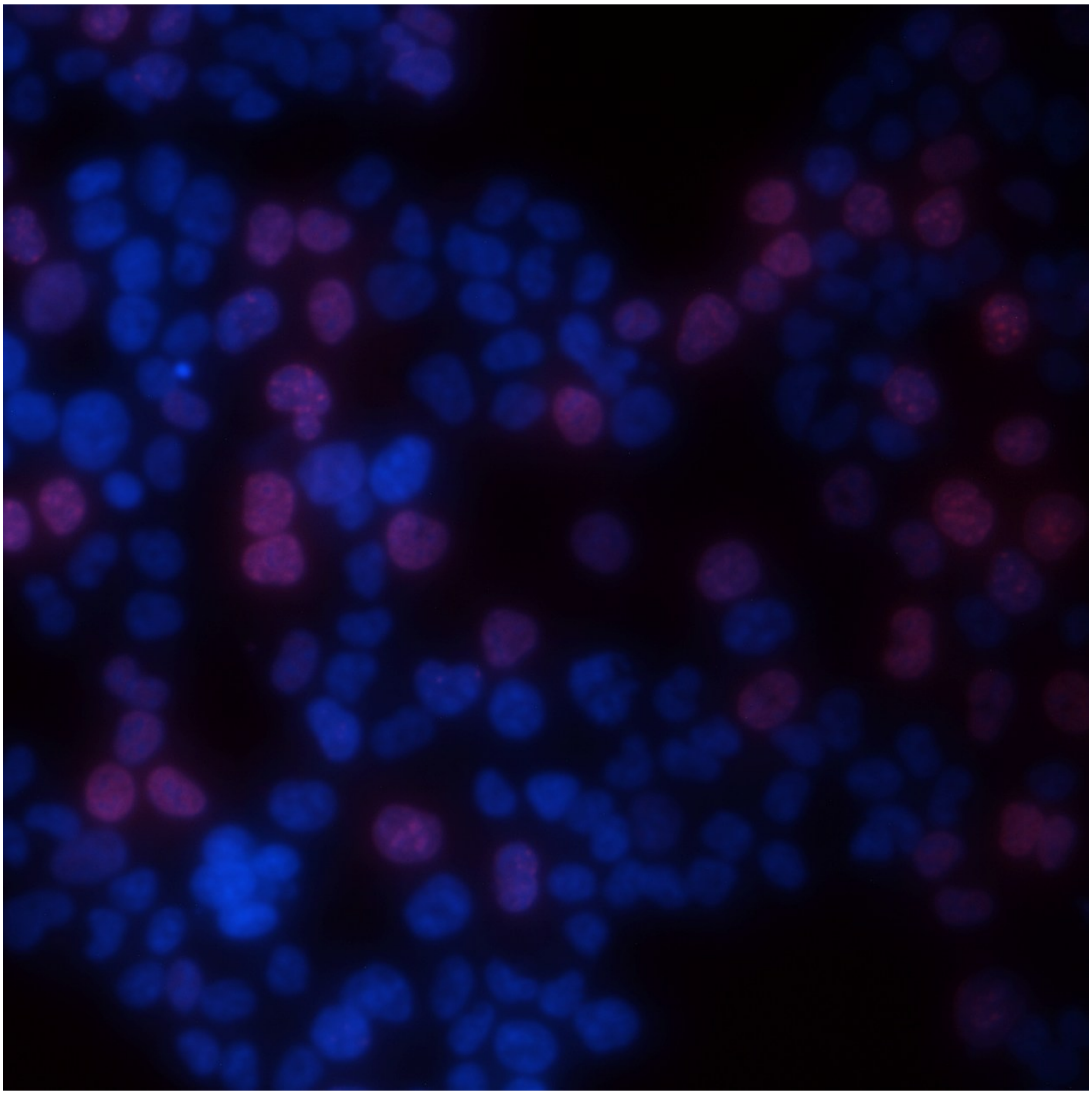

Fig.S4C-HEC-1-A-si-E2F3-2-merge

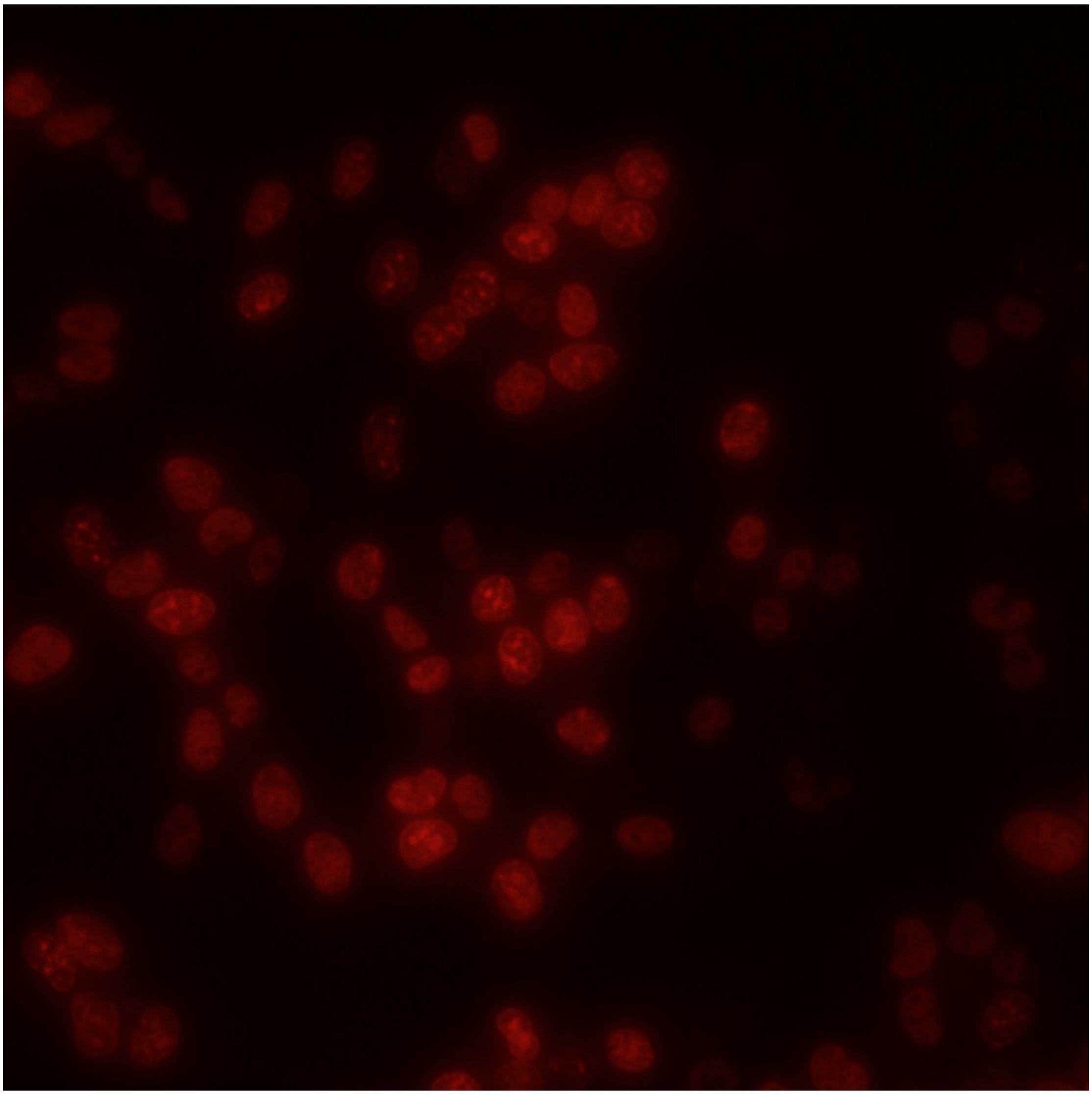

Fig.S4C-HEC-1-A-si-NC-EdU

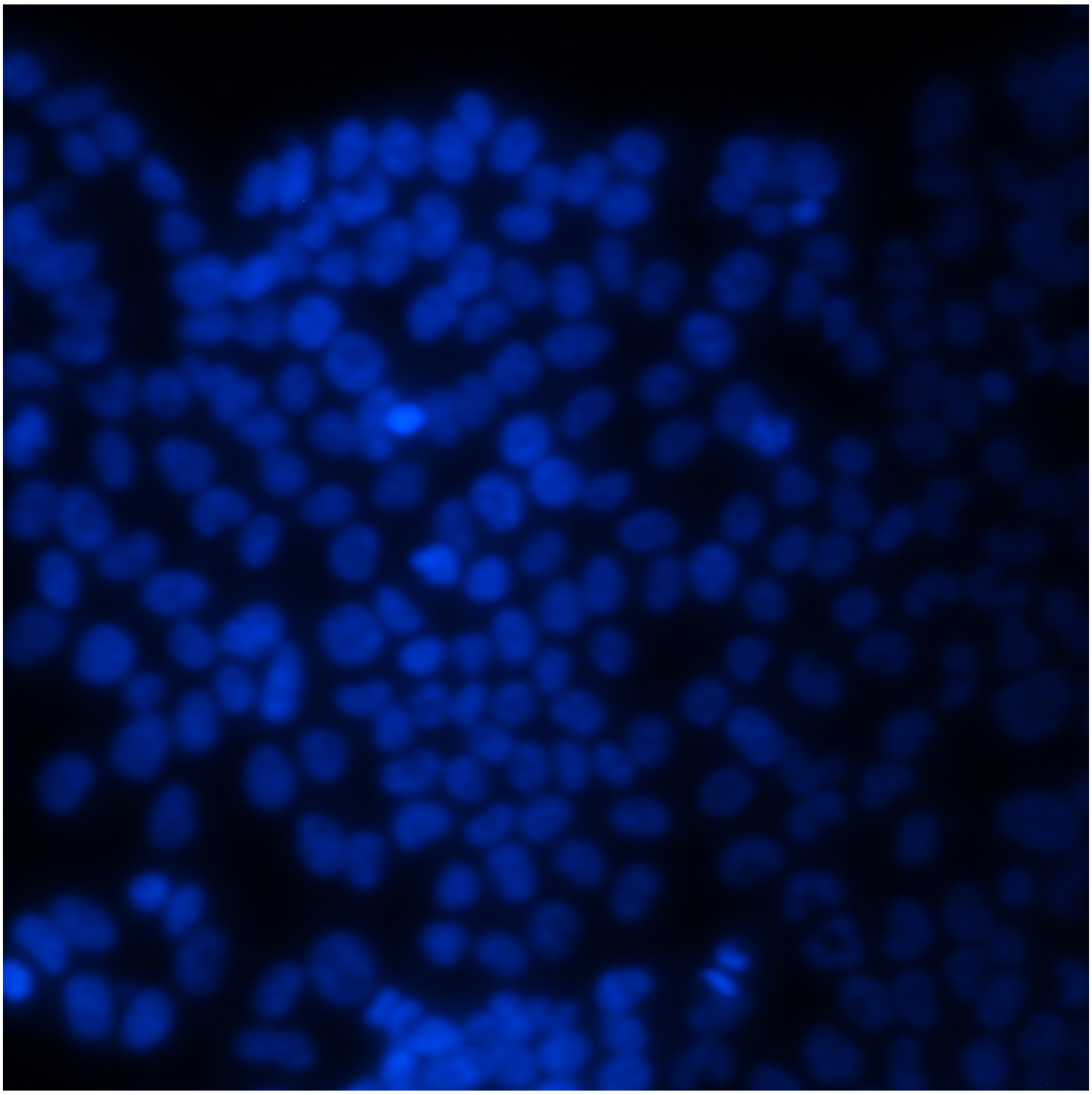

Fig.S4C-HEC-1-A-si-NC-Hoechest

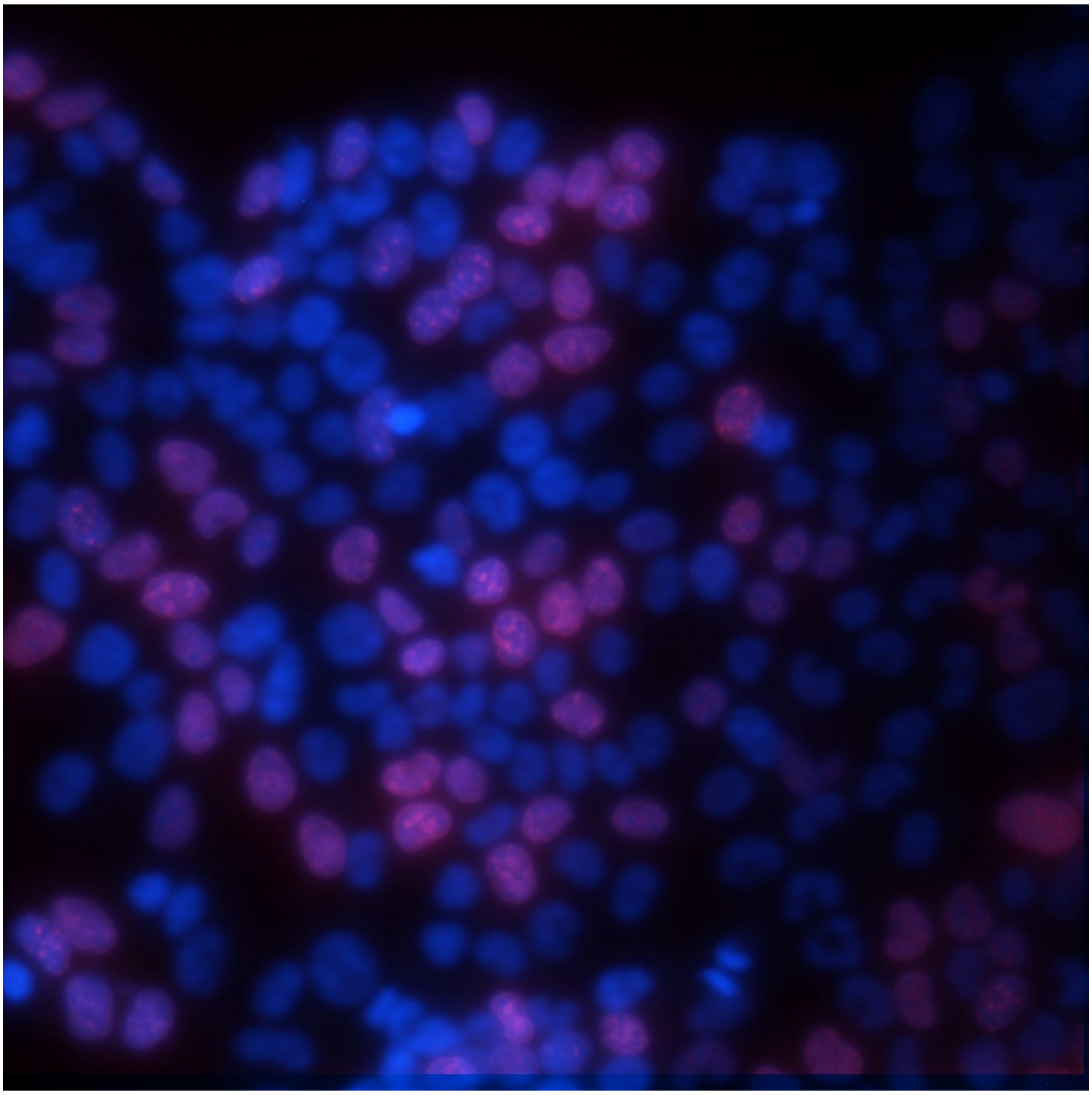

Fig.S4C-HEC-1-A-si-NC-merge

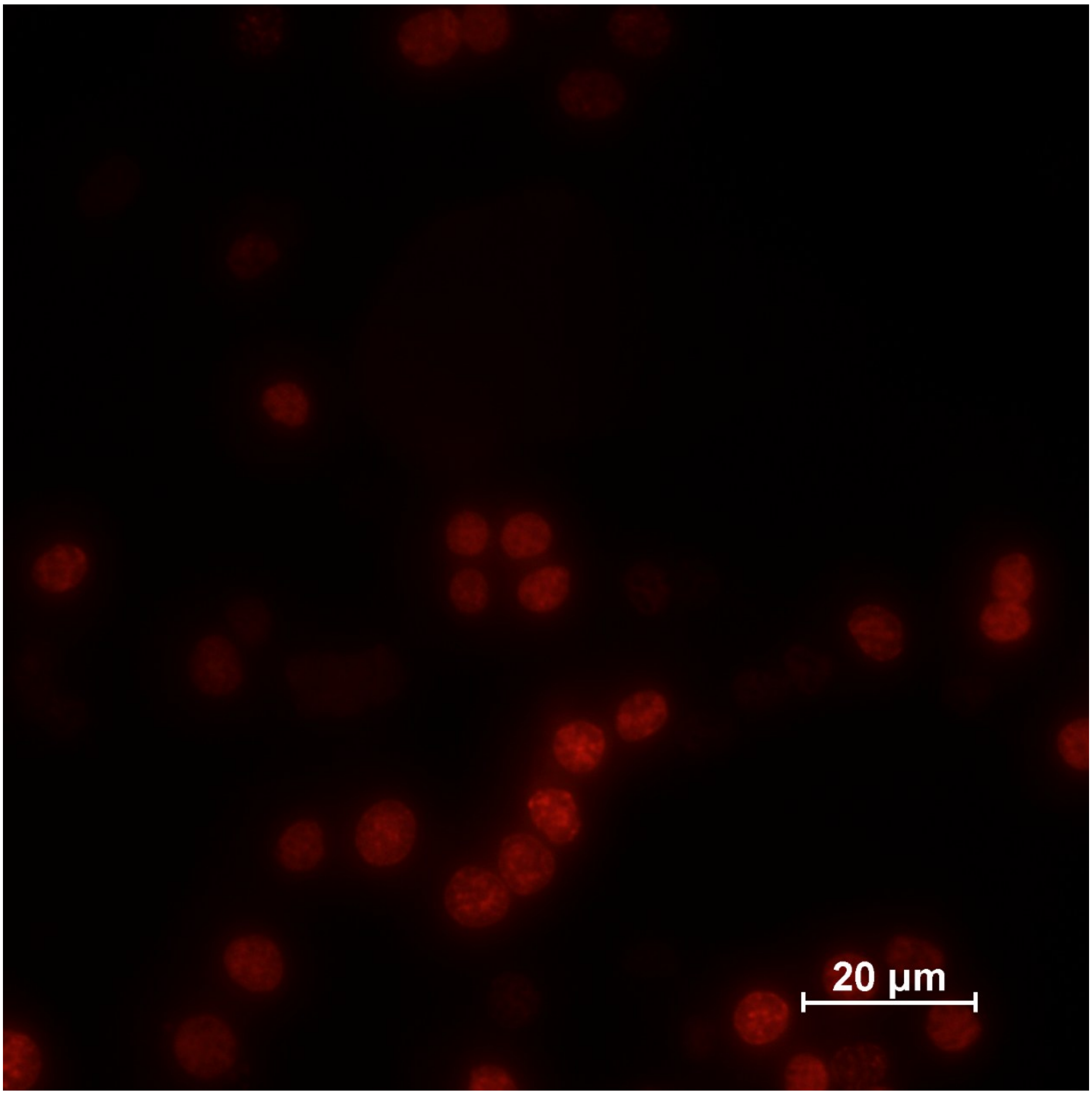

Fig.S4C-Ishikawa-si-E2F3-1-EdU

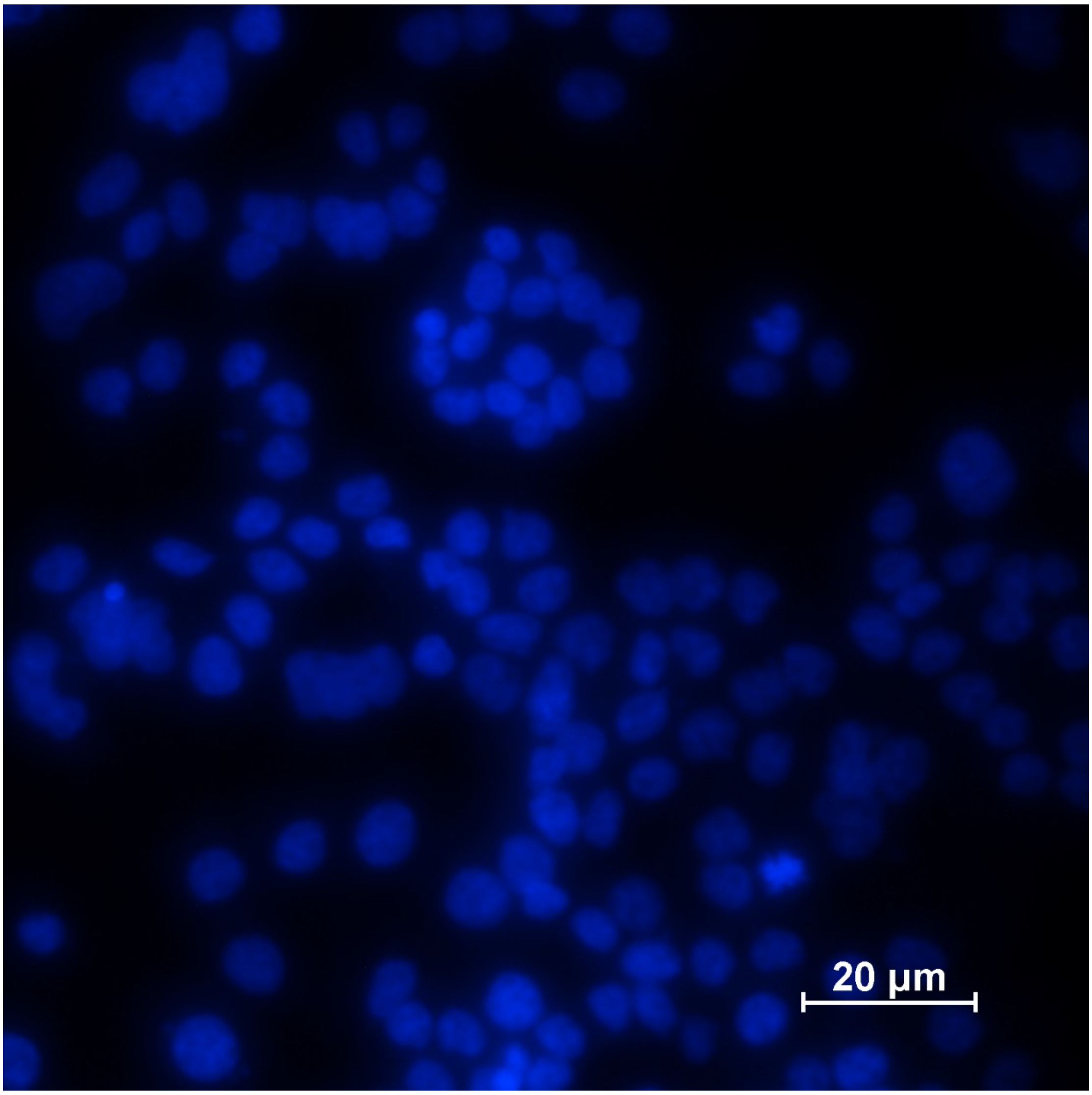

Fig.S4C-Ishikawa-si-E2F3-1-Hoechest

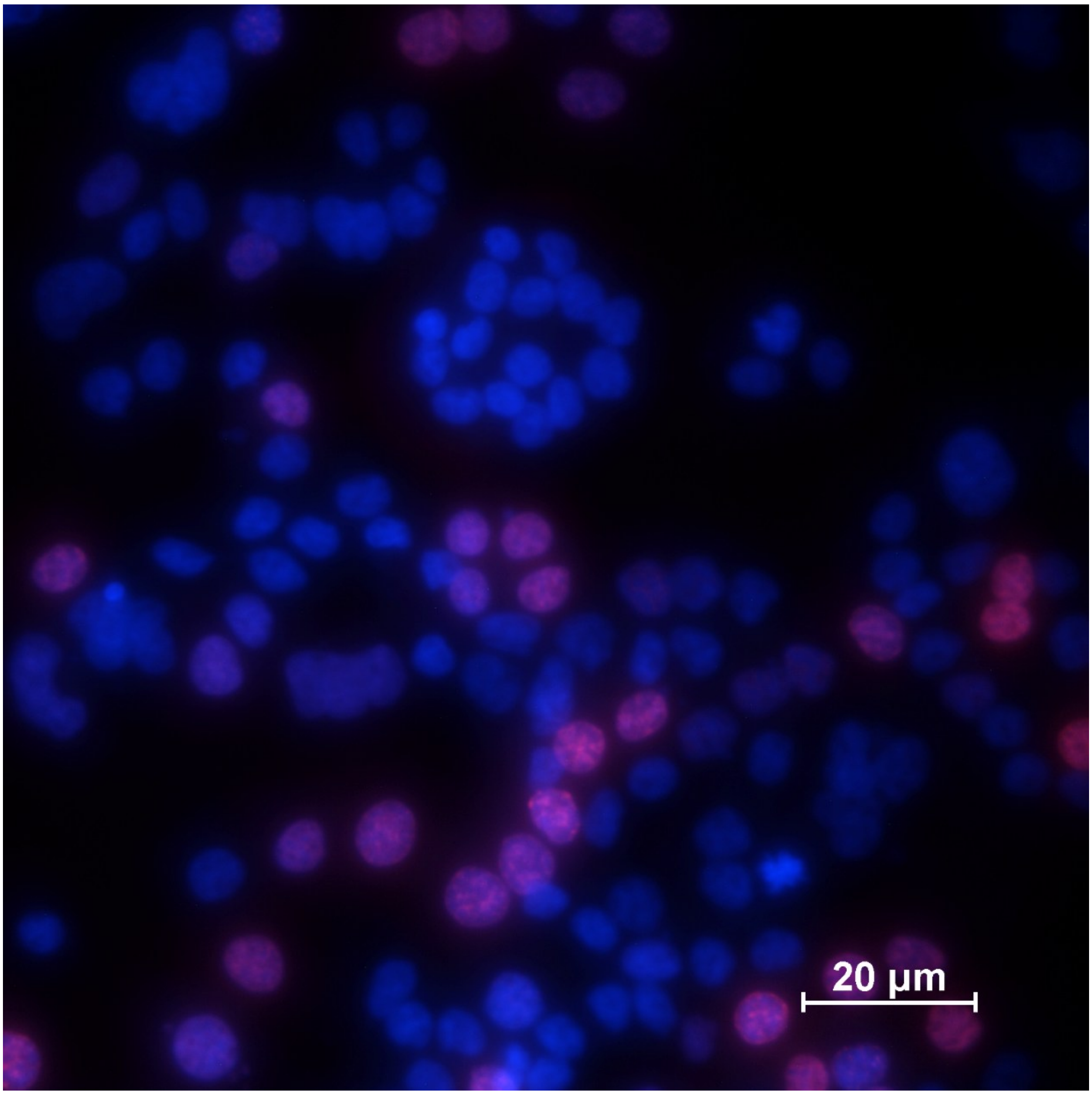

Fig.S4C-Ishikawa-si-E2F3-1-merge

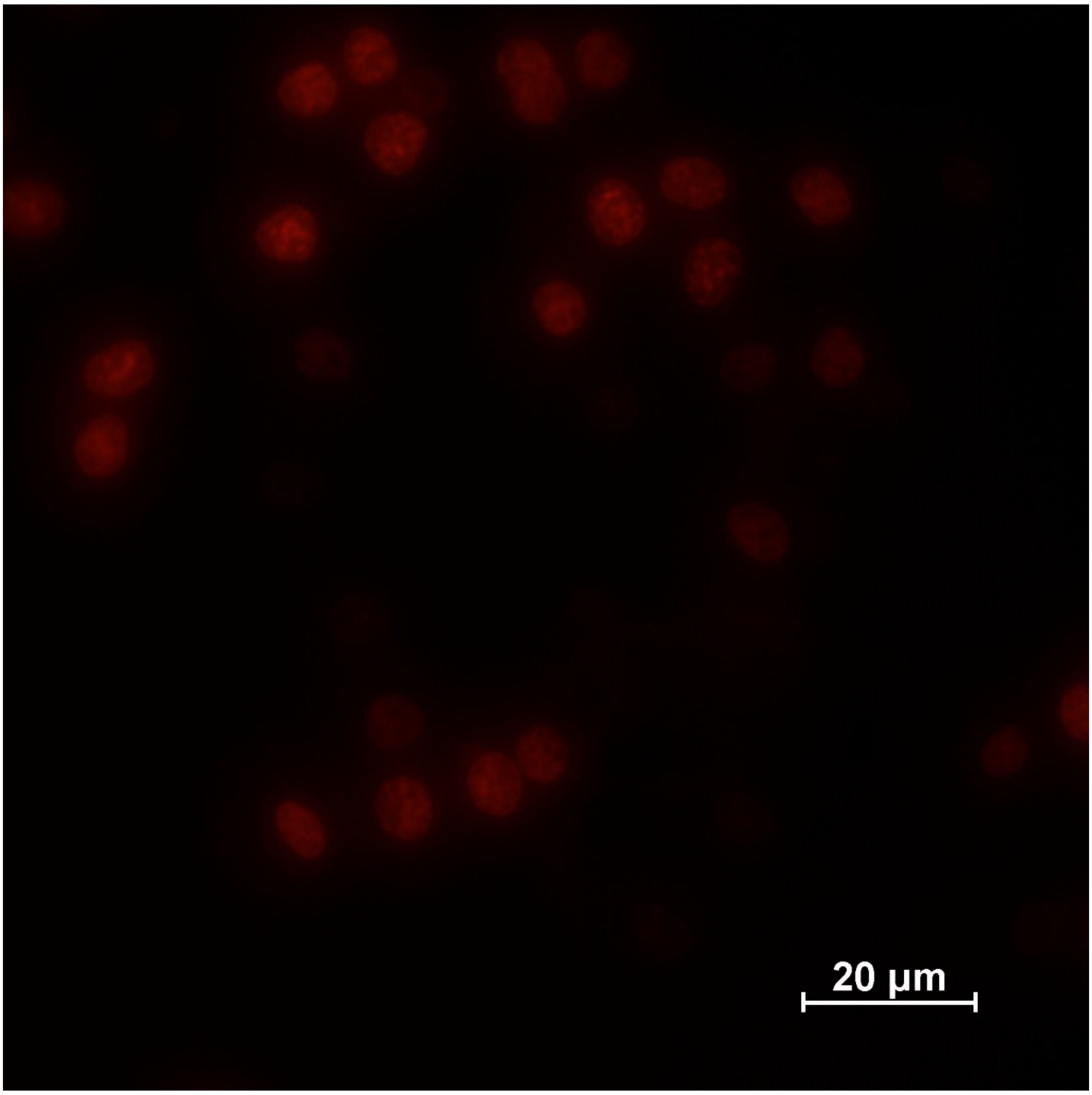

Fig.S4C-Ishikawa-si-E2F3-2-EdU

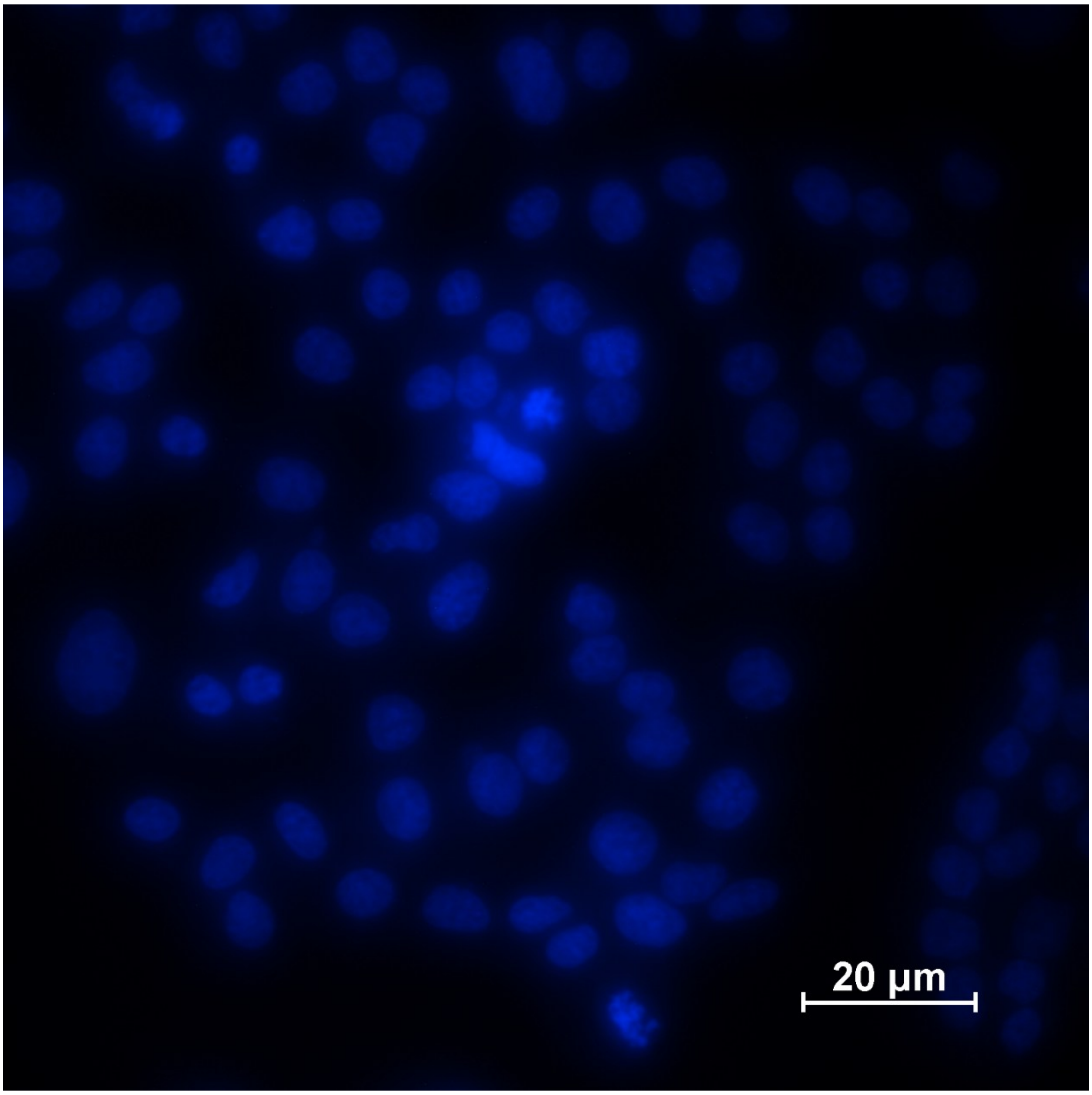

Fig.S4C-Ishikawa-si-E2F3-2-Hoechest

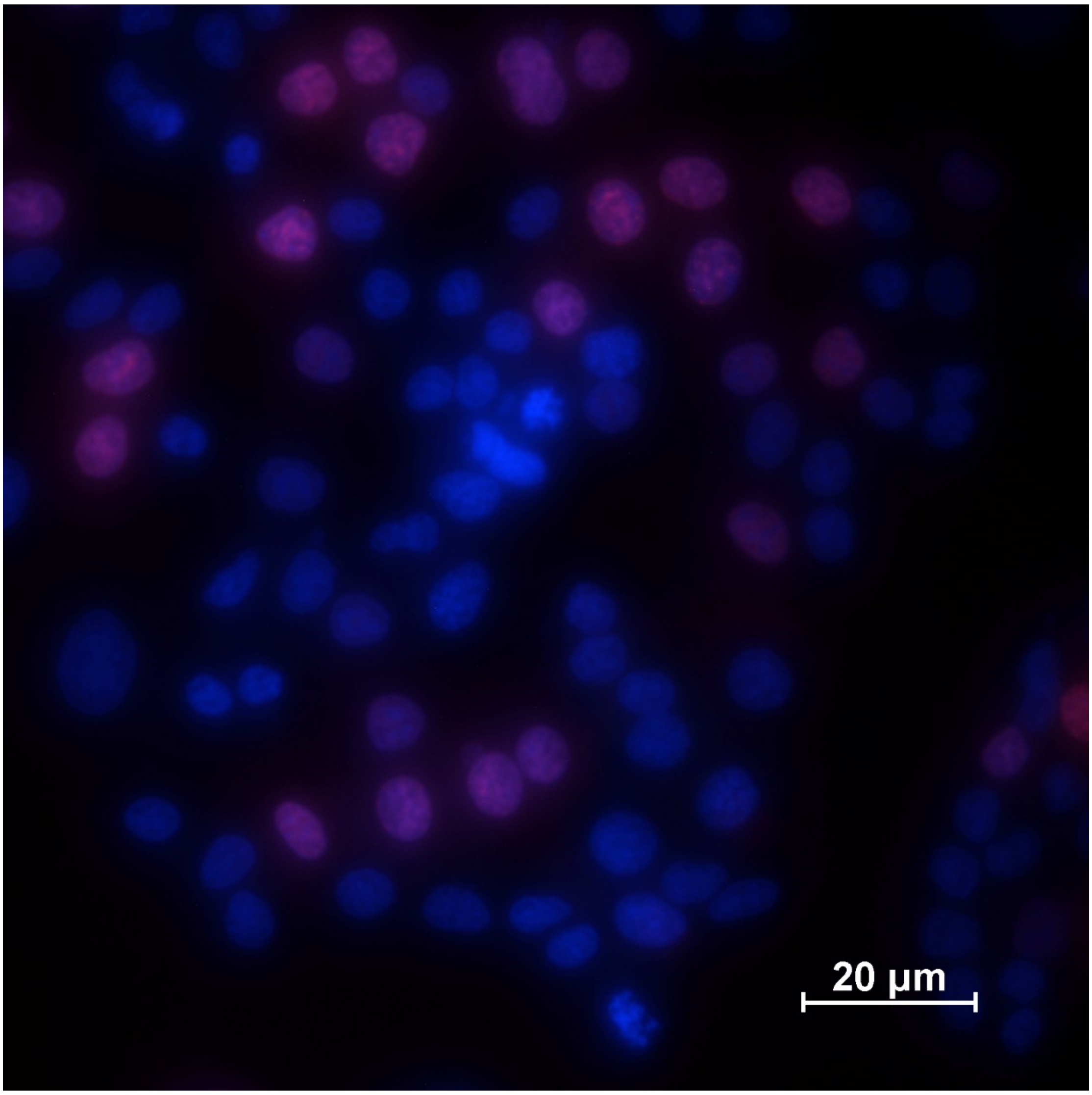

Fig.S4C-Ishikawa-si-E2F3-2-merge

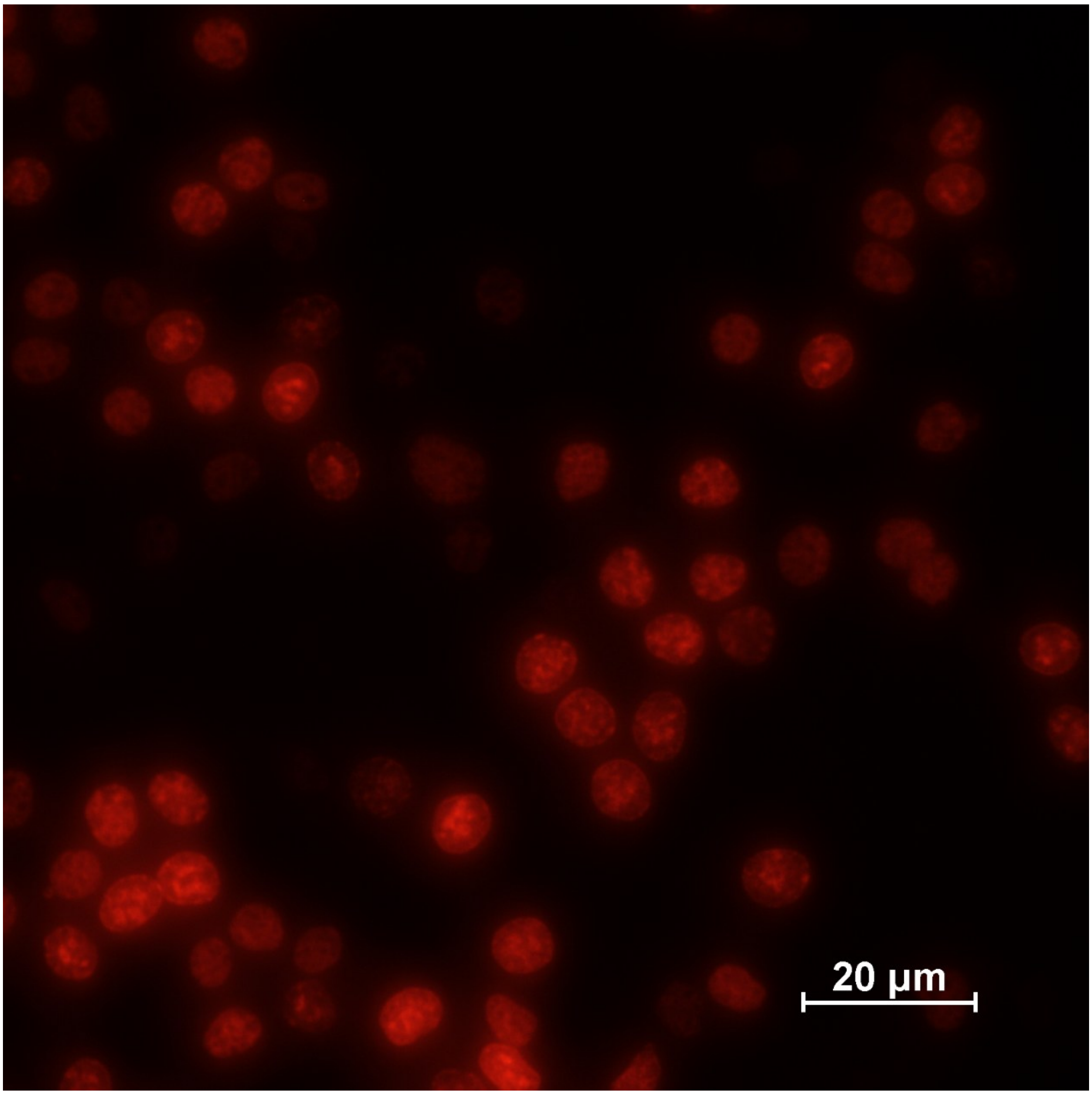

Fig.S4C-Ishikawa-si-NC-EdU

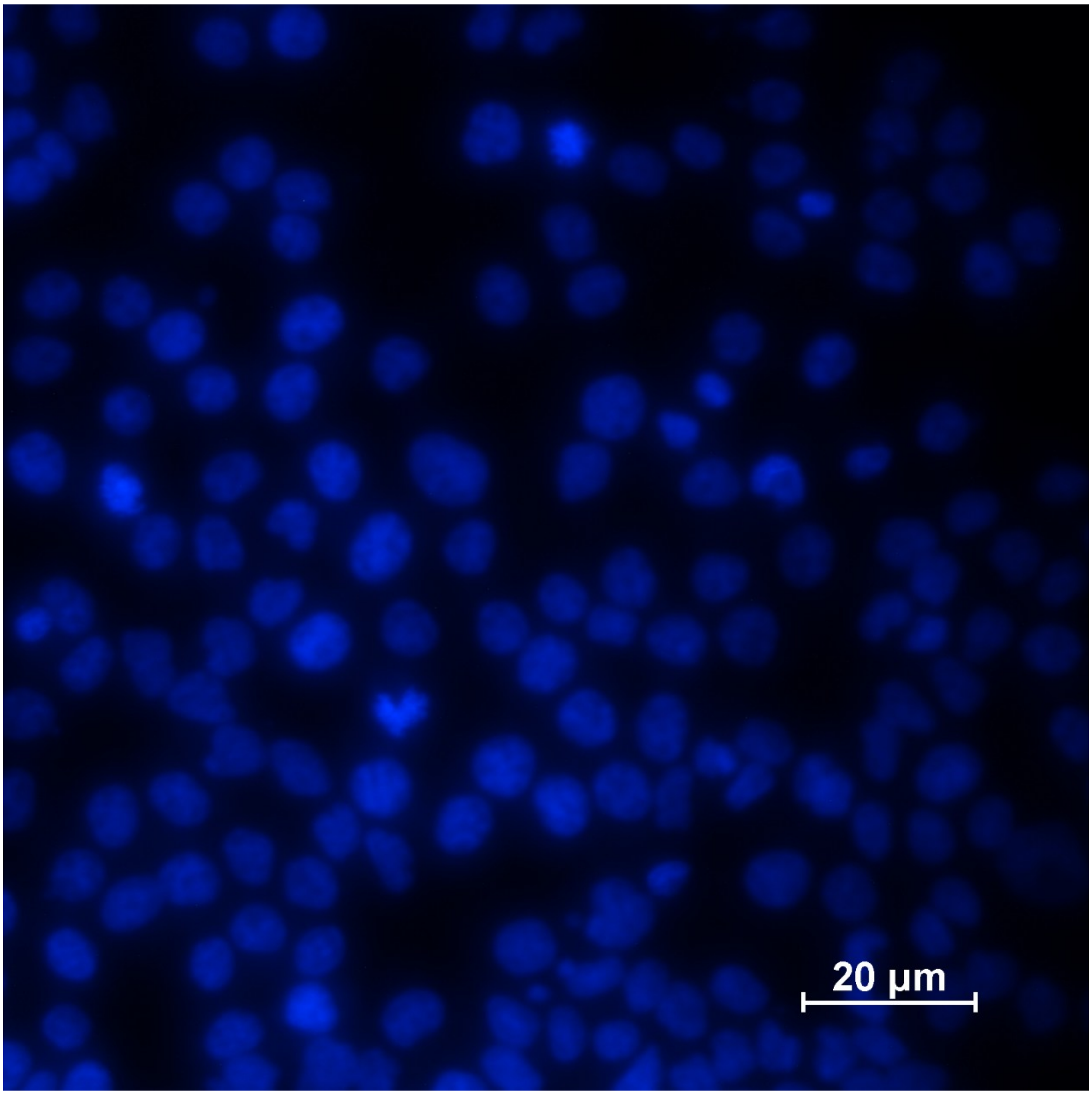

Fig.S4C-Ishikawa-si-NC-Hoechest

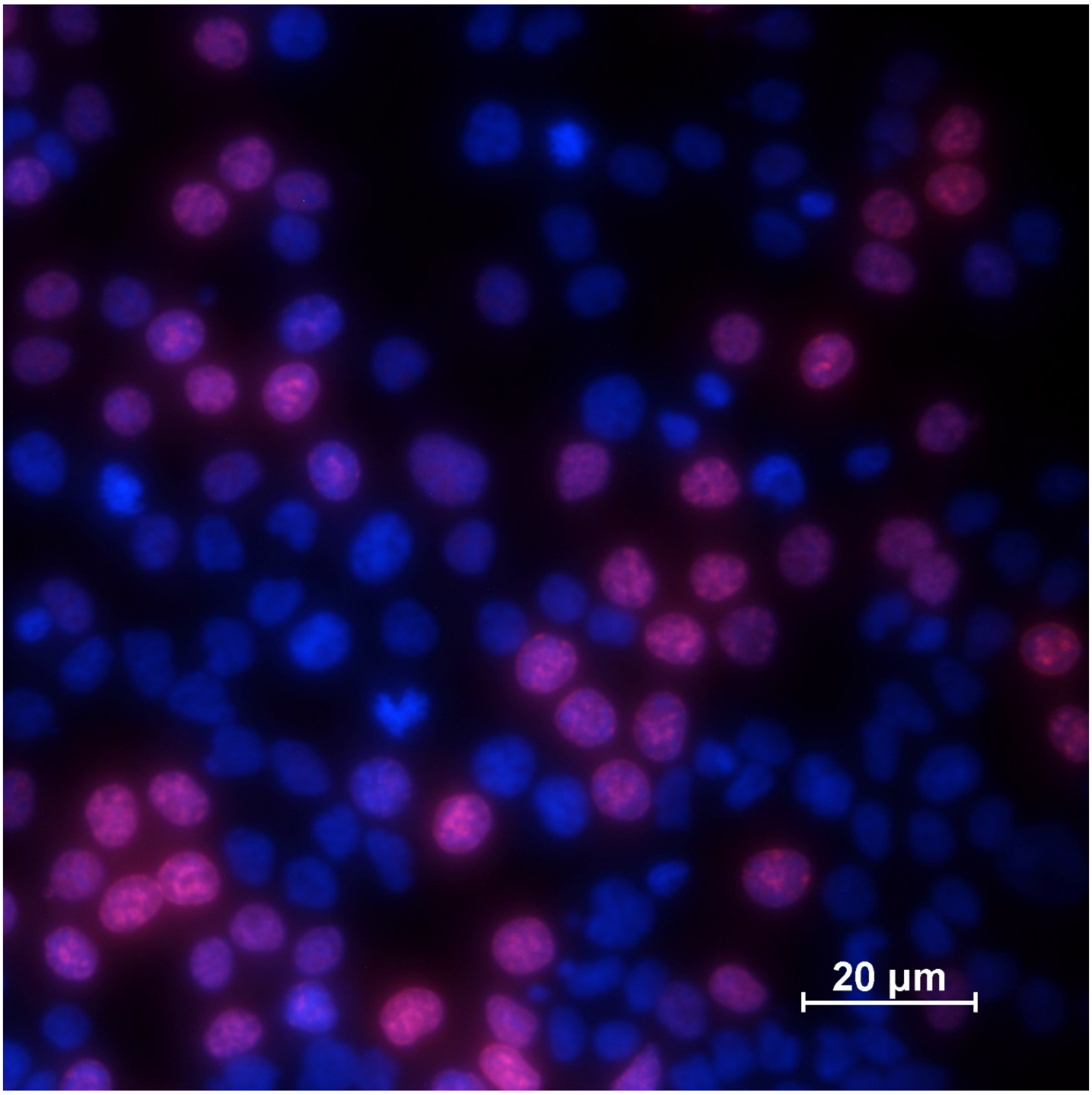

Fig.S4C-Ishikawa-si-NC-merge

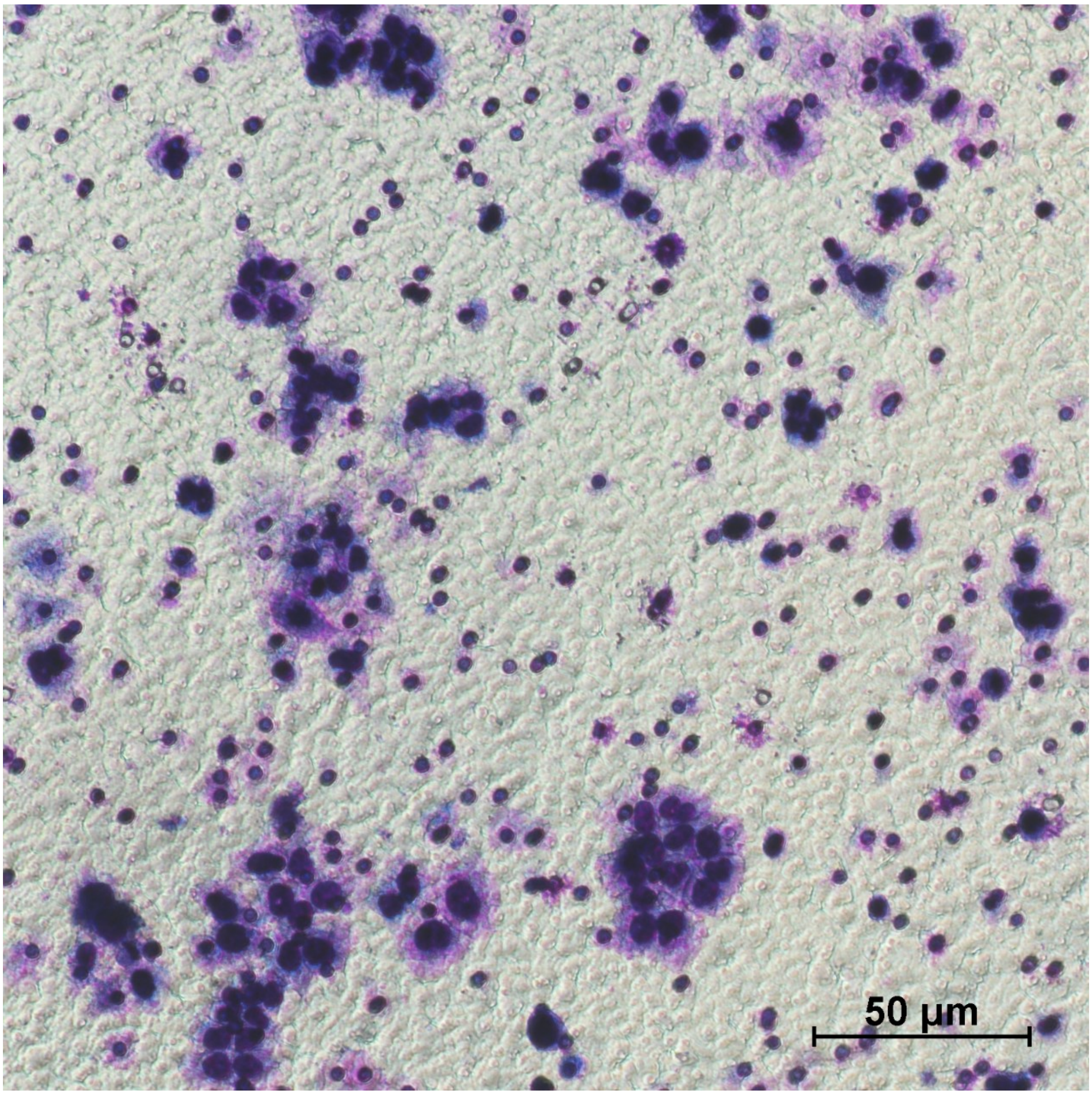

Fig.S4D-HEC-1-A-invasion-si-E2F3-1

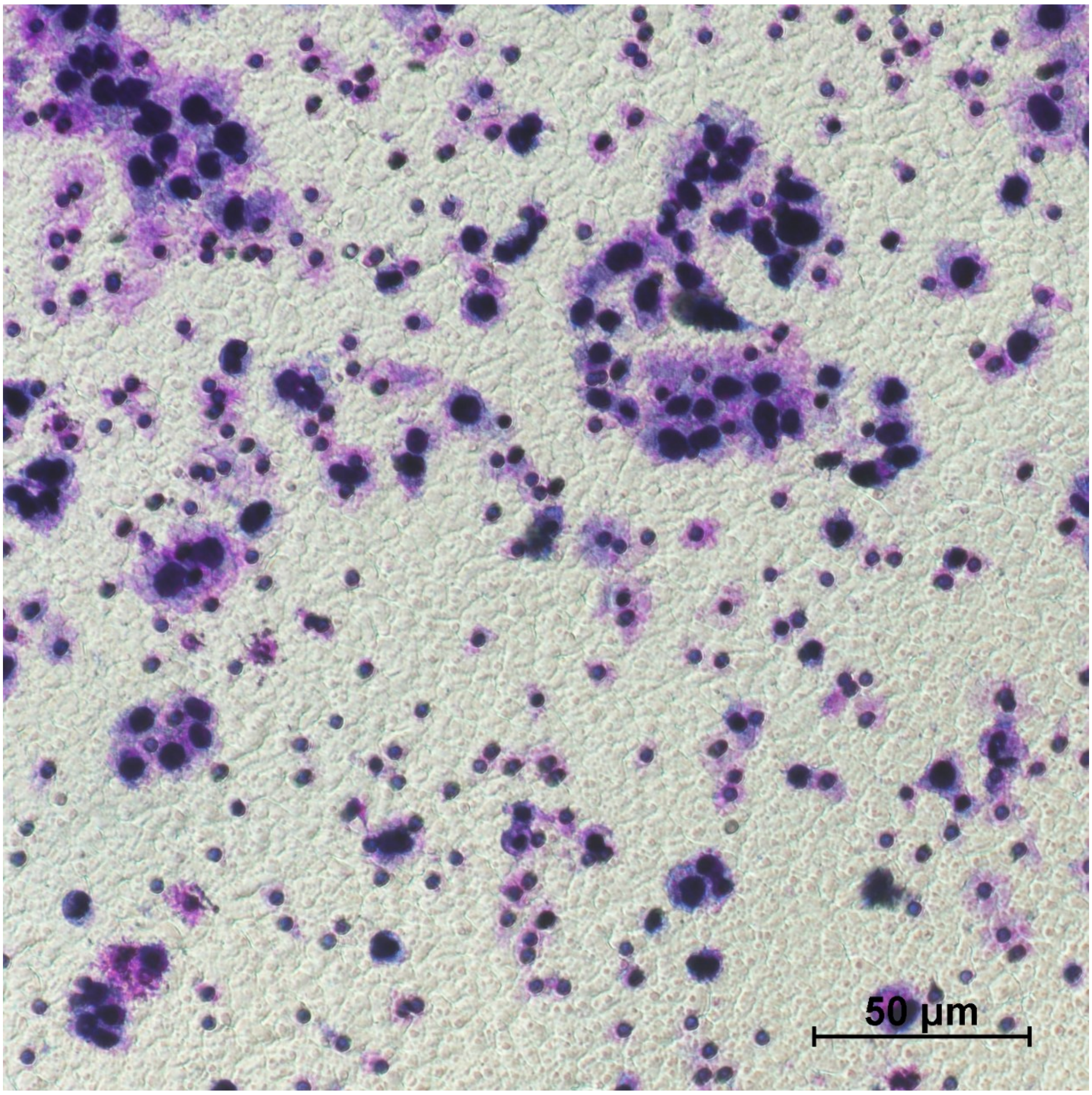

Fig.S4D-HEC-1-A-invasion-si-E2F3-2

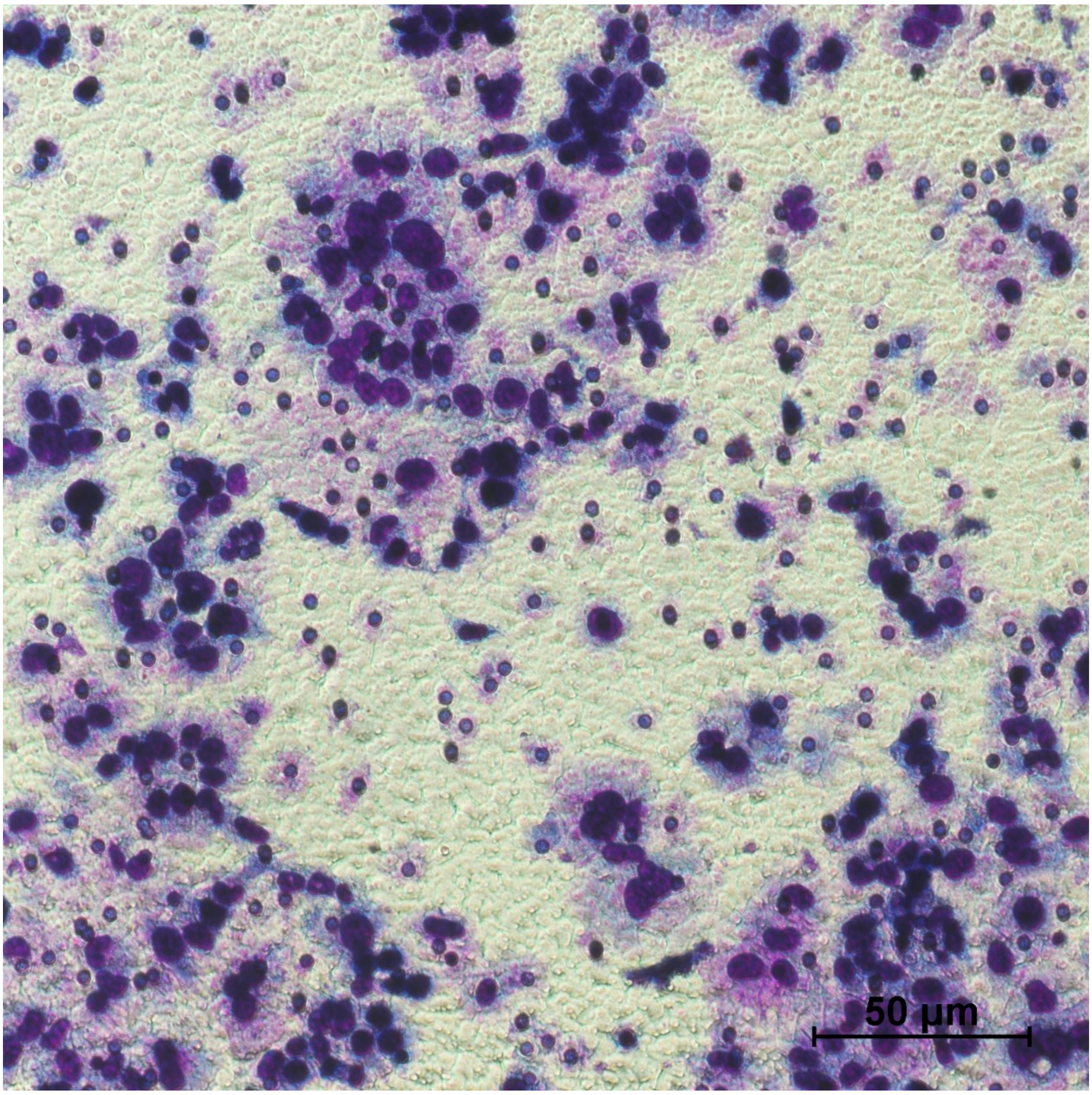

Fig.S4D-HEC-1-A-invasion-si-NC

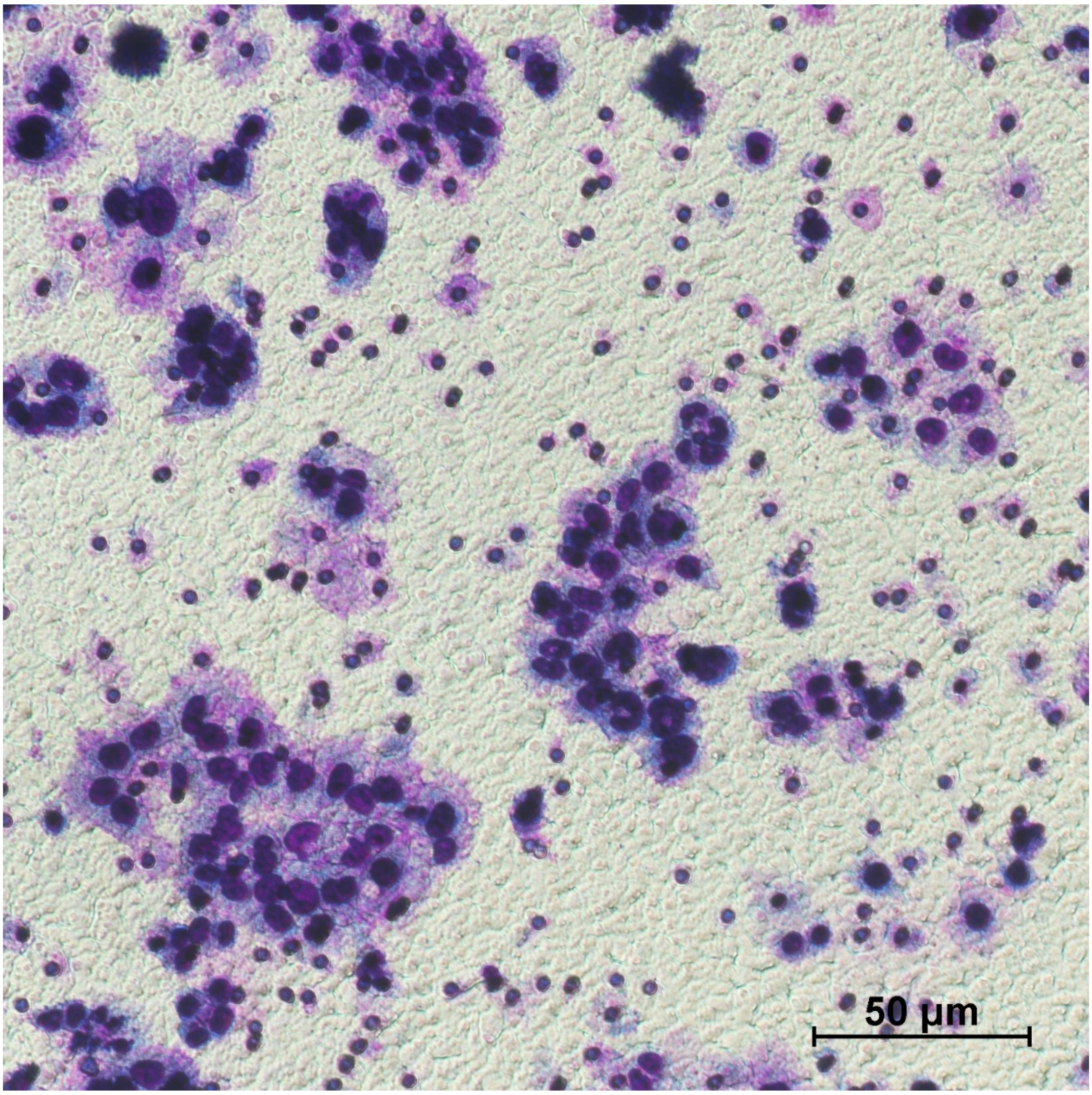

Fig.S4D-HEC-1-A-migration-si-E2F3–1

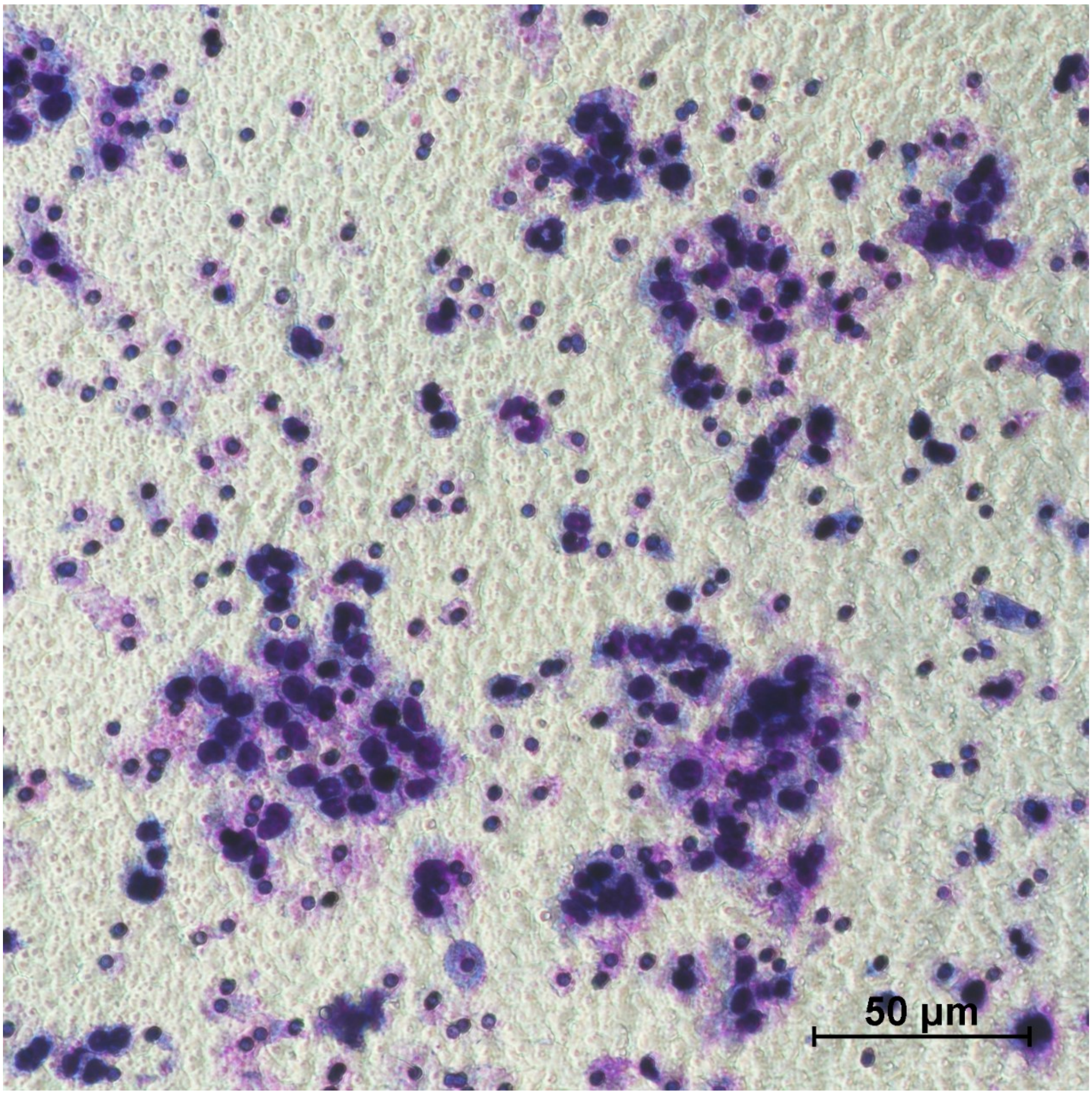

Fig.S4D-HEC-1-A-migration-si-E2F3–2

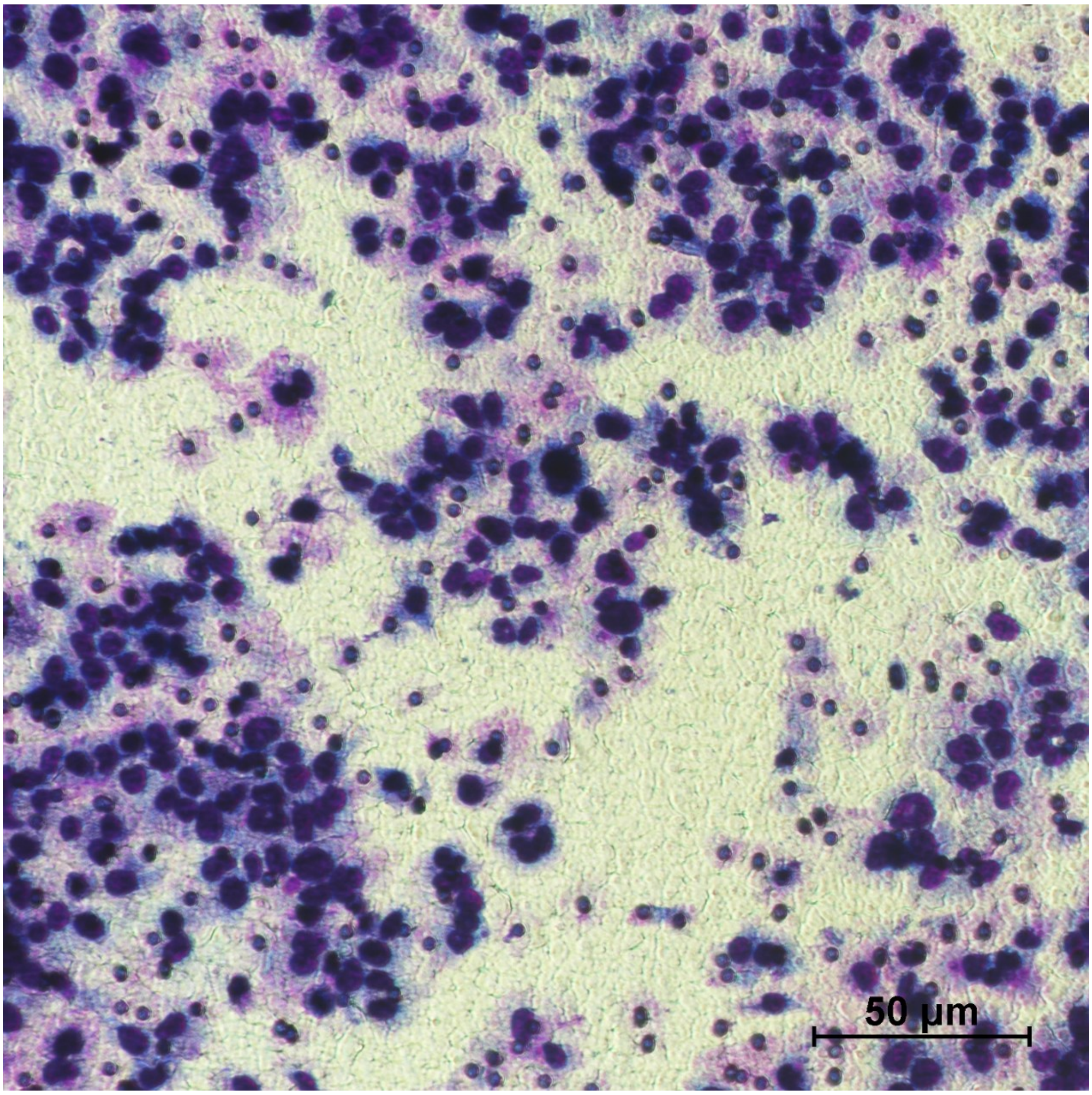

Fig.S4D-HEC-1-A-migration-si-NC

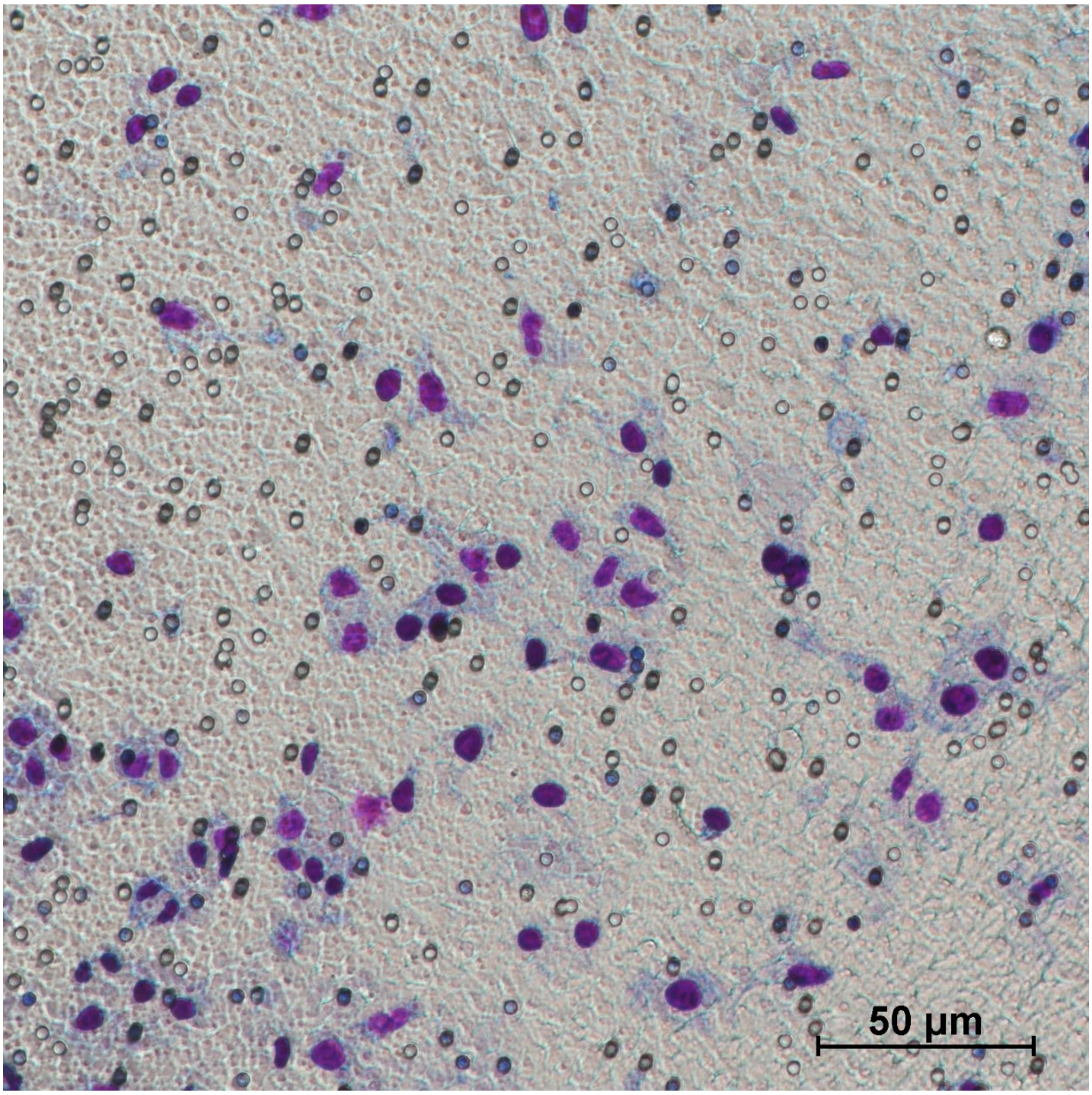

Fig.S4D-Ishikawa-invasion-si-E2F3-1

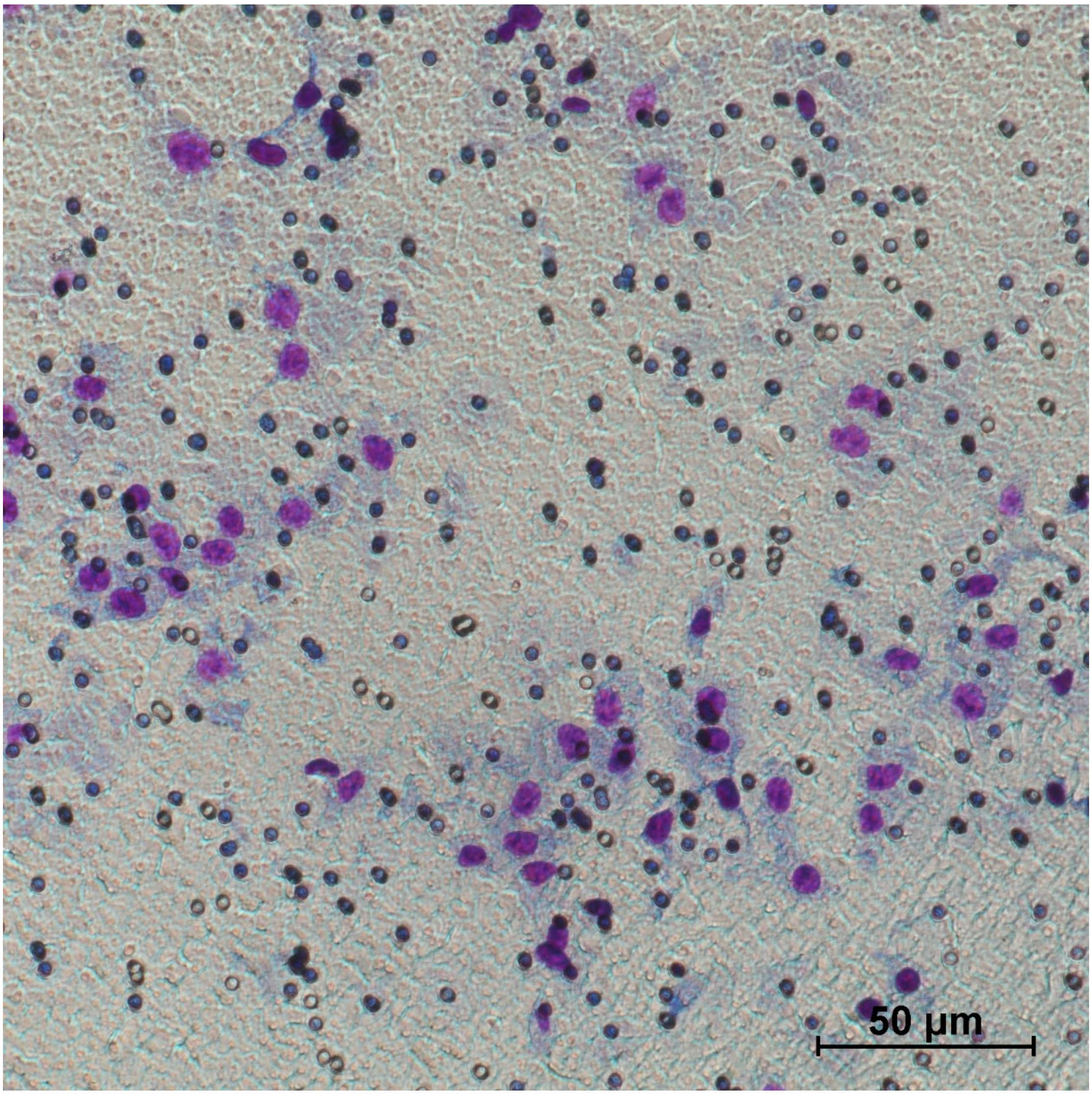

Fig.S4D-Ishikawa-invasion-si-E2F3-2

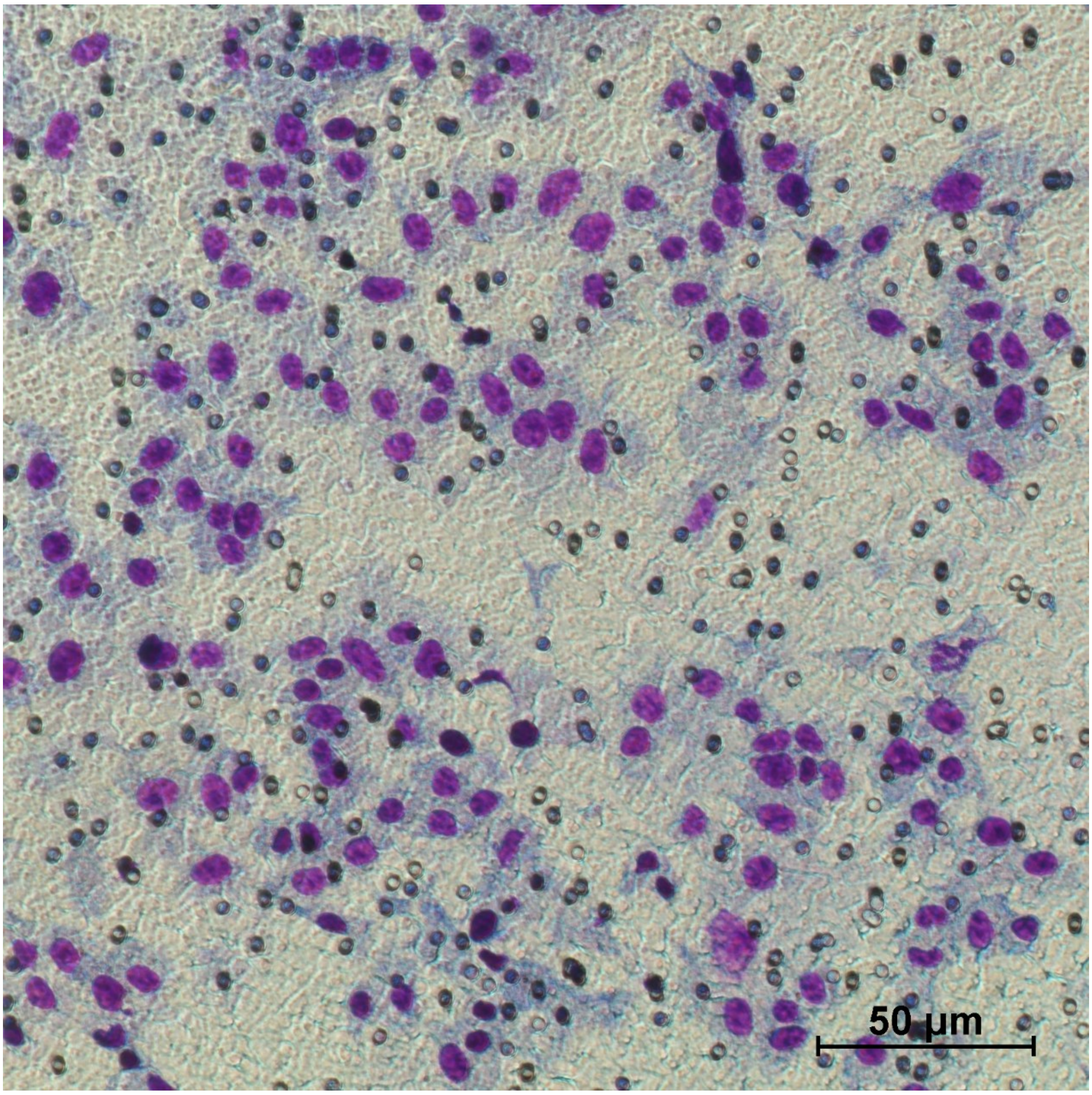

Fig.S4D-Ishikawa-invasion-si-NC

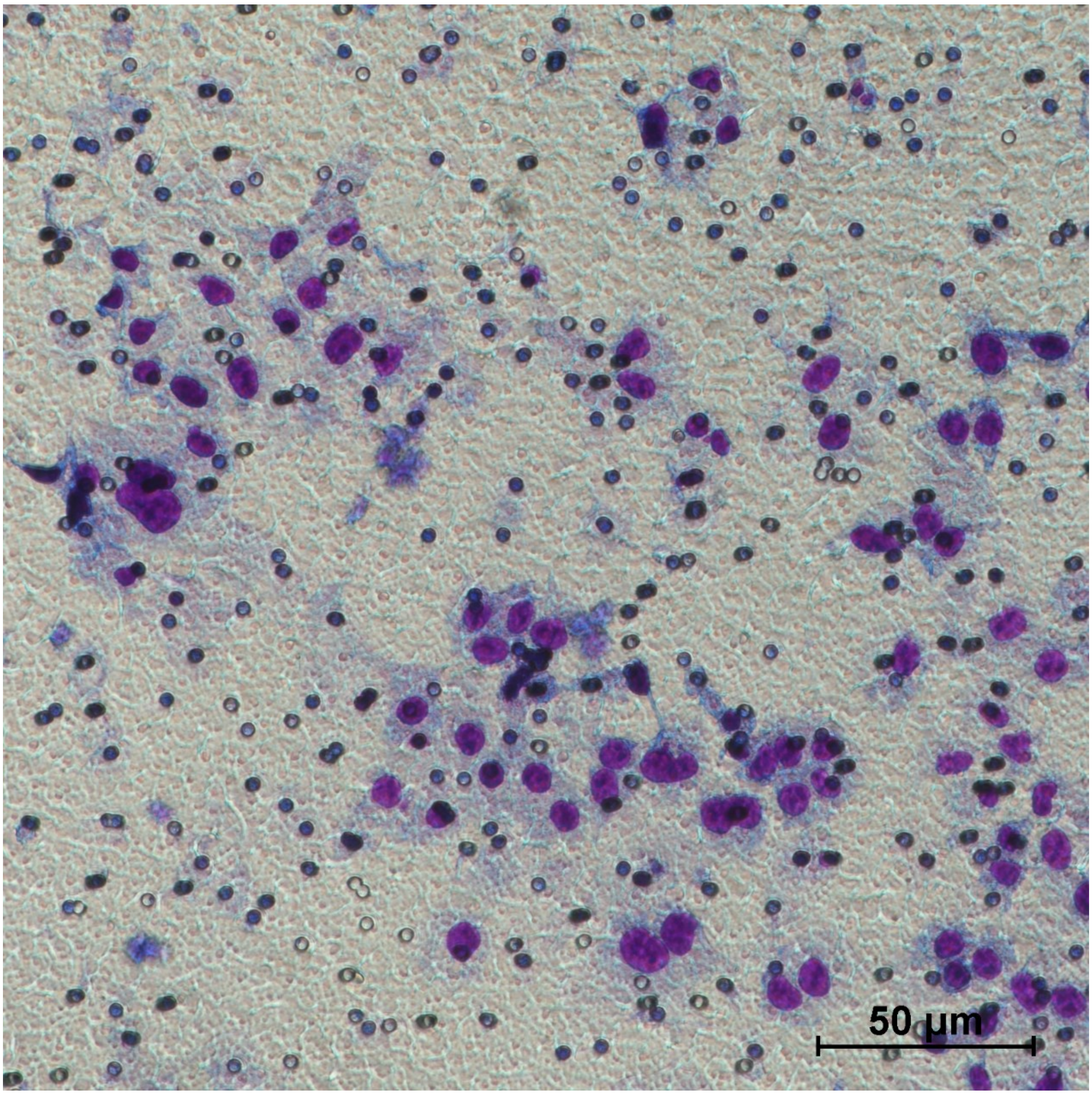

Fig.S4D-Ishikawa-migration-si-E2F3-1

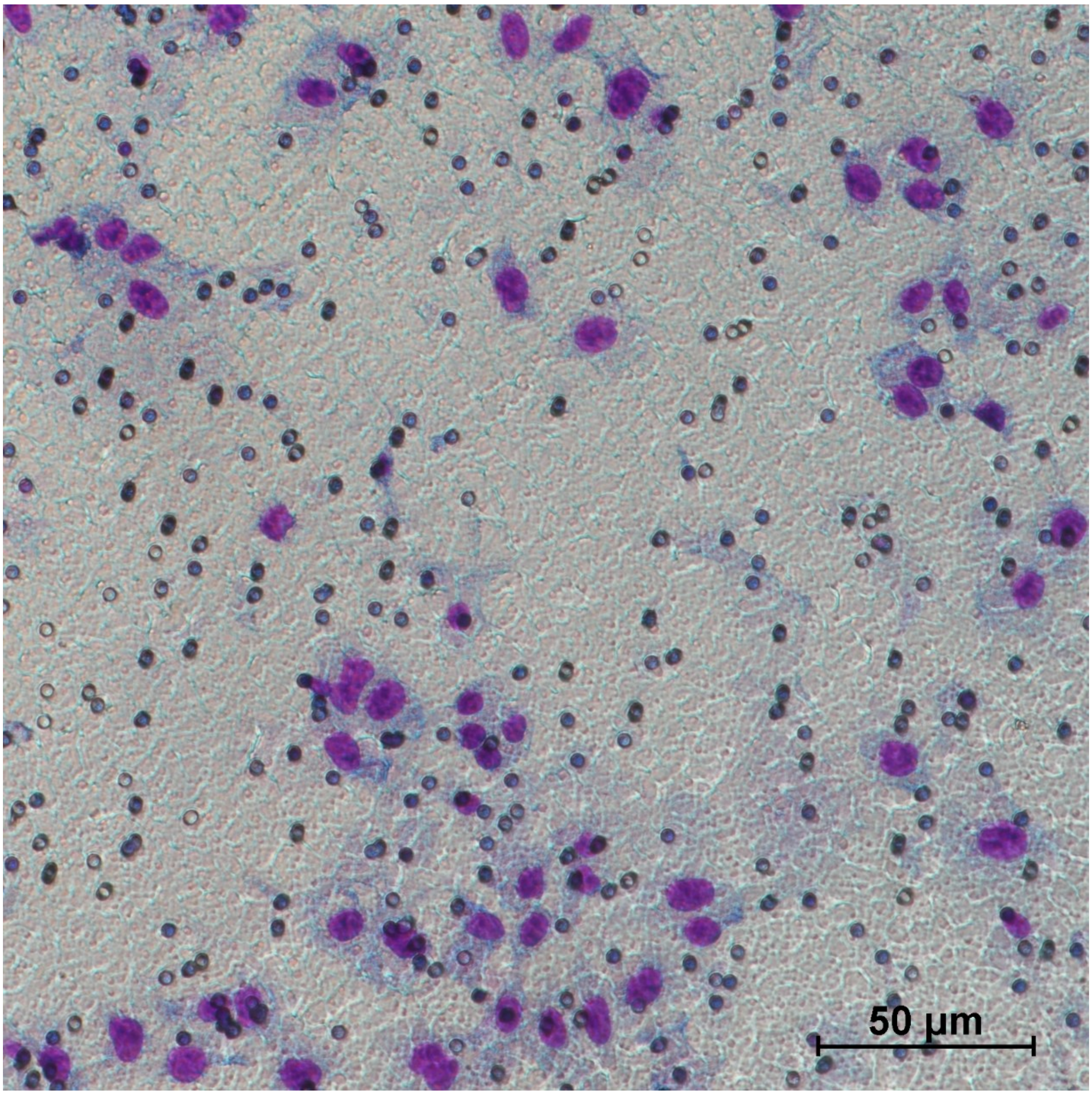

Fig.S4D-Ishikawa-migration-si-E2F3-2

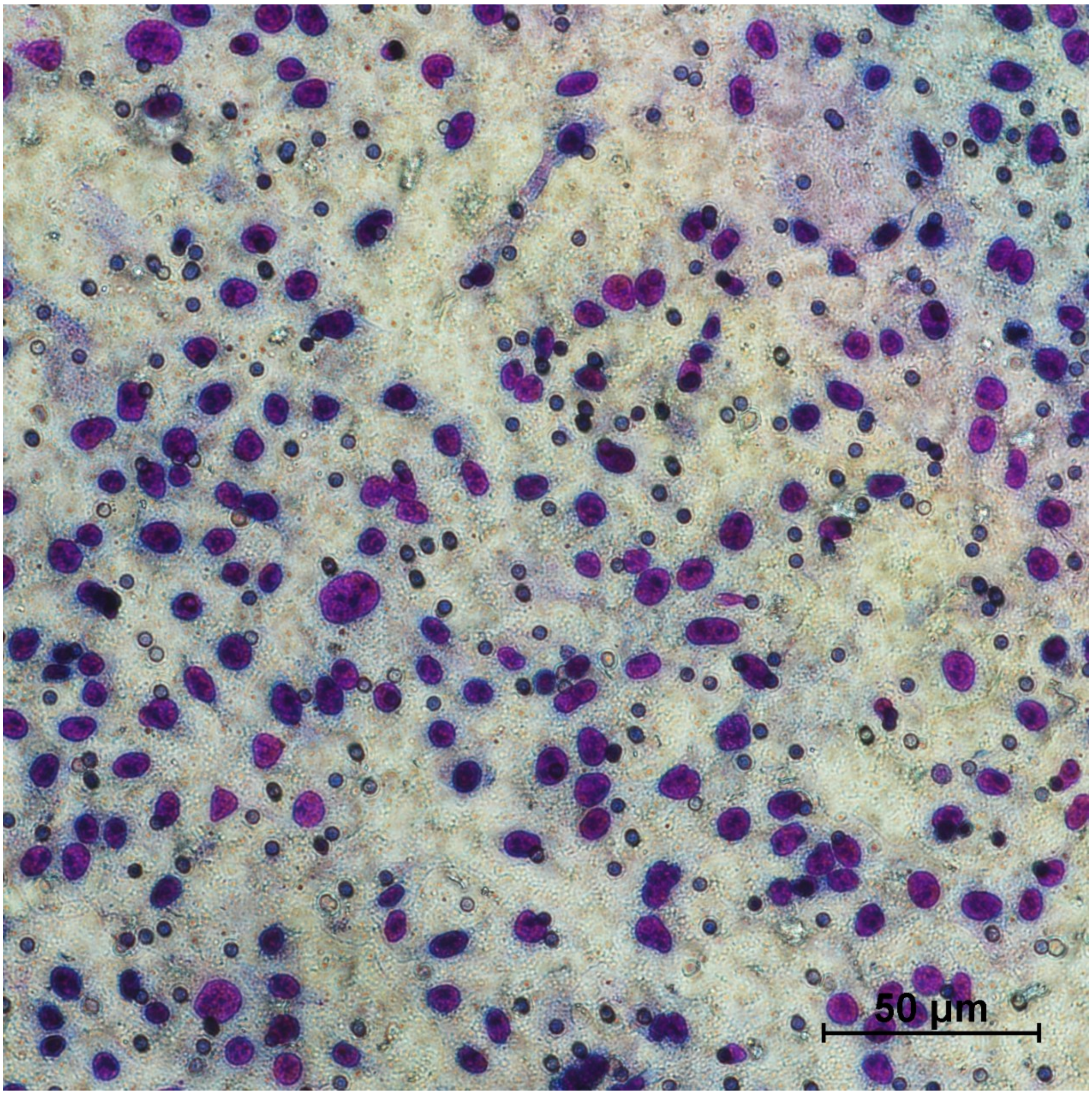

Fig.S4D-Ishikawa-migration-si-NC

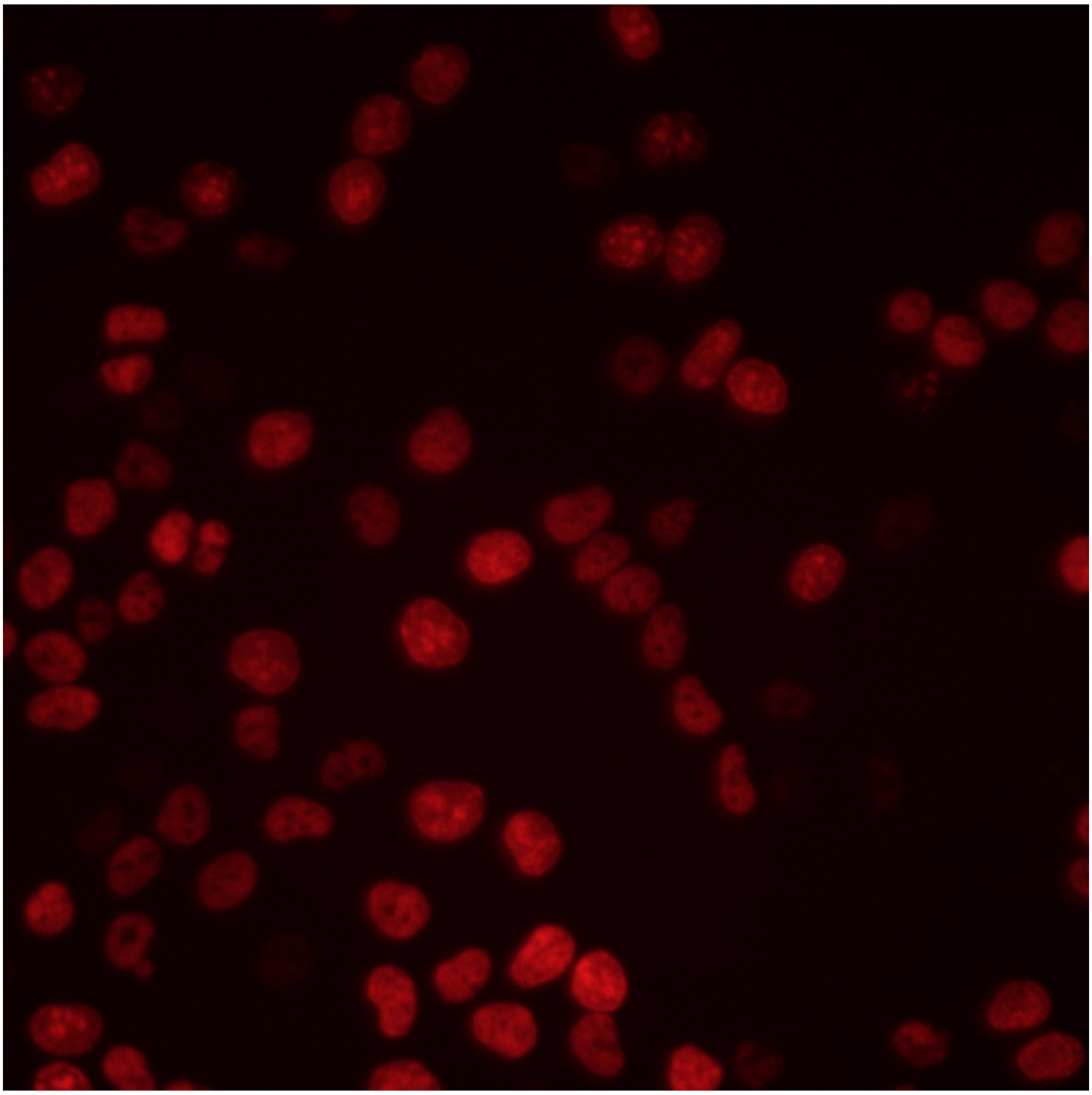

Fig.S5A-HEC-1-A-IGF2BP3-OE+si-E2F3-EdU

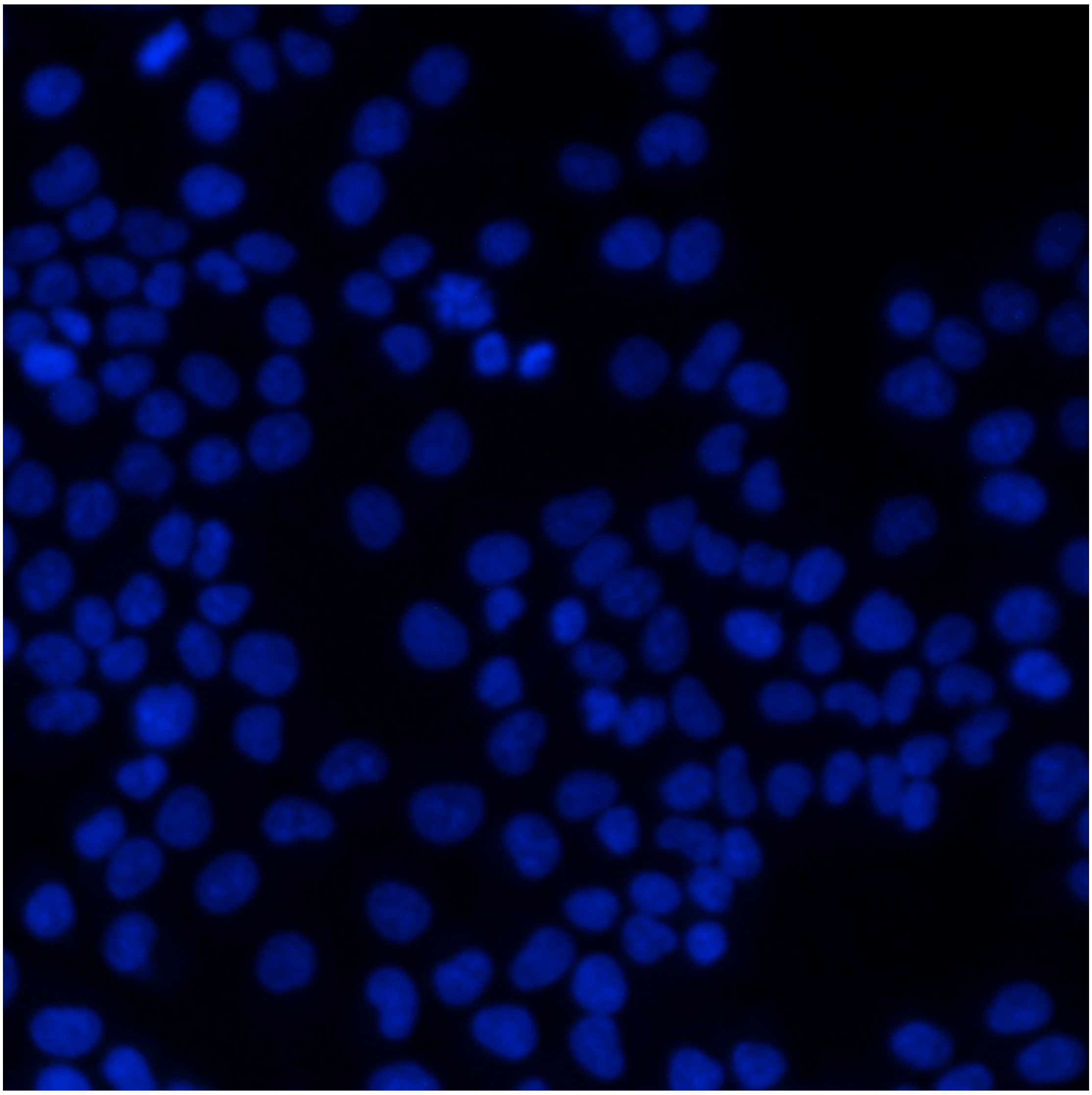

Fig.S5A-HEC-1-A-IGF2BP3-OE+si-E2F3-Hoechst

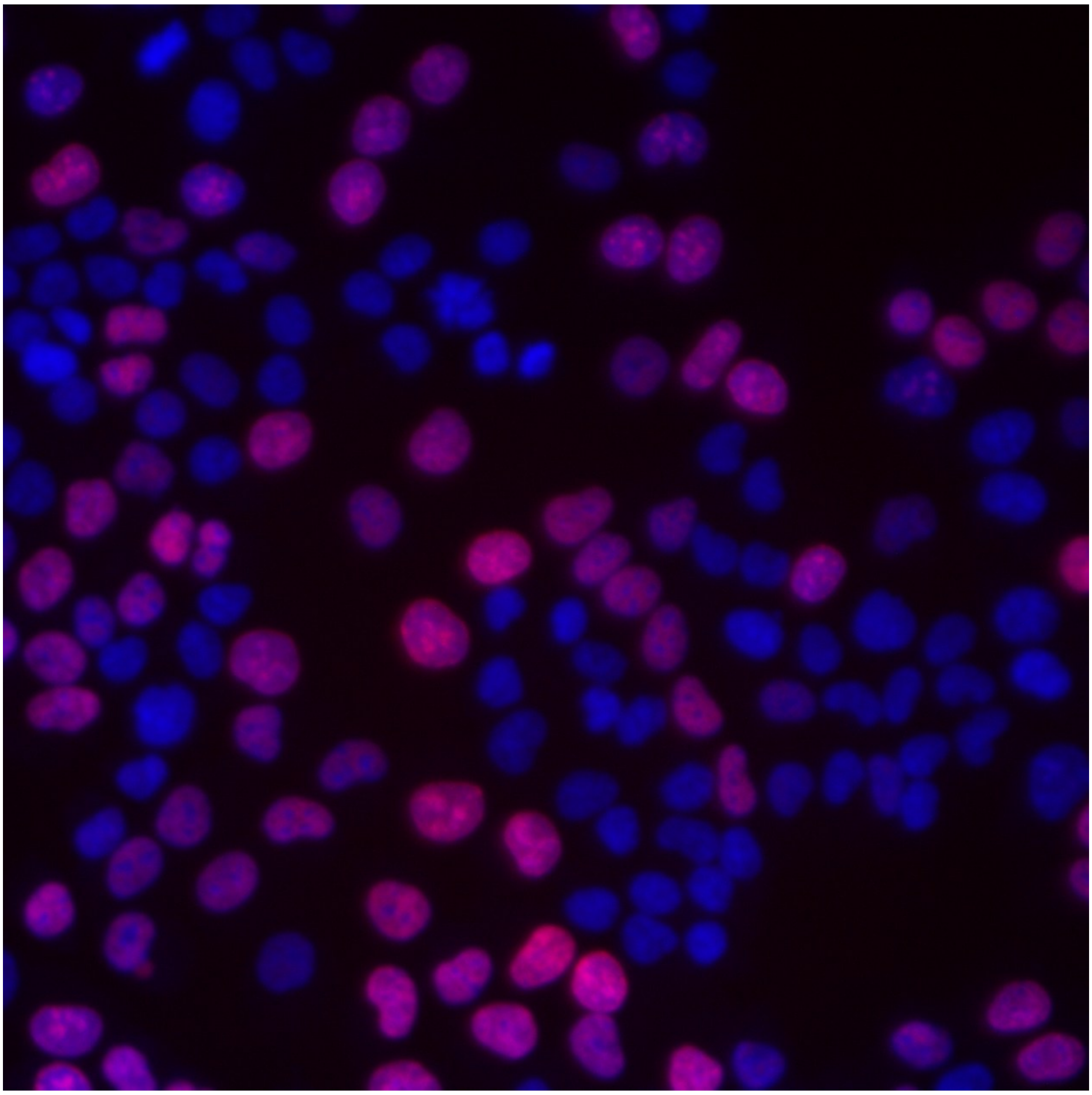

Fig.S5A-HEC-1-A-IGF2BP3-OE+si-E2F3-Merge

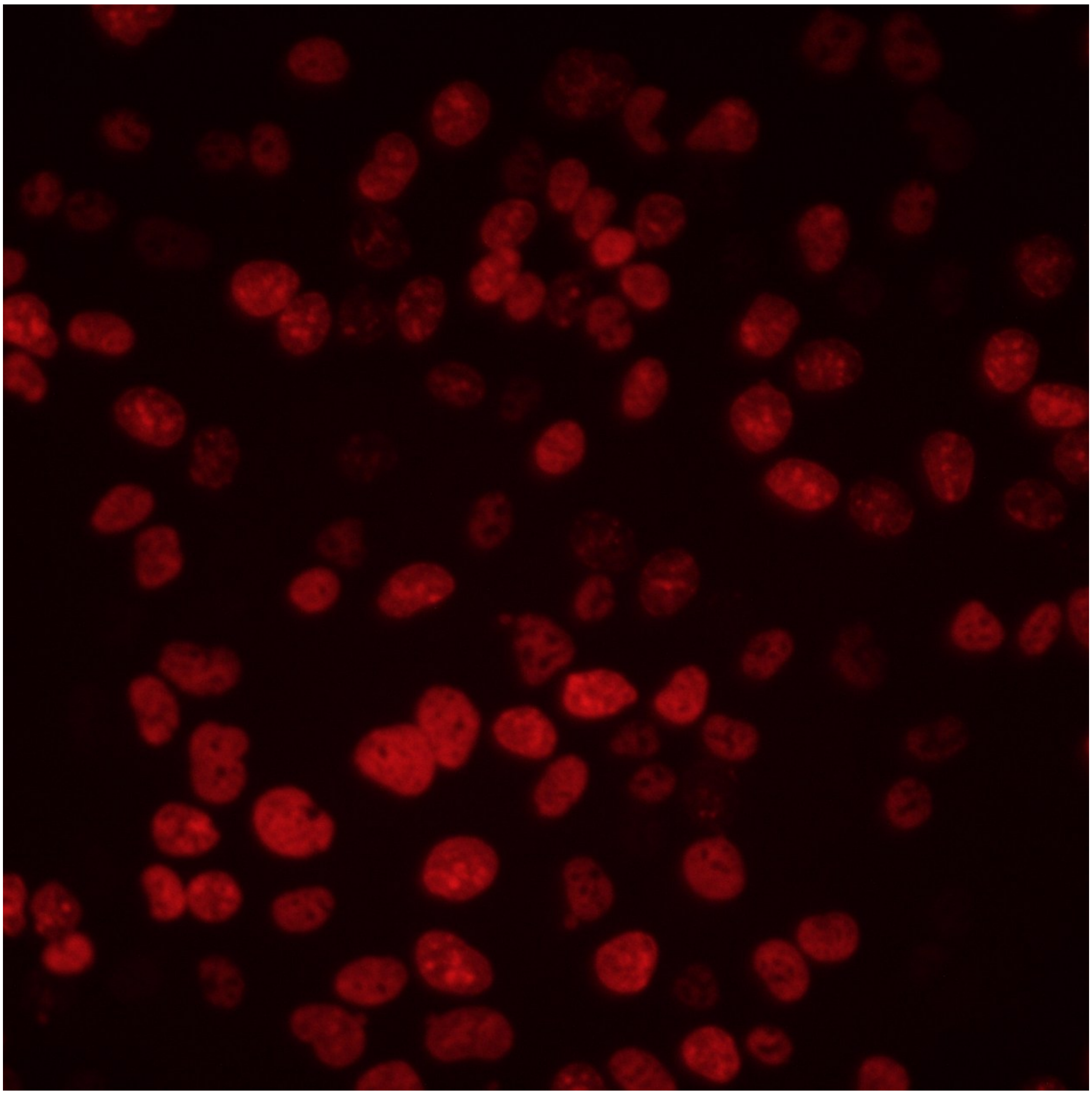

Fig.S5A-HEC-1-A-IGF2BP3-OE+si-NC-EdU

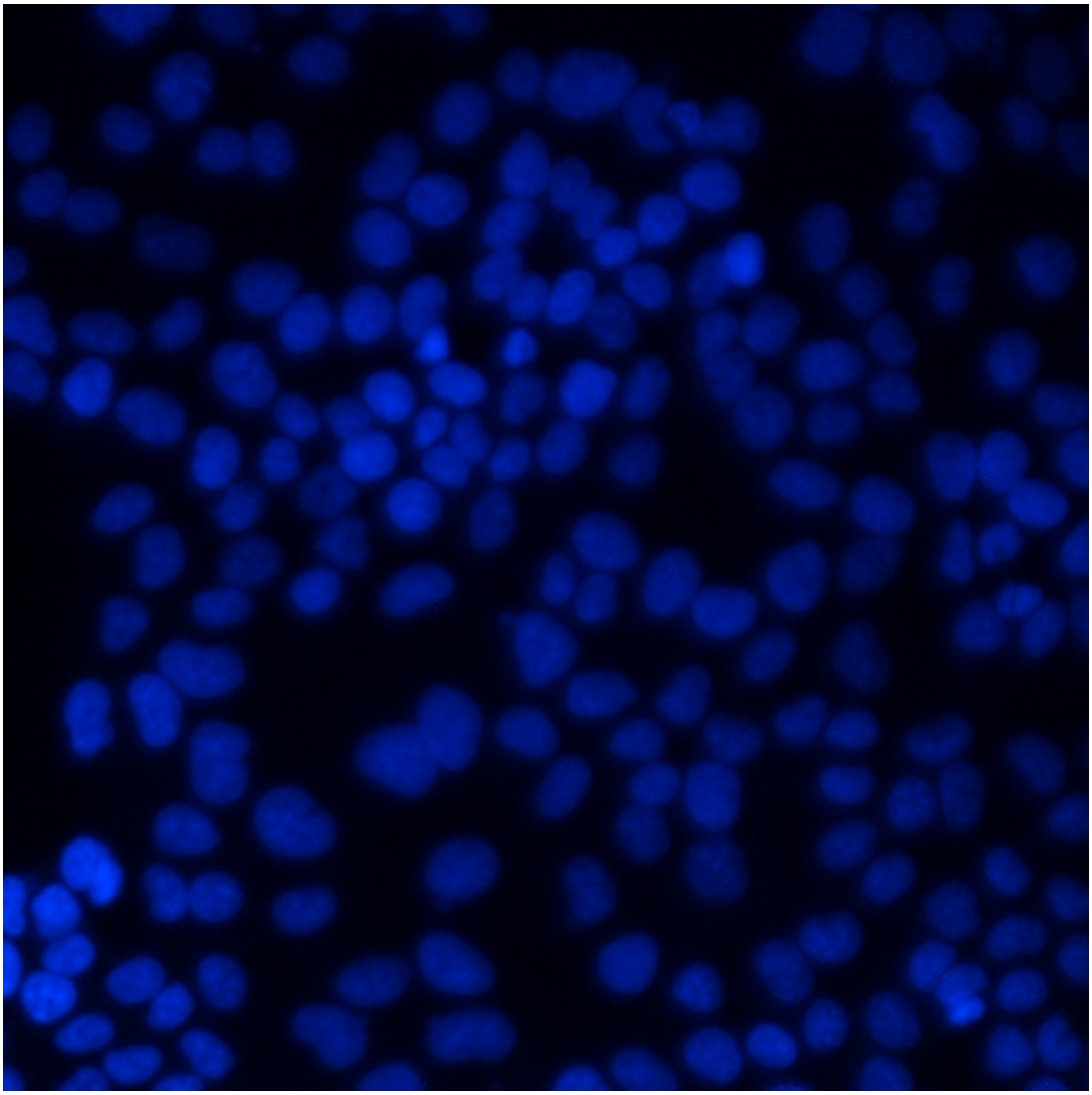
Fig.S5A-HEC-1-A-IGF2BP3-OE+si-NC-Hoechst

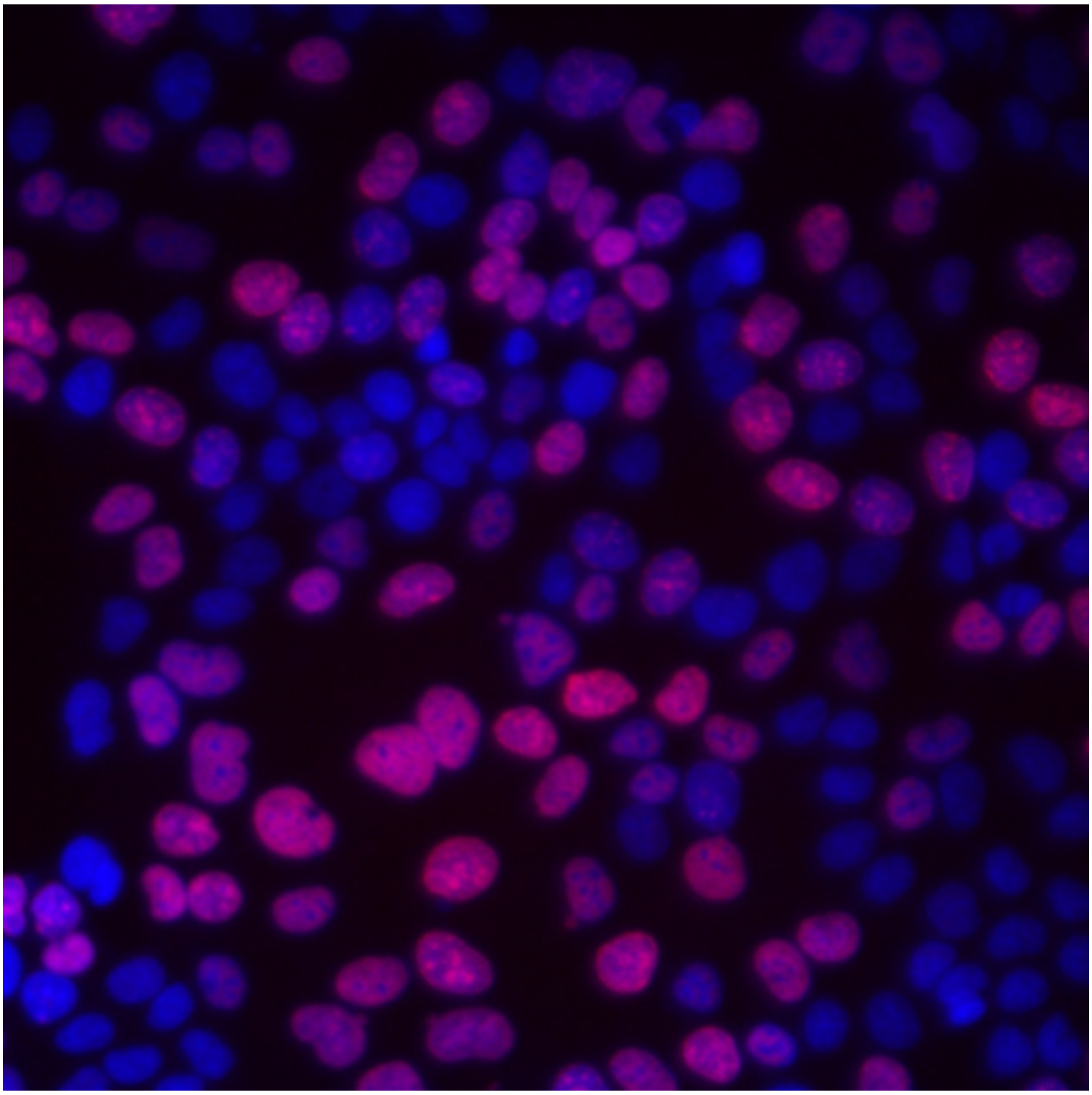

Fig.S5A-HEC-1-A-IGF2BP3-OE+si-NC-Merge

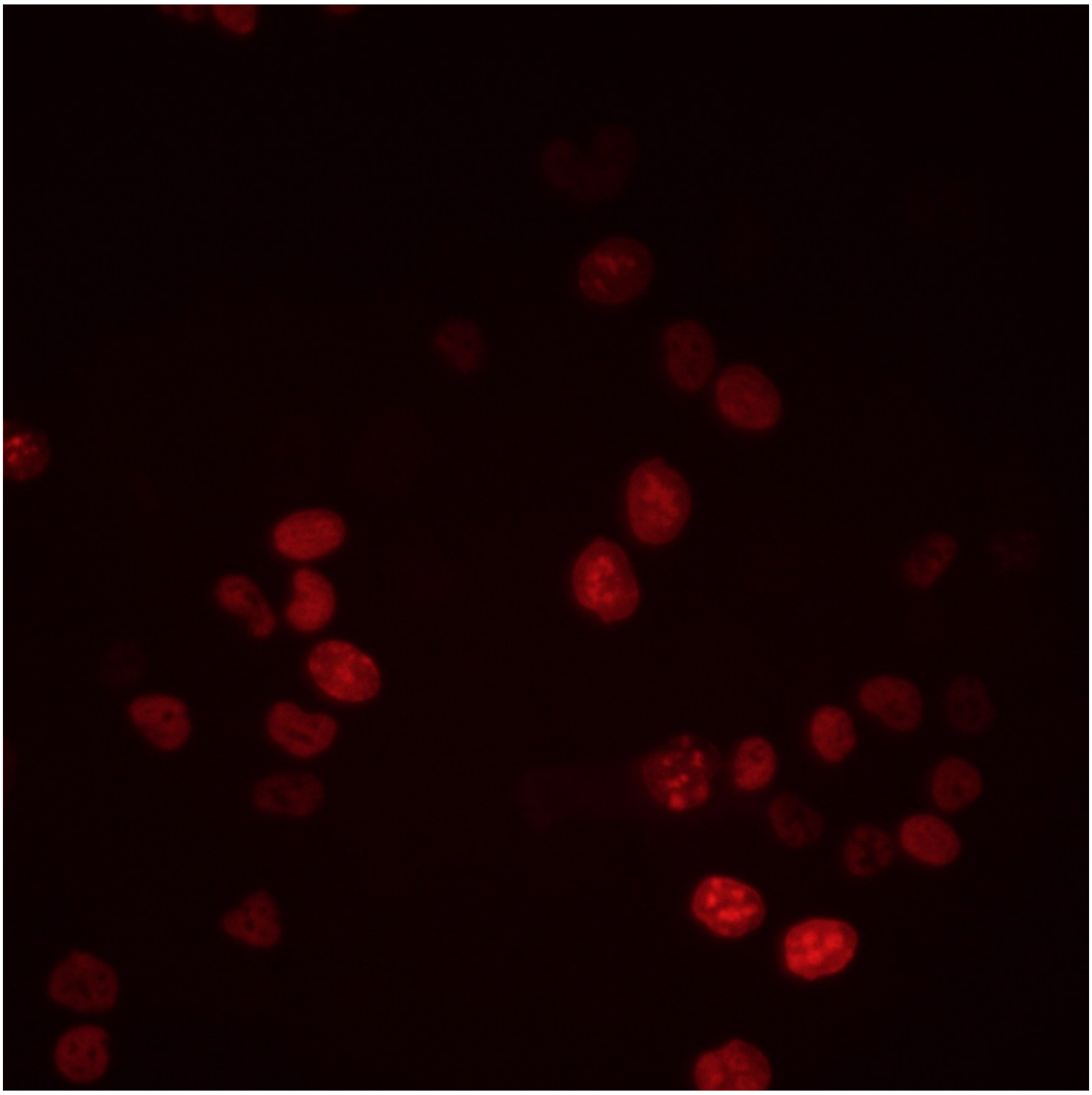

Fig.S5A-HEC-1-A-LV-NC+si-E2F3-EdU

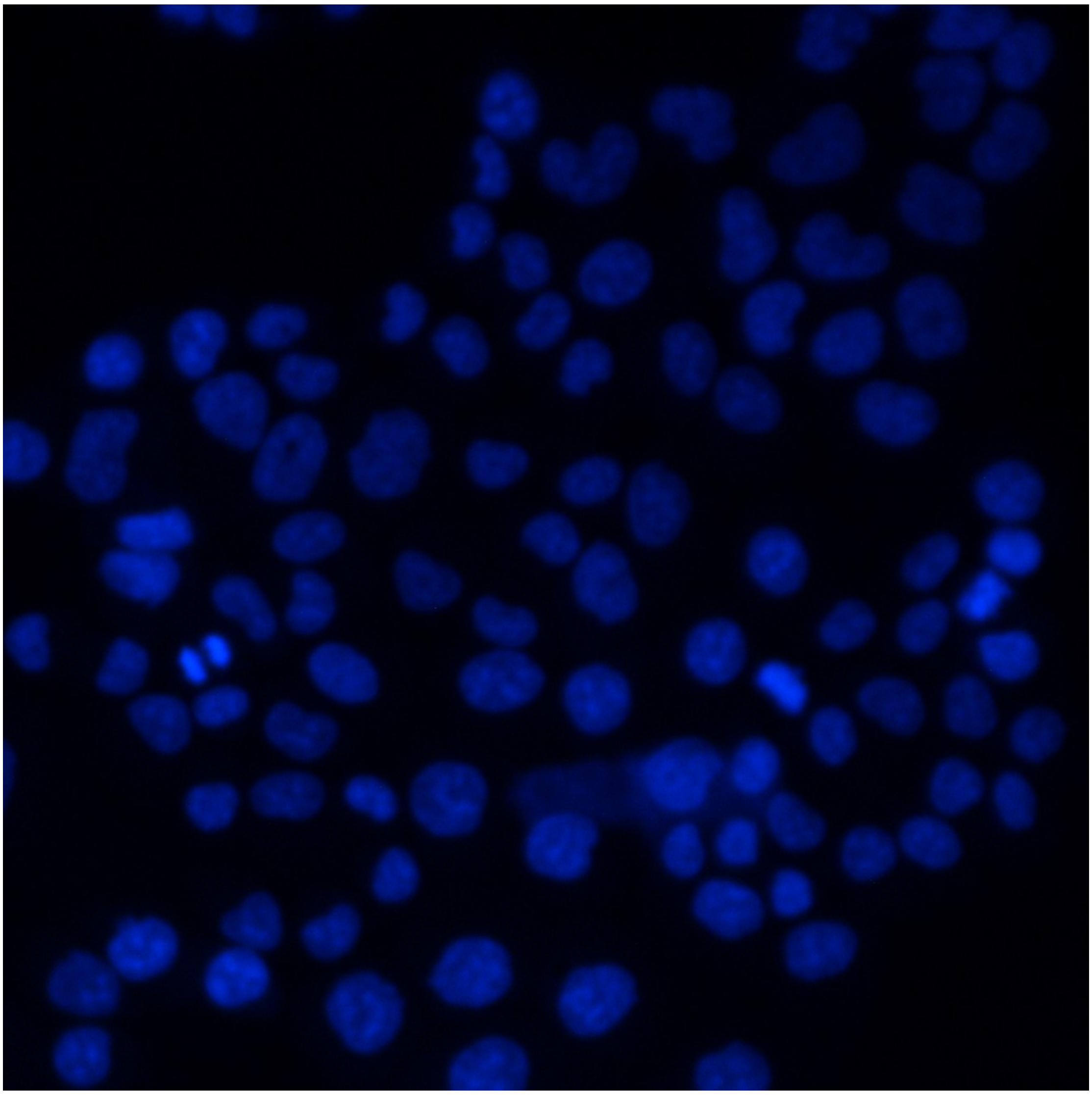

Fig.S5A-HEC-1-A-LV-NC+si-E2F3-Hoechst

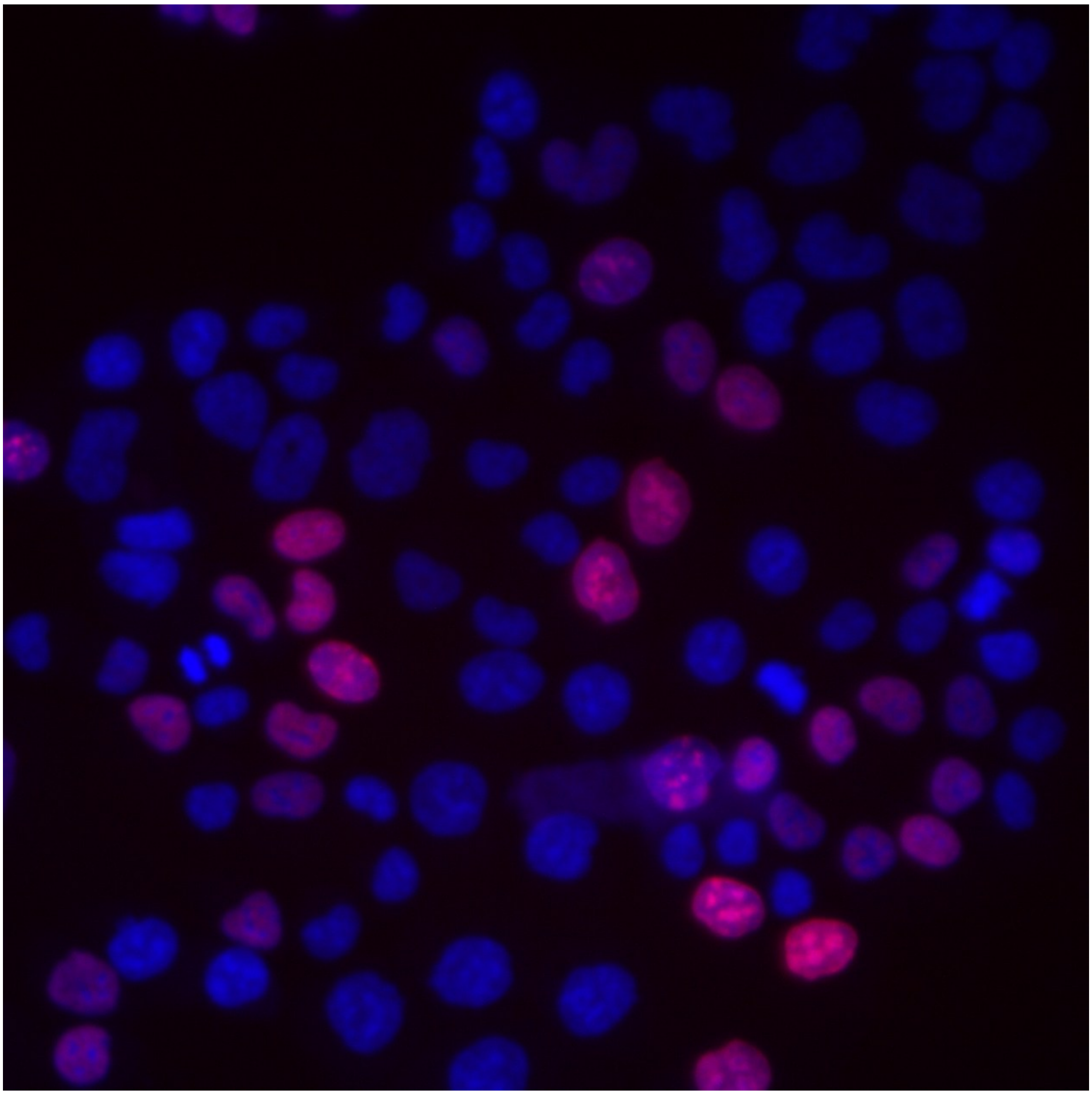

Fig.S5A-HEC-1-A-LV-NC+si-E2F3-Merge

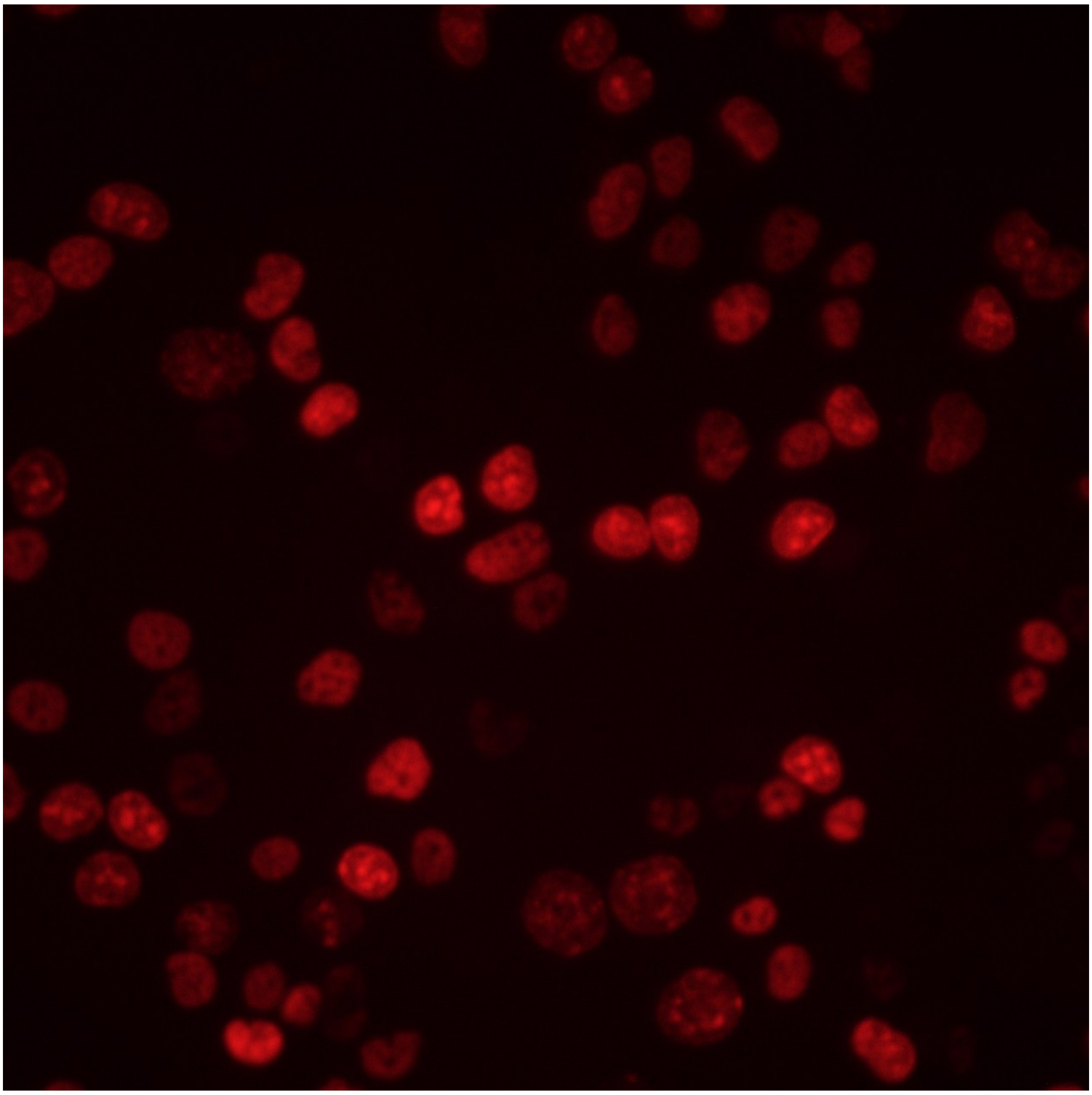

Fig.S5A-HEC-1-A-LV-NC+si-NC-EdU

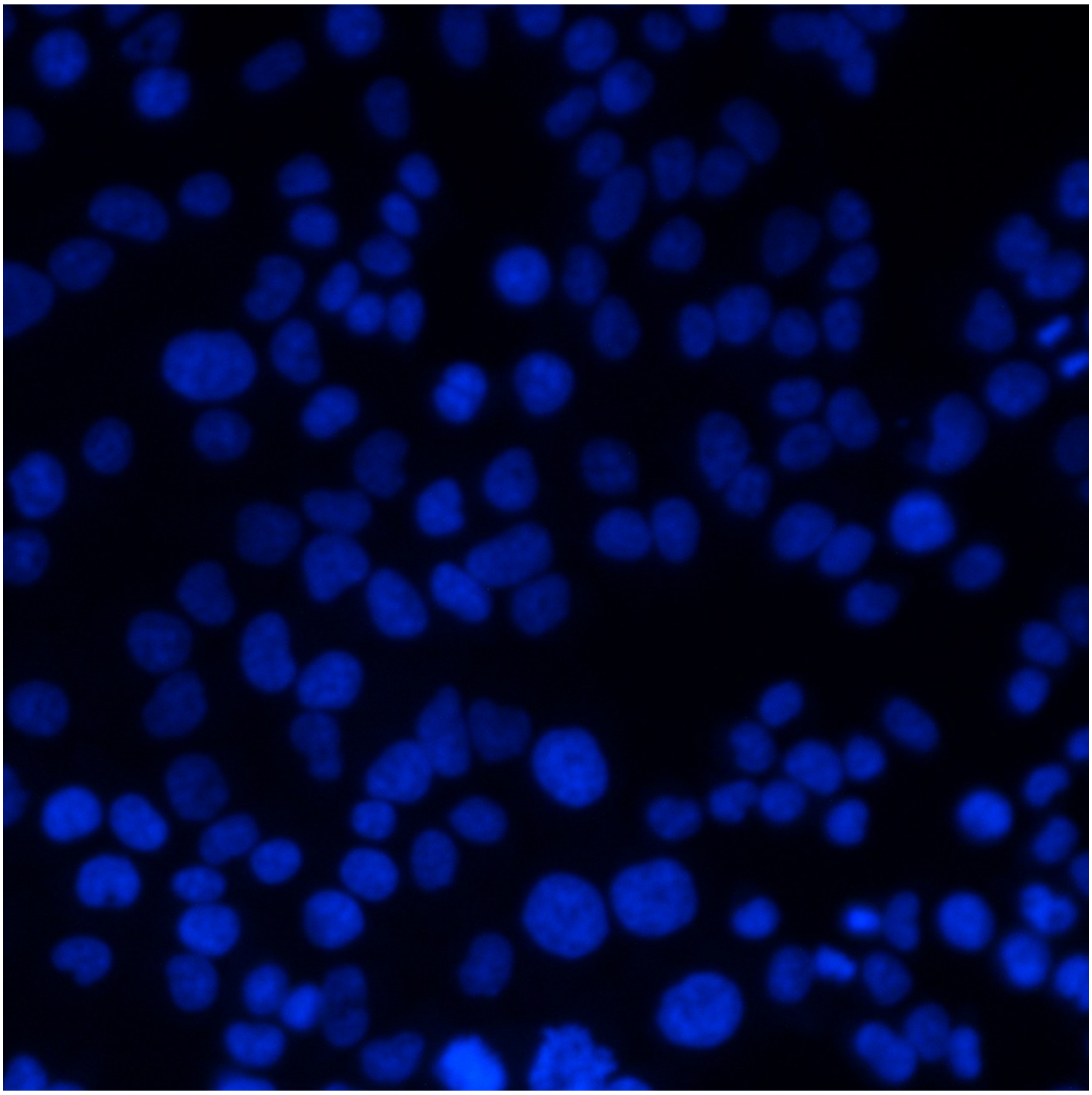

Fig.S5A-HEC-1-A-LV-NC+si-NC-Hoechst

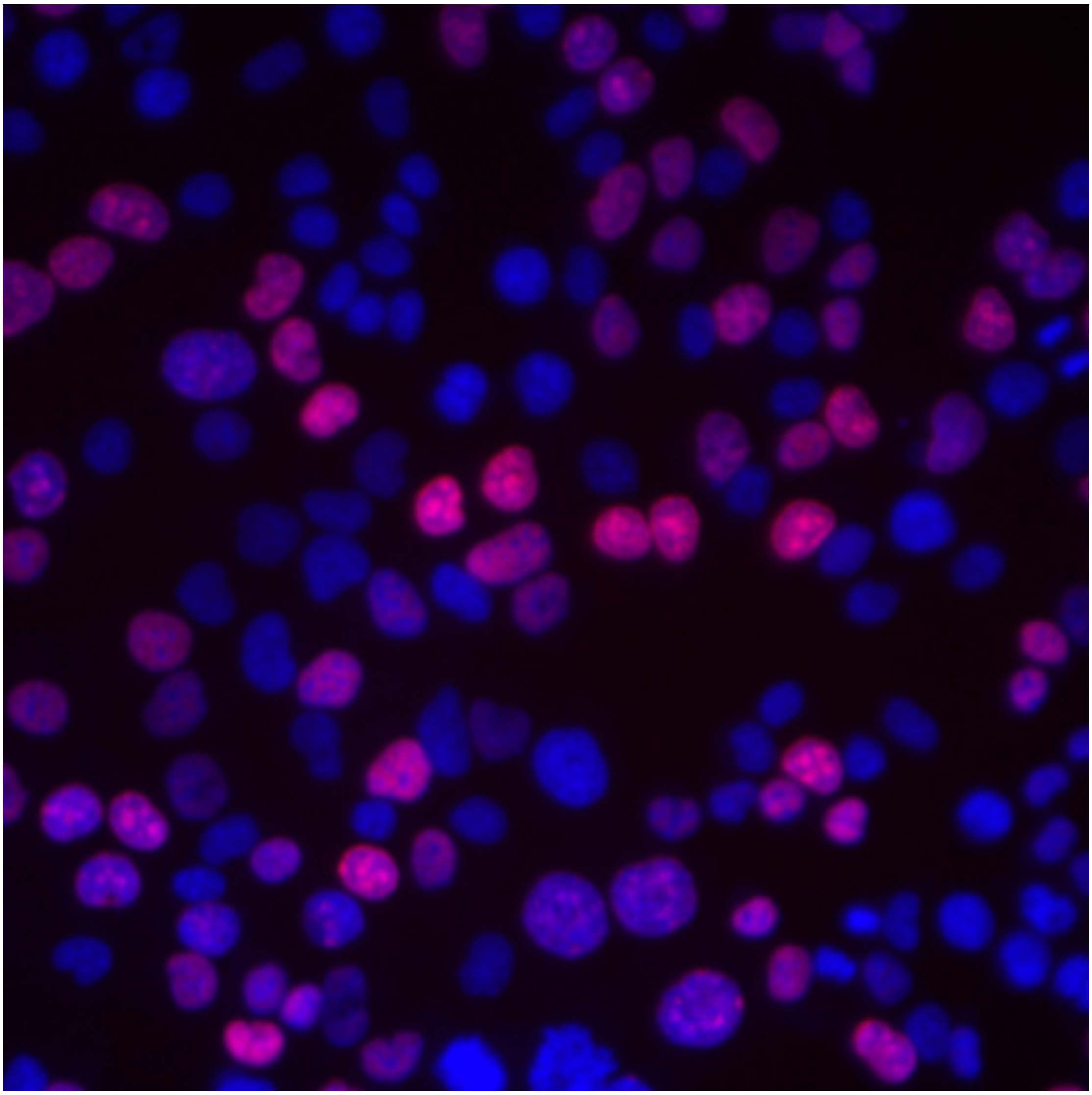

Fig.S5A-HEC-1-A-LV-NC+si-NC-Merge

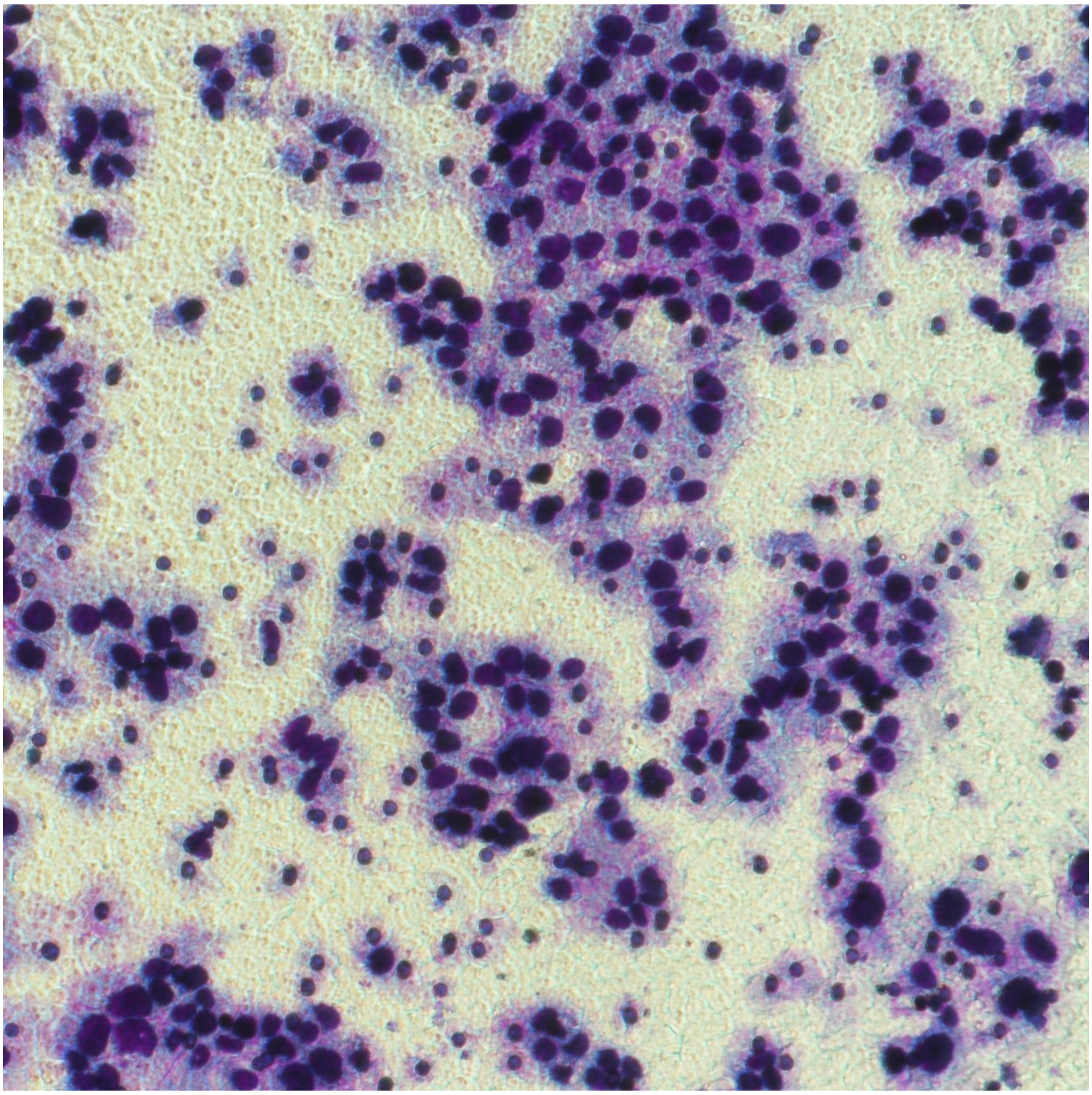

Fig.S5B-HEC-1-A-invasion-IGF2BP3-OE+si-NC

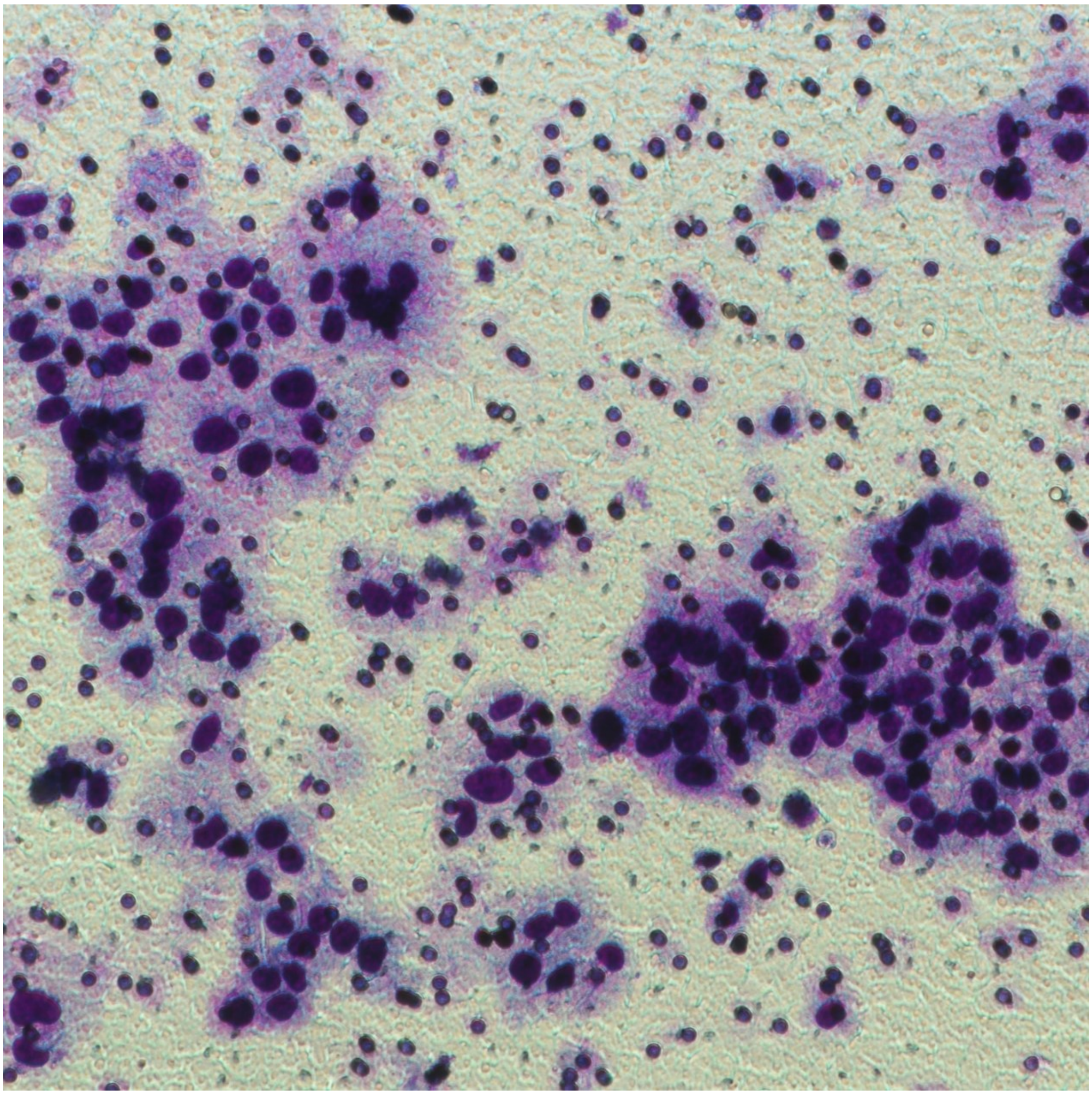

Fig.S5B-HEC-1-A-invasion-IGF2BP3-OE-si-E2F3

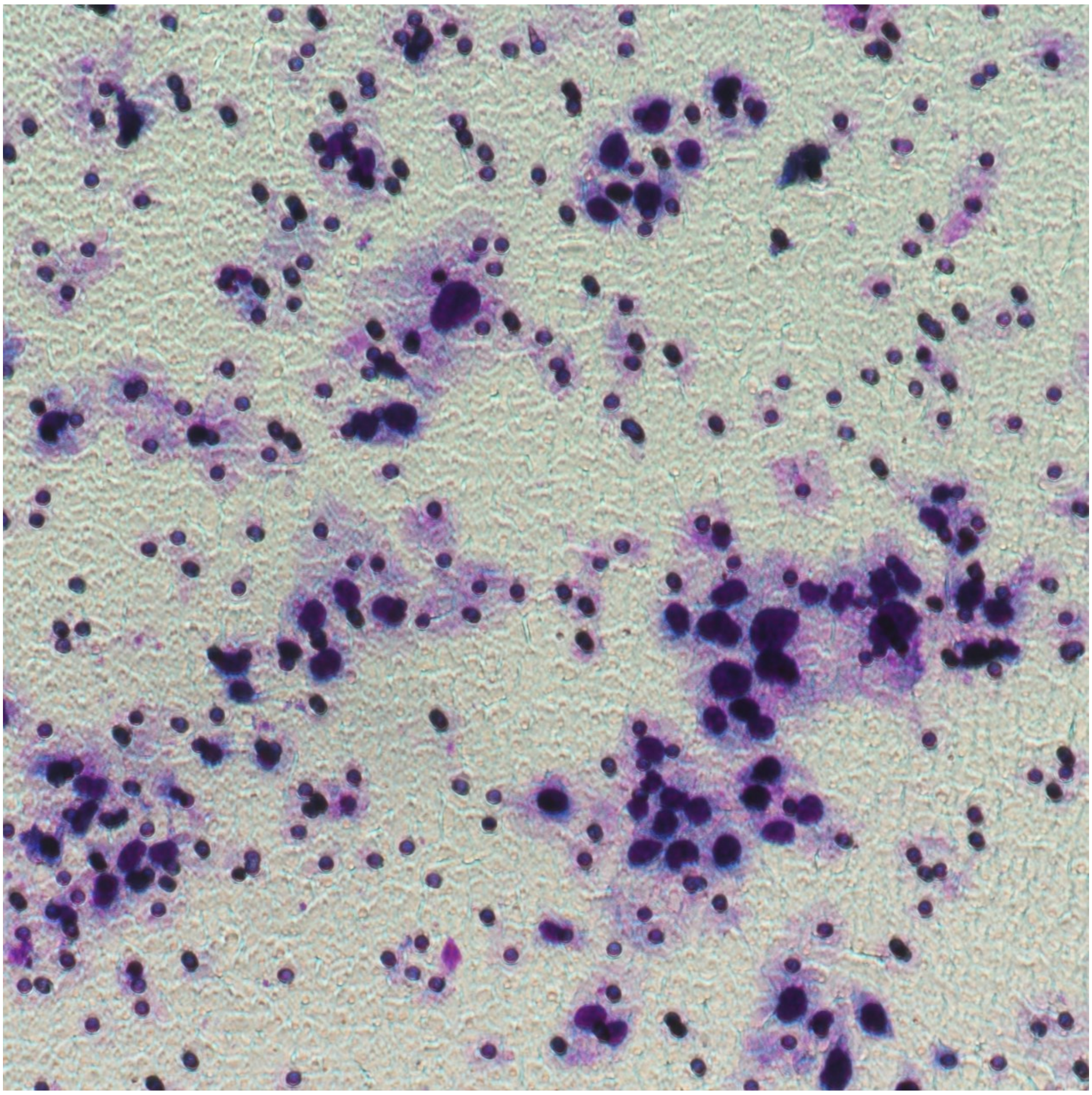

Fig.S5B-HEC-1-A-invasion-LV-NC+si-E2F3

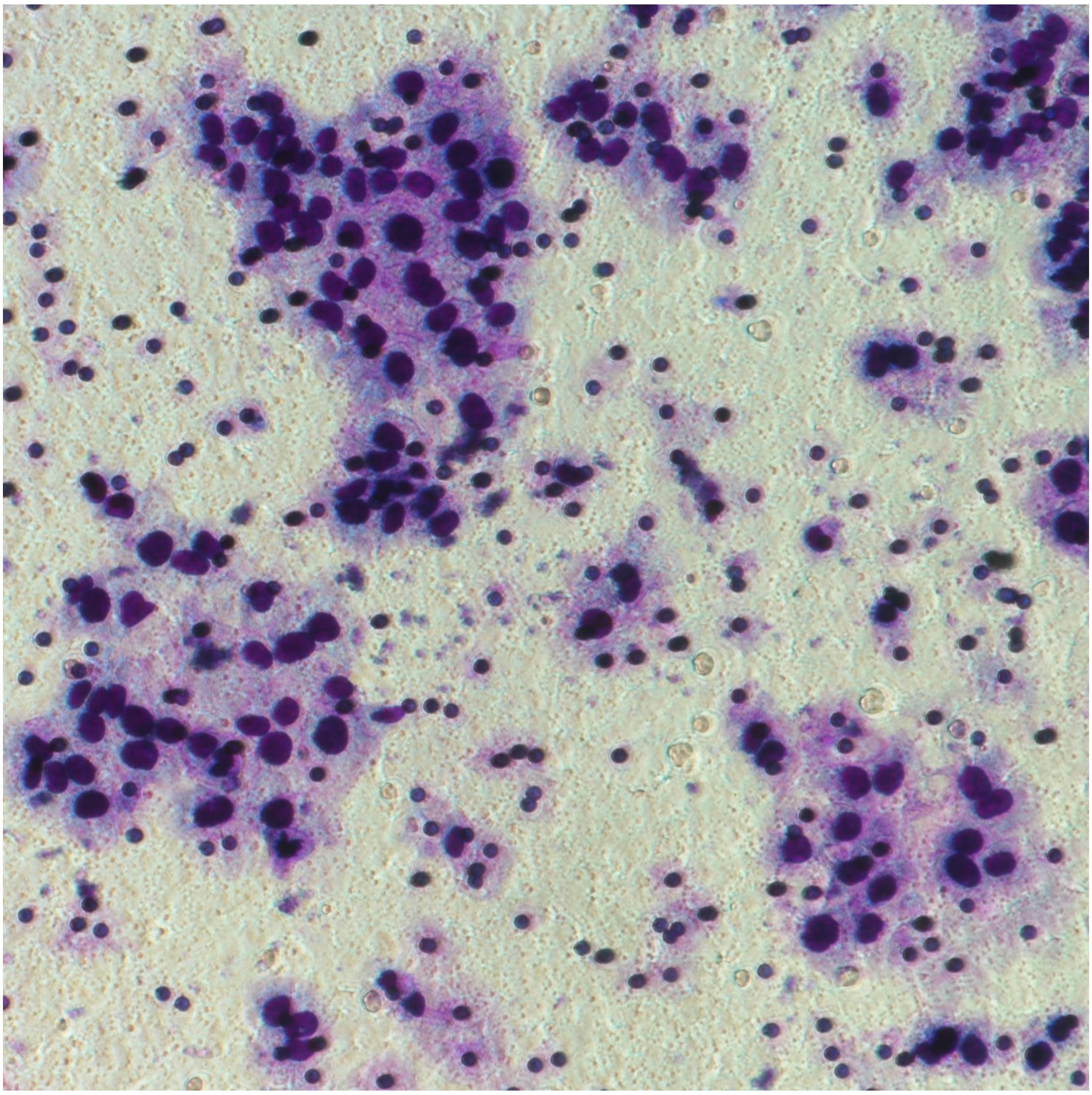

Fig.S5B-HEC-1-A-invasion-LV-NC+si-NC

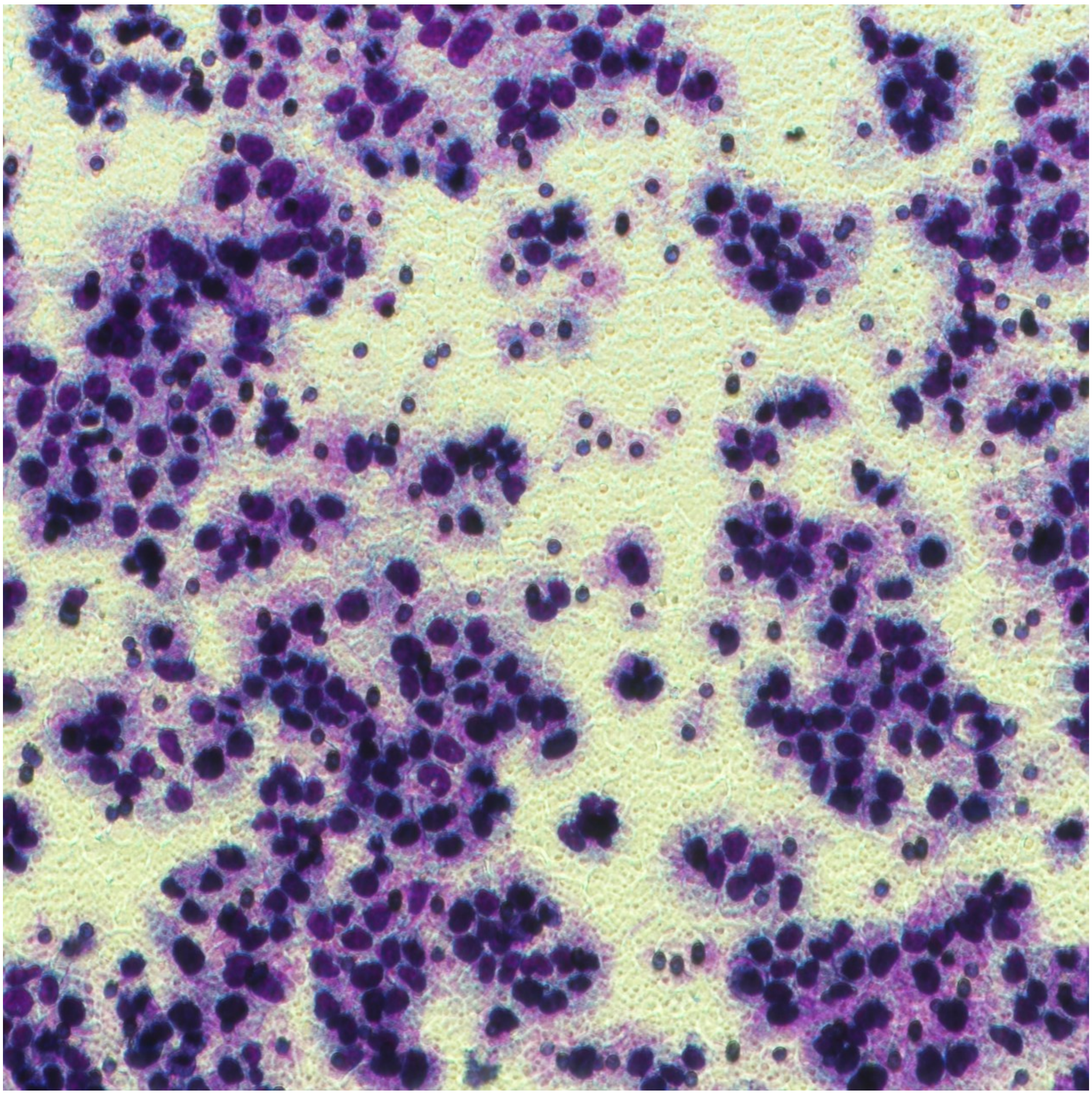

Fig.S5B-HEC-1-A-migration-IGF2BP3-OE+sh-NC

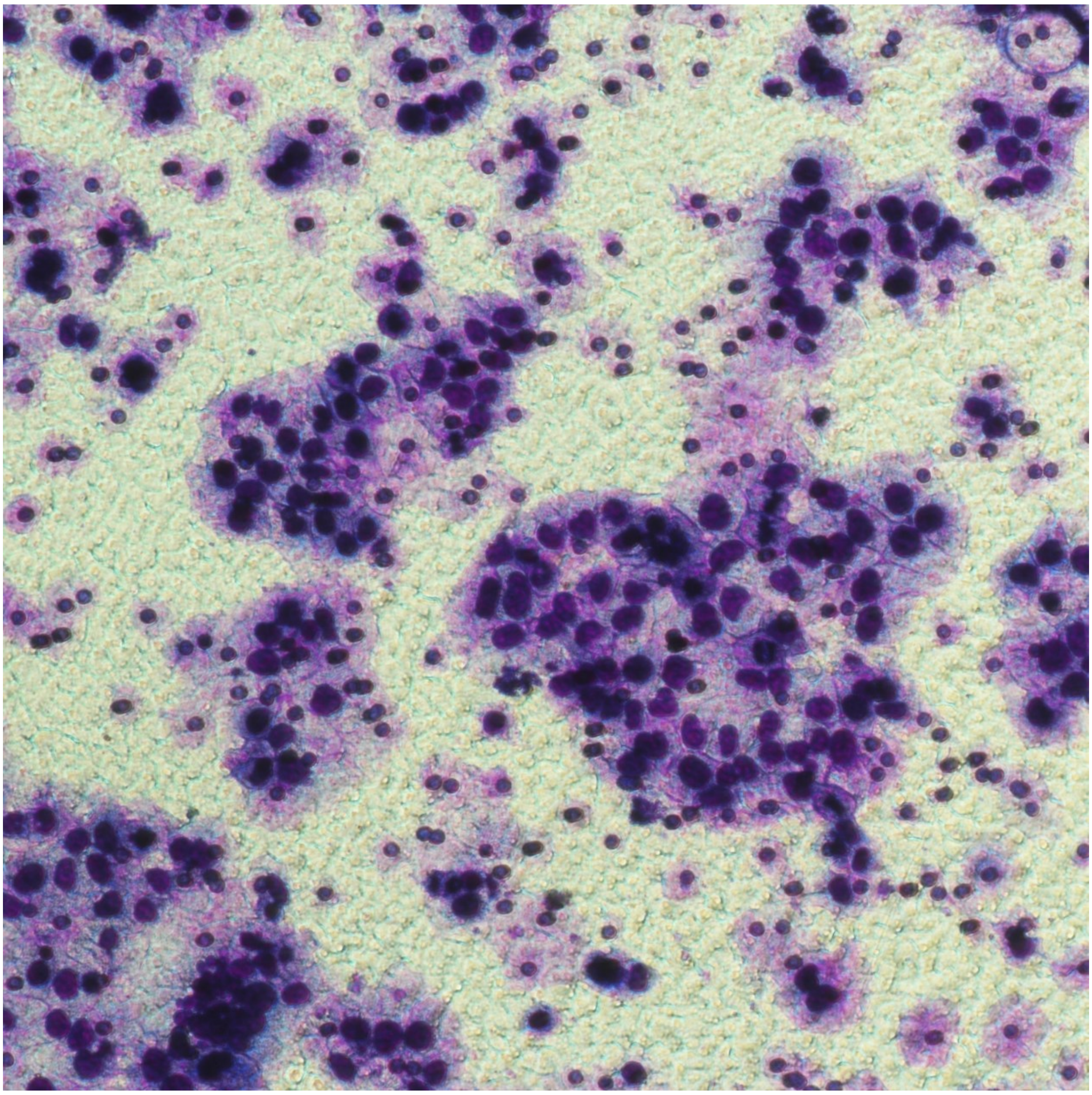

Fig.S5B-HEC-1-A-migration-IGF2BP3-OE+si-E2F3

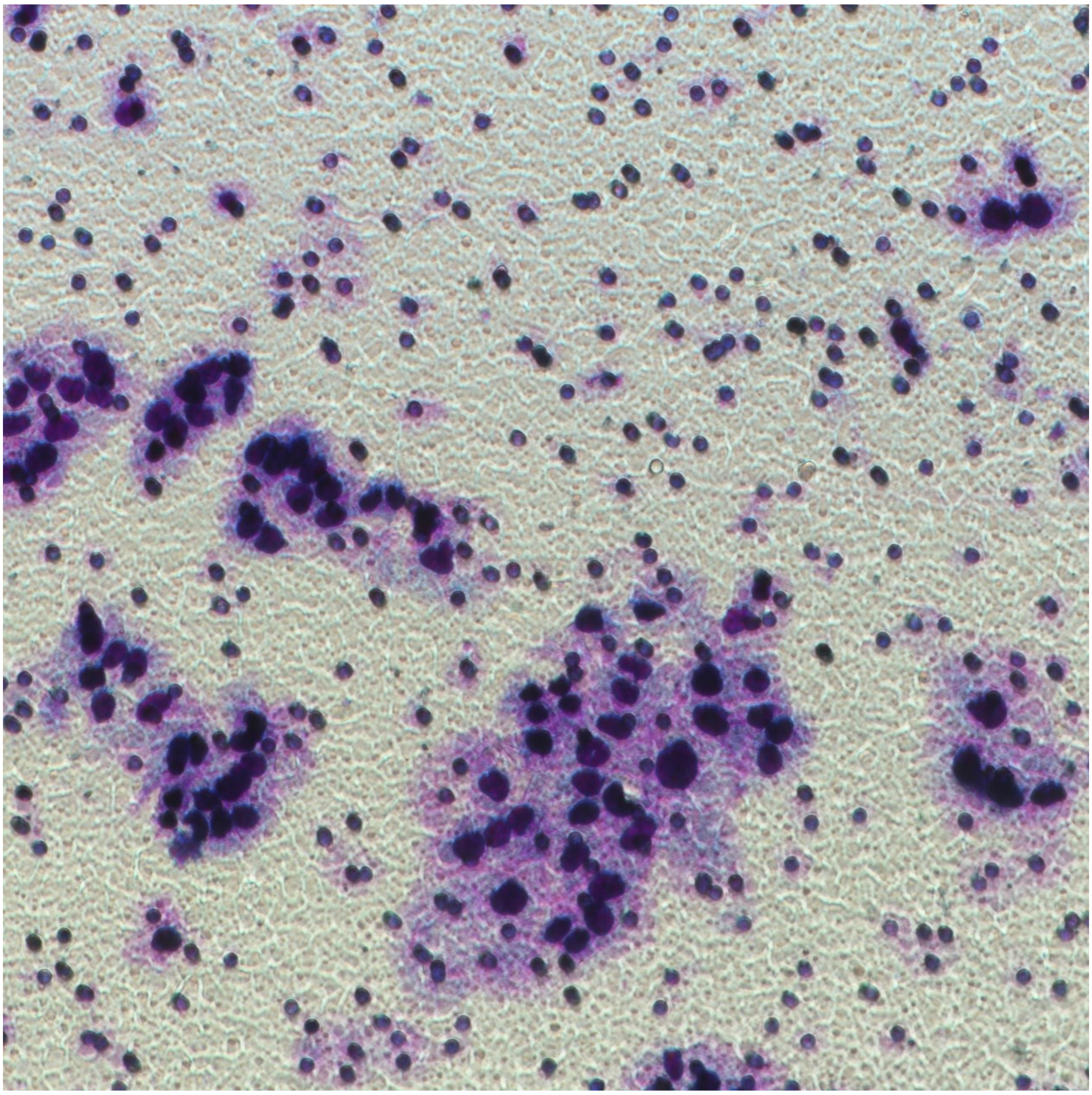

Fig.S5B-HEC-1-A-migration-LV-NC+si-E2F3

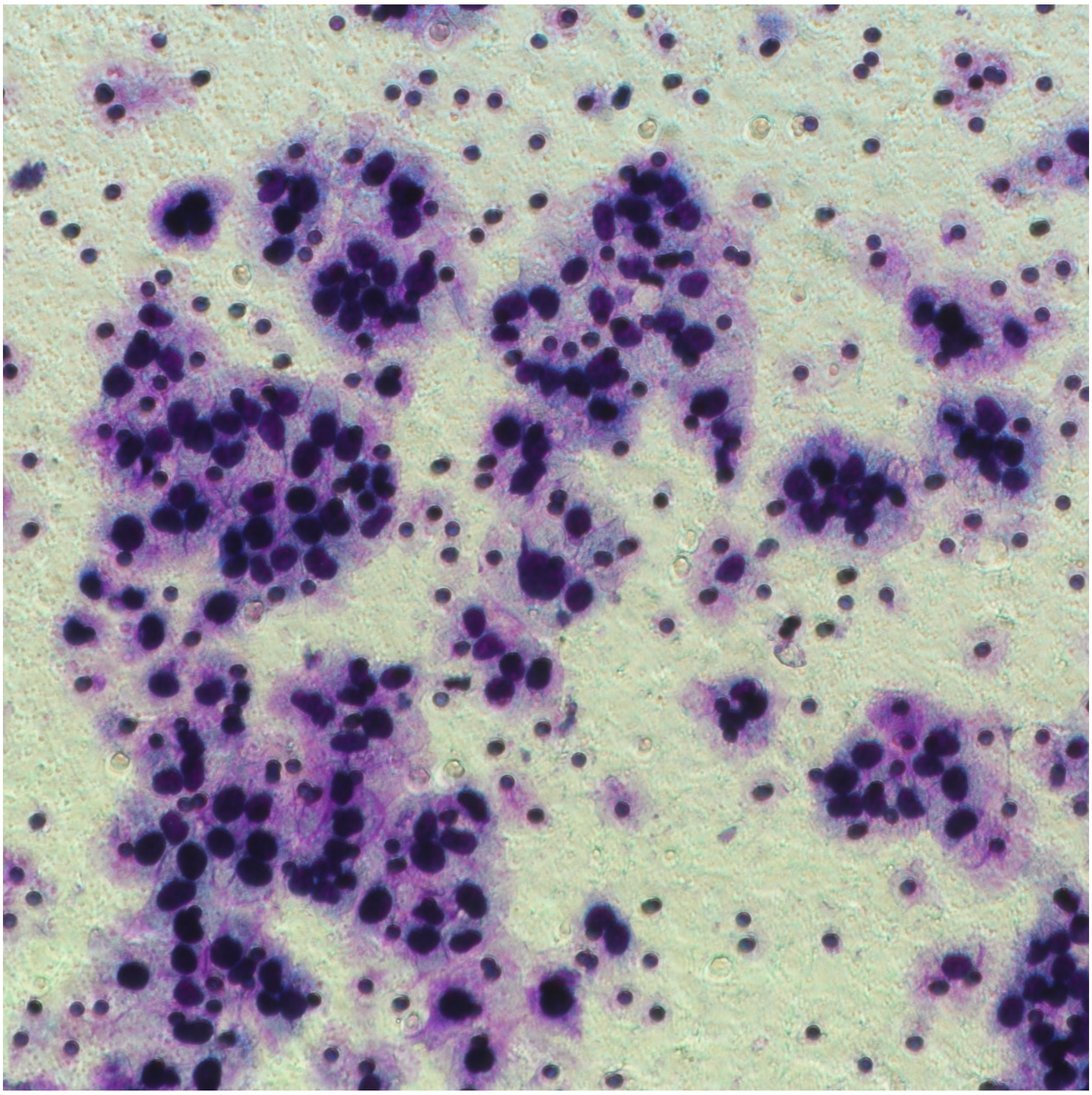

Fig.S5B-HEC-1-A-migration-LV-NC+si-NC
