# Supplementary material for: IGF2BP3 enhances the mRNA stability of E2F3 by interacting with LINC00958 to promote endometrial carcinoma progression
Source: Cell Death Discov. 2022 Jun 8;8:279. doi: 10.1038/s41420-022-01045-x (PMC9177600; doi:10.1038/s41420-022-01045-x)

**Supplemental Material:**

**The full length uncropped original western blots used in the main text**

Fig S1.B Ishikawa–IGF2BP3–sh–NC/sh–IGF2BP3–1/ sh–IGF2BP3–3


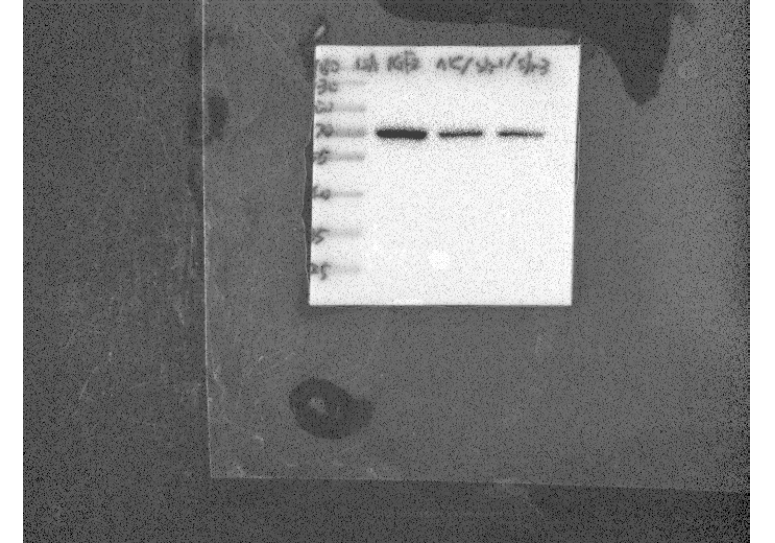


Fig S1.B Ishikawa–GAPDH–sh–NC/sh–IGF2BP3–1/ sh–IGF2BP3–3


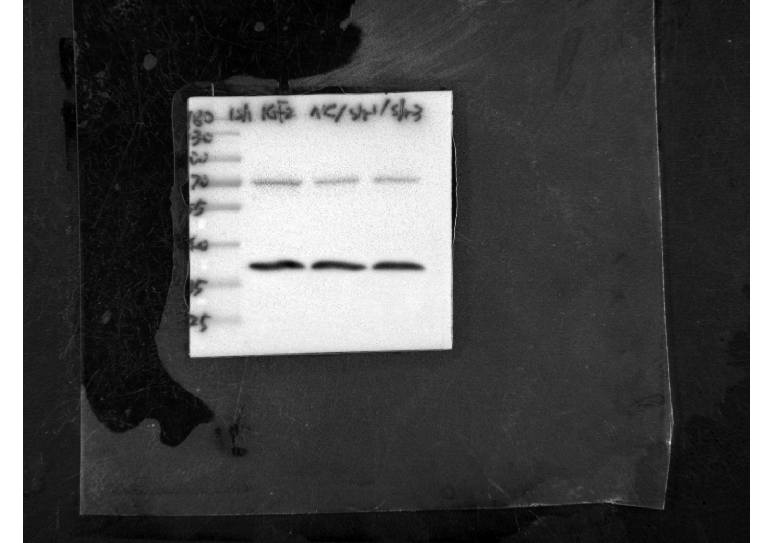


Fig S1.B HEC–1–A–IGF2BP3–sh–NC/sh–IGF2BP3–1/ sh–IGF2BP3–3


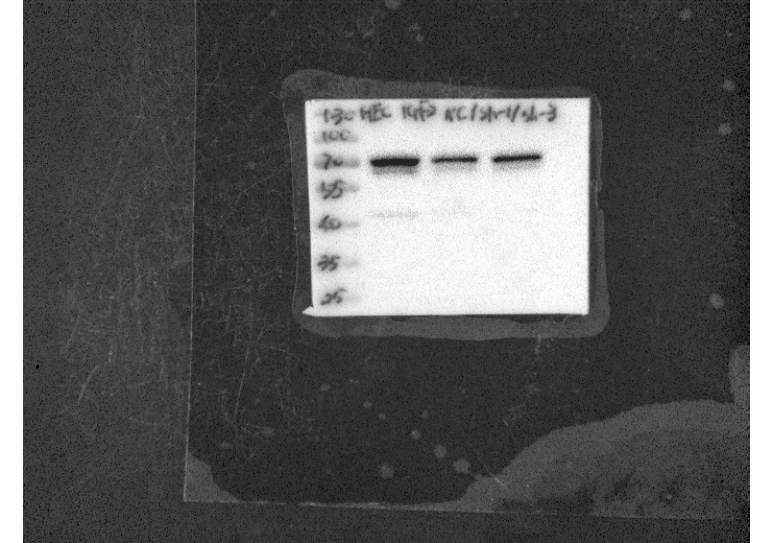


Fig S1.B HEC–1–A–GAPDH–sh–NC/sh–IGF2BP3–1/ sh–IGF2BP3–3


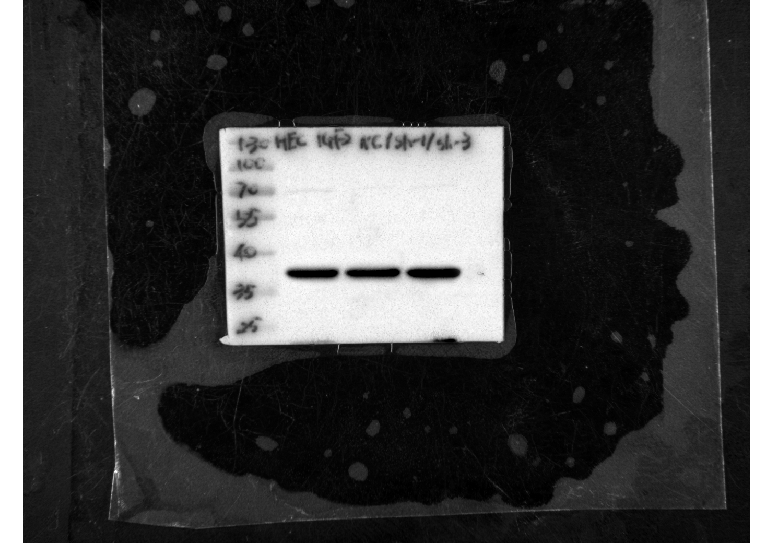


Fig S1.C Ishikawa–IGF2BP3–LV–NC/IGF2BP3–OE


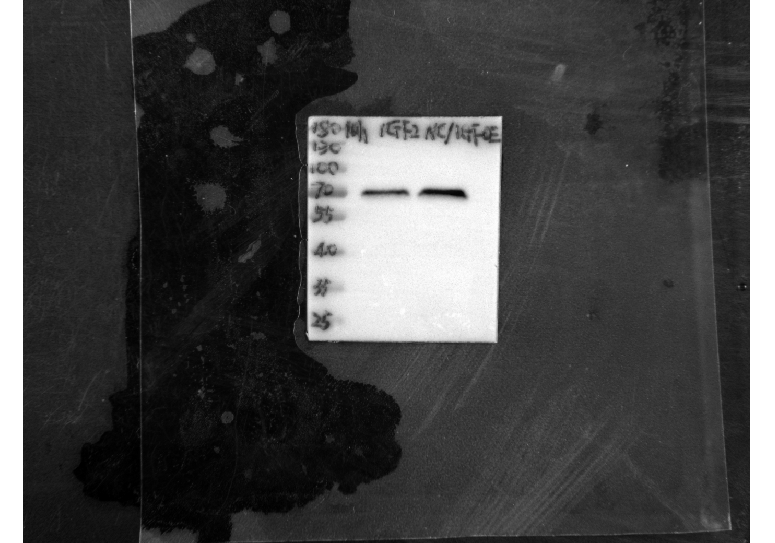


Fig S1.C Ishikawa–GAPDH–LV–NC/IGF2BP3–OE


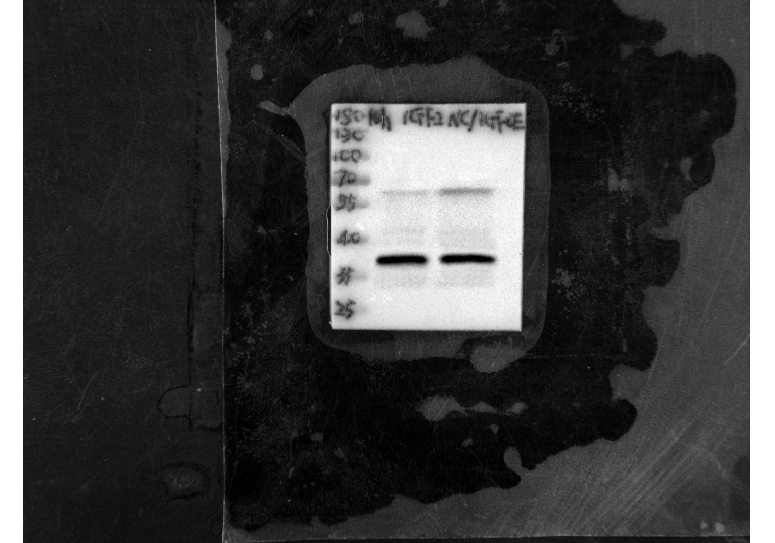


Fig S1.C HEC–1–A–IGF2BP3–LV–NC/IGF2BP3–OE


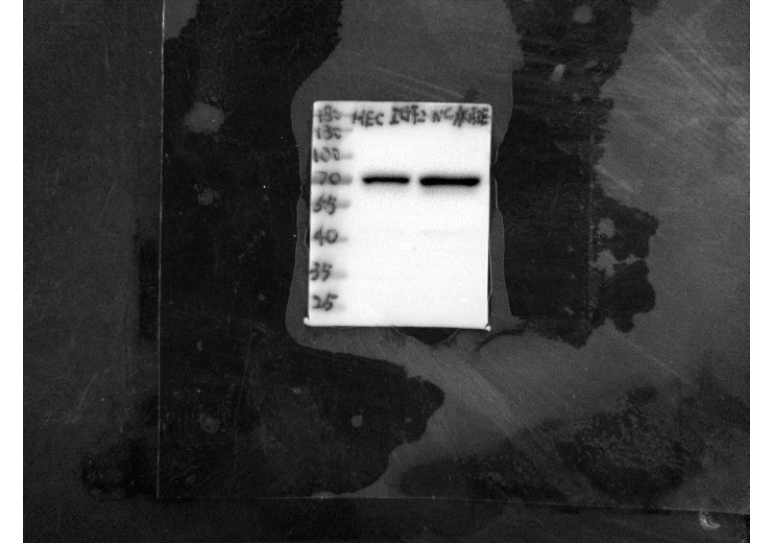


Fig S1.C HEC–1–A–GAPDH–LV–NC/IGF2BP3–OE


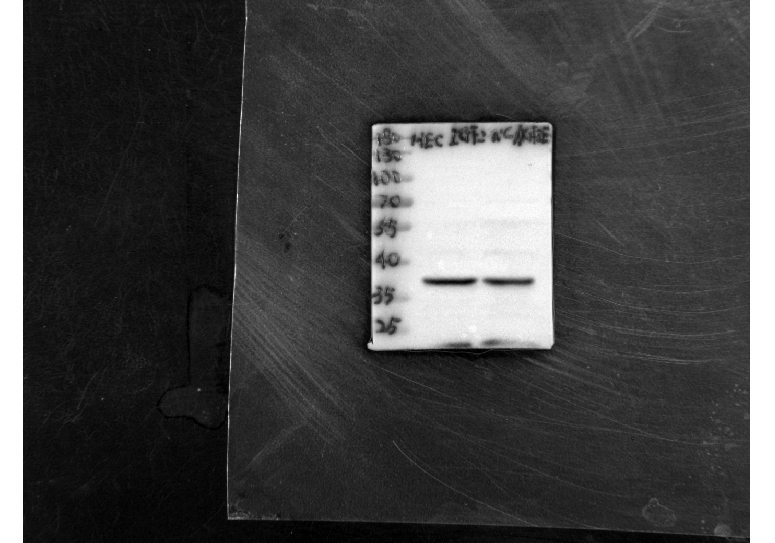


Fig. 3D Ishikawa–IGF2BP3-Input/LINC00958/LacZ


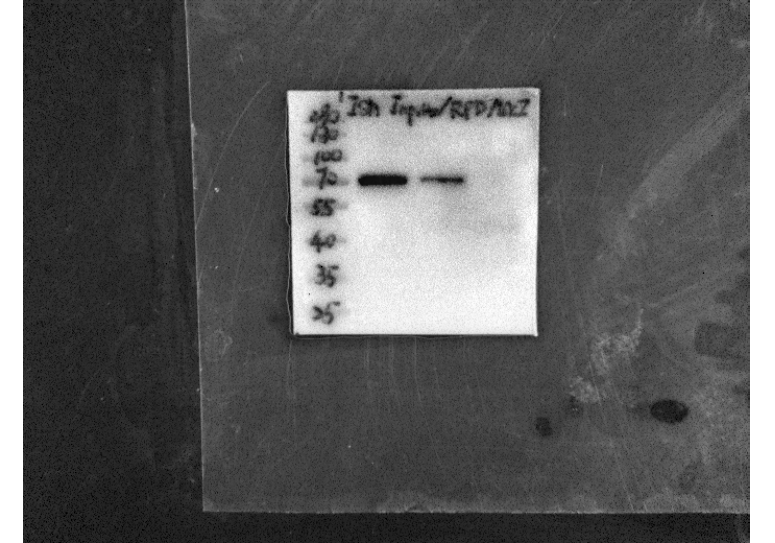


Fig. 3D Ishikawa–GAPDH-Input/LINC00958/LacZ


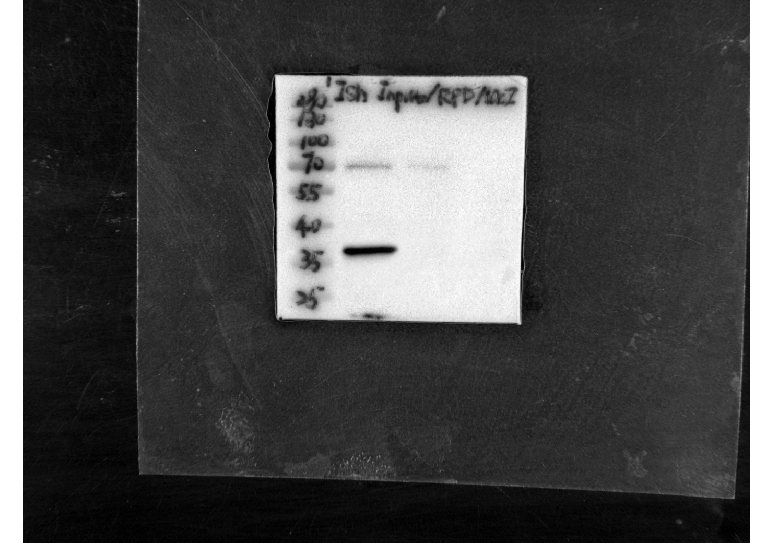


Fig. 3D HEC–1–A–IGF2BP3-Input/LINC00958/LacZ


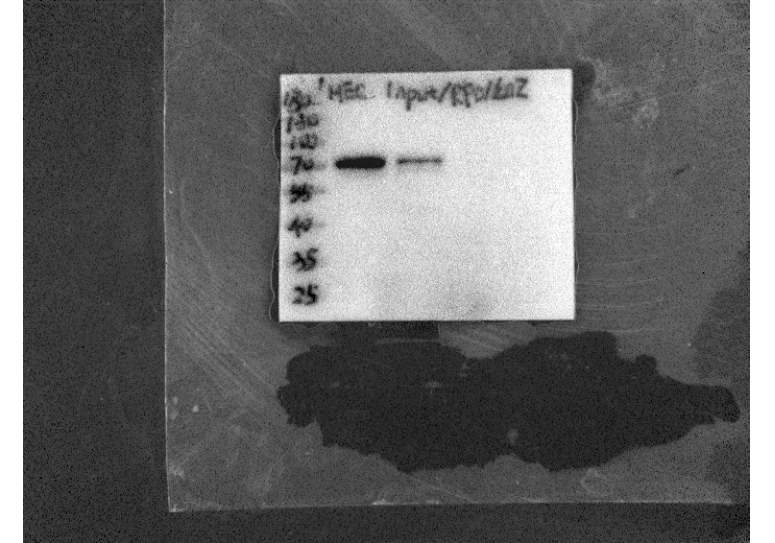


Fig. 3D HEC–1–A–GAPDH-Input/LINC00958/LacZ


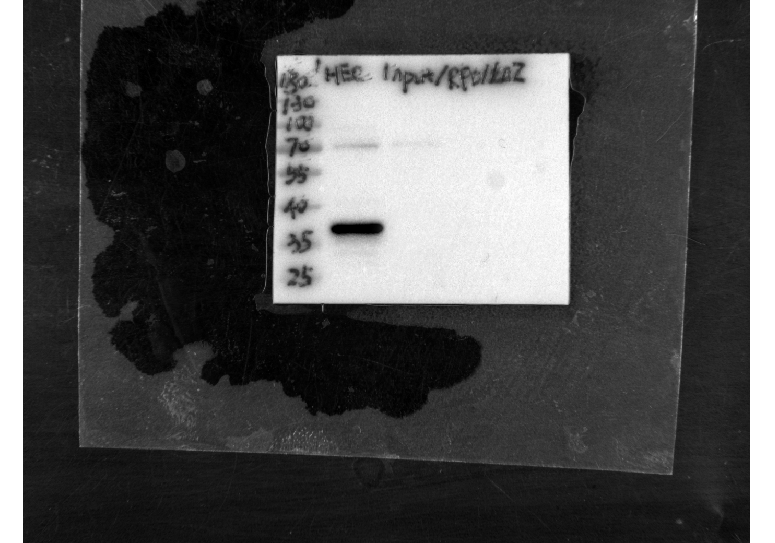


Fig. 3I Ishikawa–IGF2BP3–sh–NC/sh–LINC00958–1/sh–LINC00958–2

Ishikawa–GAPDH–sh–NC/sh–LINC00958–1/sh–LINC00958–2


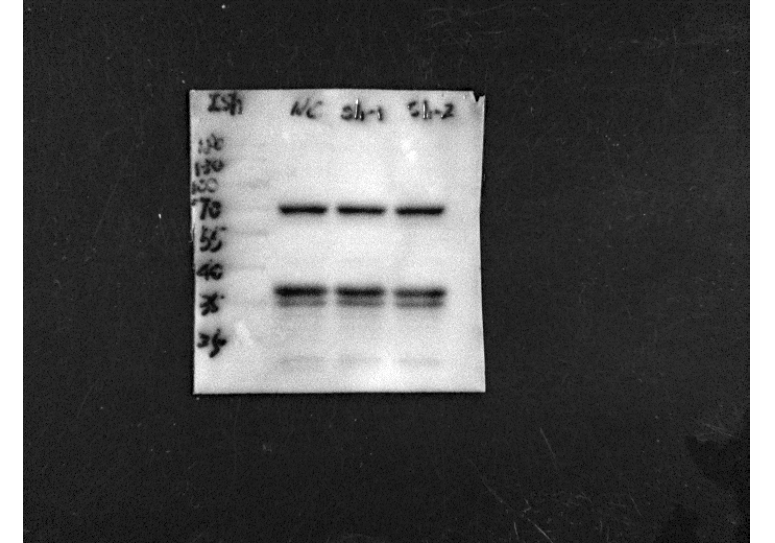


Fig. 3I HEC–1–A–IGF2BP3–sh–NC/sh–LINC00958–1/sh–LINC00958–2

HEC–1–A–GAPDH–sh–NC/sh–LINC00958–1/sh–LINC00958–2


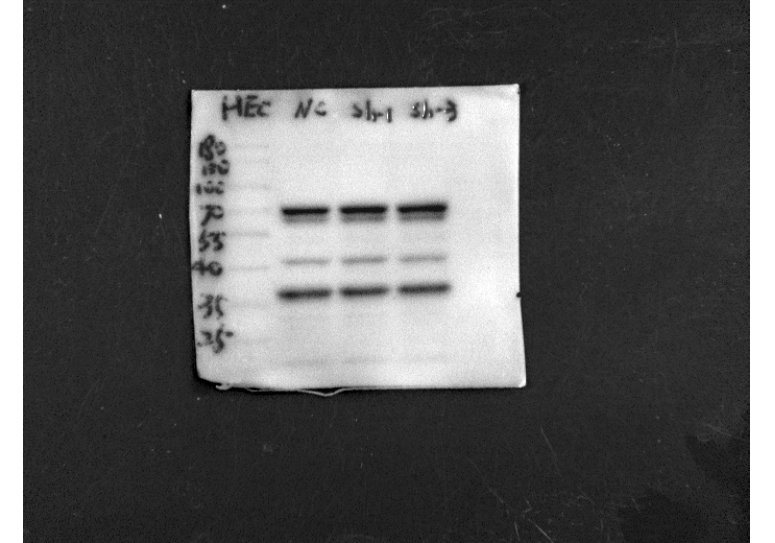


Fig S2 C Ishikawa–IGF2BP3–LV–NC/LINC00958–OE


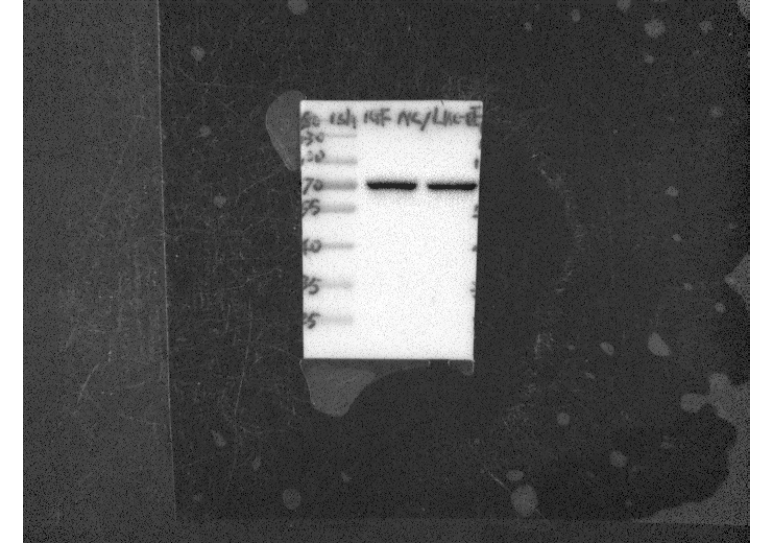


Fig S2 C Ishikawa–GAPDH–LV–NC/LINC00958–OE


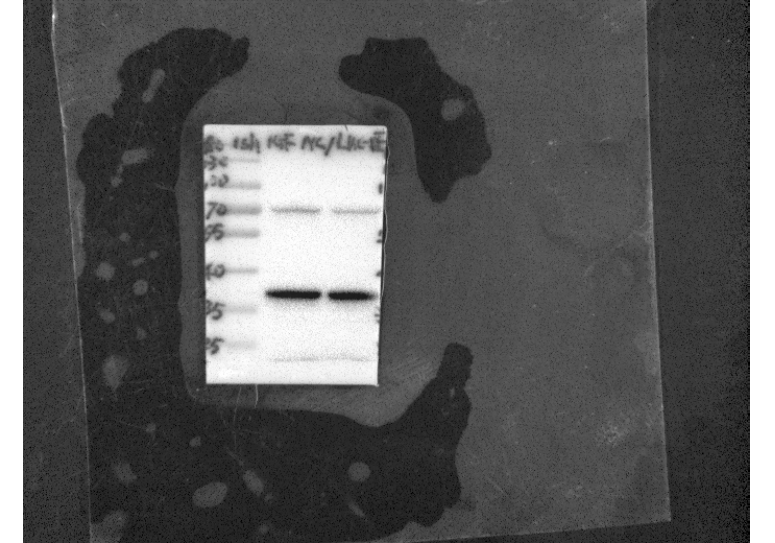


Fig S2 C HEC–1–A–IGF2BP3–LV–NC/LINC00958–OE


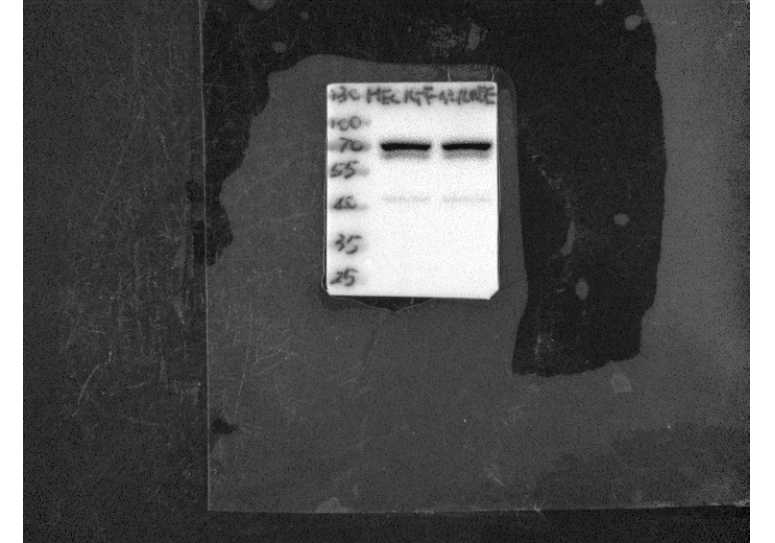


Fig S2 C HEC–1–A–GAPDH–LV–NC/LINC00958–OE


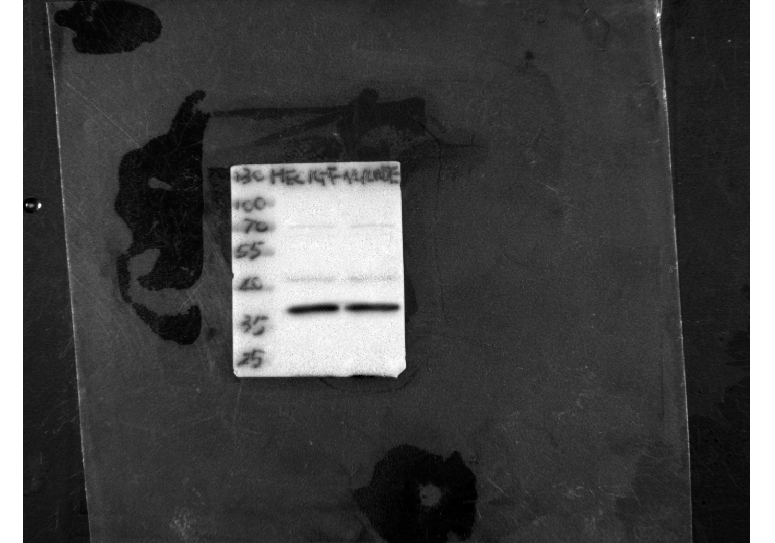


Fig. 5H Ishikawa–E2F3–sh–NC/sh–LINC00958–1/sh–LINC00958–2


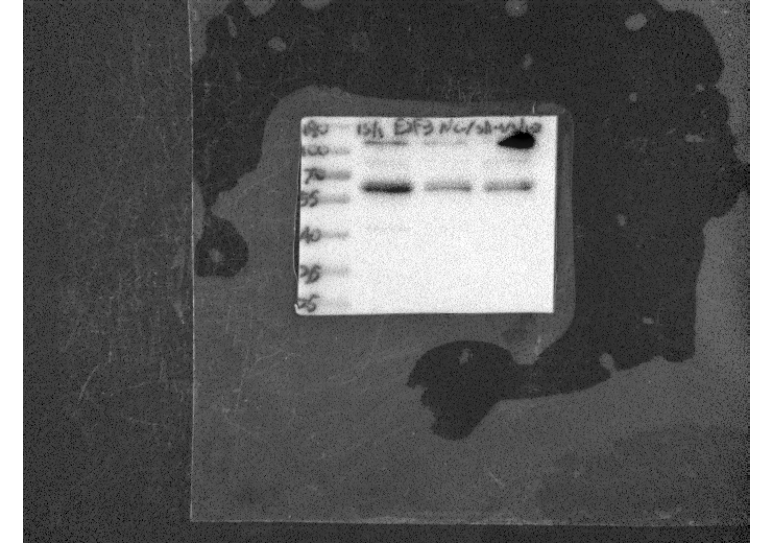


Fig. 5H Ishikawa–GAPDH–sh–NC/sh–LINC00958–1/sh–LINC00958–2


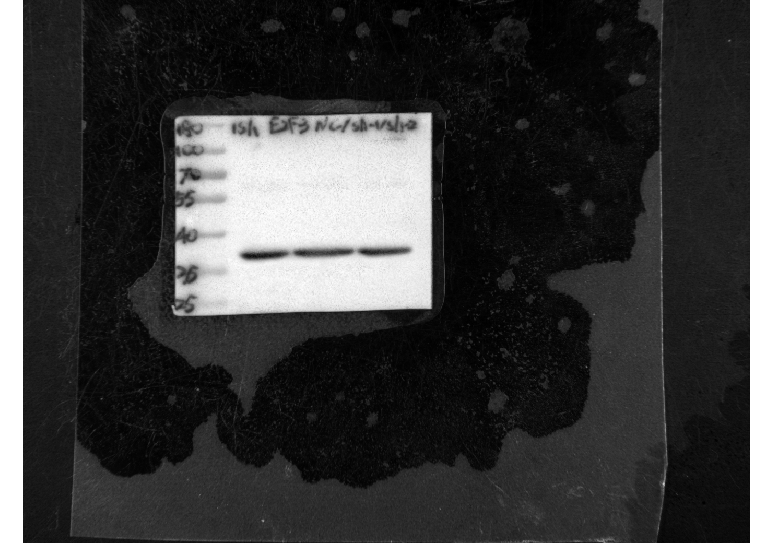


Fig. 5H HEC–1–A–E2F3–sh–NC/sh–LINC00958–1/sh–LINC00958–2


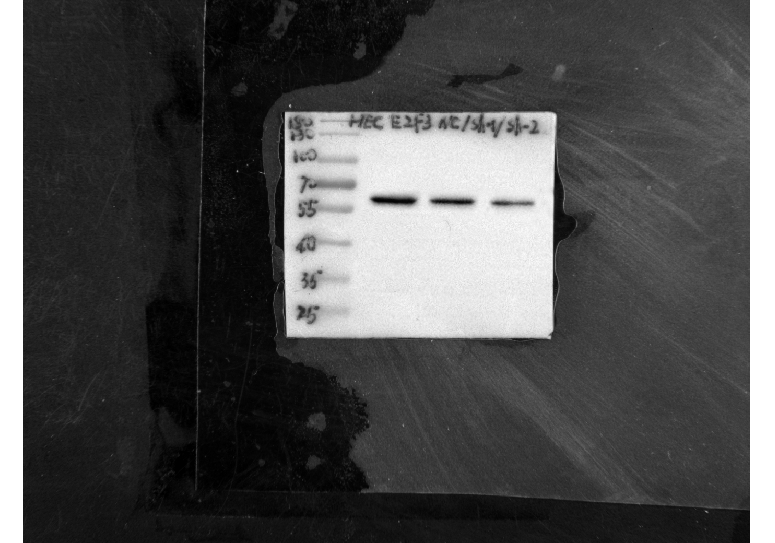


Fig. 5H HEC–1–A–GAPDH–sh–NC/sh–LINC00958–1/sh–LINC00958–2


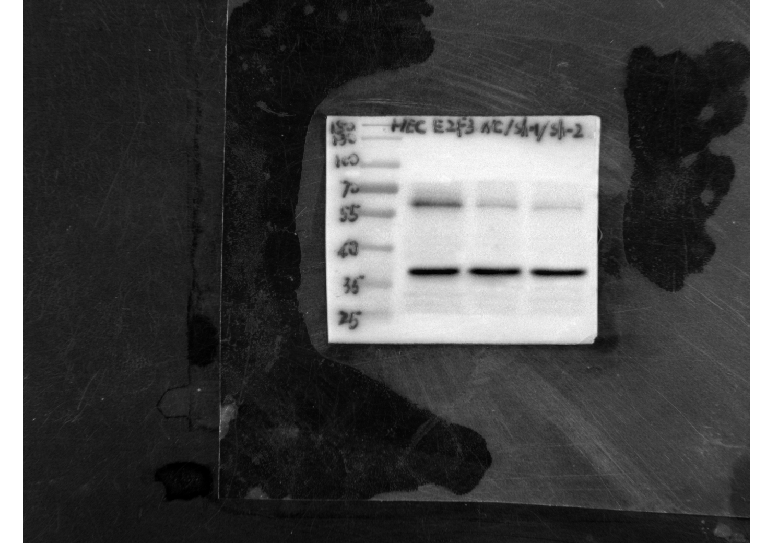


Fig. 5H Ishikawa–E2F3–LV–NC/LINC00958–OE


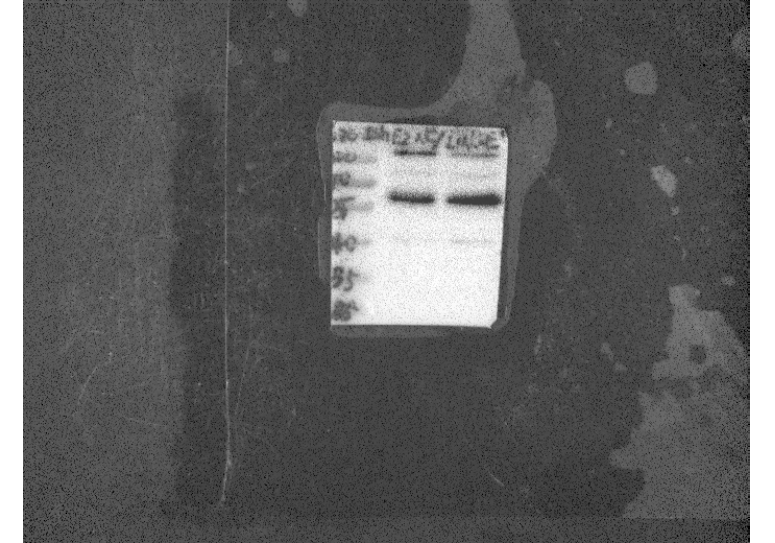


Fig. 5H Ishikawa–GAPDH–LV–NC/LINC00958–OE


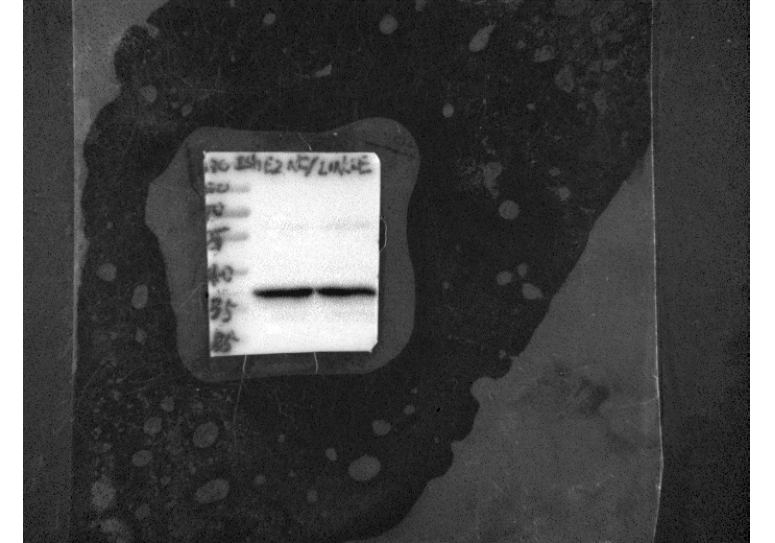


Fig. 5H HEC–1–A–E2F3–LV–NC/LINC00958–OE


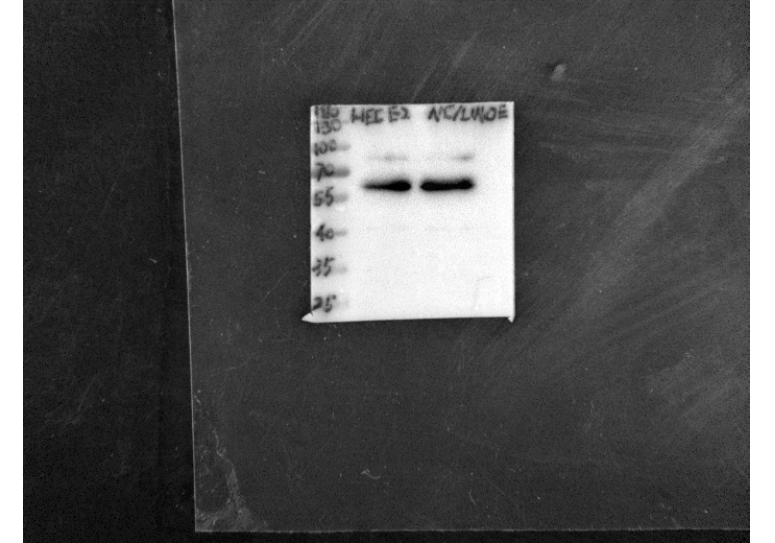


Fig. 5H HEC–1–A–GAPDH–LV–NC/LINC00958–OE


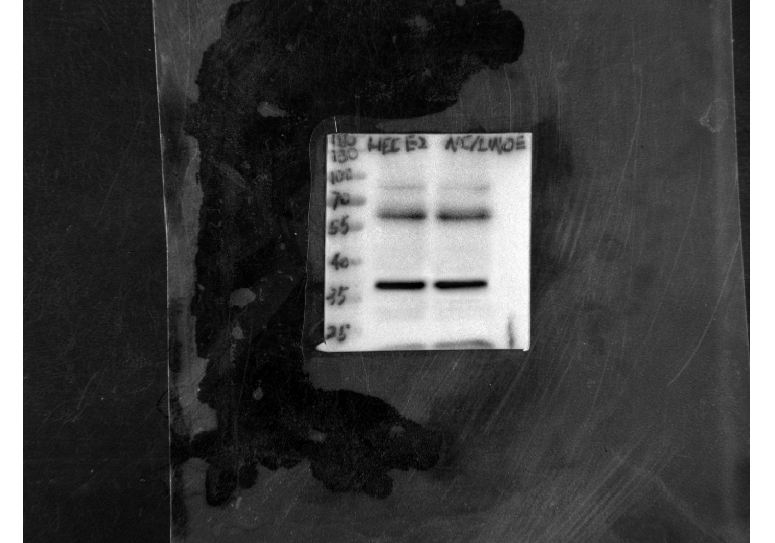


Fig. 5I Ishikawa–E2F3–sh–NC/sh–IGF2BP3–1/sh–IGF2BP3–3


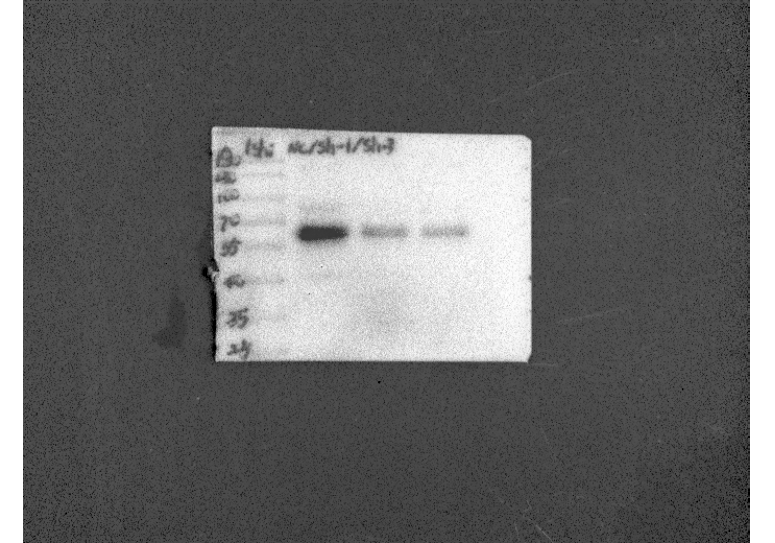


Fig. 5I Ishikawa–GAPDH–sh–NC/sh–IGF2BP3–1/sh–IGF2BP3–3


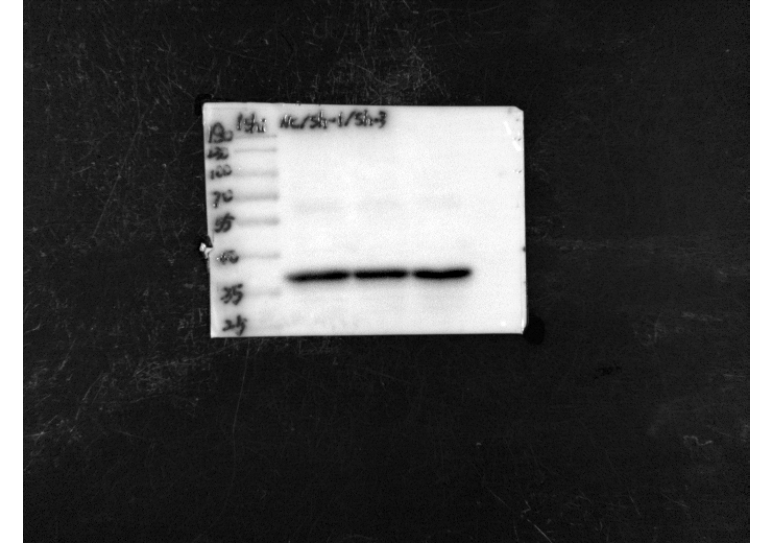


Fig. 5I HEC–1–A–E2F3–sh–NC/sh–IGF2BP3–1/sh–IGF2BP3–3


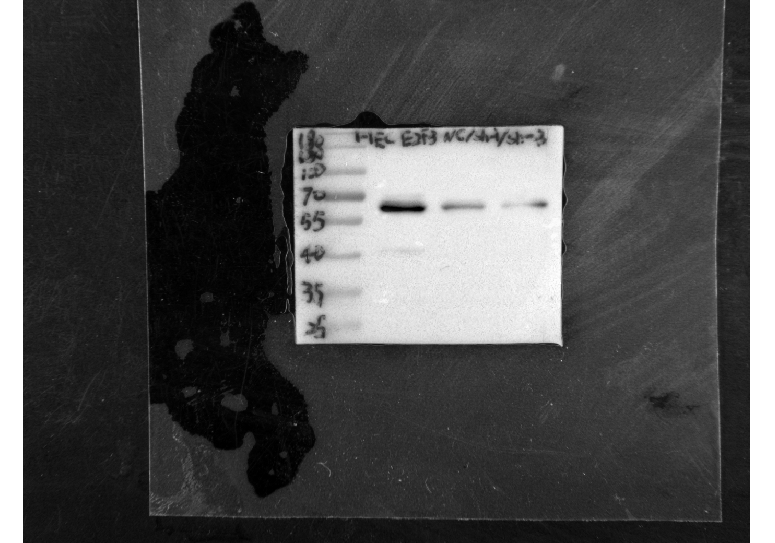


Fig. 5I HEC–1–A–GAPDH–sh–NC/sh–IGF2BP3–1/sh–IGF2BP3–3


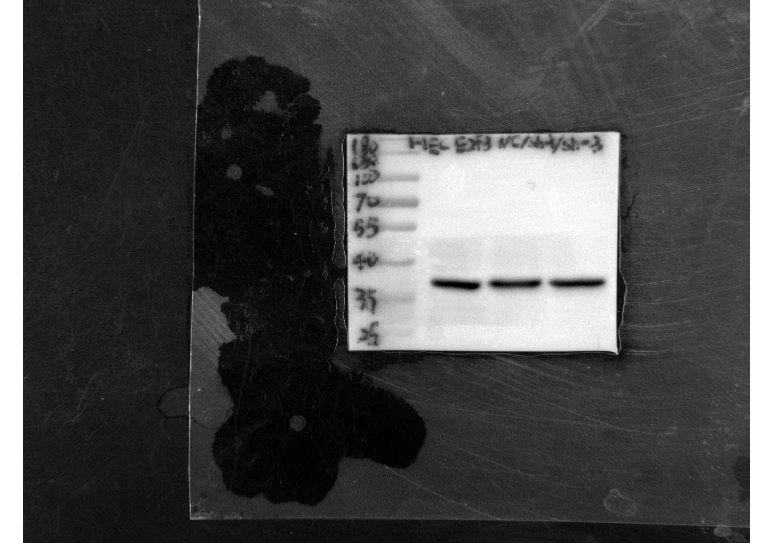


Fig. 5I Ishikawa–E2F3–LV–NC/IGF2BP3–OE


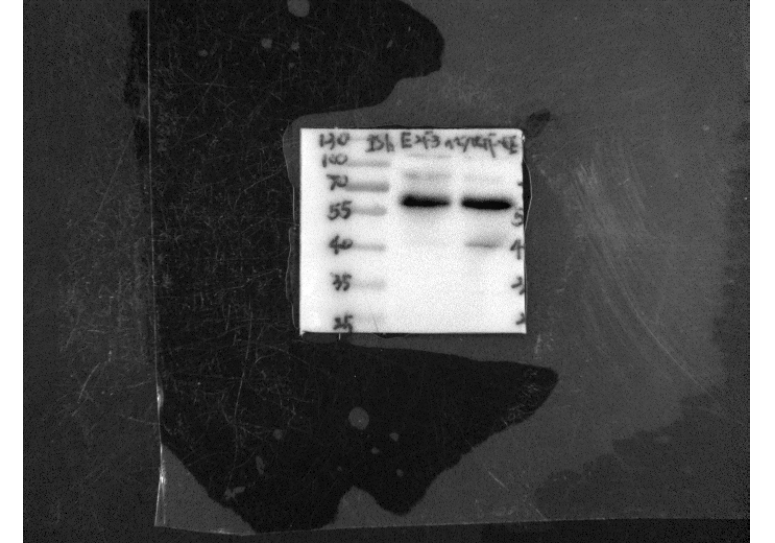


Fig. 5I Ishikawa–GAPDH–LV–NC/IGF2BP3–OE


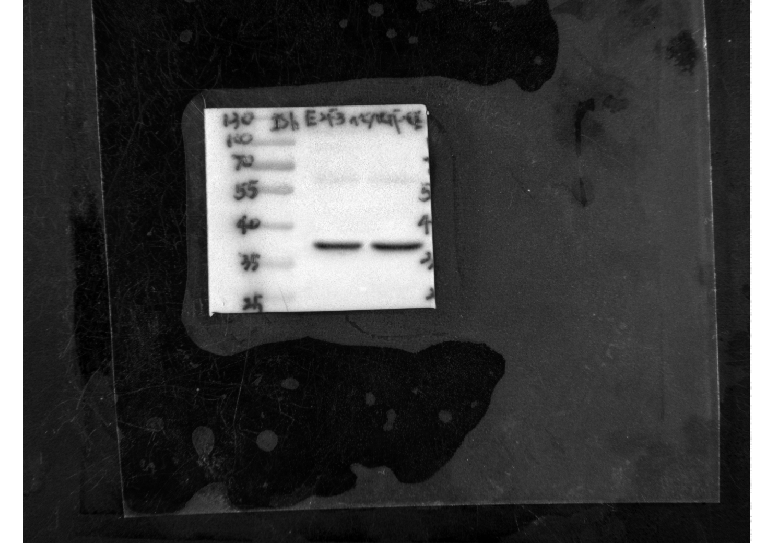


Fig. 5I HEC–1–A–E2F3–LV–NC/IGF2BP3–OE


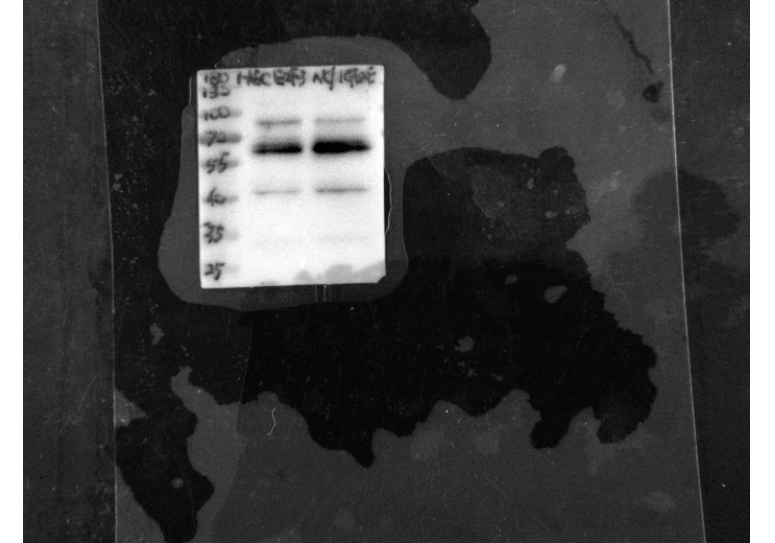


Fig. 5I HEC–1–A–GAPDH–LV–NC/IGF2BP3–OE


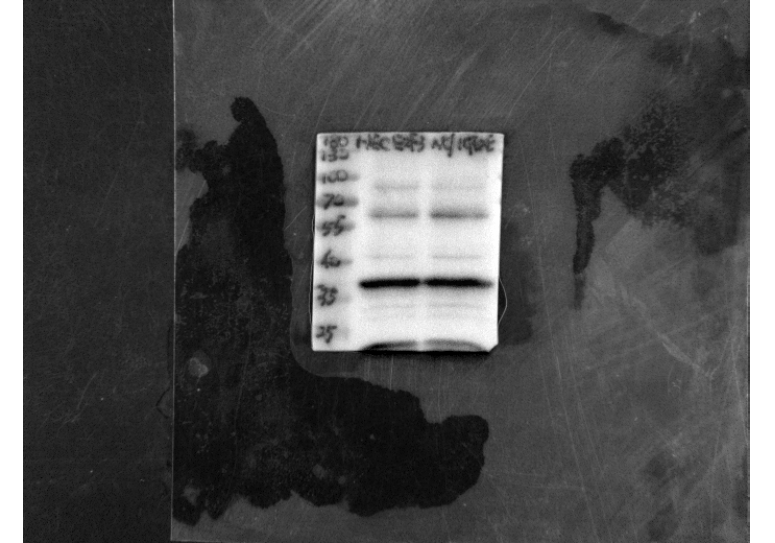


Fig. 5J Ishikawa–E2F3–LV–NC+sh–NC/IGF2BP3–OE+sh–NC/LV–NC+sh–LINC00958/IGF2BP3–OE+sh–LINC00958


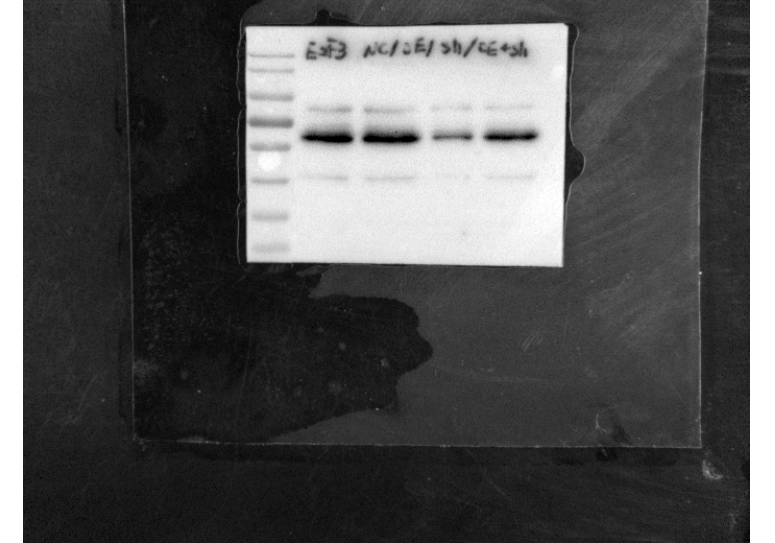


Fig. 5J Ishikawa–GAPDH–LV–NC+sh–NC/IGF2BP3–OE+sh–NC/LV–NC+sh–LINC00958/IGF2BP3–OE+sh–LINC00958


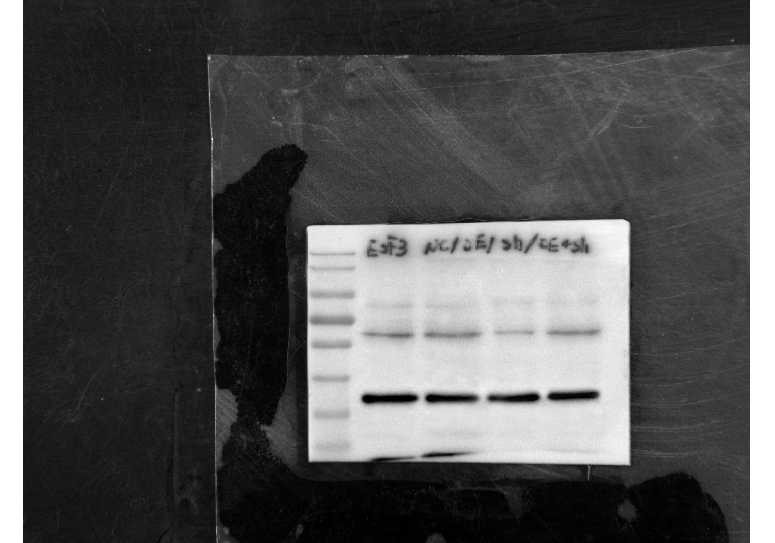


Fig. 5J HEC–1–A–E2F3–LV–NC+sh–NC/IGF2BP3–OE+sh–NC/LV–NC+sh–LINC00958/IGF2BP3–OE+sh–LINC00958


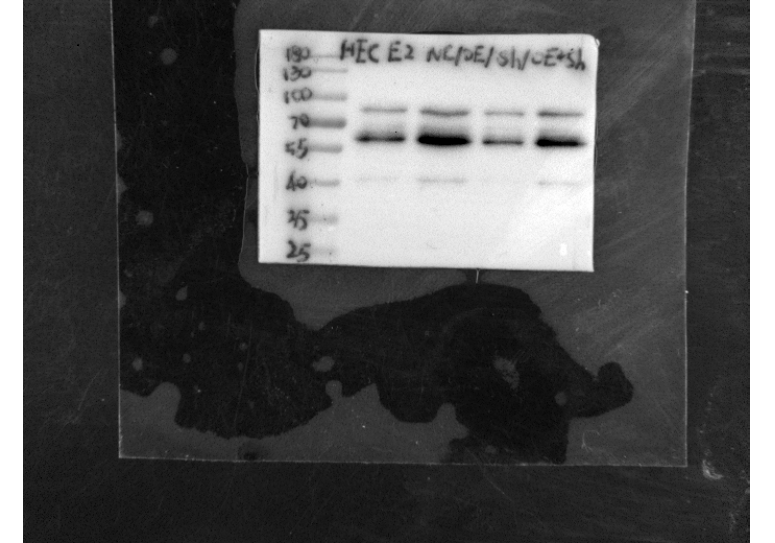


Fig. 5J HEC–1–A–GAPDH–LV–NC+sh–NC/IGF2BP3–OE+sh–NC/LV–NC+sh–LINC00958/IGF2BP3–OE+sh–LINC00958


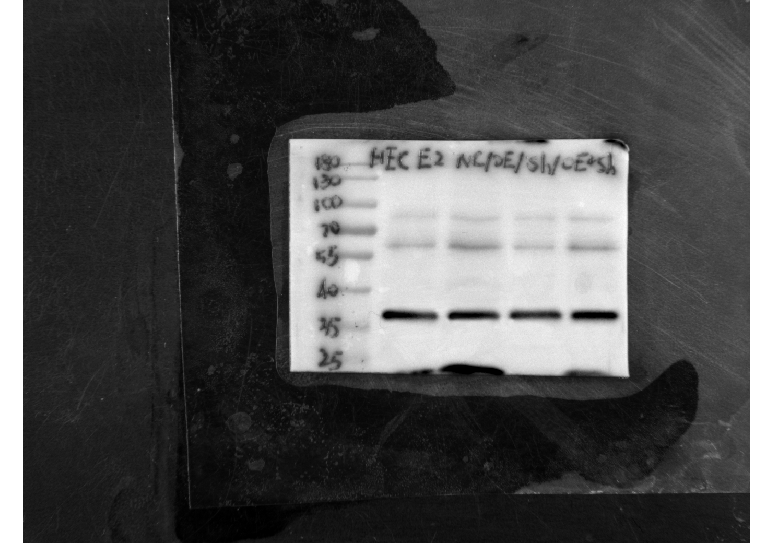

Supplement: Supplementary file 2 — Original western blot pictures [file 41420_2022_1045_MOESM2_ESM.docx]
