# Supplementary material for: IGF2BP3 enhances the mRNA stability of E2F3 by interacting with LINC00958 to promote endometrial carcinoma progression
Source: Cell Death Discov. 2022 Jun 8;8:279. doi: 10.1038/s41420-022-01045-x (PMC9177600; doi:10.1038/s41420-022-01045-x)
Supplement: Supplementary file 3 — Supplementary figure and table legends [file 41420_2022_1045_MOESM3_ESM.docx]

**Fig. S1** Overexpression of IGF2BP3 promoted EC cell progression in vitro. **A** IGF2BP3 mRNA expression was increased after lentivirus infection in EC cells. **B** The protein level of IGF2BP3 was silenced by the lentivirus RNAi system in Ishikawa and HEC-1-A cells. **C** The protein level of IGF2BP3 was enhanced after lentivirus infection. **D** and **E** CCK-8 assays (**D**) and EdU assays (**E**) were conducted to assess the cell proliferation ability in IGF2BP3-overexpressing EC cells. **F** Transwell assays were conducted to measure IGF2BP3-overexpressing EC cell migration and invasion. The quantification results are on the near side of their representative images. **P* < 0.05, ***P* < 0.01, ****P* < 0.001. Data, mean ± S.D. All experiments were independently repeated with at least three replicates.

**Fig. S2** IGF2BP3 could interact with LINC00958 in the cytoplasm of EC cells **A** and **B** The expression levels of LINC00958 after RNAi (**A**) or overexpression (**B**) lentivirus infection in HEC-1-A cells. **C** Western blot assays displayed the levels of IGF2BP3 in LINC00958-overexpressing Ishikawa and HEC-1-A cells. **D** and **E** RT–qPCR assays showed that IGF2BP3 was not regulated in either LINC00958-overexpressing or LINC00958-silenced HEC-1-A cells (**D**) or vice versa (**E**). **F** RNA-FISH coupled to immunofluorescence indicated that LINC00958 also colocalized with IGF2BP3 in HEC-1-A cells (×800). ****P* < 0.001, "ns", no statistical significance. Abbreviation: RNA–FISH: RNA fluorescence in situ hybridization. Data, mean ± S.D. All experiments were independently repeated with at least three replicates.

**Fig. S3** Knockdown of LINC00958 offset the tumor-promoting role of IGF2BP3 in EC. **A** EdU assays showed the proliferation ability of HEC-1-A cells with ectopic expression of IGF2BP3 combined with LINC00958 silencing. **B** Transwell assays indicated the migration and invasion of HEC-1-A cells stably transfected. ***P* < 0.01, ****P* < 0.001. Data, mean ± S.D. All experiments were independently repeated with at least three replicates.

**Fig. S4** Knockdown of E2F3 inhibited EC cell progression in vitro. **A** The mRNA expression level of E2F3 was knocked down by three siRNAs in Ishikawa and HEC-1-A cells. **B** and **C** CCK-8 assays and EdU assays were used to evaluate the cell proliferation ability in E2F3-silenced EC cells. **D** Transwell assays were conducted to assess migration and invasion in E2F3-silenced EC cells. The quantification results are on the near side of their representative images. **P* < 0.05, ***P* < 0.01, ****P* < 0.001. Data, mean ± S.D. All experiments were independently repeated with at least three replicates.

**Fig. S5** IGF2BP3 exerted its tumor-promoting effects via E2F3 in EC. **A** EdU assays showed the proliferation ability of IGF2BP3-overexpressing HEC-1-A cells with E2F3 silencing. **B** Transwell assays indicated migration and invasion in HEC-1-A cells overexpressing IGF2BP3 combined with E2F3-silenced expression. On the right side of representative images are their quantification results. **P* < 0.05, ***P* < 0.01, ****P* < 0.001. Data, mean ± S.D. All experiments were independently repeated with at least three replicates.

**Table S1:** The correlation between clinicopathological characteristics and expression of IGF2BP3 in 53 endometrial neoplasm patients

**Table S2:** The sequences of primers, probes, shRNAs and siRNAs used in this study
