# Supplementary material for: IGF2BP3 enhances the mRNA stability of E2F3 by interacting with LINC00958 to promote endometrial carcinoma progression
Source: Cell Death Discov. 2022 Jun 8;8:279. doi: 10.1038/s41420-022-01045-x (PMC9177600; doi:10.1038/s41420-022-01045-x)
Supplement: Supplementary file 4 — Supplementary Methods [file 41420_2022_1045_MOESM4_ESM.docx]

**Supplementary Methods**

**RNA isolation and RT–qPCR**

We extracted total RNA from tissues and cells with an RNA isolate kit (Vazyme, Nanjing, China). HiScript III RT SuperMix (Vazyme, Nanjing, China) was used to reverse-transcribe total RNA to cDNA according to the manufacturer’s instructions. RT–qPCR was conducted by SYBR qPCR Mix (Vazyme, Nanjing, China) on an ABI 7500 fast (Applied Biosystems, Carlsbad, CA, USA), and the primer sequences are listed in Table S2. The 2-△△Ct method was used to calculate the fold change, and GAPDH was used as an internal reference.

**Cell lines and cell culture**

Endometrial carcinoma cell lines (Ishikawa and HEC-1-A) were purchased from Shanghai Cell Bank, Chinese Academy of Sciences. Both cell lines were subjected to DNA tests and authenticated by authorized organizations. Ishikawa cells were cultured in RPMI 1640 medium (Biological Industries, Beit-Haemek, Israel) with 10% fetal bovine serum (Gibco, USA), whereas HEC-1-A cells were cultured in McCoy’s 5A medium (Biological Industries, Beit-Haemek, Israel) with 10% fetal bovine serum. All cells were cultured at 37°C in 5% CO2 with saturated humidity and routinely tested negative for mycoplasma.

**Cell proliferation assay**

We seeded 2 × 103 cells into 96-well plates and incubated them for 24 h, 48 h, 72 h and 96 h separately. Cell proliferation assays were performed by Cell Counting Kit-8 kits (ApexBio, Houston, USA) according to the manufacturer’s protocols. After two hours of incubation, the OD450 and OD630 were measured by a microplate reader (BioTek Instruments, ELx808, Winooski, VT, USA). The value of OD450 subtracted from that of OD630 was considered the final absorbance value of each well.

Cell viability was also evaluated by 5-ethynyl-2’-deoxyuridine (EdU) incorporation assays. We chose an EdU kit (C10310–1, RiboBio, Guangzhou, China) to detect the DNA synthesis of EC cells according to the manufacturer's protocol. A total of 7×103 cells were cultured in 96-well plates and treated with EdU solution (50 µM) for 2 h. Finally, we used a fluorescence microscope (Nikon Eclipse Ti-S, Nikon Ltd, Japan) to acquire and analyse images at an original magnification of 40×.

**Transwell assay**

For the migration assays, 5 × 104 cells were suspended in 200 µl serum-free medium and placed into the upper chambers (Corning, NY, USA), while 1 × 105 cells were added to the upper chamber for the invasion assays. We precoated the upper chamber with 40 µl Matrigel (BD Biosciences, San Jose, CA, USA, 1:10) for the invasion assay. RPMI 1640 or McCoy’s 5A medium with 20% fetal bovine serum was added to the bottom chambers. After 24 hours (migration assays) or 48 hours (invasion assays) of incubation at 37°C, the cells on the membrane were fixed with 4% formaldehyde for 15 min and stained with Giemsa stain (Leagen, Beijing, China) overnight. Then, we wiped off the cells on the upper surface thoroughly with cotton swabs. A microscope (20× magnification) was applied to count the cells infiltrated into the lower chamber surface, and cell numbers from five random fields of view were counted.

**RNA immunoprecipitation-seq (RIP–seq)**

RIP experiments, high-throughput sequencing and data analysis were performed by Seqhealth Tech (Wuhan, China). RIP assays were carried out on Ishikawa cells. The cells were lysed, and the lysis samples for immunoprecipitation reactions were incubated with anti-IGF2BP3 antibody (ab177477, Abcam, USA) or rabbit IgG (Cell Signaling Technology). The library products were enriched, quantified and finally sequenced on the Illumina PE150 platform.

**RNA sequencing**

The sample of LINC00958-silenced Ishikawa cells (sh-LINC00958, n=3) and negative control cells (sh-NC, n=2) were sent to Shanghai Sangon Biotech of China for library construction, sequencing, data preprocessing and gene mapping. Sequencing was performed on the DNBSEQ-T7 platform for the generation of raw data. Genes were considered significantly differentially expressed if the P value < 0.05 and |log2FC| > 1.
