# Supplementary figures and images for: IGF2BP3 enhances the mRNA stability of E2F3 by interacting with LINC00958 to promote endometrial carcinoma progression
Source: Cell Death Discov. 2022 Jun 8;8:279. doi: 10.1038/s41420-022-01045-x (PMC9177600; doi:10.1038/s41420-022-01045-x)

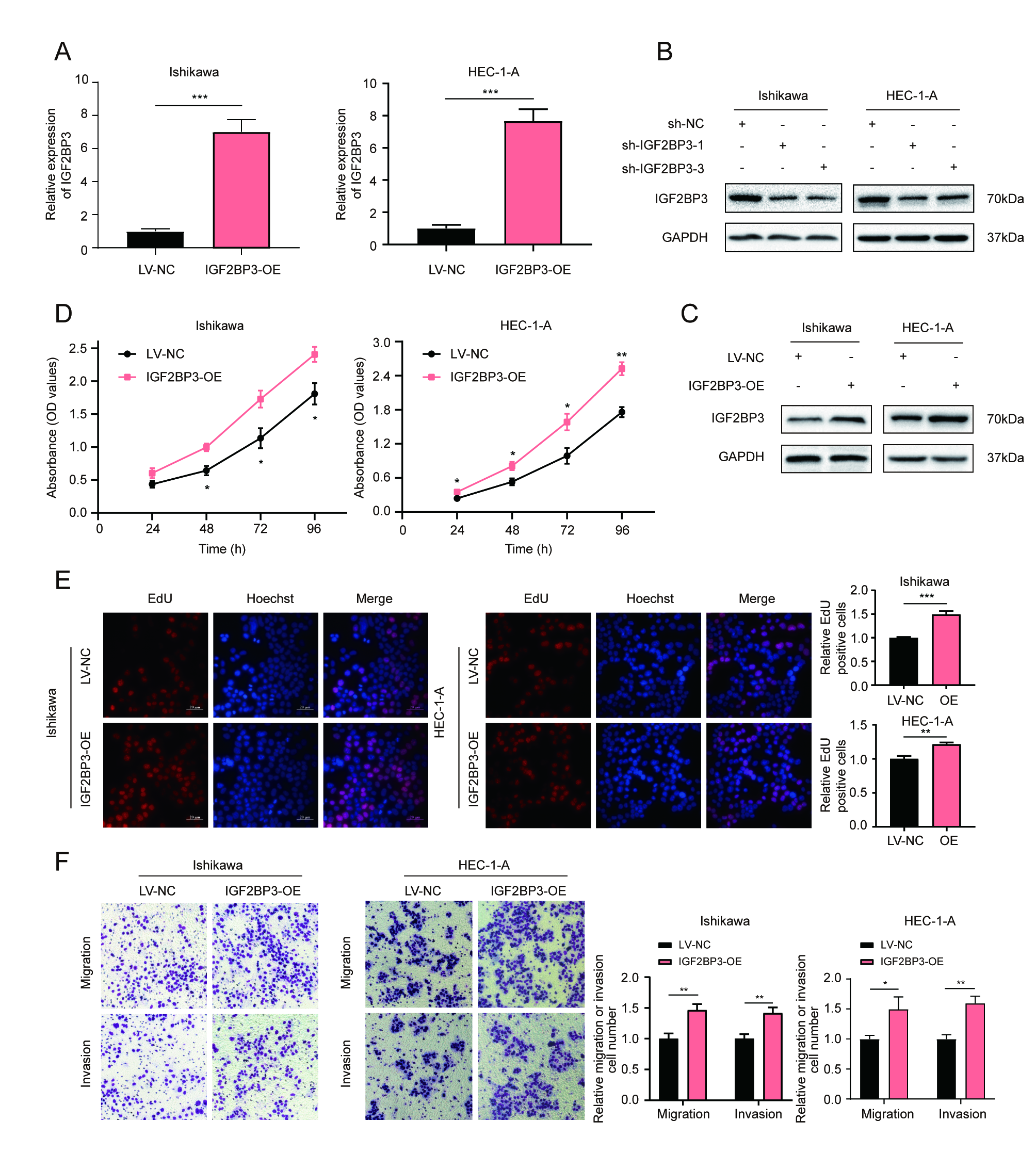

Supplement: Supplementary file 5 — Supplementary Figure 1 [file 41420_2022_1045_MOESM5_ESM.tif]

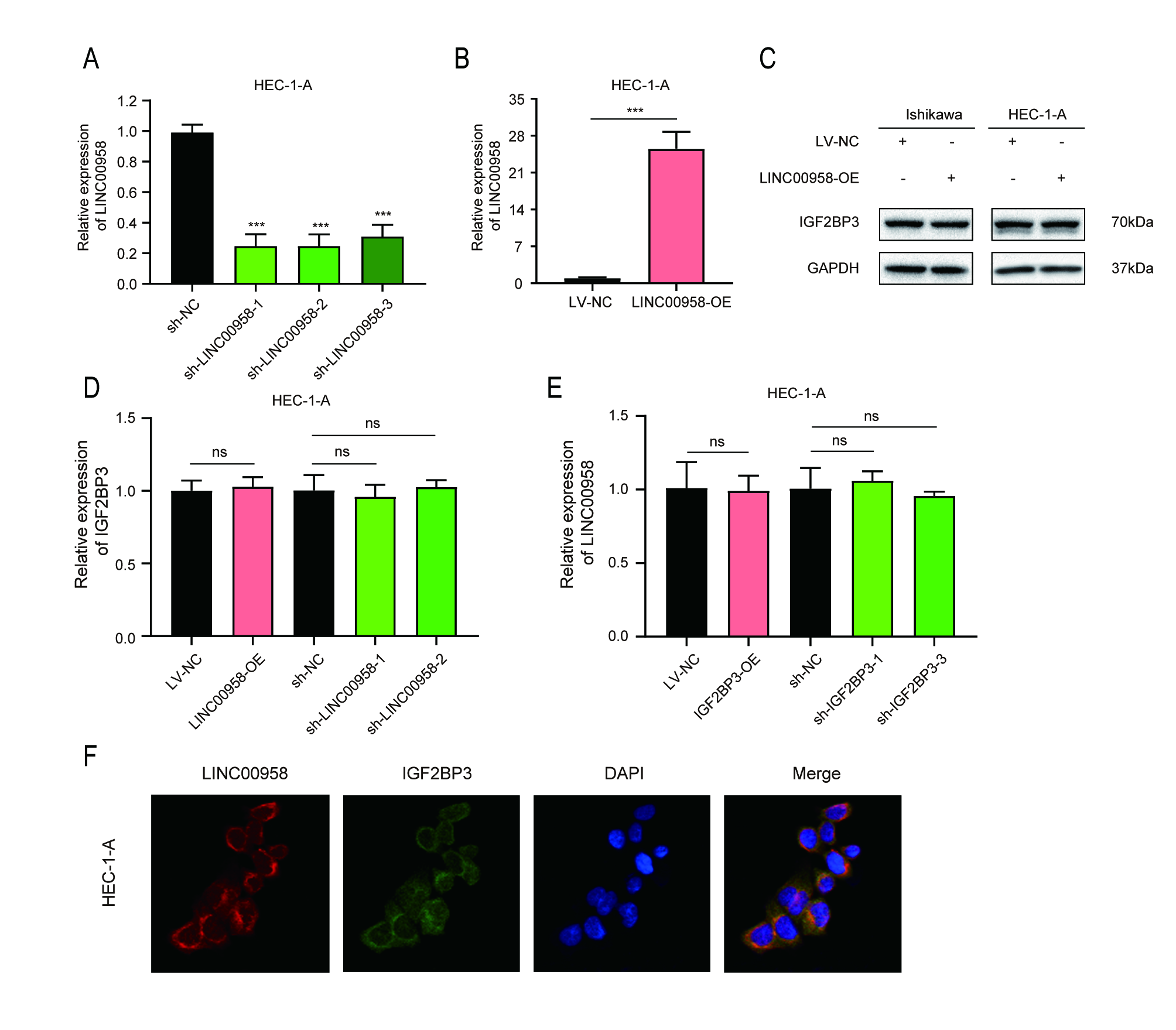

Supplement: Supplementary file 6 — Supplementary Figure 2 [file 41420_2022_1045_MOESM6_ESM.tif]

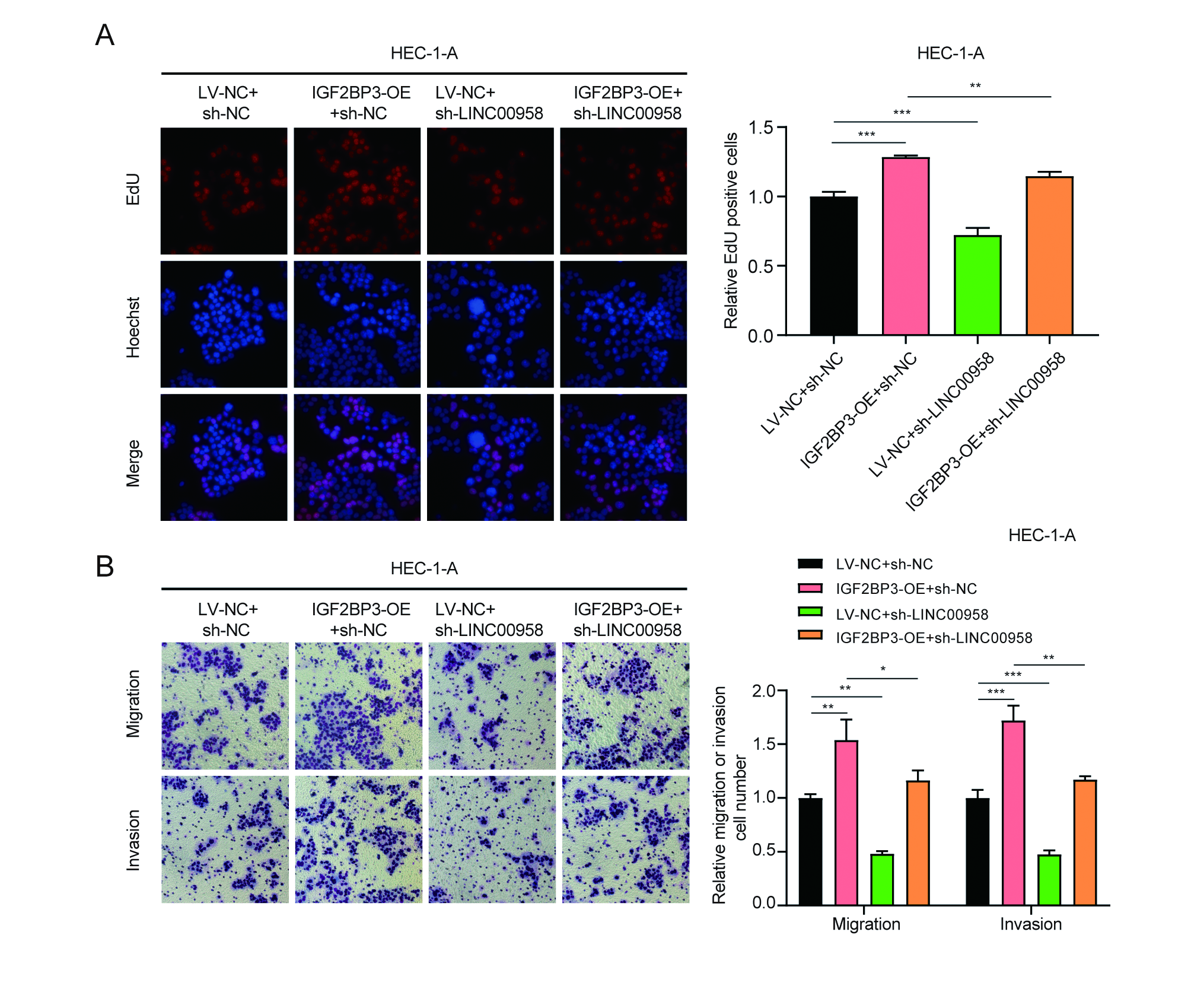

Supplement: Supplementary file 7 — Supplementary Figure 3 [file 41420_2022_1045_MOESM7_ESM.tif]

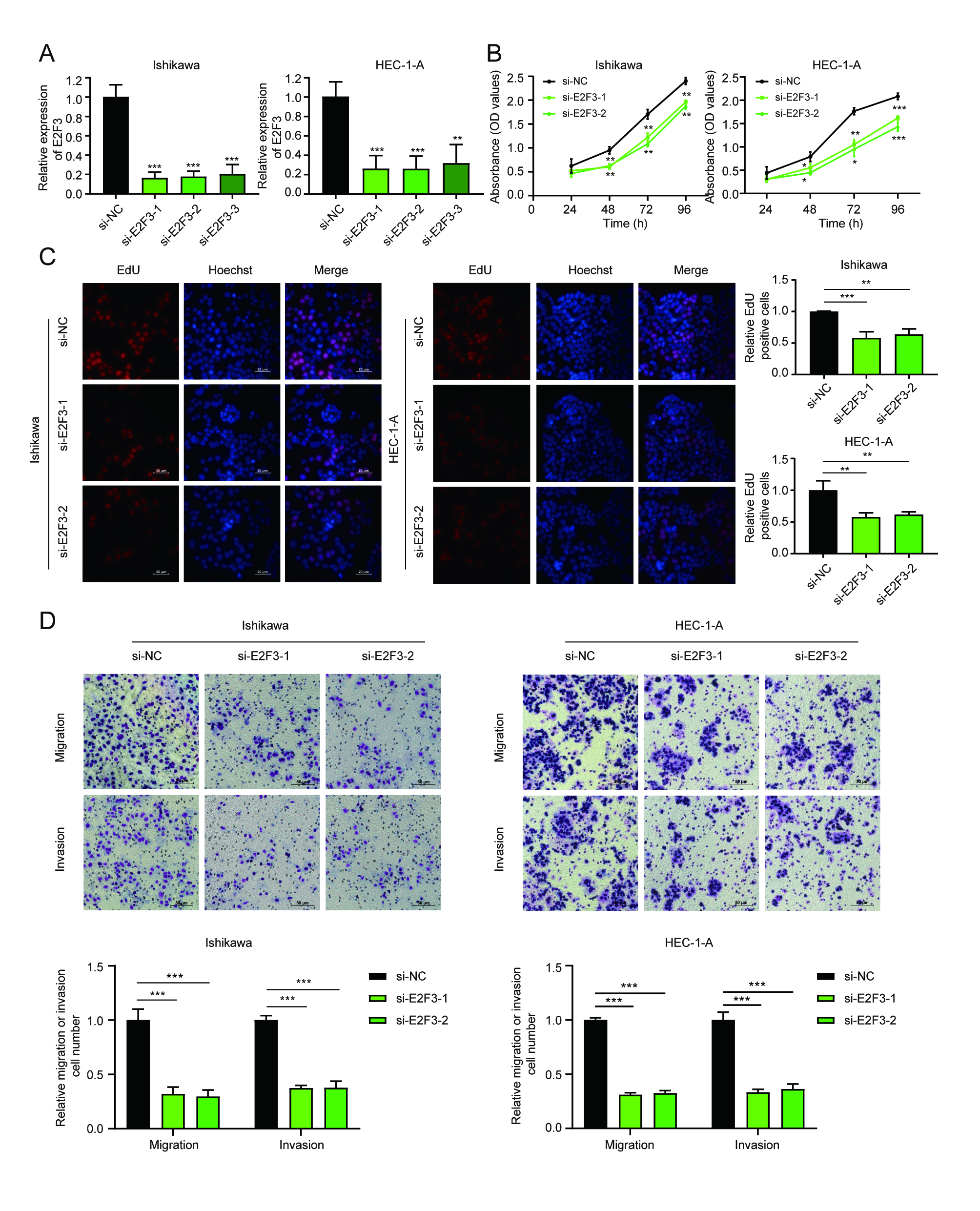

Supplement: Supplementary file 8 — Supplementary Figure 4 [file 41420_2022_1045_MOESM8_ESM.tif]

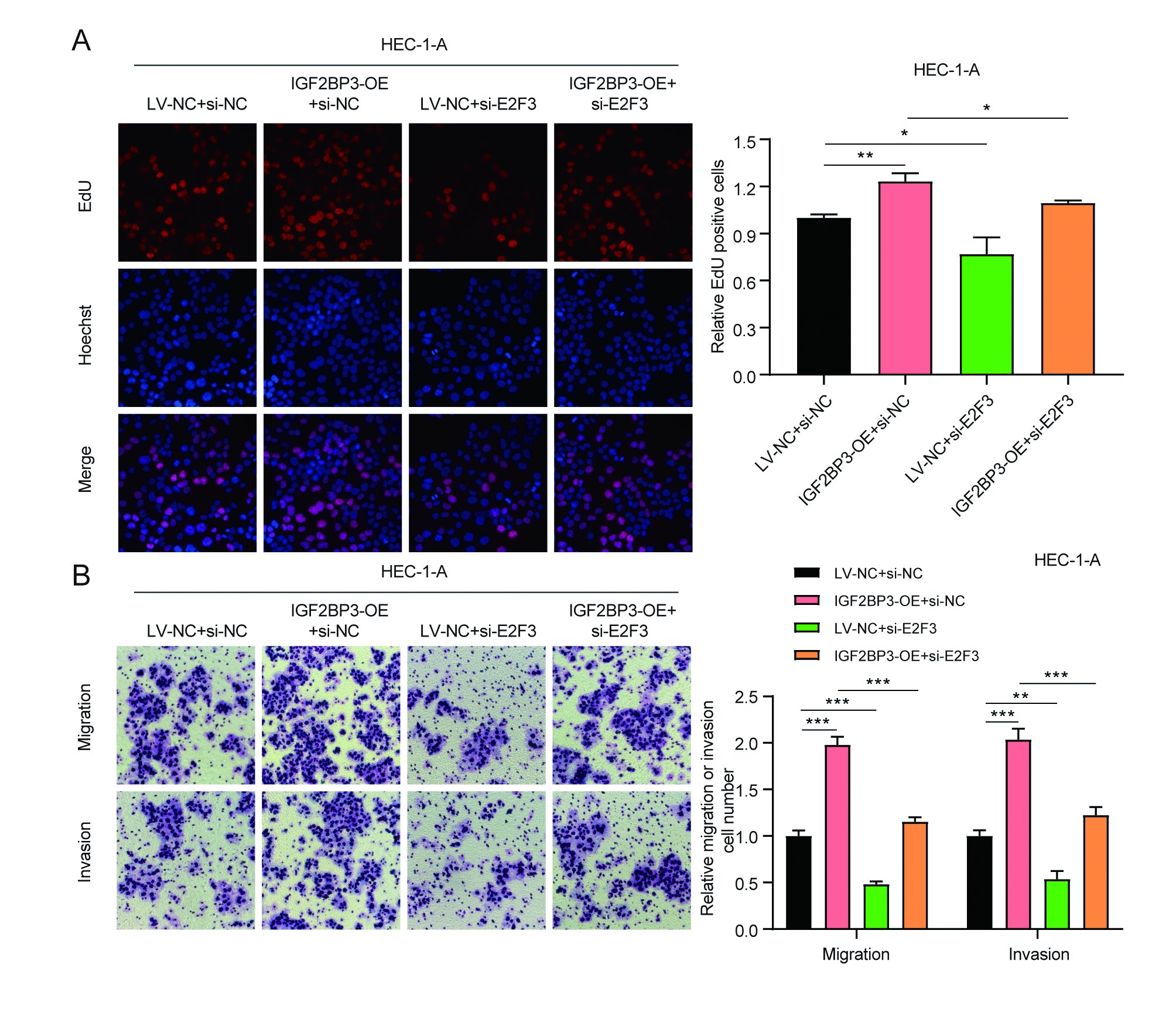

Supplement: Supplementary file 9 — Supplementary Figure 5 [file 41420_2022_1045_MOESM9_ESM.tif]
